# A Long-Standing Hybrid Population Between Pacific and Atlantic Herring in a Subarctic Fjord of Norway

**DOI:** 10.1093/gbe/evad069

**Published:** 2023-04-30

**Authors:** Mats E Pettersson, Angela P Fuentes-Pardo, Christina M Rochus, Erik D Enbody, Huijuan Bi, Risto Väinölä, Leif Andersson

**Affiliations:** Department of Medical Biochemistry and Microbiology, Uppsala University, Uppsala, Sweden; Department of Medical Biochemistry and Microbiology, Uppsala University, Uppsala, Sweden; Department of Medical Biochemistry and Microbiology, Uppsala University, Uppsala, Sweden; Department of Medical Biochemistry and Microbiology, Uppsala University, Uppsala, Sweden; Department of Medical Biochemistry and Microbiology, Uppsala University, Uppsala, Sweden; Finnish Museum of Natural History, University of Helsinki, Helsinki, Finland; Department of Medical Biochemistry and Microbiology, Uppsala University, Uppsala, Sweden; Department of Veterinary Integrative Biosciences, College of Veterinary Medicine and Biomedical Sciences, Texas A&M University, College Station, Texas

**Keywords:** gene-flow, Atlantic herring, Pacific herring, hybridization, subarctic

## Abstract

Atlantic herring (*Clupea harengus*) and Pacific herring (*C. pallasii*) are sister species that split from a common ancestor about 2 million years ago. Balsfjord, a subarctic fjord in Northern Norway, harbors an outpost population of Pacific herring within the range of the Atlantic herring. We used whole genome sequencing to show that gene flow from Atlantic herring into the Balsfjord population has generated a stable hybrid population that has persisted for thousands of generations. The Atlantic herring ancestry in Balsfjord was estimated in the range 25–26%. The old age and large proportion of introgressed regions suggest there are no obvious genetic incompatibilities between species. Introgressed regions were widespread in the genome and large, with some in excess of 1 Mb, and they were overrepresented in low-recombination regions. We show that the distribution of introgressed material is non-random; introgressed sequence blocks in different individuals are shared more often than expected by chance. Furthermore, introgressed regions tend to show elevated divergence (*F*_ST_) between Atlantic and Pacific herring. Together, our results suggest that introgression of genetic material has facilitated adaptation in the Balsfjord population. The Balsfjord population provides a rare example of a stable interspecies hybrid population that has persisted over thousands of years.

**Significance**

The existence of a hybrid population of Atlantic and Pacific herring in Balsfjord, Northern Norway, has been known for some time. However, the nature of its genetic makeup has not been understood in detail, since it has only been studied using sparse-marker data. Here, we use whole-genome sequencing and show that the Balsfjord population is stable, established thousands of generations ago, and that genetic material derived from Atlantic herring is enriched in narrow regions in the genome, suggesting a role in adaptation.

## Introduction

Adaptive genetic variation in a population can originate from standing genetic variation, de novo mutations, or gene flow from other populations. The traditional view on speciation theory predicts that populations diverge when separated, and that speciation occurs at the point where reproductive isolation is achieved and gene flow ceases (Mayr 1999). However, with the widespread availability of genomic data across a broad range of taxa (Rieseberg et al. 1999; Sankararaman et al. 2014; Lamichhaney et al. 2015), it has become evident that many species exchange genes, or has experienced gene flow from other species in the past. As a consequence, the potential for introgressive hybridization to contribute to evolutionary change is becoming increasingly apparent (Taylor and Larson 2019). However, the role, if any, that these between-species gene flow events have on adaptation, in particular around hybridization zones, remains incompletely understood. Estimating the adaptive benefit of gene flow, in the absence of direct experimental evaluation of fitness, requires detailed information concerning the genomic landscape of the populations involved, which is often challenging in wild populations. In this study, we present suggestive evidence for adaptive gene flow from the Atlantic herring (*Clupea harengus*) to an isolated population of Pacific herring (*C. pallasii*) occurring in a subarctic fjord in the Northeast Atlantic Ocean.

**Fig. 1.**
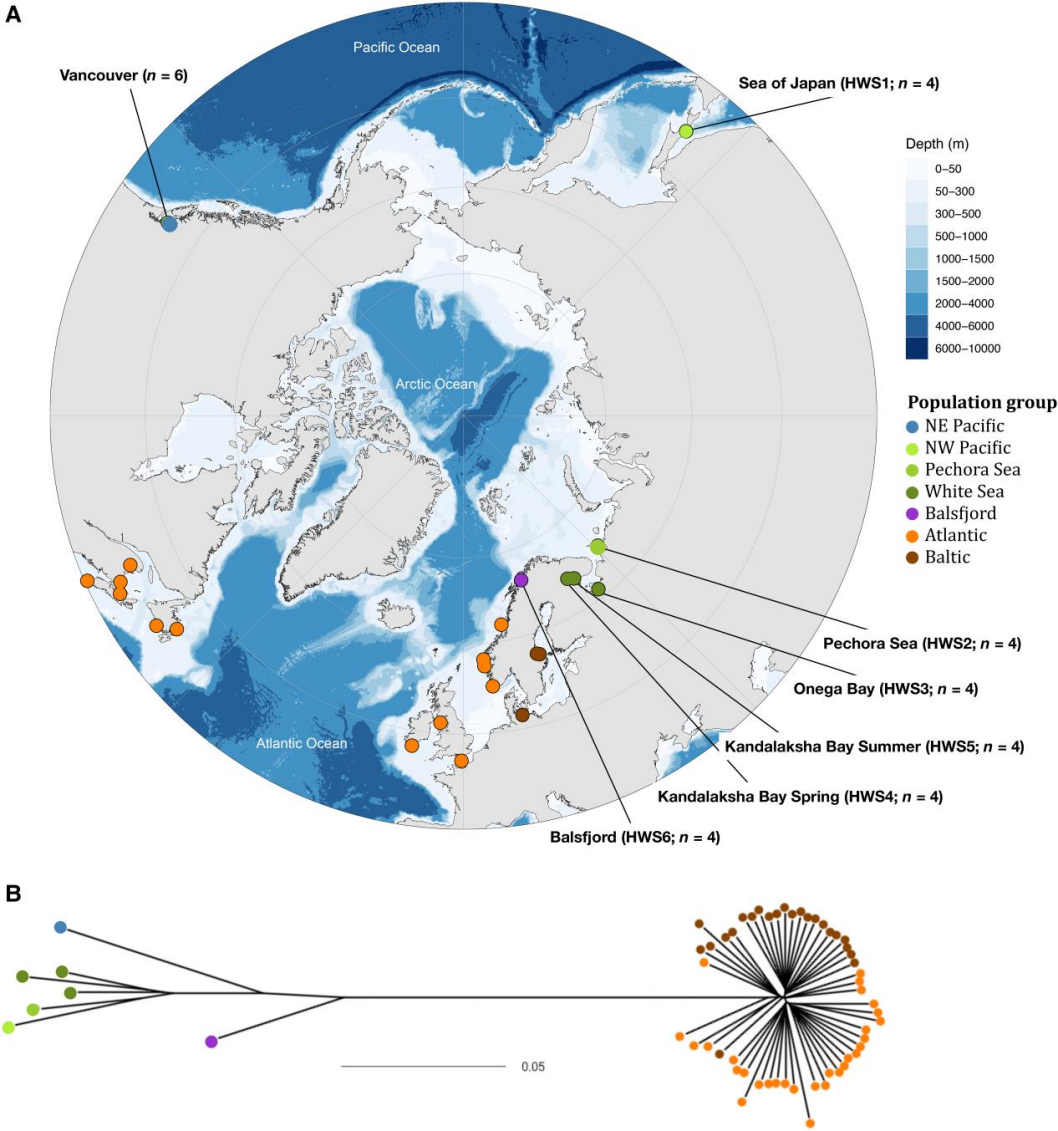
Sample locations and genetic distances among Pacific and Atlantic herring populations from different oceans and sea areas. *A)* Location of samples of Pacific herring from Balsfjord, European Russia (Pechora and White Seas), and from the NW Pacific (Sea of Japan) and NE Pacific (Vancouver) (supplementary table S1, Supplementary Material online). For detailed information on locations of Atlantic and Baltic herring samples, see Han et al. (2020). *n* denotes the number of individual samples for the respective location The base map was created using ggOceanMaps (https://mikkovihtakari.github.io/ggOceanMaps/), using data from the National Centers for Environmental Information (https://www.ngdc.noaa.gov/mgg/global/relief/ETOPO1/docs/ETOPO1.pdf). *B)* Neighbor-joining tree based on allele frequency distances from 10^5^ randomly selected SNPs in poolseq data from 60 populations of Pacific and Atlantic herring. The scale represents the average difference in allele frequency per SNP. Populations are colored as in *a*.

Pacific and Atlantic herring are sister species, separated an estimated 2 Ma (Martinez-Barrio et al. 2016) most likely due to geographic isolation during glaciations. The species pair is part of what is known as the amphi-boreal fauna, a set of species that occupies the Northern Pacific and Atlantic Oceans, which are thought to have dispersed across the Arctic basin, that is the oceanic area enclosed by the Northern coast lines of Eurasia and North America, at various time points since the opening of the Bering Strait in the early Pliocene (∼5 Ma), with the peak exchange estimated to have occurred during the Middle Pliocene (3.5 Ma) (Vermeij 1991; Laakkonen et al. 2021). At present, their respective ranges span the northern parts of the two oceans extending southward to the Sea of Japan and California in the Pacific, and to the English Channel and South Carolina in the Atlantic, and to the subarctic margins in the northern parts of the basins. The Pacific herring also occurs in Europe (Svetovidov 1952; Jørstad 2004) (fig. 1*A*) in the arctic and subarctic waters of Russia (the White and Pechora Seas), where the two species have come to a post-glacial contact (Semenova and Stroganov 2018; Semenova 2020). A disjointed, fjord-based population of Pacific herring is also present further west in Balsfjord, close to Tromsø in Northwestern Norway, well above the polar circle but within the range of Atlantic herring of the Norwegian Spring Spawning (NSSH) stock (Jørstad et al. 1994; Laakkonen et al. 2013). The two herring species are characterized by and maintain differences in their spawning behavior, particularly in depth and substrate preference. The Pacific herring breeds on seaweed in the littoral zone while Atlantic herring breeds at greater depths on rock beds near the coast (Jørstad et al. 1994; Geffen 2009). Previous studies using low-density genetic markers including allozymes and mtDNA indicated that the Balsfjord population of Pacific herring has hybridized with the Atlantic herring, but the timing and impact of hybridization on fitness remain poorly known (Laakkonen et al. 2013, 2015). Despite hybridization, the Balsfjord herring are phenotypically a Pacific herring, as they have similar mortality and growth rates, fecundity, body size, and spawning behavior as other Pacific herring populations (Mikkelsen et al. 2018).

Here, we performed whole genome sequencing of Pacific herring from Balsfjord, the White Sea, Pechora Sea, and the Sea of Japan (fig. 1*A*), along with previously published Atlantic herring data (supplementary table S1, Supplementary Material online), to estimate the extent and genomic distribution of Atlantic introgression to the local Pacific herring genomes and to evaluate the potential contribution of gene flow to local adaptation. Having confirmed the admixed status of the Balsfjord population, we show that introgression largely occurred thousands of years ago and that the genomic distribution of introgressed material is non-random; the individual haploid genomes of Balsfjord herring carry introgressed Atlantic haplotypes with overlapping physical locations more often than expected by chance, and these introgressions are overrepresented in regions of high *F*_ST_ between the sister species.

## Results

We generated whole-genome sequencing data of Pacific herring from Balsfjord (Norway), four population samples, likewise of Pacific herring from arctic–subarctic waters of European Russia (White Sea and Pechora Sea) and a Northwest Pacific population from the Sea of Japan (fig. 1*A*, supplementary table S1, Supplementary Material online). For all populations, we sequenced both individual fish (*n*: 4–6; depth >10x) and pooled population samples (*n*: 35–57 per pool; depth >30x). To identify the origin of shared genetic ancestry, we analyzed this data along with previously published data from 53 population samples of Atlantic herring and one population sample of Pacific herring from Vancouver, Canada, as well as 49 individuals from a subset of the locations (Martinez-Barrio et al. 2016; Han et al. 2020) (supplementary table S1, Supplementary Material online). For most of the following analysis, we will rely on the four individuals from Balsfjord, the four individuals from the Sea of Japan, and eight of the Atlantic herring individuals. This triad forms the basis for detection of introgressions (see Methods for details), while the rest of the samples are used to provide context for the findings. In addition, we have low-coverage data from an additional 41 Balsfjord individuals. The low-coverage data cannot be used for our introgression detection method, due to lack of confident single nucleotide polymorphism (SNP) genotype calls, but has been analyzed in terms of admixture.

### Genetic Diversity in Atlantic and Pacific Herring

Previous studies using mitochondrial and allozyme markers found that the Pacific herring is composed of a northeast (NE) and a northwest (NW) lineage, the latter of which extends to Europe and shows further geographic subdivisions (Laakkonen et al. 2013). To evaluate whether genome-wide autosomal markers show the same pattern, we built a neighbor-joining tree based on allele frequency estimates of 10^5^ randomly selected SNPs derived from sequencing data of all Pacific and Atlantic herring population pools included in this study (*n* = 60) (fig. 1*B*). Within the Pacific herring, the NE Pacific (Vancouver) population is clearly separated from a clade composed of the NW Pacific + White and Pechora Seas. The distances among Pacific herring populations are a fraction of the deep genetic split between Pacific and Atlantic herring samples, but clearly larger than between any populations of Atlantic herring (fig. 1*B*). The Balsfjord branch falls between the Pacific and Atlantic clusters but closer to Pacific, consistent with the population being of Pacific descent, but with a fraction of admixed Atlantic genetic material.

We calculated genome wide diversity metrics (*F*_ST_, *d_xy_*, and *π*) using all individually sequenced herring (supplementary figs. S1 and S2, Supplementary Material online, supplementary table S2, Supplementary Material online). Nucleotide diversity is higher in the Balsfjord herring (*π*=0.35%) than any pure Atlantic (0.25%) or Pacific (0.28%) population (supplementary table S2, Supplementary Material online). The high *π* in Balsfjord is largely the effect of the combined contribution of alleles from the two distinct herring species, essentially incorporating a noticeable part of Atlantic versus Pacific *d_xy_* into its internal diversity, in accord with the pattern in *π* reported from the mixing of the distinct Pacific and Atlantic mitochondrial lineages in the same population (Laakkonen et al. 2015).

### Genetic Structure and Mixed Ancestry of the Balsfjord Herring

**Fig. 2.**
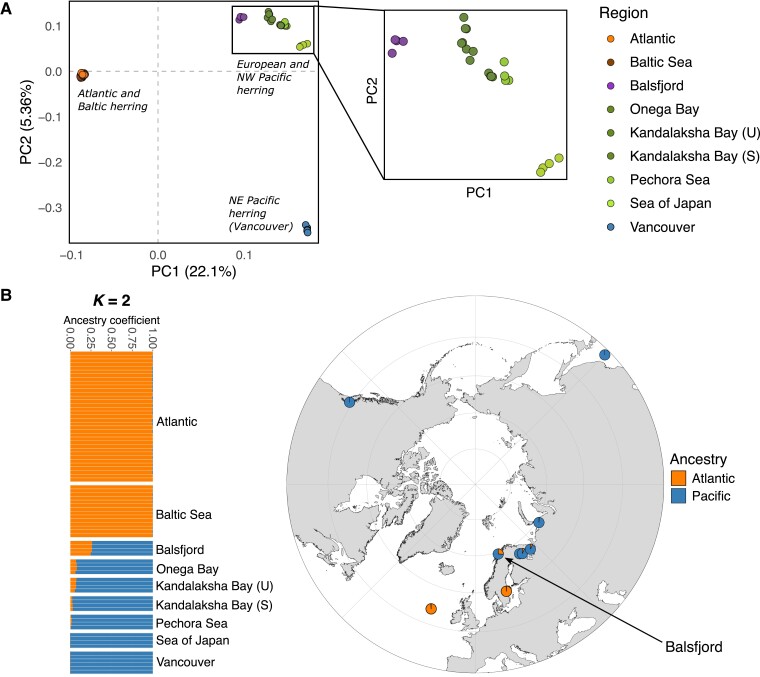
Population structure and admixture of Atlantic and Pacific herring. *A)* PCA plot based on 2.9 million SNPs, from data on 79 individually sequenced fish. Inset, zoom-in to the Balsfjord, White Sea, Pechora Sea, and NW Pacific (Sea of Japan) samples. In the Kandalaksha Bay samples, “U” and “S” indicate summer- and spring-spawning populations, respectively. *B)* Graphical representation of ancestry coefficients estimated with the program sNMF (Frichot et al. 2014). Individual ancestry coefficients for *K* = 2 are shown as bar plots (left), and average ancestry coefficients per location are shown as pie charts on a map (right), both to illustrate the extent of admixture at the contact zone in Balsfjord. In the bar plots, each row corresponds to an individual, and the size of each color division represents the proportion of an individual's genome that likely originated from Pacific and Atlantic herring.

We explored population genetic structure using Principal Component Analysis (PCA) (fig. 2*A*) and admixture analysis (fig. 2*B*) based on SNPs of 79 individual Pacific and Atlantic herring (2.9 million SNPs retained after quality control and linkage equilibrium filters). Samples of Pacific herring clustered by geographic location in the first two principal components (PCs), while populations of Atlantic herring formed a single cluster, consistent with the neighbour-joining tree (fig. 1*B*). PC1 provides a clear distinction between Atlantic and Pacific herring. PC2 in turn distinguishes the Northeast Pacific samples, from Vancouver, from all others, including the NW Pacific (Sea of Japan) and those from European Russia. The Balsfjord population clustered with the latter group, which is the (other) European Pacific herring and the NW Pacific population.

Admixture analysis based on the sNMF model (Frichot et al. 2014) identified the most likely number of ancestral populations (*K*) as either two or three, according to their minimum cross-entropy criterion values (supplementary fig. S3, Supplementary Material online). Using *K* = 2, the results capture the separation along PC1 identified by the PCA analysis, with the White Sea (Onega and Kandalaksha Bays) samples displaying a small, but detectable fraction of Atlantic material, which was not observed in the geographically adjacent sample from Pechora Sea or that from the Sea of Japan (NW Pacific) (fig. 2*B*).

An extended analysis of admixture proportions including a larger number of Balsfjord individuals sequenced to a low depth (<1X, *n* = 41) (supplementary fig. S4, Supplementary Material online) confirmed the observations made with the high coverage data, and further indicated that there is some variation in the proportion of Atlantic ancestry among Balsfjord individuals. In this dataset combining high and low coverage individuals, the most likely number of ancestral populations was confirmed as *K* = 2, according to the Evanno method (supplementary fig. S4, Supplementary Material online). The estimated ancestry proportions for each sample are listed in supplementary table S3, Supplementary Material online.

### No Indication of Mito-nuclear Incompatibility in the Balsfjord Herring

Hybridization can introduce incompatibilities between the mitochondrial and nuclear genome, leading to discordance in the level of introgression (Burton and Barreto 2012). We evaluated the potential for mito-nuclear incompatibility in the Balsfjord herring by comparing the frequency of mitochondrial DNA introgression with that of the nuclear genome. Previous work using individual sequencing of the mitochondrial *cytochrome b* gene and allozyme genotypes from the same samples analyzed here did not detect evidence for mito-nuclear incompatibility: there were no mito-nuclear disequilibria, and nuclear and mitochondrial introgression frequencies were very close (Laakkonen et al. 2015). Using pairwise genetic distance calculations, we found that one out of four whole-genome-sequenced fish from Balsfjord (HWS61) carried the Atlantic mitochondrial haplotype and the other three carried Pacific haplotypes (supplementary fig. S5, Supplementary Material online). In the pooled samples, the frequencies of SNPs fixed for different alleles in Pacific and Atlantic herring had an average of 27% Atlantic mitochondrial allele frequency in Balsfjord (supplementary fig. S5, Supplementary Material online), around 10% in the two pooled samples from the White Sea (HWS3 and HWS5), while all other samples were close to fixation for Pacific haplotypes (supplementary fig. S5, Supplementary Material online). In conclusion, in agreement with a previous more detailed analysis (Laakkonen et al. 2015), we find no evidence of a mito-nuclear incompatibility between Atlantic and Pacific herring. In the Balsfjord population, Atlantic and Pacific mitochondrial haplotypes appear in frequencies matching the admixture proportions of the nuclear genome.

### Detection of Introgressed Haplotypes

Using four individuals from the NW Pacific (Sea of Japan) and eight specimens of Atlantic herring from Bergen (Norway) as references of Pacific or Atlantic ancestry, respectively, we scanned the four Balsfjord individuals for introgressed regions. First, we phased genotype data using Beagle v4.0 (Browning and Browning 2007) to obtain locally consistent haploid genomes.

Then, we screened each Balsfjord haplotype (*n* = 8) for blocks that were significantly closer to the most similar Atlantic haplotype than to the most similar NW Pacific haplotype and vice versa, for Pacific or Atlantic similarity. To achieve this, we calculated the number of nucleotide differences from each haploid Balsfjord genome, in blocks of 20 kb each, with block size chosen to fit an expected number of 100 SNPs per window, to the closest Pacific and Atlantic reference haplotypes, respectively. Long-range phasing is not needed for this purpose—each 20 kb block is analyzed independently, which mitigates the inherent problems of reference-free phasing in a limited set of individuals. The ratio of the two distances would then form the scoring metric for the block of interest. The block-wise ratios were assessed by comparison to the genomic average (for details see Methods), to identify significant outliers. Windows that passed the significance threshold will hereafter be referred to as high-similarity regions (HSRs) (supplementary fig. S6, Supplementary Material online). Due to the evolutionary histories of the populations involved, Atlantic HSRs are likely the results of introgression, since any material shared by incomplete lineage sorting would have accumulated notable variation since divergence and highly conserved regions would also be found in the Pacific reference set. Pacific HSRs, on the other hand, may consist of a mix of introgressed blocks caused by gene flow and regions that have been under unusually strong purifying selection in Pacific herring. The latter is a possibility since Balsfjord and other populations of Pacific herring share common ancestry well after the split between Atlantic and Pacific herring.

On average, haplotypes from Balsfjord were more similar to Pacific than Atlantic genomes; the mean genome-wide distance to Atlantic haplotypes divided by the mean distance to Pacific haplotypes and then averaged across the eight haploid genomes (i.e., the mean of the individual ratios), was 1.45, which can be thought of as a reflection of the relative branch lengths from Balsfjord to the Atlantic cluster and the NW Pacific cluster, respectively (fig. 1*B*). In total, we identified 4,747 Atlantic and 4,748 Pacific HSRs across all haplotypes (supplementary table S4, Supplementary Material online). The total length of Atlantic HSRs called per haploid genome ranged from 37.5 to 44.7 Mb, or around 5% of the genome. This is lower than the estimated admixture contribution from Atlantic herring, which is expected since this analysis only considers regions where the signal is unambiguous across a full 20 kb window.

### Distribution of Introgressed Haplotypes

In order to evaluate whether the identified HSRs occur randomly across the genome, we compared their genomic positions to a null distribution of evenly spread regions across the genome. In general, HSRs occur across all chromosomes, with most individual HSRs occurring in only one of the eight haplotypes. There are 13 regions, with a total size of 0.62 Mb, where all eight haplotypes from the four sequenced Balsfjord individuals presumably originated from Atlantic herring (supplementary fig. S7, Supplementary Material online) and 23 regions, also covering a total of 0.62 Mb, where they all carry Pacific HSRs (supplementary table S5, Supplementary Material online). We largely concentrate on the subset of 8-fold recurring Atlantic HSRs in the following analyses. The size, measured in total number of bases covered, of overlapping HSRs in all eight haploid genomes, for both Atlantic and Pacific HSRs, was much larger than expected under a random distribution model. We assume, firstly, that Balsfjord herring can be represented by a randomly mating Wright–Fisher population, and, secondly, that HSRs in each haplotype are independent vis-à-vis other haplotypes, and, lastly, that each position in the callable genome is equally likely to harbor an HSR. Then, the expected likelihood for any given nucleotide position to be within an HSR in all eight haploid genomes is the product of the fractions (*f_i_*) that is found inside HSRs in each haploid genome. Multiplying this likelihood by the size of the callable genome, that is the regions where the parent species are sufficiently differentiated to call an introgression with confidence, (*S*_callable_; the size of the genome with inter-species *F*_ST_ >0.15), yields the expected number of nucleotide positions where all eight haploid genomes carries an HSR (*n*_8_):


(1) $$n_{8}=\left(\prod_{i=1}^{8}\;f_{i}\right)*S_{\mathrm{callable}}$$

where *S*_callable_ = 468 Mb, out of a total assembly size of 786 Mb (including unplaced scaffolds) and *f_i_* values of approximately 0.10 (Atlantic) and 0.06 (Pacific), this yields an expected *n*_8_ of 3.3 bases for Atlantic HSRs—corresponding to roughly a 1 in 6,000 chance of finding one overlapping 20 kb block in the genome—and 0.05 bases for Pacific HSRs. This is in stark contrast to the observed data (both 0.62 Mb, or 31 blocks of 20 kb), resulting in huge overrepresentations, *M*-values of 17.5 and 23.5, respectively [*M* = log_2_(Observed/Expected)]. The expected number of bases with other overlap counts (*k*) can be calculated analogously, from a binomial distribution, by assuming they each have the same HSR fraction:


(2) $$n_{k}=\left(\begin{array}{c} 8\\ k \end{array}\right)F_{i}^{k}*(1-F_{i}{)}^{8-k}*S_{\mathrm{callable}};1\leq k\leq 8,$$

where *F_i_* is the highest of the fractions *f_i_*. The corresponding *M*-values are shown in supplementary figure S8, Supplementary Material online, indicating that the excess of observed overlap is consistently rising with increased introgression coverage.

Furthermore, in the regions where all eight haplotypes are from the same species, the allele frequencies in the pooled sample from Balsfjord are similar to those found in either Atlantic or Pacific reference pools, depending on the origin of the HSR (supplementary figs. S9 and 10, Supplementary Material online), indicating that the pattern detected in the individual samples is representative of the population. The marked over-representation of regions in which introgressed haplotypes occur at a high frequency, compared with the expectation based on a random model (supplementary fig. S8, Supplementary Material online), suggests that the introgressed material might confer an adaptive advantage in the Balsfjord population.

### Verification of Recurring HSRs by *Twisst* Analysis

**Fig. 3.**
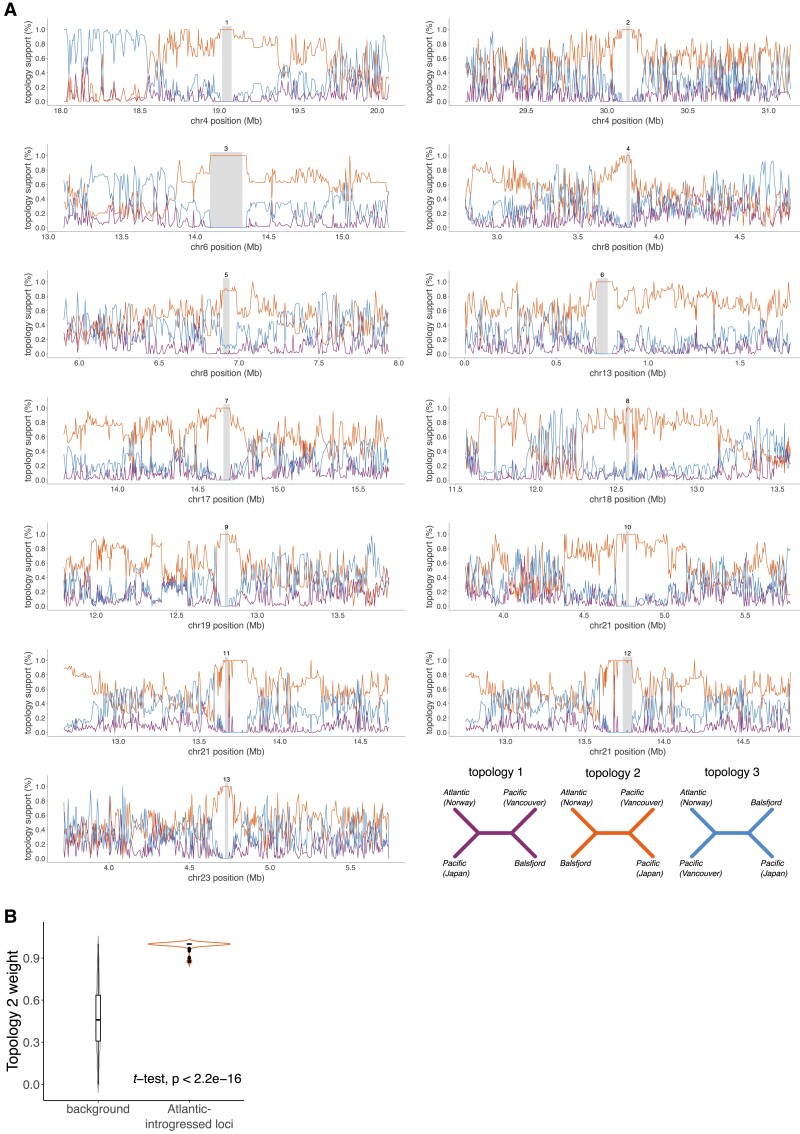
*Twisst* results for regions harboring 8-fold recurring Atlantic HSRs in Balsfjord herring. *A) Twisst* topology support around regions that were predicted to harbor 8-fold recurring Atlantic HSRs. The called 8-fold recurring regions are indicated by grey rectangles. *B)* Average support for topology 2 in the background and in 8-fold recurring Atlantic HSRs, respectively.

For the genomic regions where the bespoke method described above indicated that all Balsfjord haploid genomes carried an Atlantic HSR, we expect the phylogenetic topology to differ from the species phylogeny. To test this expectation, we used *Twisst*, a method for topology weighting by iterative sampling of subtrees (Martin and Van Belleghem 2017). *Twisst* evaluates, across the genome, the support for competing phylogenetic hypotheses among closely related taxa. The method generates phylogenetic trees in sliding windows across the genome, and calculates the fraction of subtrees that match each taxon topology. As seen in figure 3*A*, all 13 regions with 8-fold recurring HSRs show support for the topology which groups Balsfjord and Atlantic samples, with 99% median support (fig. 3*B*), a highly significant increase compared to the rest of the genome (*P*_*t*_test_ < 2*10^−16^).

### Neighboring Regions on Chromosome 4 With Different Origins

**Fig. 4.**
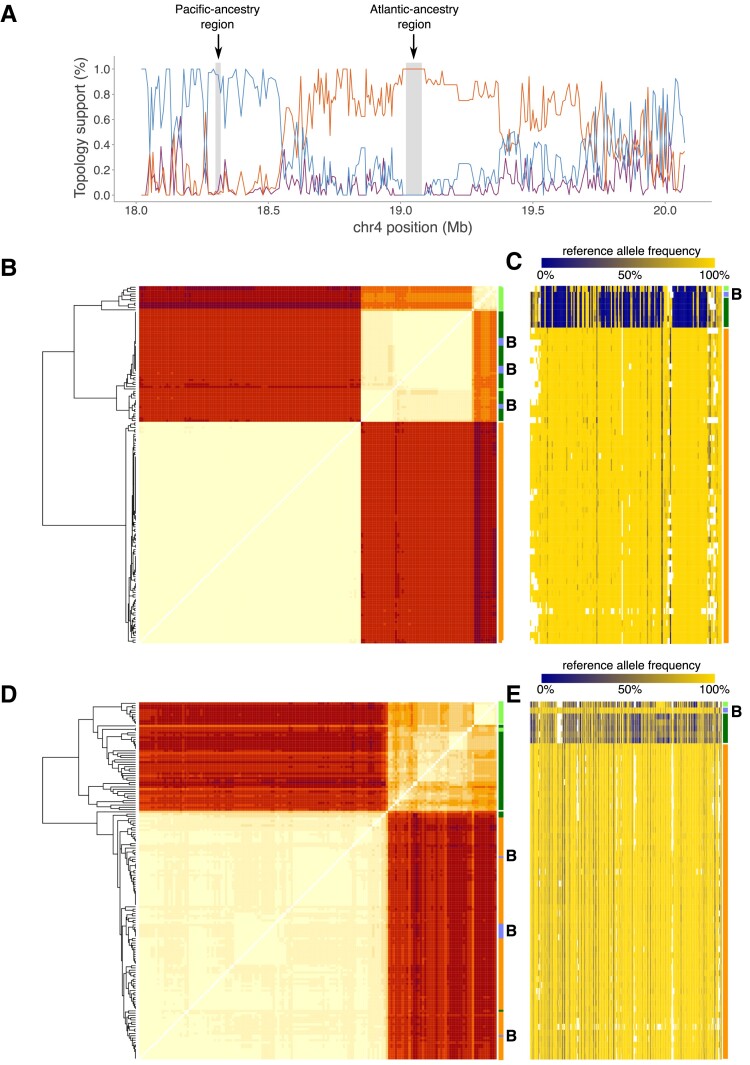
Two neighboring regions on chromosome 4 with different origin among Balsfjord haplotypes. These regions, only 700 kb apart, comprise one with Pacific ancestry (Chr4:18.30–18.32 Mb), and one with Atlantic ancestry (Chr4:19.02–19.08 Mb). *A*) Topology support (%) for Balsfjord individuals inferred by *Twisst* in windows of 100 SNPs along the region Chr4:18.0–20.0 Mb. Line colors match each of the topologies examined, with colors as in figure 3. The gray shaded areas highlight the 8-fold recurring HSRs. Panels *B* and *D* show heatmaps representing the Hamming (or Edit) distances between individual haplotypes for the region with Pacific [*B*] and Atlantic [*D*] ancestries, respectively. Cell colors indicate pairwise distance, normalized by the largest distance. Panels *C* and *E* show heatmaps that represent the reference allele frequencies of pooled samples for the region in *B* (Pacific) and *D* (Atlantic). The purple rectangles and capital B indicate Balsfjord herring haplotypes; the bars on the side indicate Northeast Pacific (light green), Northwest Pacific and White Sea (dark green) or Atlantic (orange) haplotypes.

A narrow region on Chr. 4 provides an example of how nearby genomic regions have opposite patterns in the examined Balsfjord samples. In this region, all eight Balsfjord haplotypes switch from Pacific HSR haplotypes in the interval 18.30 to 18.32 Mb to Atlantic HSR haplotypes in the interval 19.02 to 19.08 Mb (fig. 4). This region has a low recombination rate and this pattern is unlikely to occur by chance, although it could be facilitated by a local recombination hotspot located between 18.30–18.40 Mb (see supplementary fig. S1, Supplementary Material online in Pettersson et al. 2019). Notably, in the 18.30 to 18.32 Mb interval, hierarchical clustering of Hamming distances between haplotypes in the two regions revealed very little within-species divergence (*π*): 0.02% within all individual Atlantic herring samples and 0.05% in the European Pacific herring individuals. These low levels of nucleotide diversity are roughly 1/10 of the genomic average (supplementary table S2, Supplementary Material online), and indicative of selection, either positive or purifying, acting in the recent past for both sister species. On the other hand, *d_xy_* in this region is 0.47%, well within the background range, which indicates that different haplotypes have been favored in the two species.

In contrast, for the 19.02 to 19.08 Mb interval, only Atlantic herring shows signs of a sweep (intra-clade diversity 0.05%, compared to 0.20% for the European Pacific clade) (fig. 4*B* and *C*), again without affecting *d_xy_* (0.45%). The pooled samples show a matching switch in allele frequencies (fig. 4*d* and *e*). The first interval contains only one gene: *KCNA10* (potassium voltage-gated channel subfamily A member 10), the second interval covers most, but not all, of *GRM4* (glutamate metabotropic receptor 4).

### Overlapping HSRs Rarely Share Close Ancestry

**Fig. 5.**
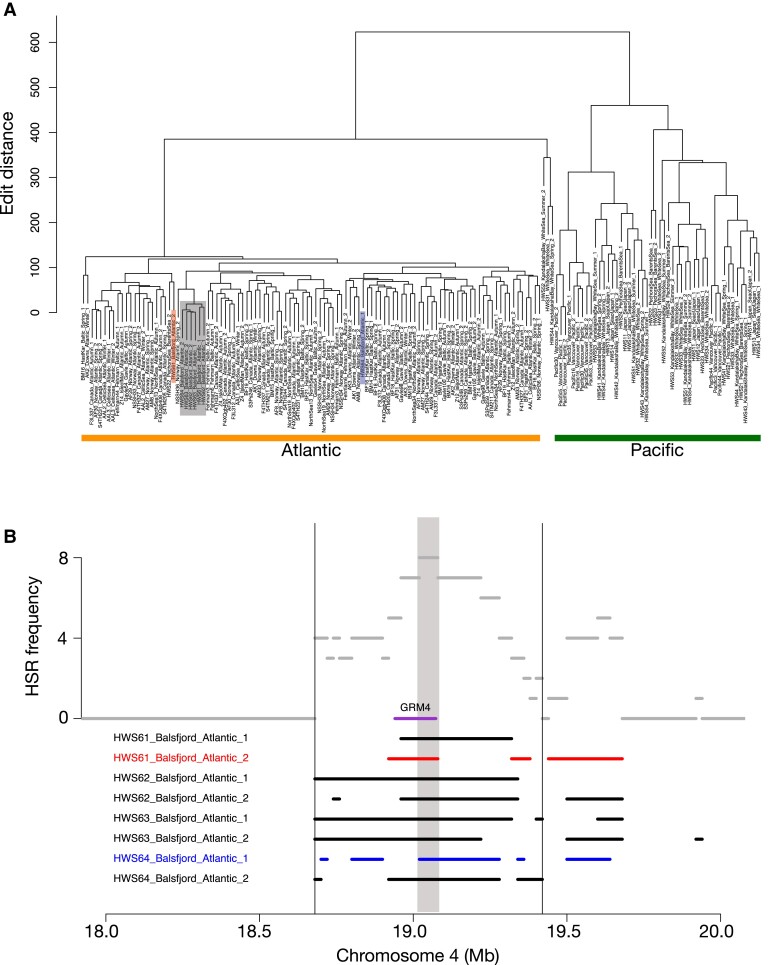
Eight overlapping HSRs on chromosome 4:19.0–19.1 Mb are derived from three different Atlantic haplotypes. *A)* A hierarchical clustering dendrogram based on Hamming distances between haplotypes. The shaded rectangles indicate separate Atlantic haplotypes introgressed into Balsfjord herring. *B)* Schematic view of the introgressed haplotypes, and the resulting introgression coverage, with haplotypes colored according to the groups in *a*. The location of the *GRM4* gene is indicated.

HSRs that are fixed or close to fixation—by which we mean the 8-fold recurring HSRs—and thus presumably contain adaptive introgressed material, could reflect introgression of a single or multiple haplotypes, which largely corresponds to hard (single) or soft (multiple) selective sweeps (Hermisson and Pennings 2005; Pennings and Hermisson 2006a, 2006b). For introgression of a single haplotype or strong selection for a specific haplotype, the recurrent introgressed haplotypes will cluster closely in a distance tree; a typical signal of a hard selective sweep. In general, we find this not to be the case, but overlapping Atlantic HSR haplotypes in Balsfjord individuals are instead dispersed among the cluster of other Atlantic haplotypes in a distance tree (supplementary fig. S11, Supplementary Material online). This indicates that multiple introgressed haplotypes per locus are the rule at a given locus with overlapping HSRs, and suggests that introgression from Atlantic herring represents largely a random sample of Atlantic haplotypes and that any adaptive benefit is due to genetic differences between Atlantic and Pacific herring. In essence, while the situation does not involve selection on standing variation as such, the Atlantic gene pool serves as a source of many haplotypes sharing the same adaptive variant(s) on different backgrounds, resulting in a soft-sweep profile. However, we also do find some examples where most of the introgressed haplotypes are of a common origin, in varying states of decay. For example, five of the eight Atlantic haplotypes in the region around *GRM4* on chromosome 4 are identical-by-descent (fig. 5*A*).

### Introgression From Atlantic Herring is not Dominated by Recent Events

**Fig. 6.**
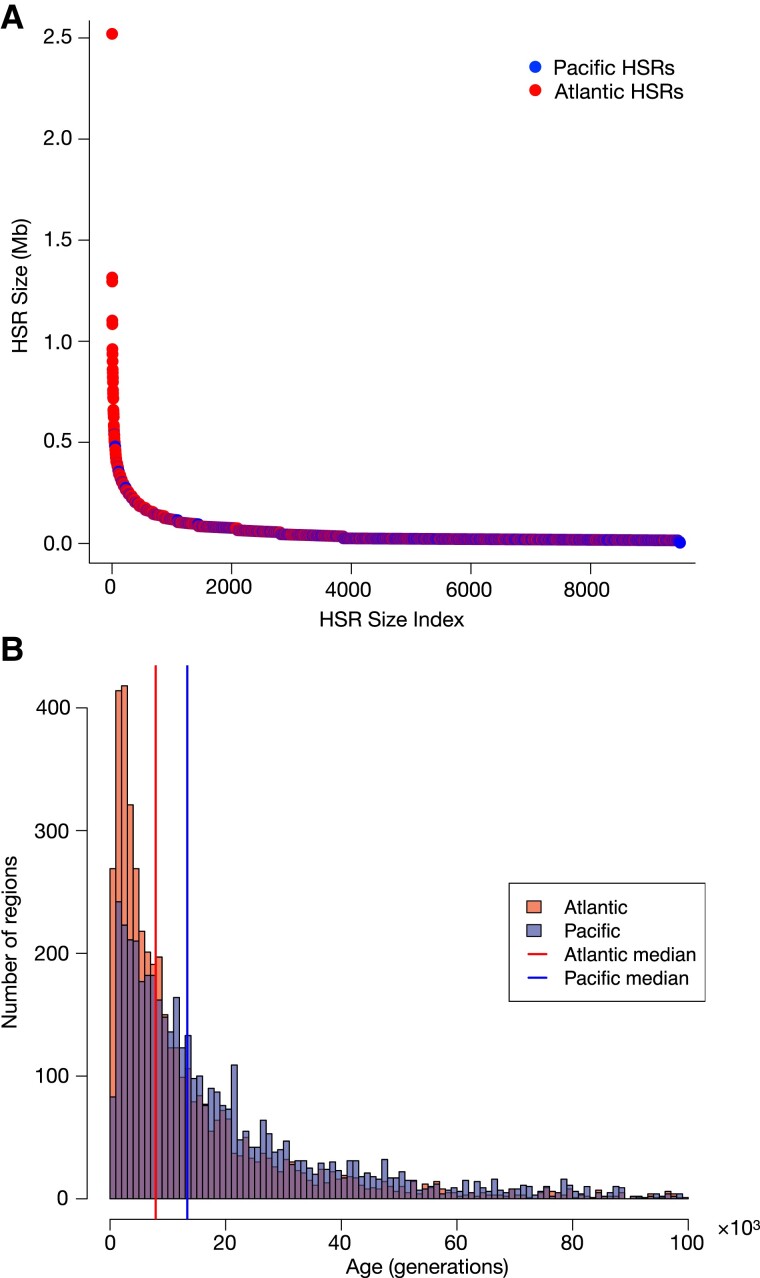
Size ranking and estimated age distribution of Atlantic and Pacific HSRs in Balsfjord herring. *A)* The detected HSRs have been ordered with respect to size, from largest to smallest. Color coding, by origin, demonstrates the on-average larger size of Atlantic HSRs *B)* Distribution of the estimated time since introgression, up to 10^5^ generations ago. There are 248 Pacific and 136 Atlantic regions with age estimates outside of this range. Medians (indicated by vertical lines) are calculated from all loci, including the outliers with estimated ages above 10^5^ generations.

Introgressed haplotypes will shrink over time due to recombination and we leveraged this property to estimate the age of the introgression events. Atlantic HSRs are over-represented among the larger regions, compared to the Pacific counterparts (*P*_wilcox_ = 6.8*10^−72^; fig. 6*A*). However, recombination is not even across the genome, and will variably influence haplotype size. We estimated the age of each HSR using the previously established herring recombination map (Pettersson et al. 2019) (*n*_Atl_ = 4,572, *n*_Pac_ = 4,578) and a simulation procedure adapted from Hill et al. (2019) that incorporates recombination rate. The idea is, essentially, that we apply the estimated recombination map to simulated introgressed chromosomes and record the time when the remaining fragment becomes smaller than the observed HSRs in question. We estimated that the Atlantic HSRs are considerably younger (median age = 7.9*10^3^ generations) than their Pacific counterparts (median age = 13.4*10^3^ generations) (fig. 6*B*). These estimates correspond to a median age of introgression of roughly 19 and 32 kYA, respectively, based on a generation time of 2.4 years for Pacific herring (Kitada et al. 2017; Mikkelsen et al. 2018). The genomic regions where all Balsfjord haplotypes represent introgression have lower estimates (due to longer introgressed haplotypes) than the respective overall set, both for Atlantic (median age = 2.1*10^3^ generations) and Pacific HSRs (median age = 8.5*10^3^ generations).

Given the continuous exposure to recombination events, it is unlikely for long introgressed haplotypes to be found at high frequencies unless they contain variants subject to positive selection. This is similar to hitch-hiking of linked variants during a classical selective sweep (Maynard-Smith and Haigh 1974). Selection can either cause near fixation in a short time span, which will prevent disruption by recombination since most events will occur between similar haplotypes, or, alternatively, if there is more than one variant subject to selection in an HSR, non-recombinant haplotypes can be favored and thus persist.

### Identification of Putatively Adaptive Introgressed Regions

**Fig. 7.**
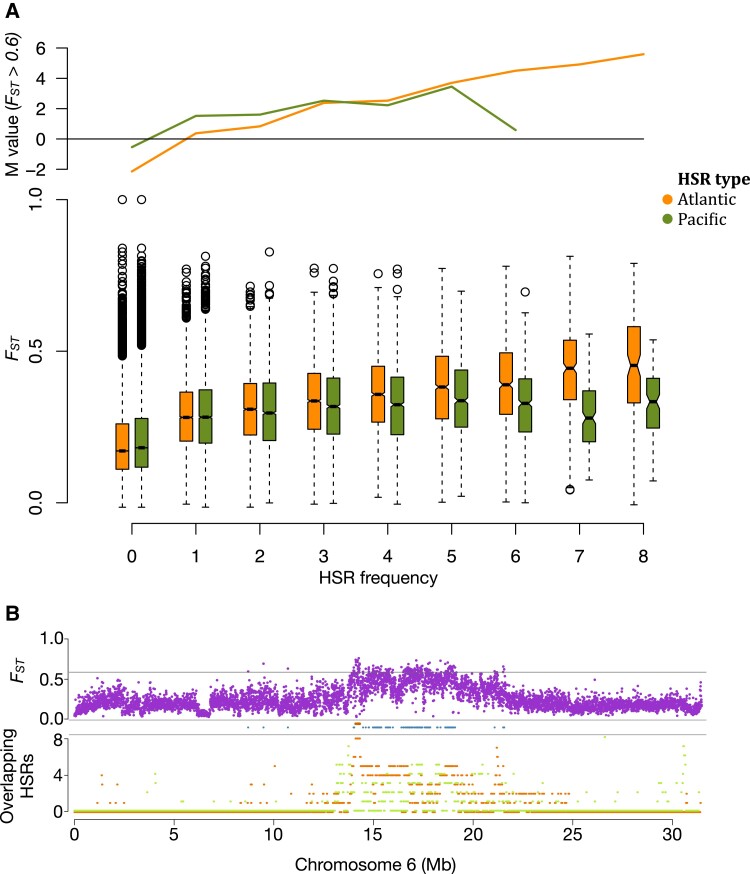
Correlation between HSRs in Balsfjord herring and estimated *F*_ST_ between Atlantic and Pacific herring. *A)* Boxplots showing the *F*_ST_ distribution for regions binned by the number of recurring HSRs. Atlantic HSRs are shown in orange, Pacific ones in green. *M*-values are based on comparing *F*_ST_ values within introgressed regions against the genomic average abundance of 5 kb blocks with *F*_ST_ > 0.6. *B)* HSR coverage (bottom track: Atlantic in orange, Pacific in green) and inter-species *F*_ST_ (top track: the grey line is the high *F*_ST_ threshold of 0.6) and their overlap (mid track: blue bars indicate high *F*_ST_ (>0.6) regions, orange bars indicate regions that have both high *F*_ST_ and eight Atlantic HSRs) shown across chromosome 6, as an example of their co-variation. See supplementary figure S12, Supplementary Material online for all chromosomes.

Regions of the genome where the two species show higher genetic differentiation than the genomic average are likely to have been affected by directional selection in either one or both species since their divergence (Lewontin and Krakauer 1973). In Balsfjord, we examined whether genomic regions in which Pacific and Atlantic herring are unusually divergent also show disproportionately high introgression frequency in the Balsfjord population. A population expanding into an environment (i.e., transitioning from NW Pacific to Atlantic) may be facilitated by the introgression of genetic material from a local population carrying locally adapted alleles. Assuming that beneficial variants confer the same advantage to the range-expanding population, introgressed loci may serve as a source of beneficial genetic material for the expanding population. In order to evaluate this possibility, we divided the genome into bins based on the number of haplotypes that carry either a Pacific or Atlantic HSR and asked if the genome with a high proportion of introgressed Atlantic haplotypes are skewed towards high *F*_ST_ regions. We found that introgressed regions tend to fall in regions of elevated *F*_ST_ (defined as average *F*_ST_> 0.6 across a 5 kb window, which corresponds to the top 0.5% of the genome) (fig. 7*A*; *P*_binomial_ < 3*10^−26^ for all groups with three or more overlapping Atlantic HSRs).

Additionally, 5 out of the 13 intervals (38%) where all eight Balsfjord haplotypes originate from Atlantic herring overlap these high-*F*_ST_ regions, even though only 3.3 Mb, 0.5% of the genome exceed the high-*F*_ST_ threshold. This co-occurrence is illustrated in figure 7*B* for a region on chromosome 6 with Atlantic HSRs and in supplementary figure S12, Supplementary Material online for all other regions showing high introgression levels. This result is consistent with the hypothesis that Balsfjord herring, which lives within the range of Atlantic herring, has acquired some Atlantic haplotypes that are beneficial in the local environment.

### Properties of Recurrently Introgressed Loci

Previous research in Atlantic herring identified that non-synonymous coding changes are the most over-represented type of mutations in regions strongly associated with ecological adaptation (Martinez-Barrio et al. 2016) and we predicted that regions associated with adaptive introgression are gene-rich, in comparison to the rest of the genome. We therefore examined the exon density in all regions where all eight Balsfjord haplotypes contained HSRs. The Atlantic HSRs were not gene rich, as 8.2% of these HSRs are covered by exons, compared to 8.9% for the entire annotated genome. The Pacific HSRs, on the other hand, are almost twice as exon-dense (15.7%, *P*_chisq_ = 2.1*10^−11^) as the genomic average.

If the recurrently introgressed regions were mainly due to drift, largely representing neutral parts of the genome, they are expected to have a within- species nucleotide diversity (*π*) similar to that of the rest of the genome. In order to evaluate this, we examined the diversity summary statistics *π* and *d_xy_* in the target regions. Atlantic herring nucleotide diversity is significantly lower in windows harboring recurring HSRs, both of Atlantic (*π̅* = 0.08%; *P*_*t*_test_ = 1.1*10^−19^) and Pacific (*π̅* = 0.10%; *P*_*t*_test_ = 4.8*10^−6^) origin, with sample groups as defined in figure 1, compared to 1,000 randomly selected 20 kb windows (*π̅* = 0.20%) (supplementary fig. S13, Supplementary Material online). In contrast, the NW Pacific and European Pacific herring samples have lower *π* only in Pacific HSR regions (*π̅* = 0.15%; *P_t__*_test_ = 8.6*10^−6^); the levels in regions corresponding to those where Atlantic introgression occurs in Balsfjord (*π̅* = 0.22%; *P*_*t*_test_ = 0.25) did not deviate significantly from the background level (*π̅* = 0.24%) (supplementary fig. S13, Supplementary Material online). *d*_*xy*_, between Atlantic and Pacific herring, was significantly lower in regions with Balsfjord Atlantic HSRs (*d*^®^_*xy*___atl_ = 0.35%) than in the rest of the genome (*d*^®^_*xy*___bg_ = 0.41%; *P*_*t*_test_ = 0.002) and the opposite trend was noted for the Pacific HSRs, but it did not reach statistical significance (*d*^®^_*xy*___pac_ = 0.47%; *d*^®^_*xy*___bg_ = 0.41%; *P*_*t*_test_ = 0.09) (supplementary fig. S13, Supplementary Material online). However, in both cases the relative difference in means between the groups for *d*_*xy*_ (around 15% of the background mean) was much smaller than for *π* (roughly 55% of the background mean). Overall, HSRs of Atlantic origin behave in a way consistent with positive selection, while Pacific HSRs seem more likely to be due to long-term purifying selection.

### Introgression is not Overrepresented in High Recombination Regions

Several studies have indicated that successful introgression events are more likely to occur in regions of elevated recombination rate (Schumer et al. 2018; Edelman et al. 2019). We evaluated this pattern in herring by comparing the recombination rates in the 8-fold recurring HSRs to the genomic background, using the previously calculated recombination profile for Atlantic herring (Pettersson et al. 2019). In the Balsfjord herring, recurring HSRs are predominantly found in regions with lower than average recombination rates (*ρ̅*_bg_ = 2.10 cM/Mb; *ρ̅*_atlhsr_ = 0.61 cM/Mb, *P*_*t*_test_ = 5.1*10^−10^; *ρ̅*_pachsr_ = 0.71 cM/Mb, *P*_*t*_test_ = 2.6*10^−5^) (supplementary fig. S14, Supplementary Material online), suggesting that a positive correlation between recombination rate and high introgression rates is not a general phenomenon across taxa.

## Discussion

The Atlantic and Pacific herring add to a long list of closely related species pairs where interspecies hybridization occurs occasionally (Abbott et al. 2013; Taylor and Larson 2019). We previously estimated the time since divergence of these sister species to about 2 million years before present based on mtDNA sequence differences (Martinez-Barrio et al. 2016). While the Balsfjord population is of Pacific herring origin, we observed that each of the four individually sequenced Balsfjord herring had a proportion of Atlantic ancestry in the range 25–26%, based on the Admixture analysis (fig. 2*B*). These very similar values suggest that the introgression pattern is not dominated by recent events, which is supported by the age estimates of individual introgressed segments. A larger sample of individuals sequenced to low depth, (<1X, *n* = 41), corroborated the observations made from the high coverage data, and also showed a small number of individuals with higher Atlantic contributions, indicating more recent hybridization (supplementary fig. S4, Supplementary Material online). Based on the length of haplotype blocks and on previously obtained recombination rates, we estimated the average time since introgression from Atlantic herring to the Balsfjord population to be about 20,000 years before present. Given that the current presence of Pacific herring in Europe is thought to be the result of a post-glacial invasion from the Pacific through the Bering strait, that is younger than 12,000 years (Laakkonen et al. 2013), it is, however, possible that the timing of introgression using HSR size resulted in an overestimation and that mixing instead started when the two species came into contact following re-establishment of the inter-oceanic connection. The overestimation could result from an underestimation of the true HSR size, which in fact is indicated by the *Twisst* analysis showing that some of the 8-fold recurring Atlantic HSRs do not cover the entire region with full support of the topology linking Balsfjord samples with the Atlantic herring. Even with these caveats, our results indicate that much, but not necessarily all, of the introgressed material present today is the result of events that occurred many thousand years ago.

The maintenance of this hybrid population over such long period of time implies that the Balsfjord herring is a distinct subpopulation. This stable hybrid population provides a rare example of the substrate needed to initiate a process leading to homoploid speciation, similar to the emergence of the Big Bird lineage of Darwin's finches by hybridization between *Geospiza fortis* and *G. conirostris* on Daphne Major (Lamichhaney et al. 2018). However, the major difference between the two systems is that the Big Bird lineage is a recent event and the population has been documented over six generations. On the other hand, the Balsfjord hybrid population has persisted, in all likelihood, for thousands of generations, and has established a unique genetic profile by replacing parts of its ancestral, Pacific-derived, genome with introgressed material of Atlantic origin.

The NSSH has a breeding population exceeding 100 billion individuals (Feng et al. 2017) and is present in Balsfjord and its near vicinity. This raises the question, why has the huge number of Atlantic herring present in its surroundings not simply absorbed the Balsfjord population? One scenario consistent with our results is that hybridization between Atlantic and Pacific herring occurred under extreme circumstances, the population size of at least the Atlantic herring was severely reduced during glaciations (Martinez-Barrio et al. 2016), and that introgression has been very limited subsequently, but it has not ceased completely. However, the Balsfjord hybrid population remains sufficiently isolated to maintain its distinctness from the surrounding Atlantic herring population, likely because it maintains the distinct littoral spawning behavior of Pacific herring, contrasted with the sub-littoral breeding of the Atlantic herring (Alderdice and Hourston 1985). Furthermore, although Atlantic herring inhabit Balsfjord, it is not a major spawning ground; the great majority of Atlantic herring spawn further south on the Norwegian coast.

It was previously reported that introgression between two sister species of swordfishes (*Xiphophorus*) was significantly overrepresented in regions of high recombination (Schumer et al. 2018). This pattern has also been seen in other systems (Sankararaman et al. 2014; Edelman et al. 2019), and the suggested explanation was that it is due to recombination breaking up linkage to alleles that are deleterious in hybrids. However, in the Balsfjord population, we found the opposite—introgression was overrepresented in genomic regions with low recombination. This overrepresentation may reflect a slight statistical bias because it is easier to detect larger haplotype blocks that have not been disrupted by recombination. Nevertheless, we found no indication of an excess of introgression in high recombination regions. Furthermore, there was no indication of a mito-nuclear incompatibility as introgression of mtDNA and nuclear sequences have occurred at a similar frequency. We therefore conclude that genetic incompatibility between Atlantic and Pacific herring is of minor importance, if it exists at all. The lack of genetic incompatibility is notable, because the nucleotide sequence divergence between Atlantic and Pacific herring is slightly larger than the one between the two swordtail sister species (∼0.45%) that show extensive genetic incompatibility (Schumer et al. 2018). The most striking difference between these two pairs of sister species is the drastically larger effective population sizes in herring, presenting the possibility that genetic incompatibilities may be less likely to occur in wide-ranging species with few geographic barriers, low genetic drift and efficient purifying selection.

We identified several non-random patterns when examining regions with high recurrence (frequency) of introgression among the eight haploid genomes studied. These can, broadly speaking, be explained by either purging of deleterious introgressed material in critical regions of the genome or by positive selection on adaptive loci. For the introgressed Atlantic regions, the allele frequency distribution, size distribution and positive correlation with inter-species *F*_ST_ are consistent with adaptive introgression. Additionally, some of these regions are also found to be introgressed in high frequencies in other contact zone populations in the White Sea (supplementary fig. S9, Supplementary Material online). However, the, albeit slight, reduced exon density of the introgressed regions overall is more consistent with tolerance of introgression in regions with little functional genic information. This suggests that the observed pattern stems from a combination of neutral and adaptive introgression events. Conversely, a high exon density in regions of distinctly Pacific character fits with the idea that these HSRs are rather slow-evolving, conserved regions with high similarity across all Pacific herring. However, if the main explanation for the non-random distribution of Atlantic introgression in the Balsfjord population is purging of deleterious material, the observed pattern would require a large fraction of the genome to be subject to such purifying counter-selection. This is at odds with the observation that adaptation to salinity and spawning time in Atlantic herring is mostly restricted to discrete genomic regions of small to modest size (Pettersson et al. 2019; Han et al. 2020) that, in total, cover a small part of the genome. In addition, in the absence of obvious genetic incompatibility between Atlantic and Pacific herring, it is difficult to identify a plausible mechanism for purifying selection driving the differential introgression of some regions over others in Balsfjord.

Populations colonizing new territory, with accompanying environmental differences, may receive locally beneficial alleles through introgression from nearby populations. In Balsfjord, we found that the retention of introgressed material from the Atlantic herring is non-random, in terms of genomic distribution, and likely contributes to adaptation by facilitating colonization of a new environment by co-opting alleles that are well suited to the local conditions.

In conclusion, the Balsfjord herring constitutes a stable hybrid population that has been maintained in its subarctic environment for thousands of generations, and our results suggest that this has been facilitated by the introgression of alleles from the Atlantic herring.

## Materials and Methods

### Sample Collection and Genome Resequencing

The study is based on sequence data generated in this study, as well as those reported in our previous studies (Martinez-Barrio et al. 2016; Lamichhaney et al. 2017; Han et al. 2020) (supplementary table S1, Supplementary Material online). Tissue samples from 35 to 57 Pacific herring per locality were obtained from the Pacific and from different European localities in Russia and Norway [fig. 1*a*; most of these samples were also used by Laakkonen et al. (Laakkonen et al. 2013, 2015)]. Genomic DNA was prepared using the DNeasy tissue kit (Qiagen). Pooled and individual whole genome sequencing were carried out as previously described (Martinez-Barrio et al. 2016). Illumina 2 × 150 bp short read sequences for the new samples in this study were analysed together with read sets that were generated previously.

### Alignment and Variant Calling

Paired-end reads of individuals sequenced to high and low depth were separately aligned to the Atlantic herring reference genome (Pettersson et al. 2019) using BWA v0.7.17 component BWA-MEM (Li 2013) and duplicates marked within the Sentieon wrapper (Freed et al. 2017). We called genomic variants (SNPs and indels) using the Haplotyper algorithm of the Sentieon wrapper which is based on the GATK 4 version of HaplotypeCaller (McKenna et al. 2010). Joint genotyping was performed using the Sentieon genotyper wrapper, which is a function that parallelizes GATK 4 calls of GenotypeGVCFs. During variant calling, all sites, including invariant, were retained. Following joint genotyping, stringent filtration of the raw SNP variants was done following the criteria “MQ < 40.0 || MQRankSum < -12.5 || ReadPosRankSum < -8.0 || QD < 2.0 || FS > 60.0” and removing genotypes of “DP < 2, DP > 100, GQ <20” using GATK VariantFiltration and SelectVariants functions. We retained high quality invariant sites in order to accurately calculate genetic diversity statistics. To explore haplotypes in the downstream studies, we performed statistical, reference free, phasing of the individual data using BEAGLE v4.0 (Browning and Browning 2007).

### Genotype Likelihood Estimation

As genotype calling is not reliable in low coverage data (<1–2X), we identified variant sites and estimated genotype likelihoods using the program ANGSD v0.933 (Korneliussen et al. 2014) using these parameters:

$ANGSD_PATH/angsd -bam $BAM_LIST -ref $REF_GENOME -nThreads $N_CPU -r $CHR_target
-doMaf 1 -GL 2 -doGlf 2 -doMajorMinor 1 -doCounts 1 -doGeno -4 -doPost 1 -postCutoff 0.8 -doPlink 2 \
-uniqueOnly 1 -remove_bads 1 -only_proper_pairs 1 -trim 0
-minMapQ 20 -minQ 20 -minInd $MIN_IND
-minMaf 0.05 -SNP_pval 1e-6 \
-out $OUTPUT_PREFFIX.$CHR_target

### Genetic Diversity Calculations

We calculated unbiased estimates of *F*_ST_, *d*_*xy*_, and *π* using the software pixy (v0.95.2) (Korunes and Samuk 2021) using the same set of individuals, grouped according to the labeling in figure 1, and our VCF file that included both invariant sites and SNPs. We calculated *F*_ST_, *d*_*xy*_, and nucleotide diversities based on individual Pacific and Atlantic herring samples in non-overlapping 5 kb windows and by applying a minor-allele frequency filter of 0.05 within the pixy command line.

### Population Genetic Structure

We examined the extent of genetic population subdivision using three approaches: an NJ tree, PCA, and admixture analysis. The NJ tree was based on estimates of allele frequencies of 60 pools, while the PCA and the admixture analyses were based on the genotypes of 79 individuals (supplementary table S1, Supplementary Material online). The NJ tree was generated based on summed allele frequency distances using the *bionj* method, with default parameters, from the R-package *ape* (v5.3) (Paradis and Schliep 2019) based on 10^5^ random SNPs.

Given that the PCA and admixture analysis assume markers are independent, we first thinned the individual SNP dataset by applying linkage disequilibrium (LD) pruning with plink v.1.9 (www.cog-genomics.org/plink/1.9/) (Chang et al. 2015), using a 50 kb window size and 10 bp window step size and an *r*^2^ threshold greater than 0.1. The PCA was performed using plink and plotting was done in R using the package ggplot (Wickham 2016). Individual ancestry coefficients were estimated based on the sparse nonnegative matrix factorization sNMF algorithm (Frichot et al. 2014) implemented in the R package LEA (http://membres-timc.imag.fr/Olivier.Francois/lea.html). We ran the program assuming 1 to 5 ancestral populations (*K*) using 10 repetitions and 200 iterations. The cross-entropy criterion was used to determine the most likely number of *K*. A map showing the admixture proportions for all the sampled sites was created in R using the packages ggplot and ggOceanMaps (https://mikkovihtakari.github.io/ggOceanMaps/).

Additionally, we performed an admixture analysis for all the individual samples, including high- and low-coverage sequenced individuals (*n* = 120), using the package NGSadmix (Skotte et al. 2013). This analysis was conducted on the set of LD-pruned SNPs identified in the high coverage data, which were supplied to the NGSadmix algorithm using the flag “-sites”. We ran the program for *K* = 1 to 5, with 10 replicates for each *K* value tested. To determine the most likely number of *K*, we used the Evanno method (Evanno et al. 2005) implemented in the program CLUMPAK (Kopelman et al. 2015). This method is based on the calculation of Delta *K* (Δ*K*), metric that estimates the rate of change in the log probability of data between successive *K* values. Thus, the most probable *K* for a given dataset corresponds to the highest value. Plotting was conducted using ggplot in R.

### Scoring Metric for HSR Detection

Our metric for detection of HSRs is based on the relative distance to the closest Atlantic and Pacific reference haplotype, respectively. This was achieved with the following procedure:

1. The genome was divided into 20 kb windows.
2. Reference haplotypes were extracted in each window from pre-defined sets of individuals that constitute the “Atlantic-” (the eight samples originally described in (Martinez-Barrio et al. 2016), labeled AM or AF) and “Pacific-” (Northwest Pacific samples from the Sea of Japan, labeled HWS1) comparison group.
3. Minimum Hamming distances were calculated from each sample haplotype, per window, to the corresponding haplotypes in each comparison group. Here, we eliminate windows where the largest of these distances is <20, to account for regions with very low total SNP diversity.
4. The ratio of these distances was taken to get the final introgression score. The use of a ratio provides an internal correction for the overall variability within each window and also generates a metric that is symmetric in log space, which is convenient for simultaneously detecting HSRs in both directions.
5. Based on the typical number of SNPs per window, we set thresholds for technically recognizing HSRs. These were at ratios 8 times higher than the mean ratio (1.45) for “introgressed” Pacific blocks (i.e., ∼12), and at lower than 1/8 of the mean ratio for Atlantic blocks (∼0.18). While the exact power will vary from window to window, depending on the number of SNPs, these thresholds have a <10^−10^ chance of being exceeded, by accidental sorting of SNPs, when the true distance ratio of a window is that of the genomic average. This is well in excess of Bonferroni correction for the number of windows across the genome (*n* = 38,553).
6. Finally, closely located introgressed regions less than 50 kb apart were combined into contiguous regions.

The scores were calculated using custom R scripts, adapted from the package HaploDistScan (Pettersson et al. 2017).

### *Twisst* Analysis

For this analysis, we used the SNP genotype set described above. The individuals included in the analysis were: the two parental species populations, Atlantic—Norway (*n* = 8) and Pacific—Sea of Japan (*n* = 4); the admixed population of Balsfjord (*n* = 4); and a more distantly related population from the Pacific—Vancouver (*n* = 6). Therefore, the *Twisst* analysis consisted in four taxa, resulting in three possible taxon topologies. We performed the analysis per chromosome. Thus, we first created a separate VCF file for each chromosome using vcftools (Danecek et al. 2011) and converted the resulting files into the “geno” format required by *Twisst* using the script “genomics_general/VCF_processing/parseVCF.py”. Subtrees along each chromosome were generated in windows of 100 SNPs (shorter window sizes, 25 and 50 SNPs, were tested but 100 SNPs offered the best resolution), requiring a minimum of 70 SNPs per individual in each window, and using the script “genomics_general/phylo/phyml_sliding_windows.py”, which invokes PhyML 3.0 (Guindon et al. 2010) The topology weights per window were calculated with the script “twisst/twisst.py”. Plotting of the topology support along the genome was performed using the package *ggplot2* (Wickham 2016) in the R framework (R Core Team 2019). All the referenced python scripts are publicly available in the github repositories https://github.com/simonhmartin/twisst and https://github.com/simonhmartin/genomics_general.

### Age Simulations

The simulations for age of the introgressed blocks were performed using custom R (R Core Team 2019) code, where up to one recombination event was placed on the chromosome each generation, with each base having a probability to be chosen proportional to the estimated recombination rate at its position. The recombination rates were calculated by dividing up the total genetic map distance for each chromosome proportionally to the fraction of recombination events assigned to each 100 kb window, using data from (Pettersson et al. 2019). The procedure was adapted from Hill et al. (2019).

### Exon Density Calculations

The exon density was calculated based on the annotation of the Atlantic herring genome included in ENSEMBL release 99. The value as obtained by dividing the size of all exons overlapping the target region for three sets of sequences: regions with recognized Atlantic HSRs, those with corresponding Pacific HSRs, or all chromosomes. This ignores the edge effects of exons spanning the edge of a region, but, given that herring exons are typically short (mean = 247 bases), this will cause only marginal overestimation of the exon density in HSRs.

### Data Availability Statement

The sequence data generated in this study is available from the NCBI Short Read Archive (SRA) in Bioproject PRJNA642736. Allele frequencies for each SNP in each population, as well as the individual genotypes used, are available at: DOI:10.17044/scilifelab.22361761.

### Code Availability Statement

The analyses of data have been carried out with publicly available software and all are cited in the Methods section. Custom scripts used are available in Github (https://github.com/LeifAnderssonLab/Arctic_Herring_Introgression).

## Supplementary Material

evad069_Supplementary_Data
