# Supplementary material for: A Long-Standing Hybrid Population Between Pacific and Atlantic Herring in a Subarctic Fjord of Norway
Source: Genome Biol Evol. 2023 Apr 30;15(5):evad069. doi: 10.1093/gbe/evad069 (PMC10182735; doi:10.1093/gbe/evad069)
Supplement: evad069_Supplementary_Data [file evad069_supplementary_data.zip › Supplementary_Figure_11.pdf]

Supplementary Figure 11: Hierarchical clusterings of haplotypes in 8-fold Atlantic HSRs
The dendrograms are based on Hamming distances between haplotypes in the indicated regions. One 20 kb window has been selected from each recurring HSR.

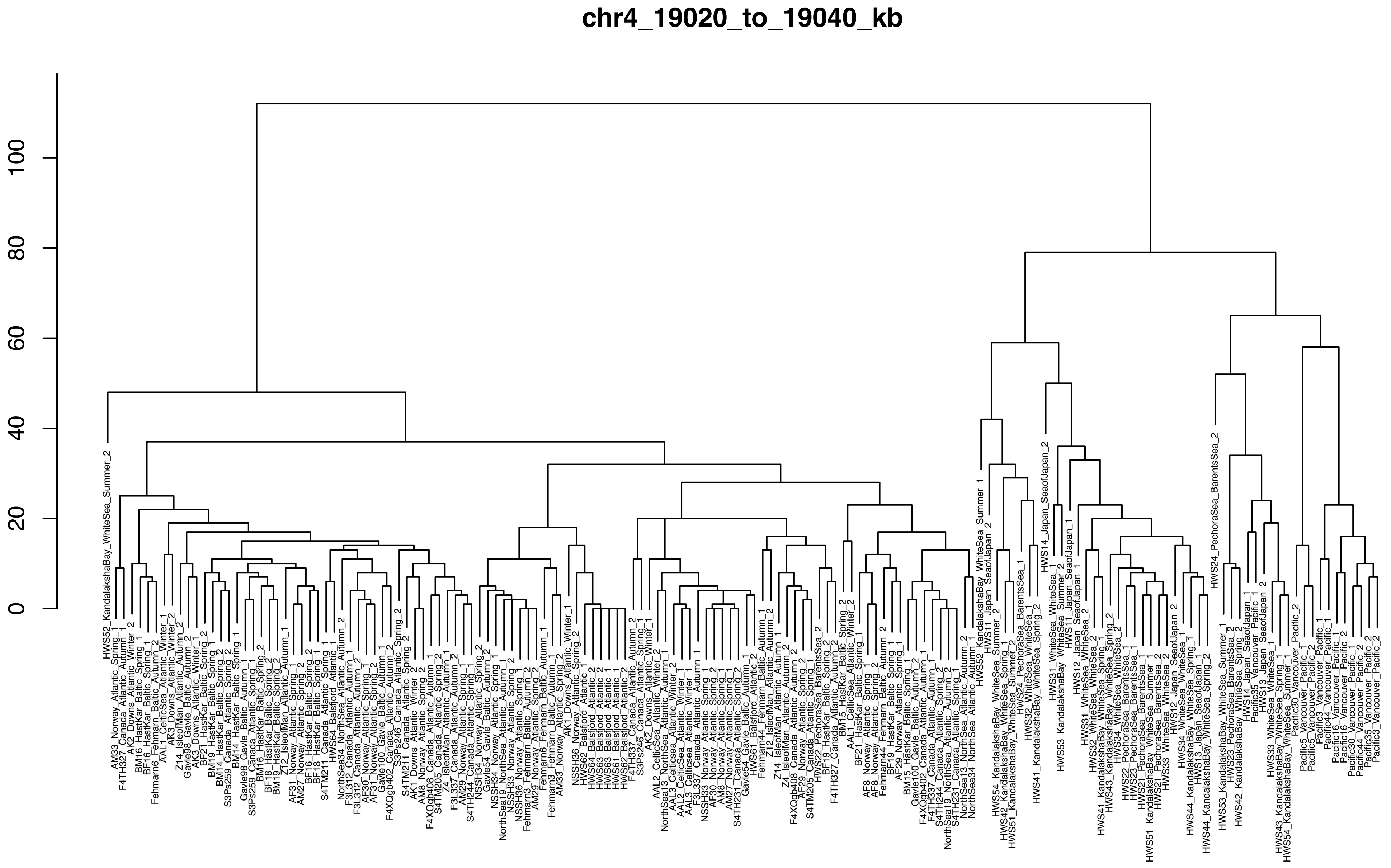
chr4_19020_to_19040_kb
0
20
40
60
80
100

chr4_30120_to_30140_kb

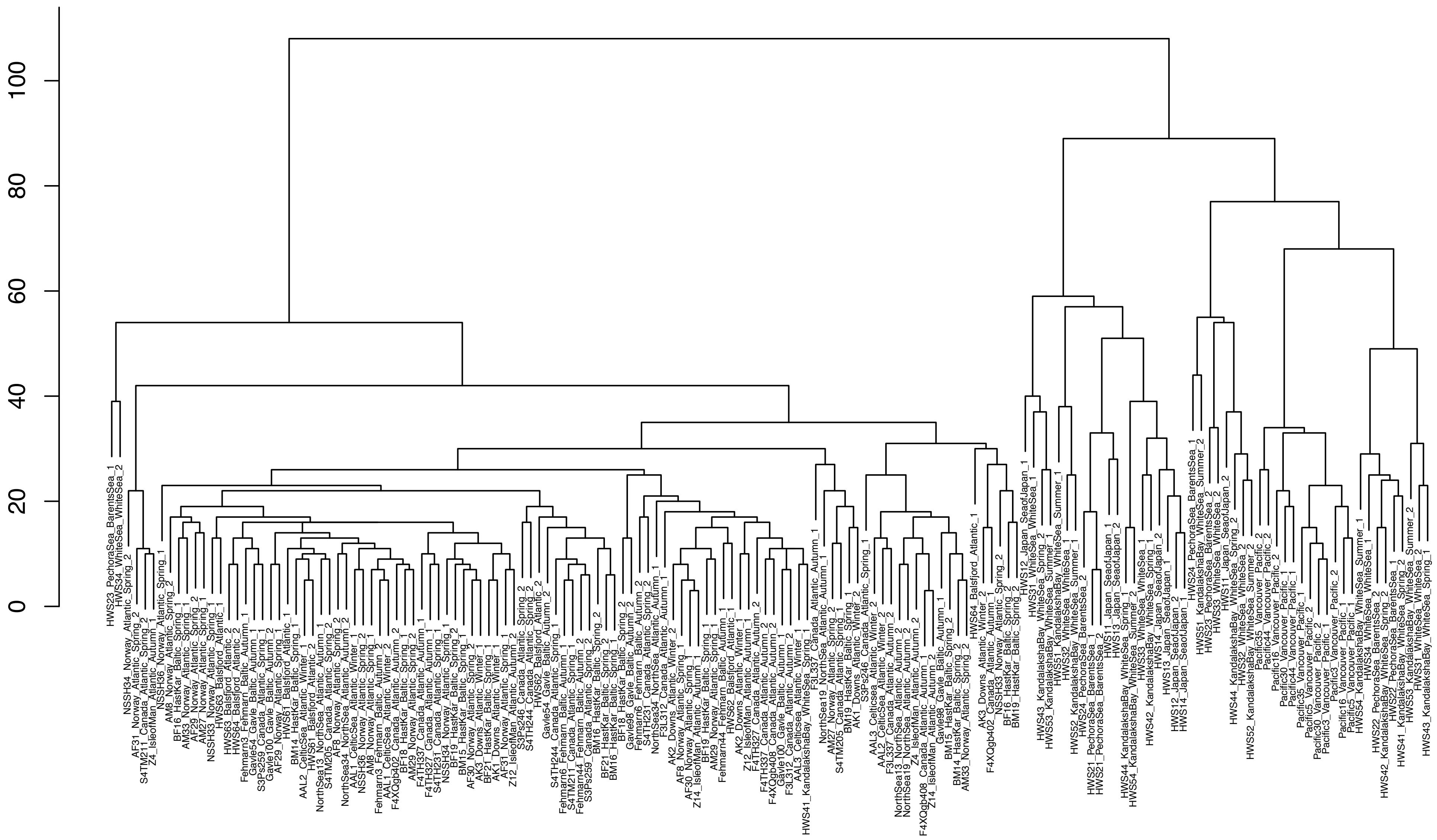

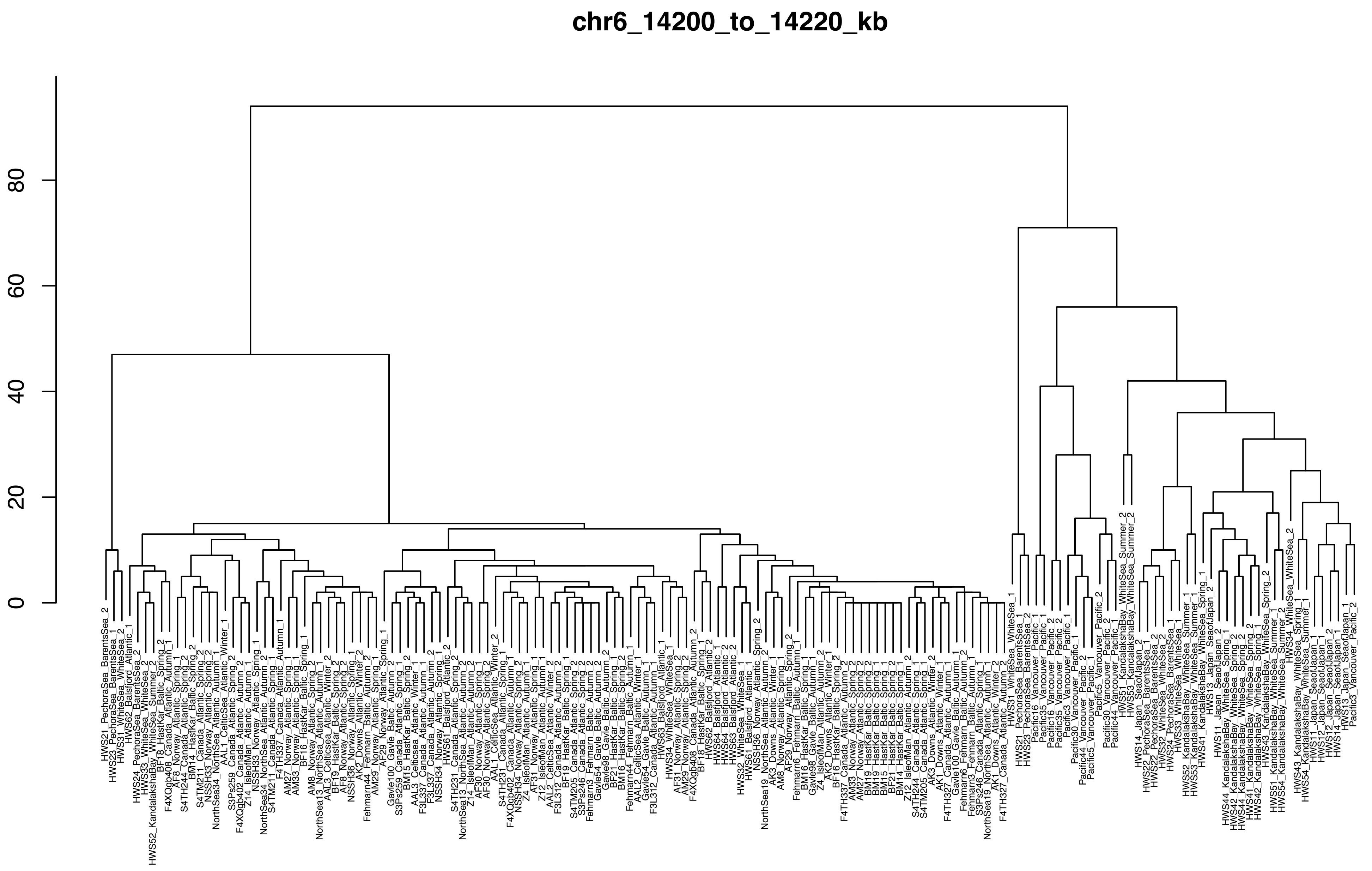
chr6_14200_to_14220_kb
0
20
40
60
80

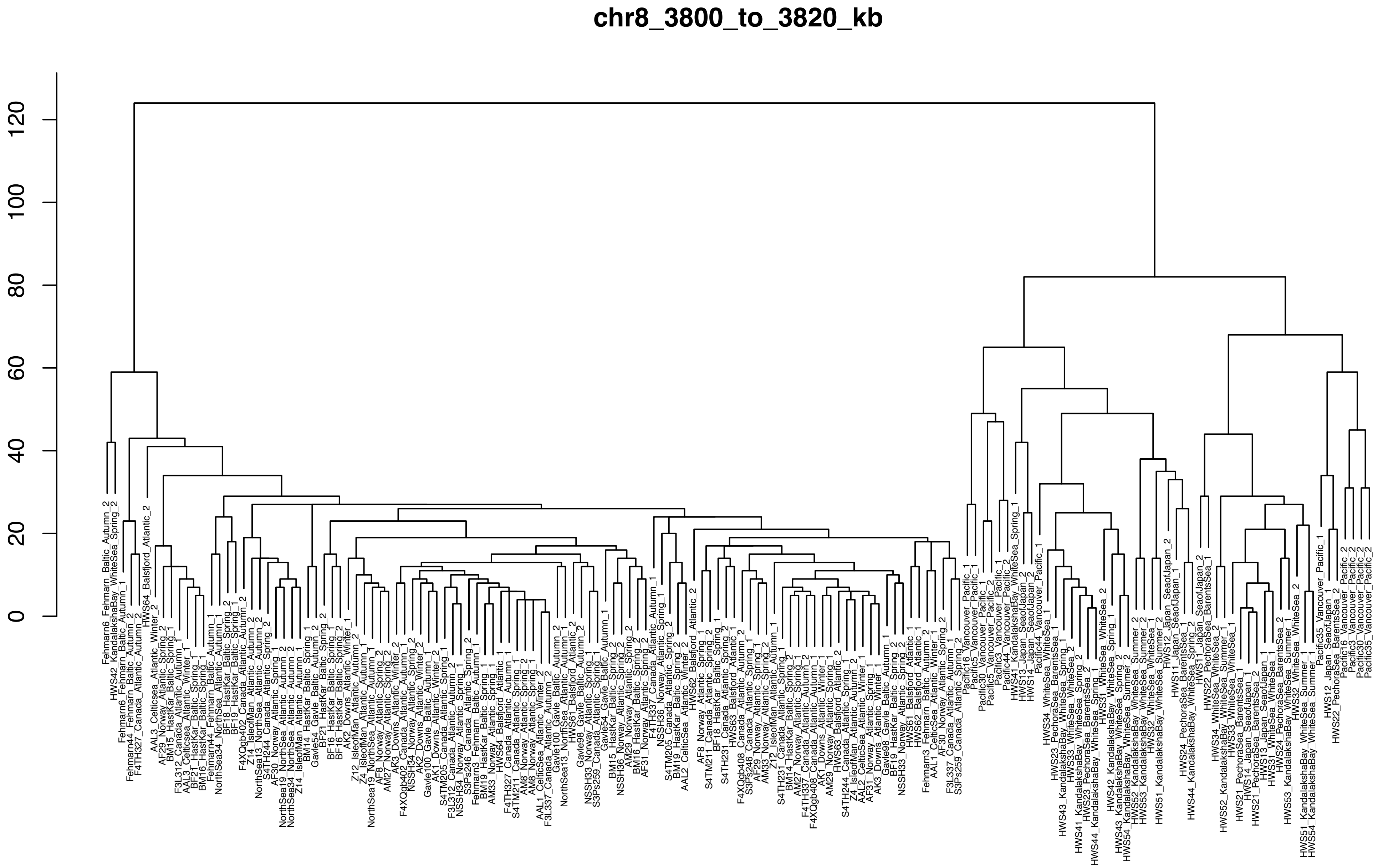
chr8_3800_to_3820_kb
0
20
40
60
80
100
120

chr8_6920_to_6940_kb

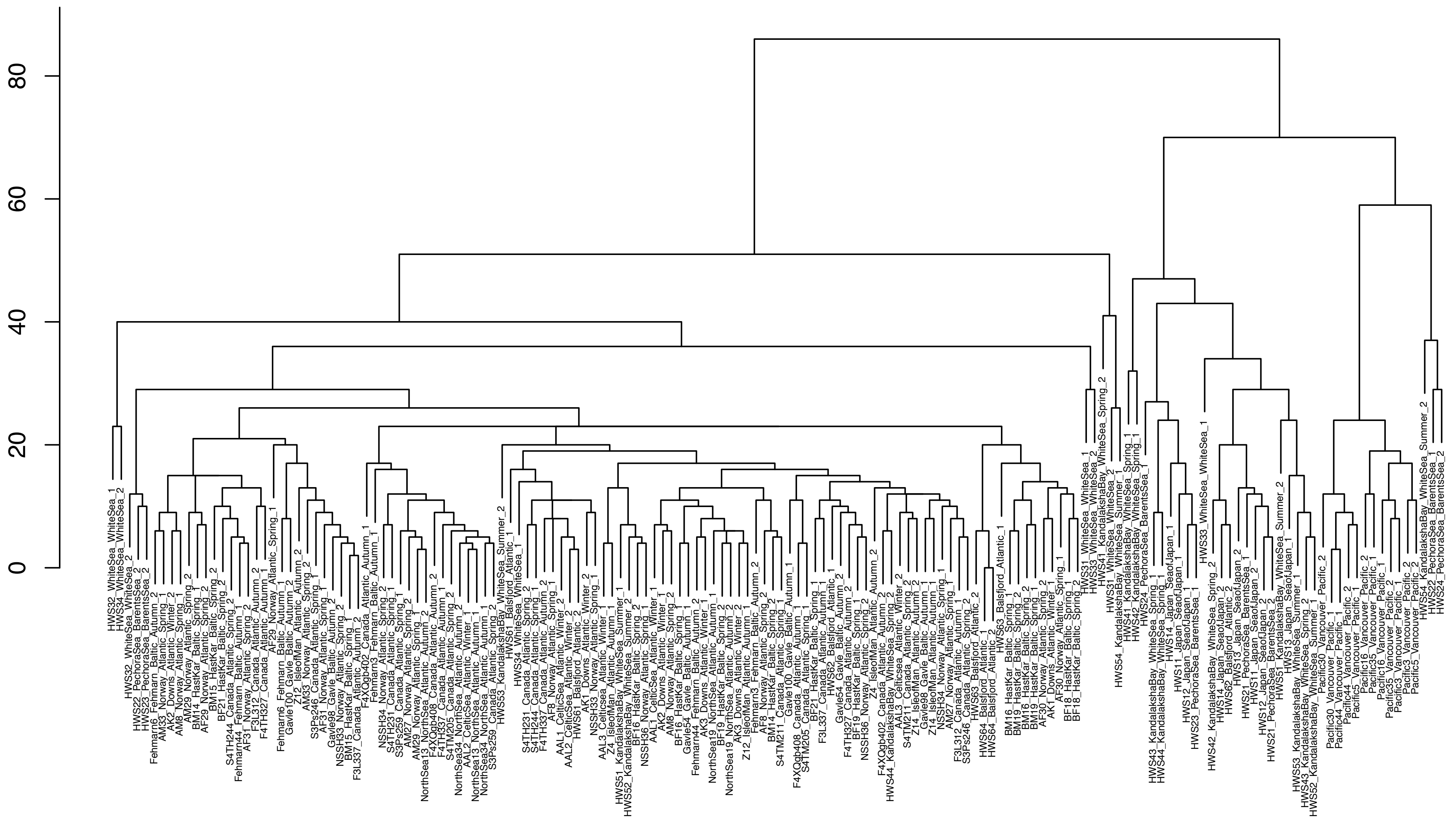

chr13_740_to_760_kb

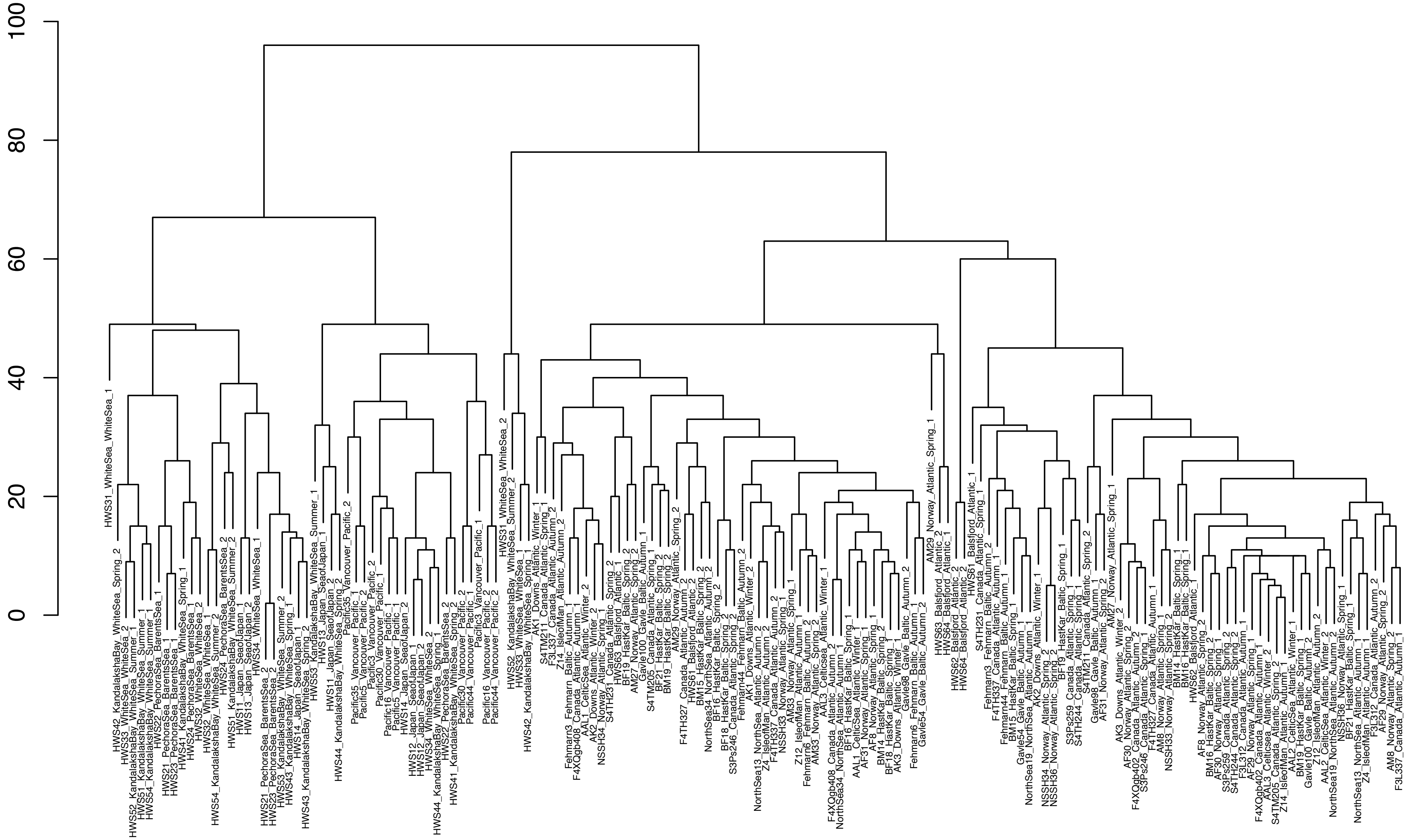

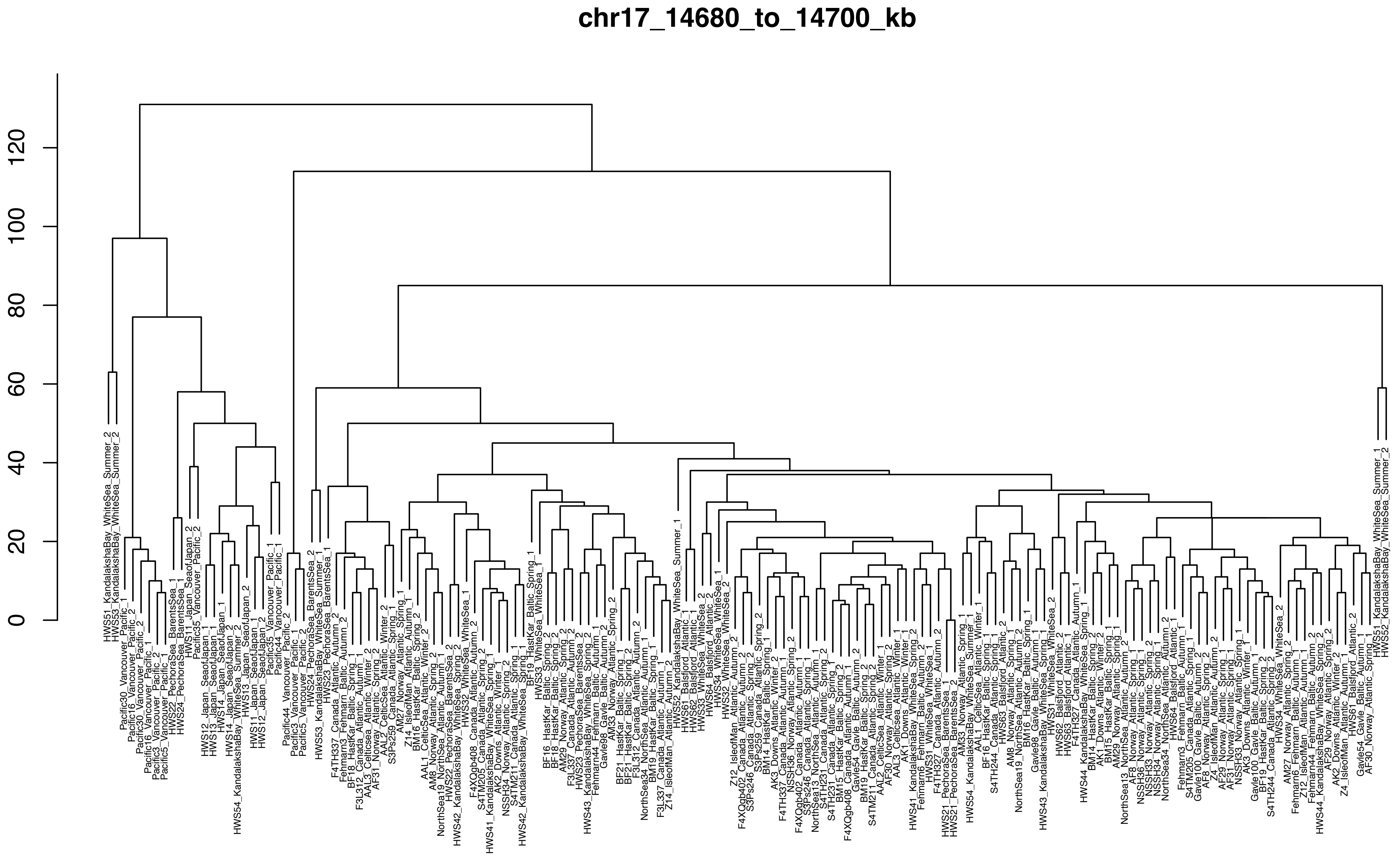
chr17_14680_to_14700_kb
0
20
40
60
80
100
120

chr18_12560_to_12580_kb

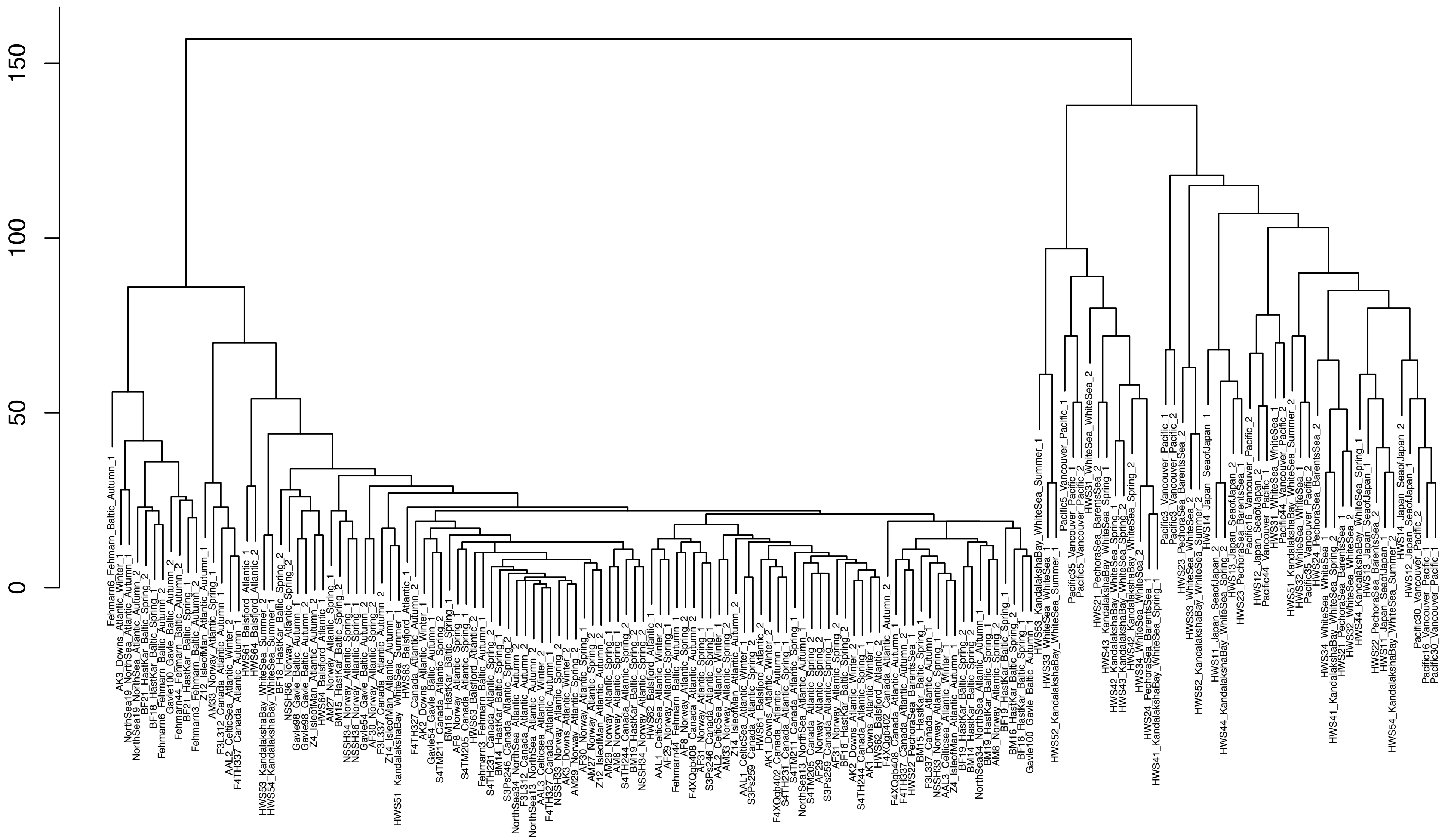

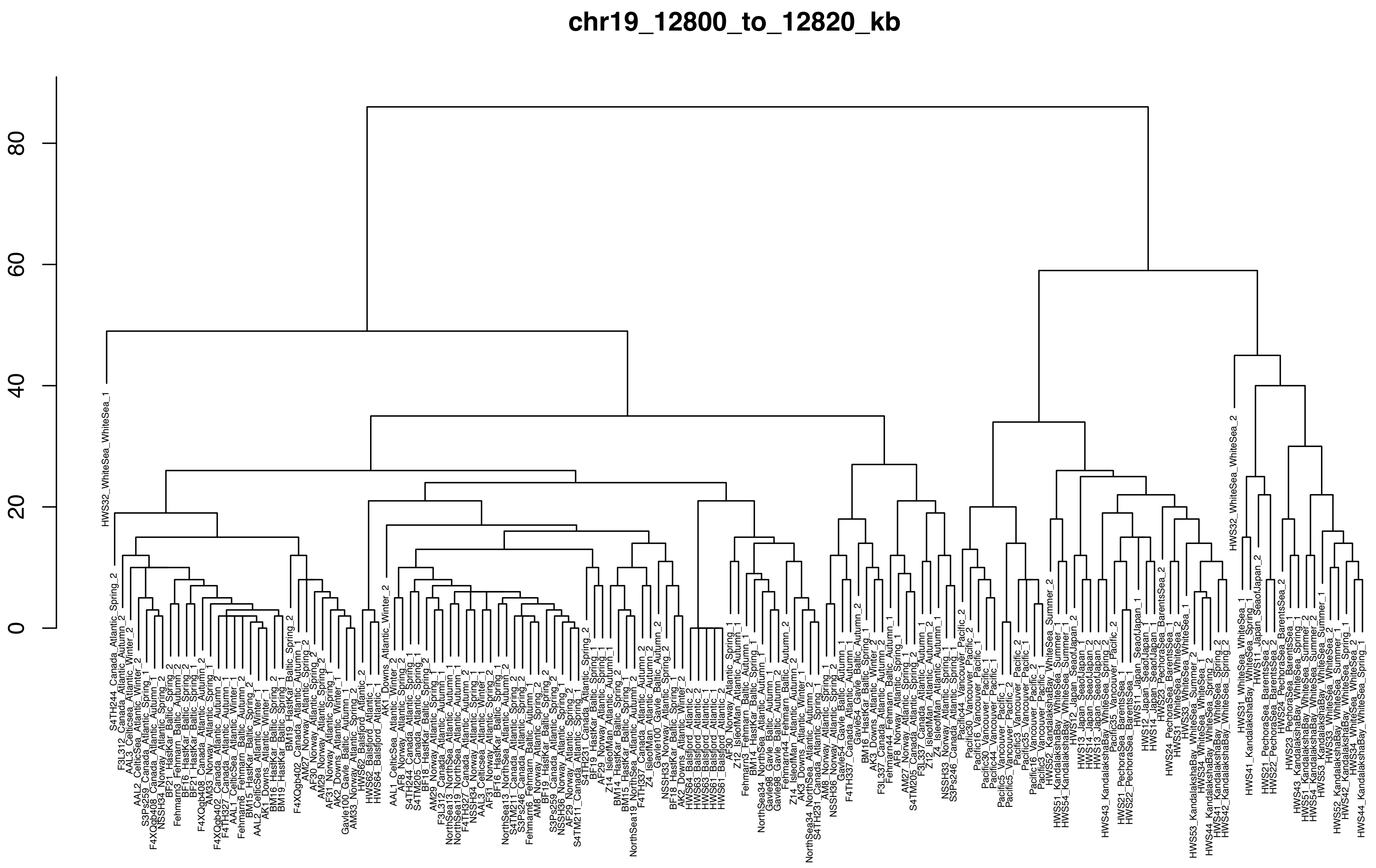
chr19_12800_to_12820_kb
0
20
40
60
80

chr21_4760_to_4780_kb

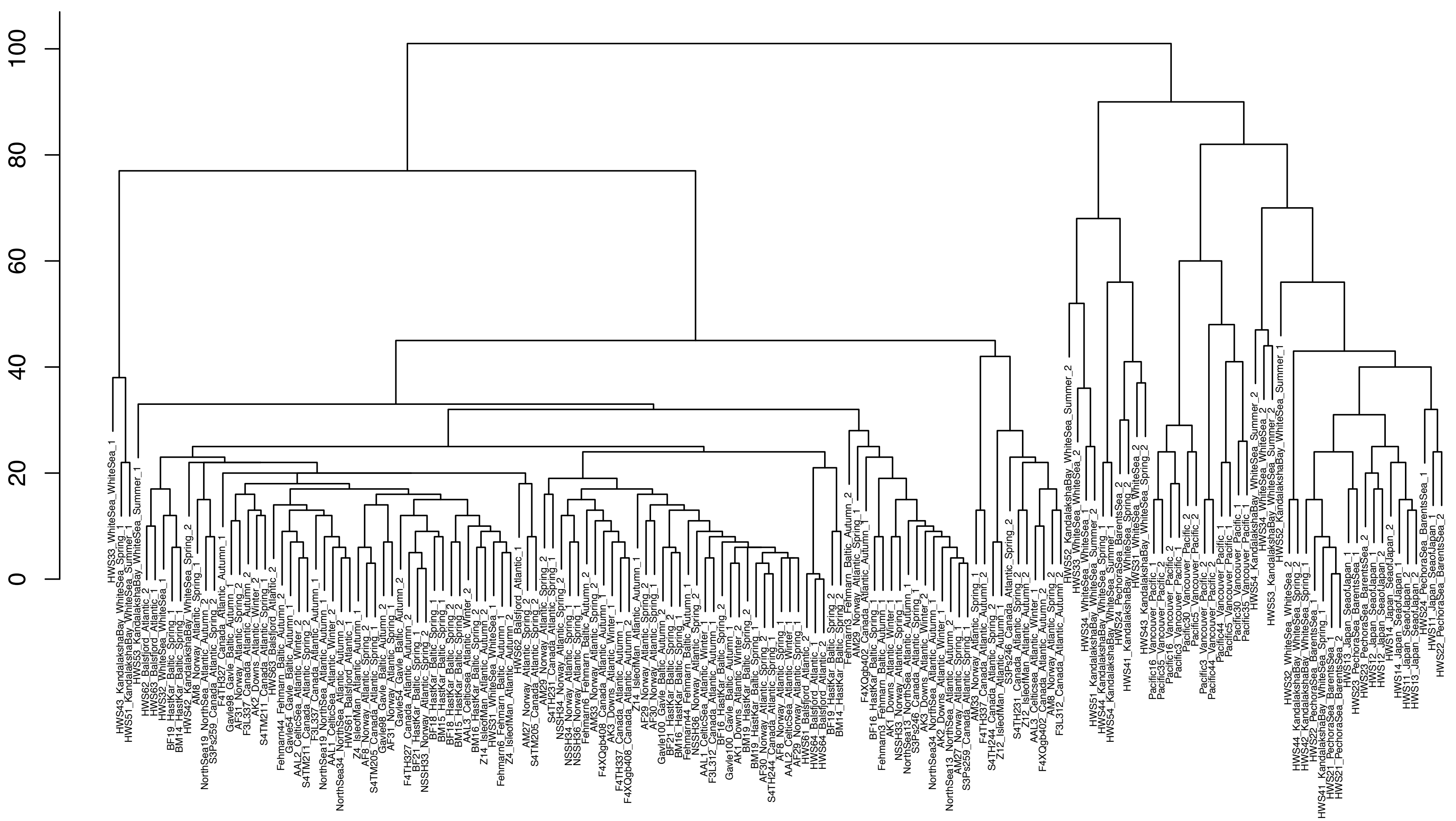

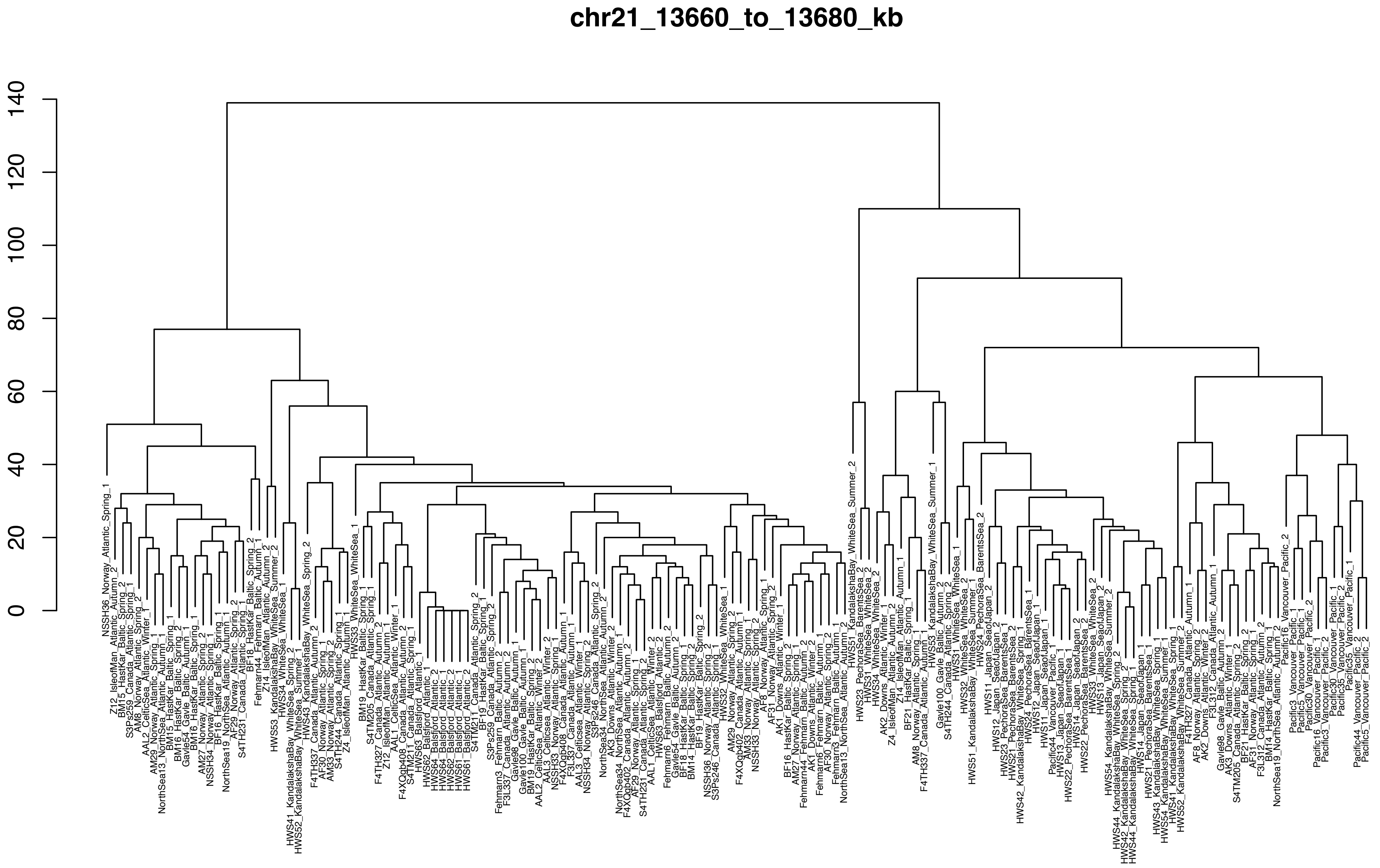
chr21_13660_to_13680_kb
0
20
40
60
80
100
120
140

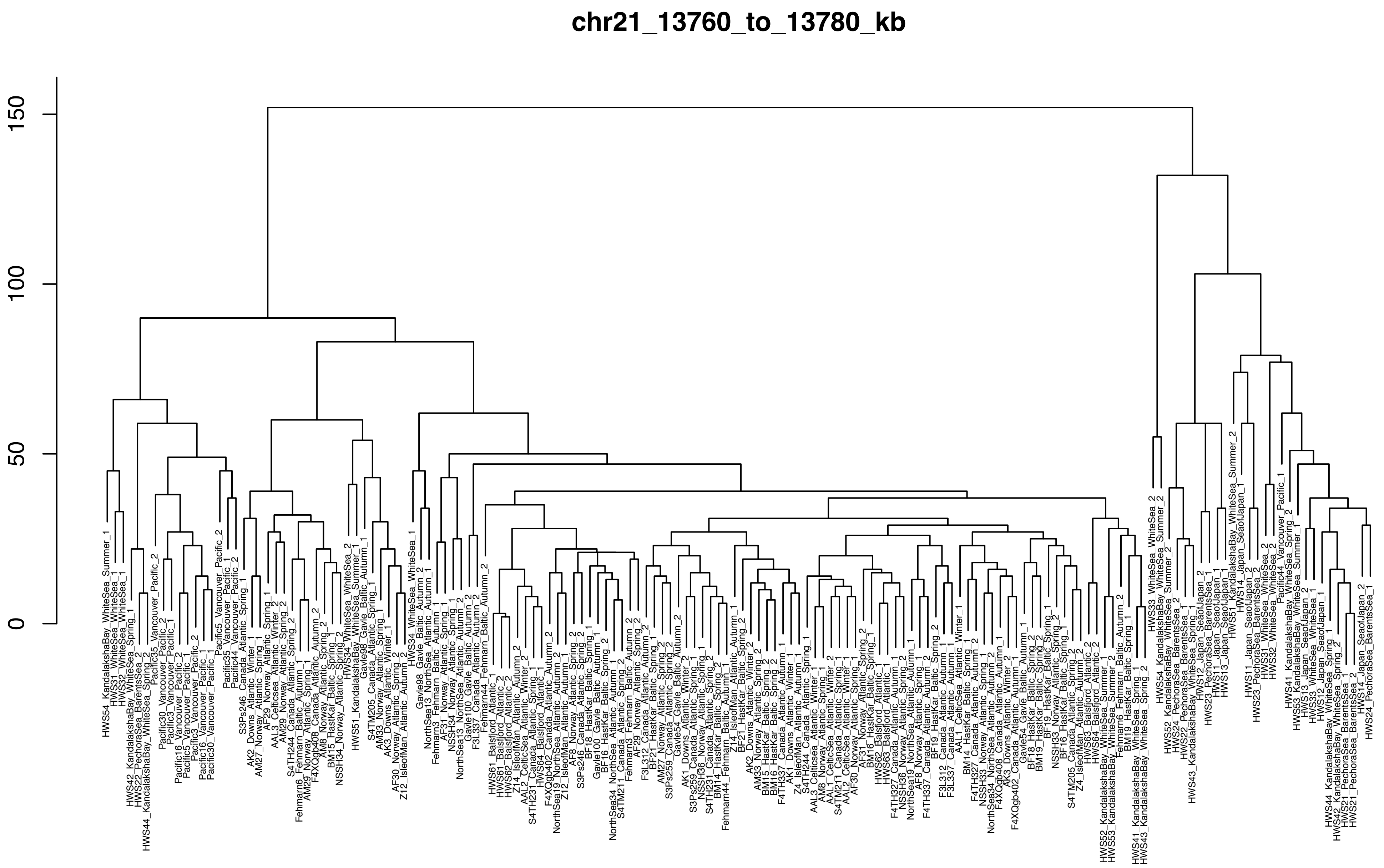
chr21_13760_to_13780_kb
0
50
100
150

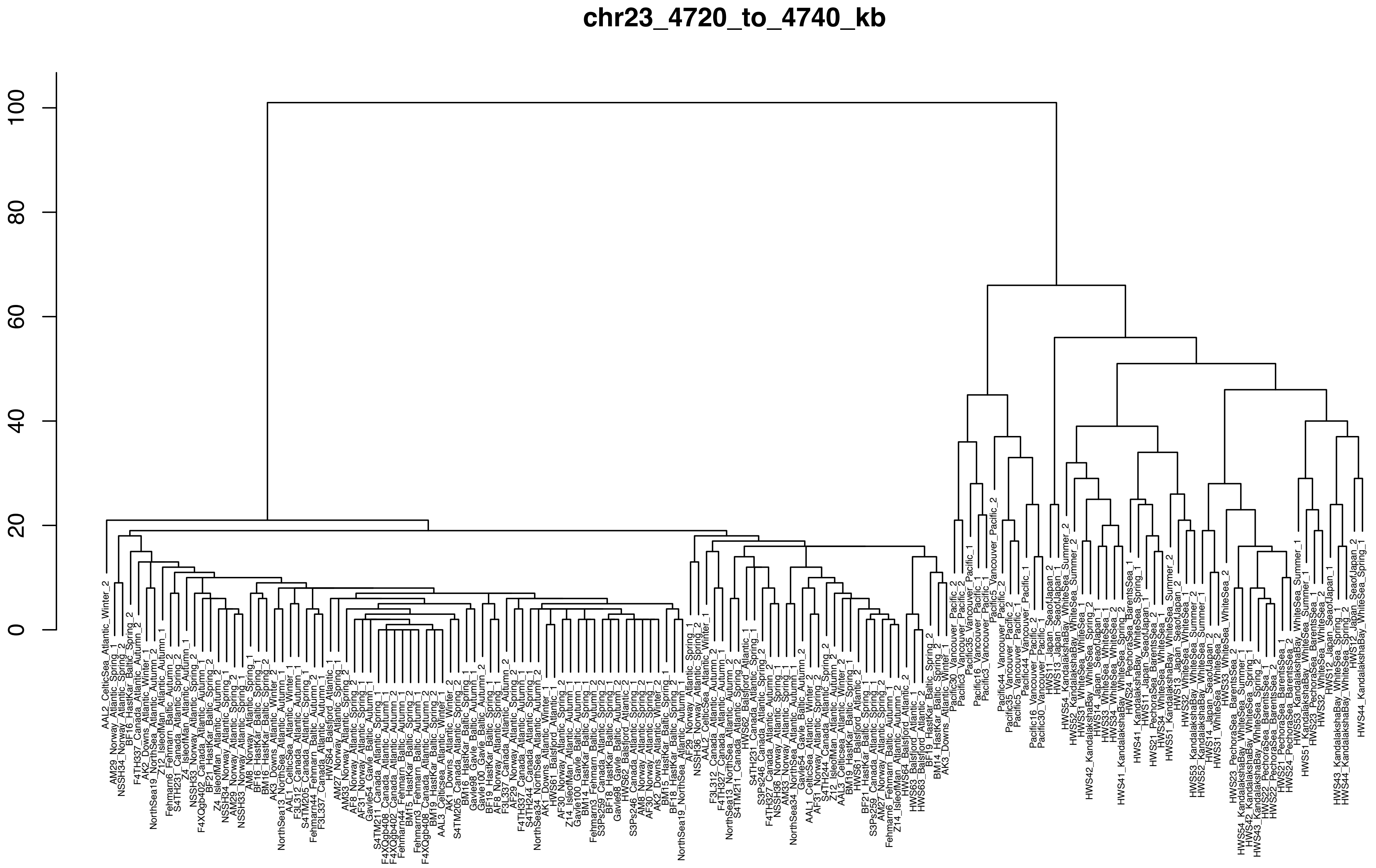
chr23_4720_to_4740_kb
100
80
60
40
20
0
