# Supplementary material for: A Long-Standing Hybrid Population Between Pacific and Atlantic Herring in a Subarctic Fjord of Norway
Source: Genome Biol Evol. 2023 Apr 30;15(5):evad069. doi: 10.1093/gbe/evad069 (PMC10182735; doi:10.1093/gbe/evad069)
Supplement: evad069_Supplementary_Data [file evad069_supplementary_data.zip › Supplementary_Figure_12.pdf]

Supplementary Figure 12. Inter-species FST and number of HSRs in Balsfjord samples (of Pacific and Atlantic origin) across all chromosomes.

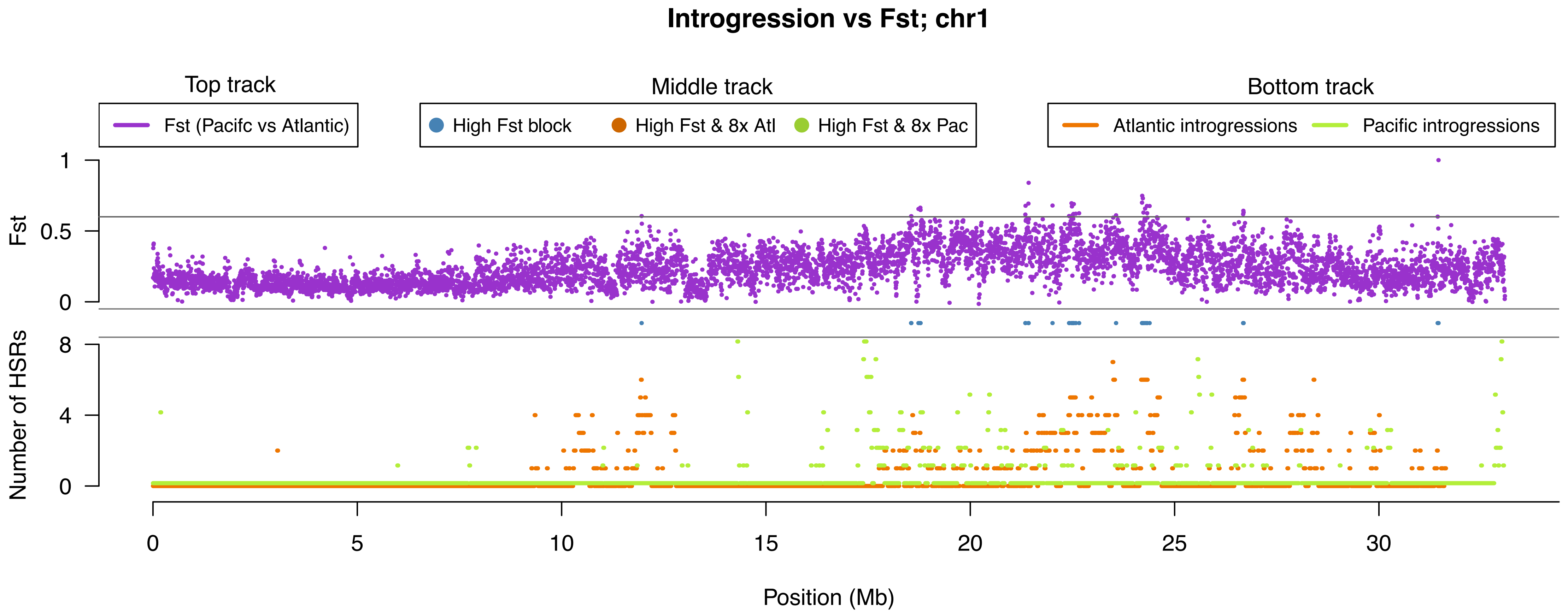

Introgression vs Fst; chr1
Top track
Fst (Pacifc vs Atlantic)
Middle track
High Fst block
High Fst & 8x Atl
High Fst & 8x Pac
Bottom track
Atlantic introgressions
Pacific introgressions
Fst
1
0.5
0
Number of HSRs
8
4
0
0
5
10
15
20
25
30
Position (Mb)

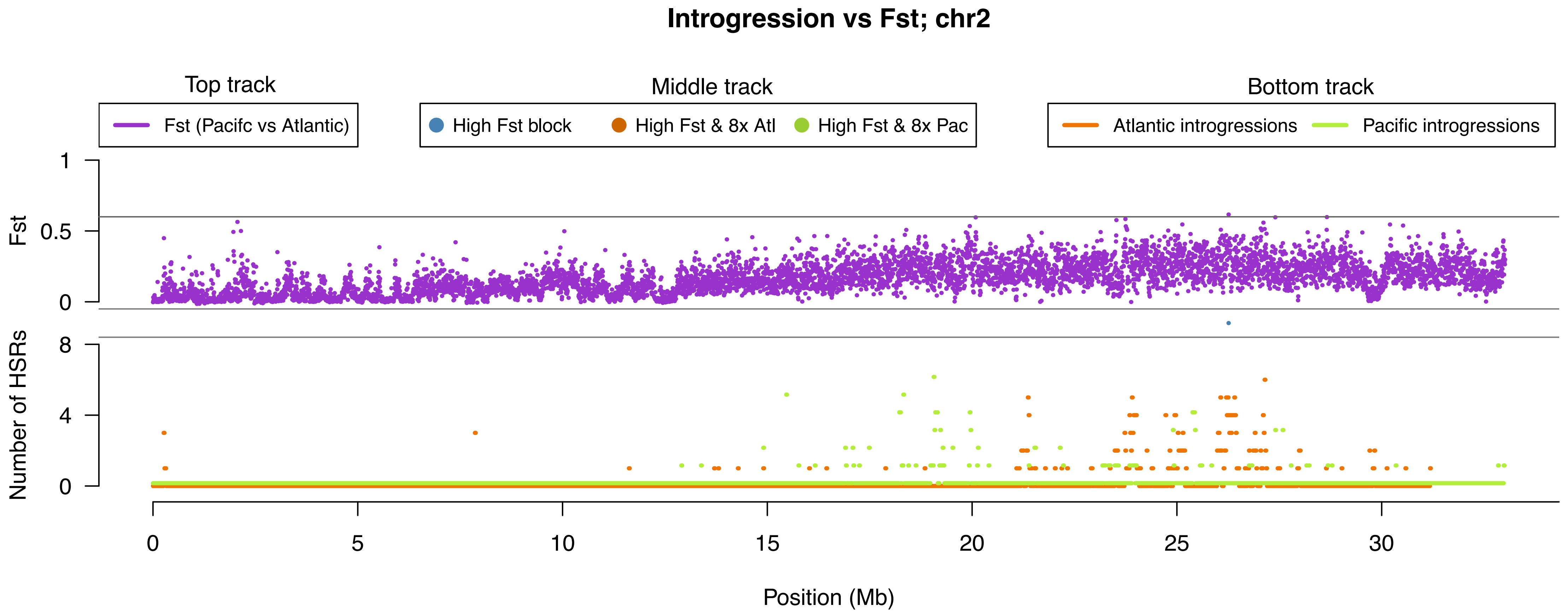

Introgression vs Fst; chr2
Top track
Fst (Pacifc vs Atlantic)
Middle track
High Fst block
High Fst & 8x Atl
High Fst & 8x Pac
Bottom track
Atlantic introgressions
Pacific introgressions
Fst
1
0.5
0
Number of HSRs
8
4
0
0
5
10
15
20
25
30
Position (Mb)

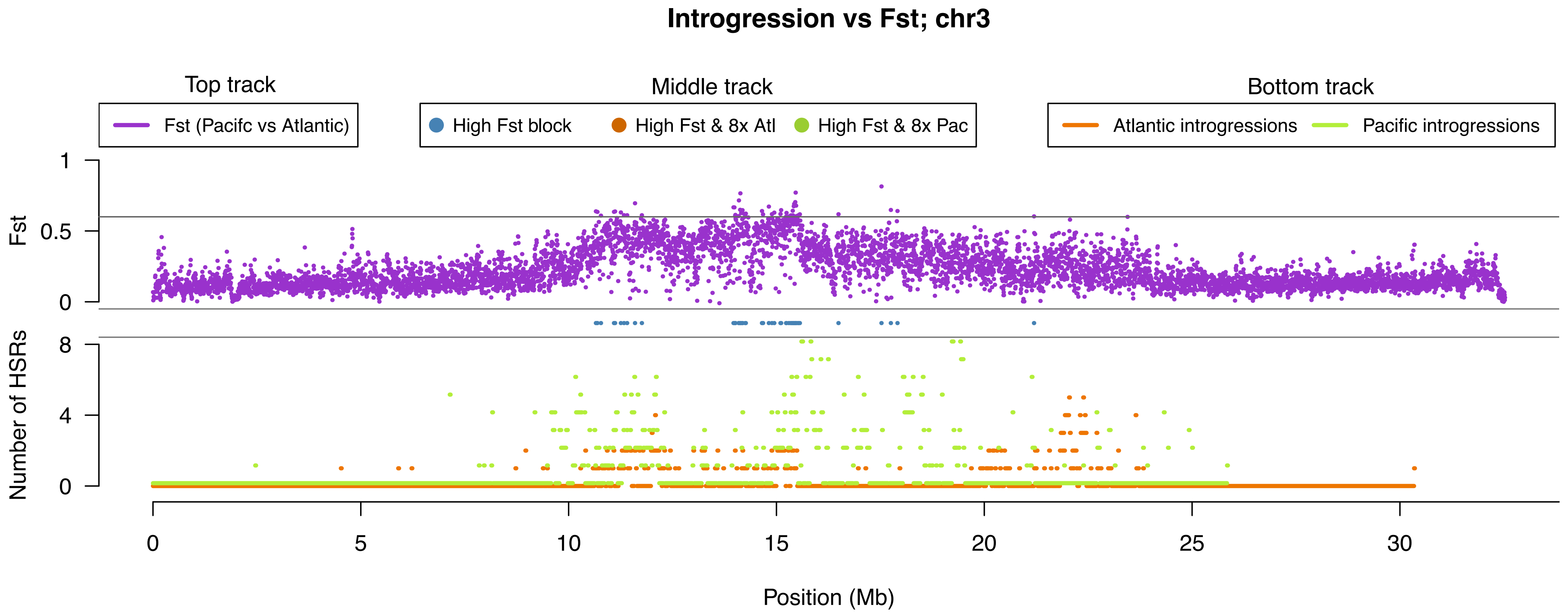

Introgression vs Fst; chr3
Top track
Fst (Pacifc vs Atlantic)
Middle track
High Fst block
High Fst & 8x Atl
High Fst & 8x Pac
Bottom track
Atlantic introgressions
Pacific introgressions
Fst
1
0.5
0
Number of HSRs
8
4
0
0
5
10
15
20
25
30
Position (Mb)

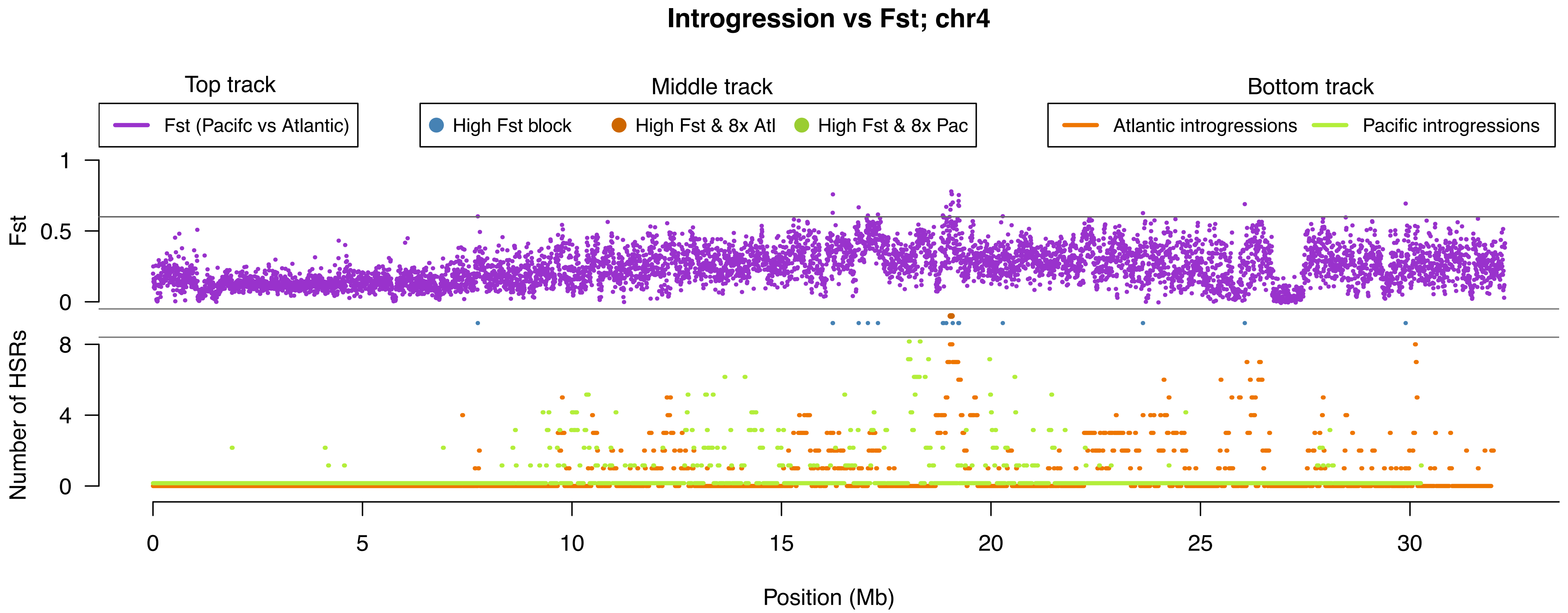

Introgression vs Fst; chr4
Top track
Fst (Pacifc vs Atlantic)
Middle track
High Fst block
High Fst & 8x Atl
High Fst & 8x Pac
Bottom track
Atlantic introgressions
Pacific introgressions
Fst
0
0.5
1
Number of HSRs
0
4
8
0
5
10
15
20
25
30
Position (Mb)

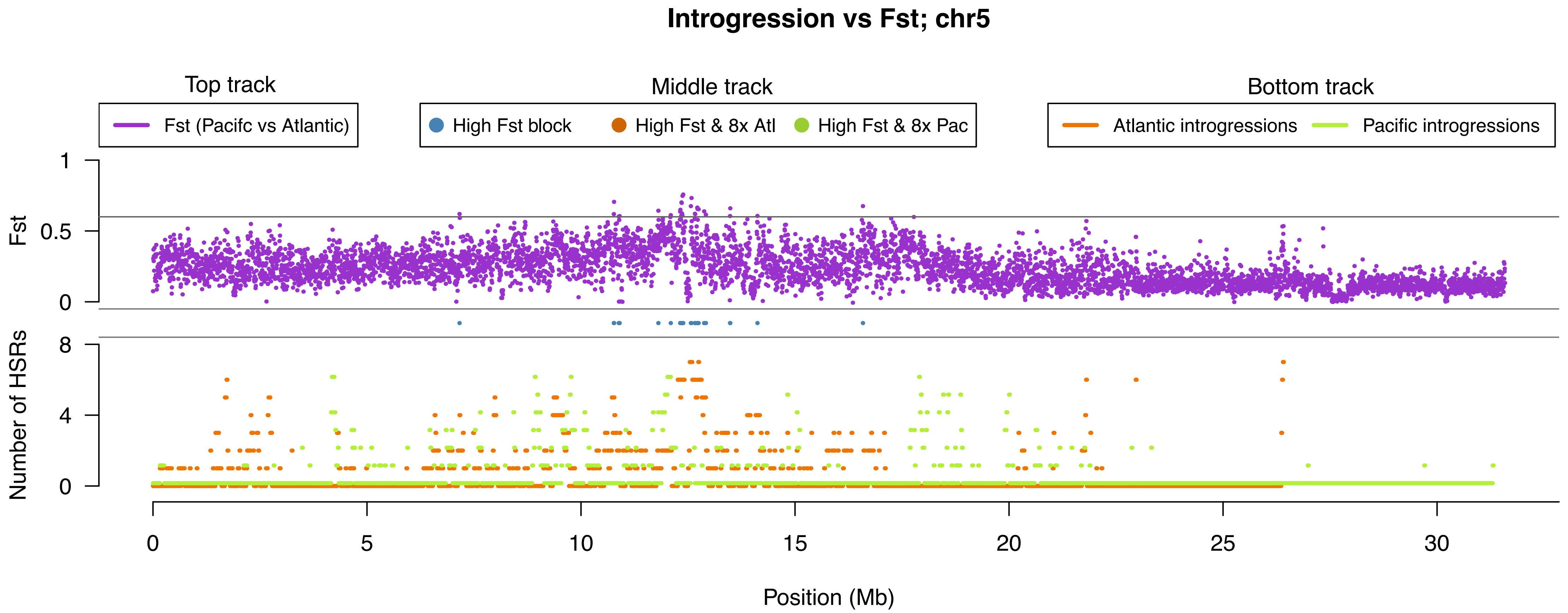
Introgression vs Fst; chr5
Top track
Fst (Pacifc vs Atlantic)
Middle track
High Fst block
High Fst & 8x Atl
High Fst & 8x Pac
Bottom track
Atlantic introgressions
Pacific introgressions
Fst
0
0.5
1
Number of HSRs
0
4
8
0
5
10
15
20
25
30
Position (Mb)

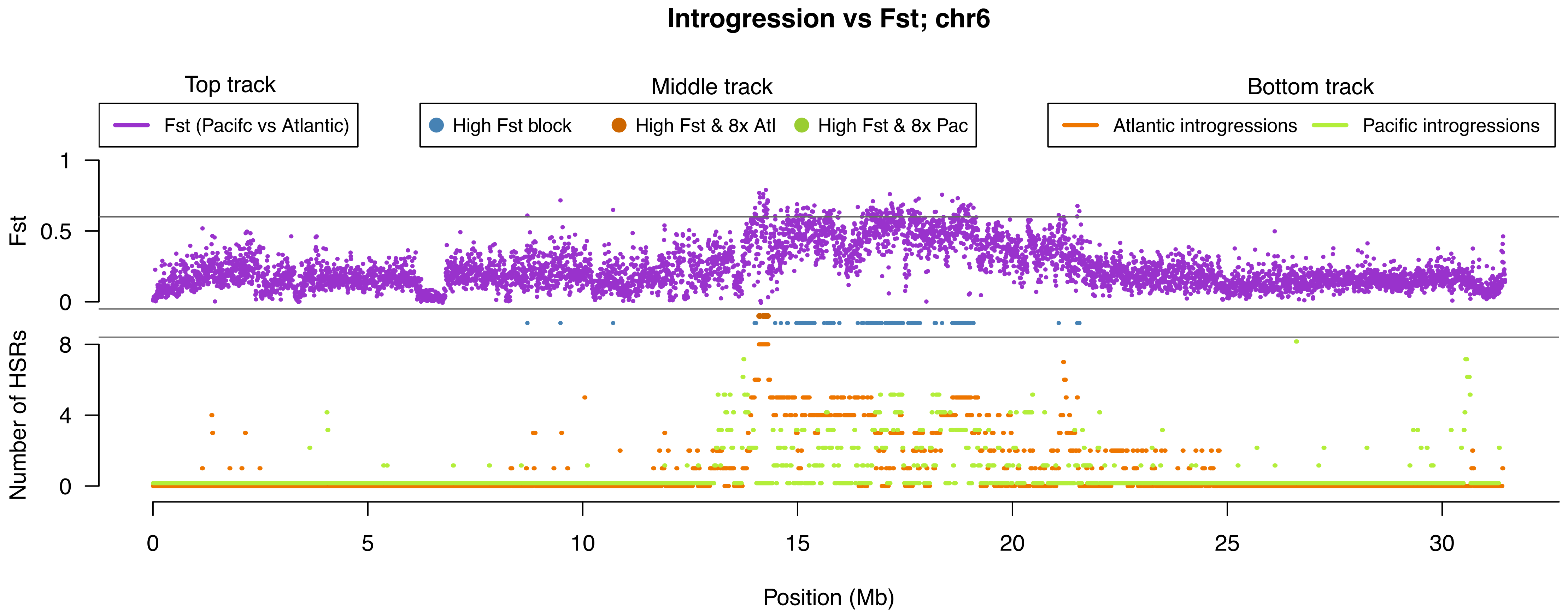

Introgression vs Fst; chr6
Top track
Fst (Pacifc vs Atlantic)
Middle track
High Fst block
High Fst & 8x Atl
High Fst & 8x Pac
Bottom track
Atlantic introgressions
Pacific introgressions
Fst
1
0.5
0
Number of HSRs
8
4
0
0
5
10
15
20
25
30
Position (Mb)

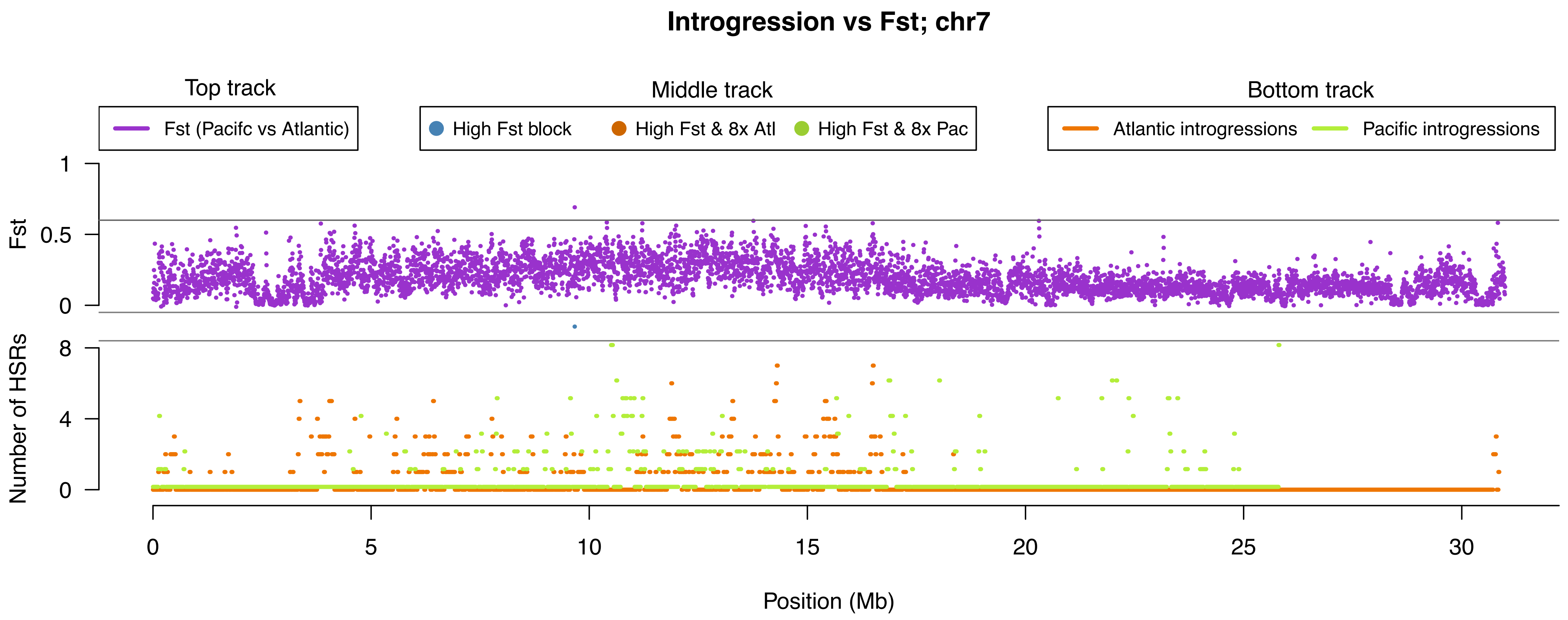

Introgression vs Fst; chr7
Top track
Fst (Pacifc vs Atlantic)
Middle track
High Fst block
High Fst & 8x Atl
High Fst & 8x Pac
Bottom track
Atlantic introgressions
Pacific introgressions
Fst
1
0.5
0
Number of HSRs
8
4
0
0
5
10
15
20
25
30
Position (Mb)

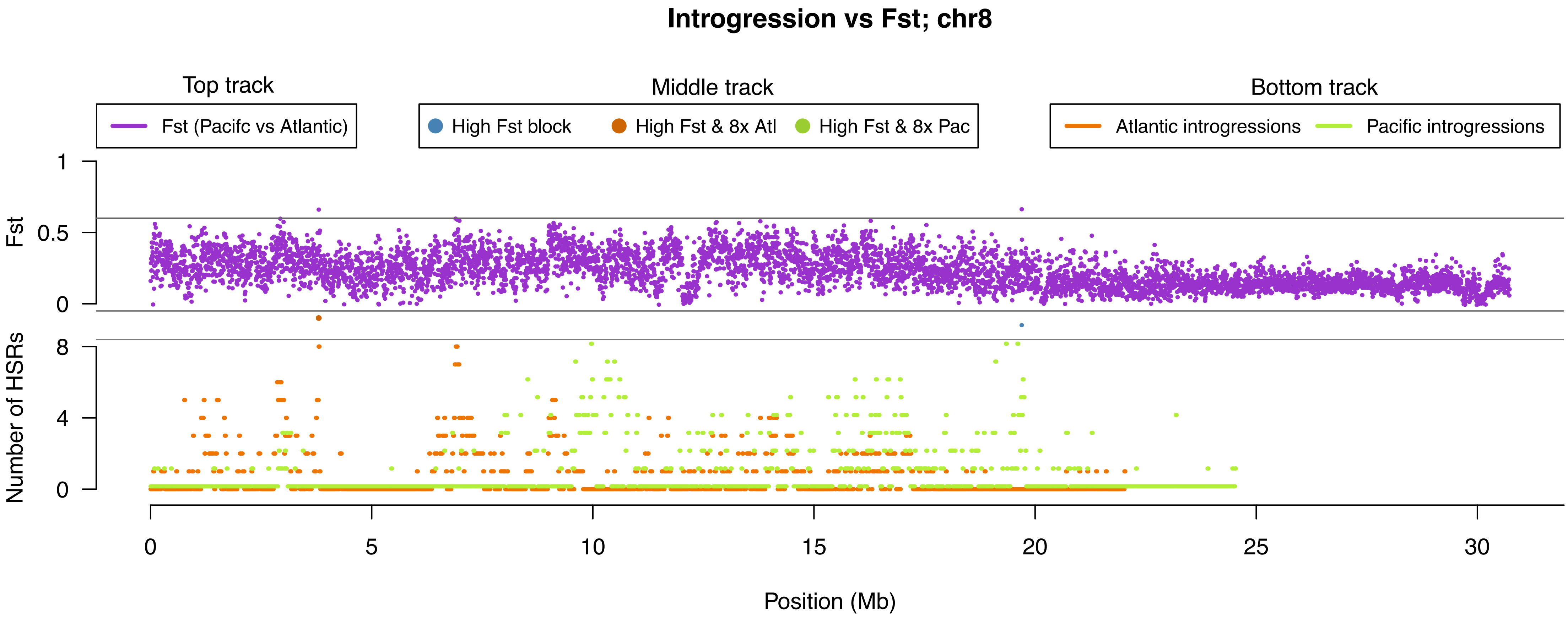
Introgression vs Fst; chr8
Top track
Fst (Pacifc vs Atlantic)
Middle track
High Fst block
High Fst & 8x Atl
High Fst & 8x Pac
Bottom track
Atlantic introgressions
Pacific introgressions
Fst
1
0.5
0
Number of HSRs
8
4
0
0
5
10
15
20
25
30
Position (Mb)

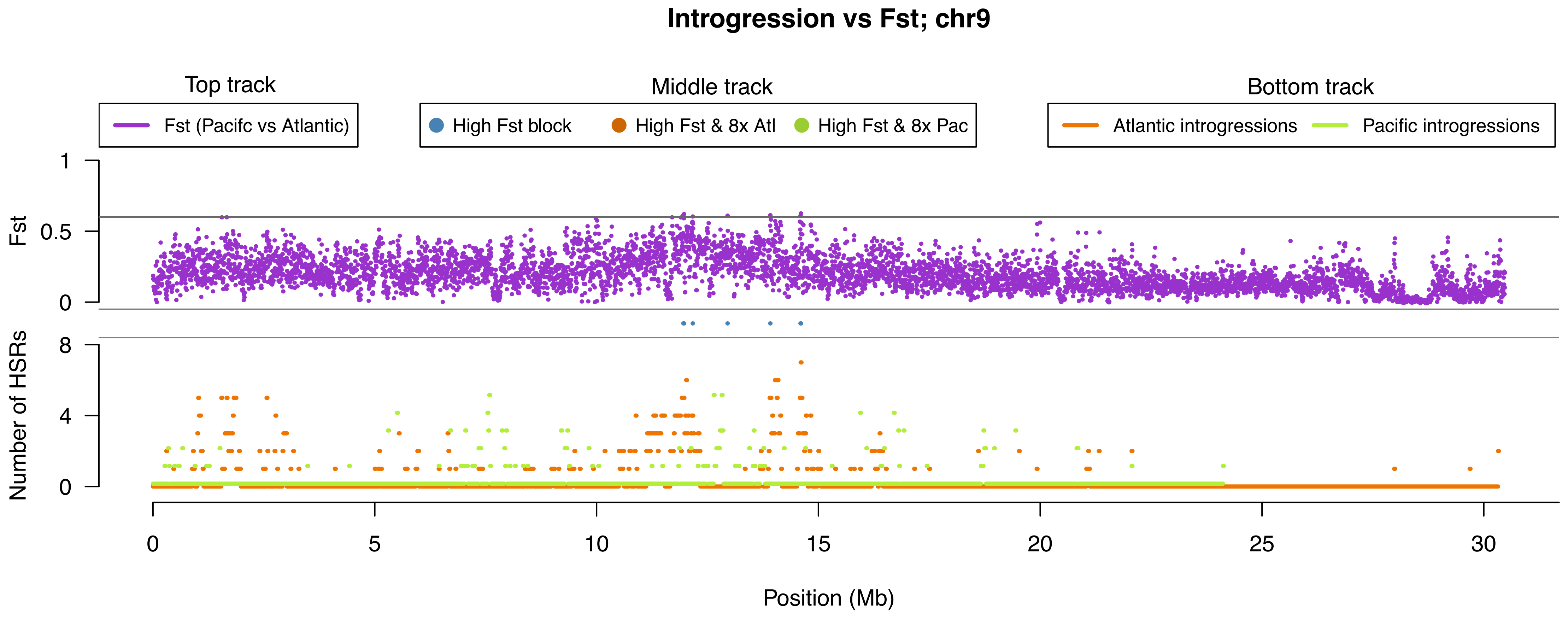

Introgression vs Fst; chr9
Top track
Fst (Pacifc vs Atlantic)
Middle track
High Fst block
High Fst & 8x Atl
High Fst & 8x Pac
Bottom track
Atlantic introgressions
Pacific introgressions
Fst
Number of HSRs
Position (Mb)

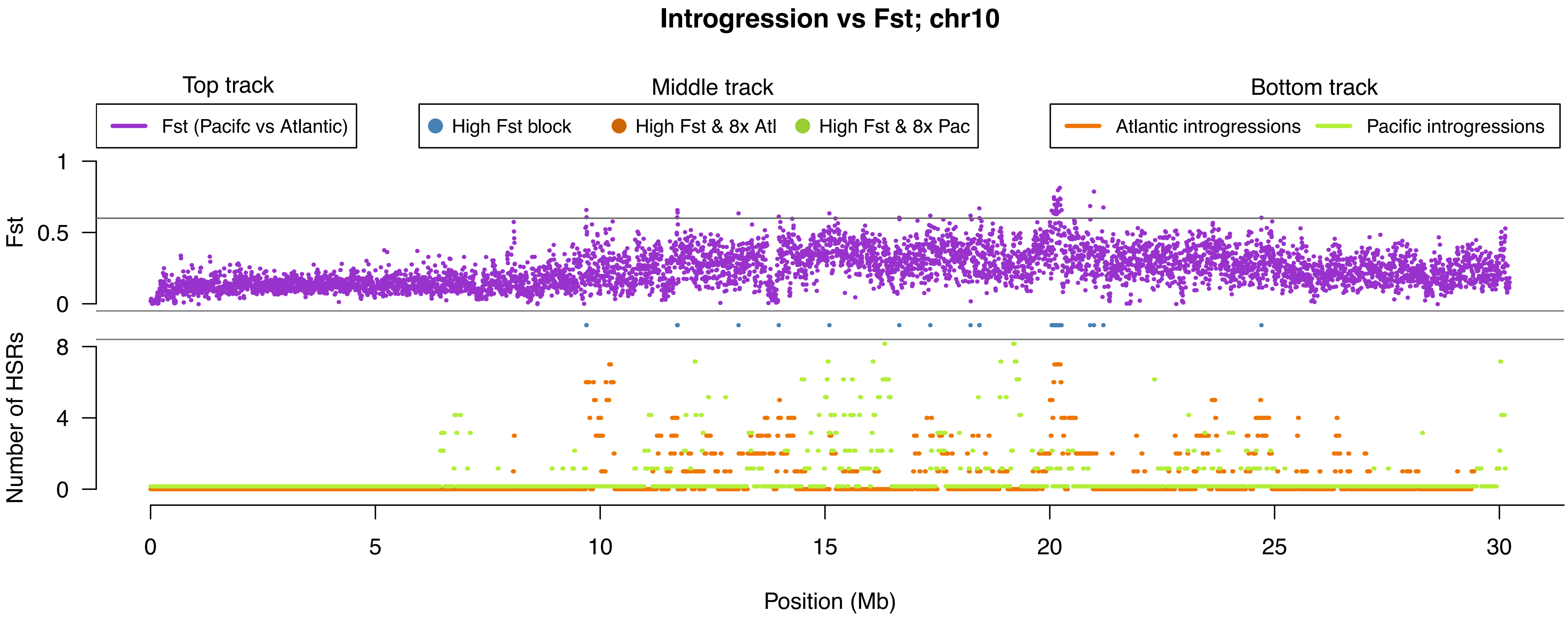

Introgression vs Fst; chr10
Top track
Fst (Pacifc vs Atlantic)
Middle track
High Fst block
High Fst & 8x Atl
High Fst & 8x Pac
Bottom track
Atlantic introgressions
Pacific introgressions
Fst
1
0.5
0
Number of HSRs
8
4
0
0
5
10
15
20
25
30
Position (Mb)

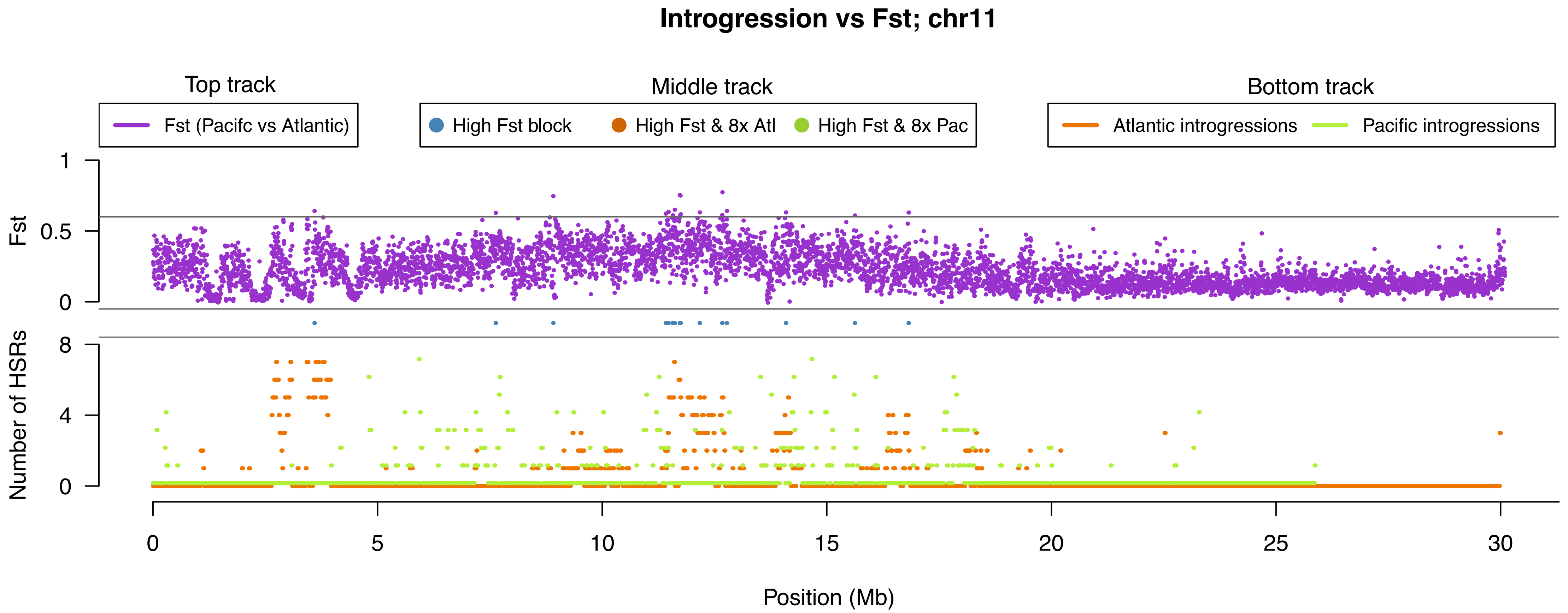
Introgression vs Fst; chr11
Top track
Fst (Pacifc vs Atlantic)
Middle track
High Fst block
High Fst & 8x Atl
High Fst & 8x Pac
Bottom track
Atlantic introgressions
Pacific introgressions
Fst
Number of HSRs
Position (Mb)
1
0.5
0
8
4
0
0
5
10
15
20
25
30

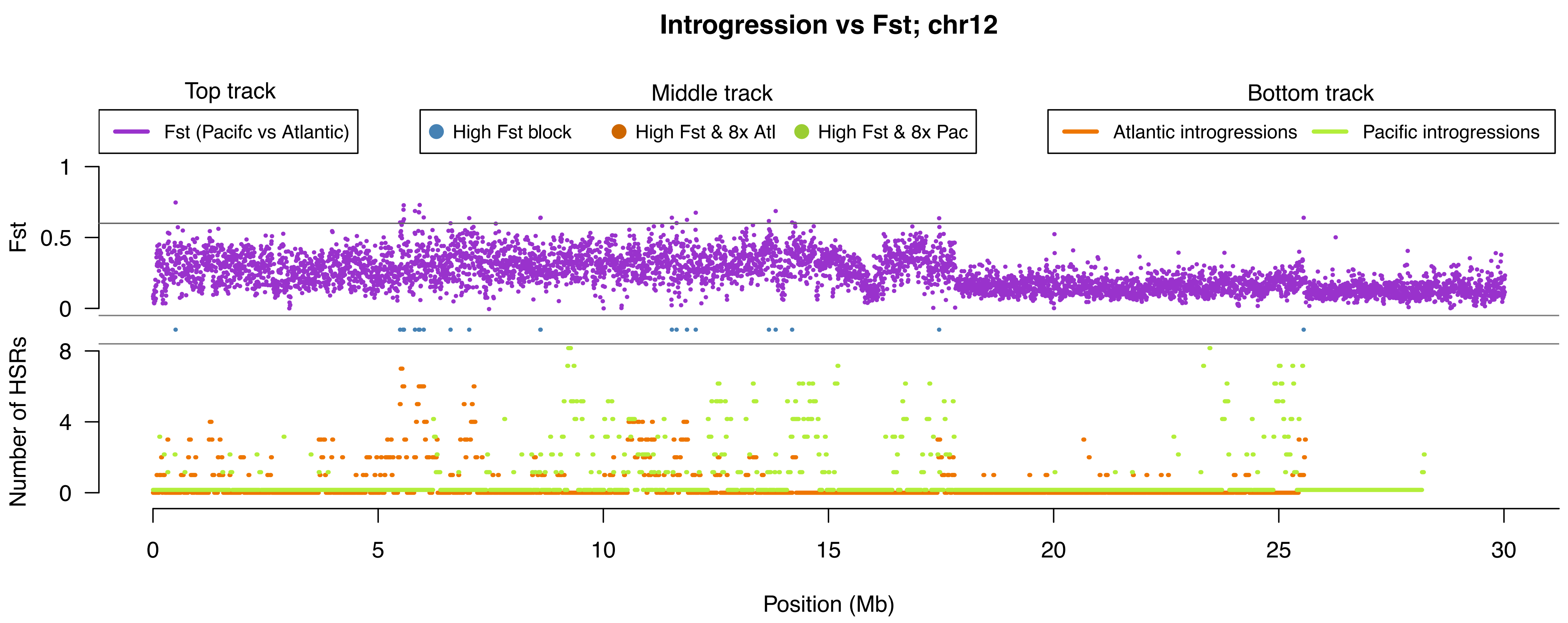
Introgression vs Fst; chr12
Top track
Fst (Pacifc vs Atlantic)
Middle track
High Fst block
High Fst & 8x Atl
High Fst & 8x Pac
Bottom track
Atlantic introgressions
Pacific introgressions
Fst
1
0.5
0
Number of HSRs
8
4
0
0
5
10
15
20
25
30
Position (Mb)

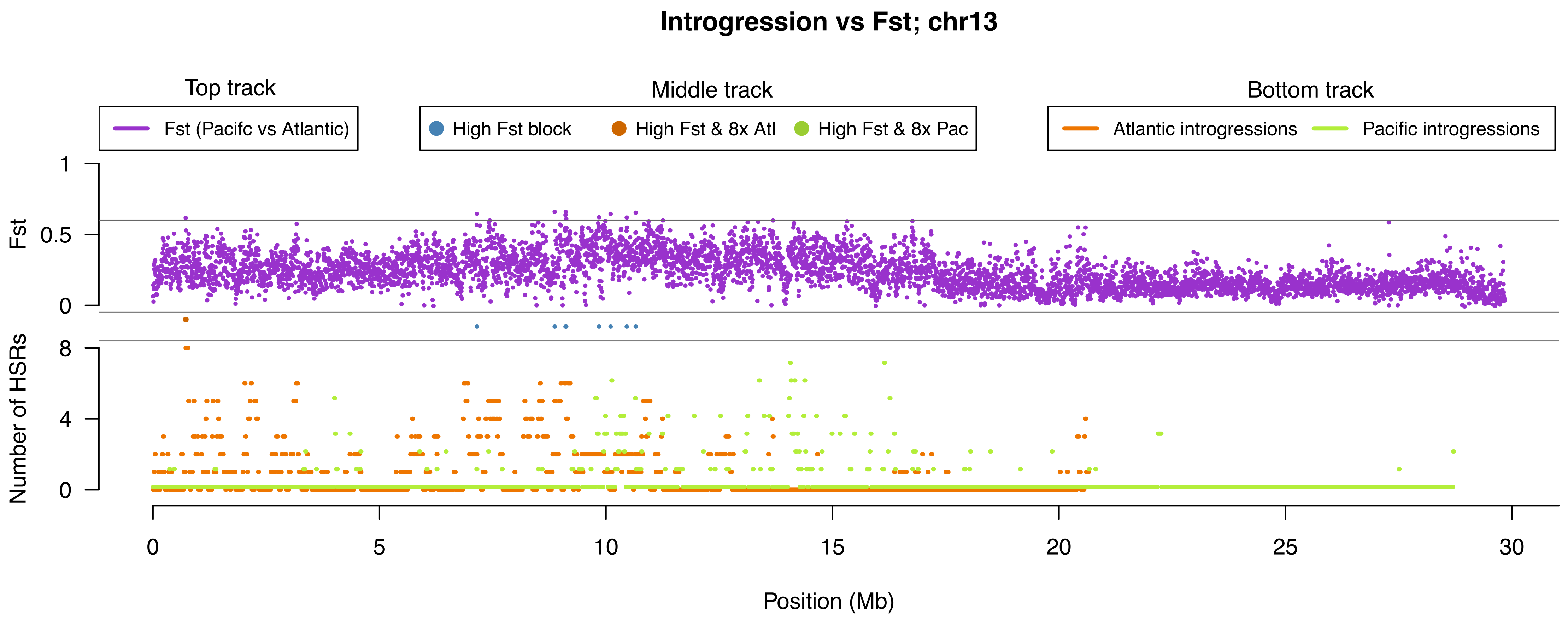

Introgression vs Fst; chr13
Top track
Fst (Pacifc vs Atlantic)
Middle track
High Fst block
High Fst & 8x Atl
High Fst & 8x Pac
Bottom track
Atlantic introgressions
Pacific introgressions
Fst
1
0.5
0
Number of HSRs
8
4
0
0
5
10
15
20
25
30
Position (Mb)

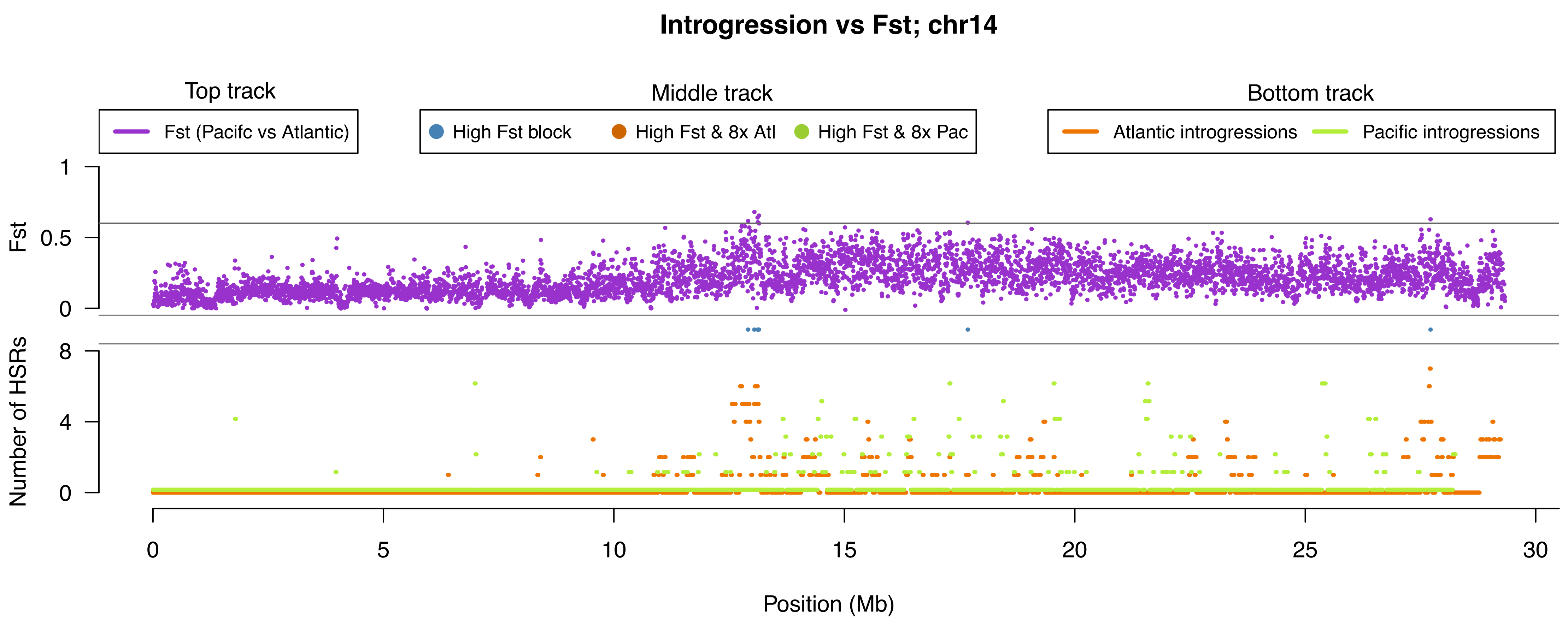

Introgression vs Fst; chr14
Top track
Fst (Pacifc vs Atlantic)
Middle track
High Fst block
High Fst & 8x Atl
High Fst & 8x Pac
Bottom track
Atlantic introgressions
Pacific introgressions
Fst
1
0.5
0
Number of HSRs
8
4
0
0
5
10
15
20
25
30
Position (Mb)

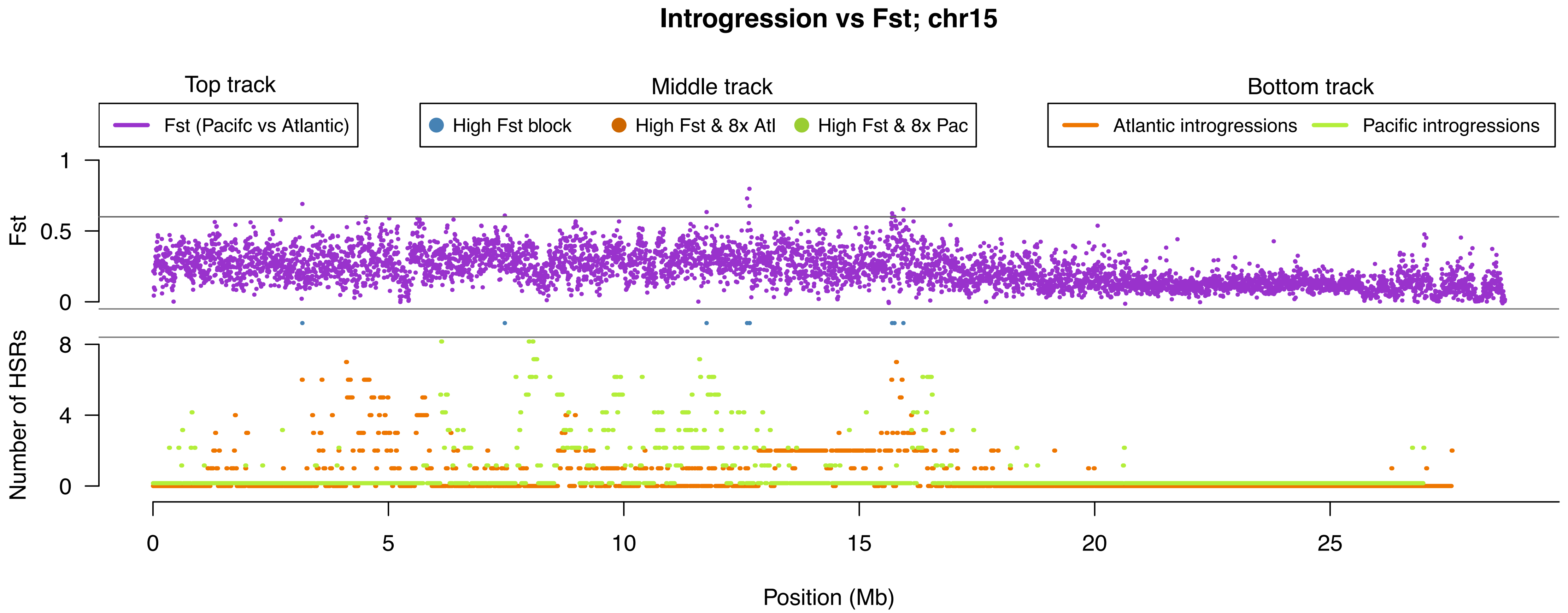

Introgression vs Fst; chr15
Top track
Fst (Pacifc vs Atlantic)
Middle track
High Fst block
High Fst & 8x Atl
High Fst & 8x Pac
Bottom track
Atlantic introgressions
Pacific introgressions
Fst
1
0.5
0
Number of HSRs
8
4
0
0
5
10
15
20
25
Position (Mb)

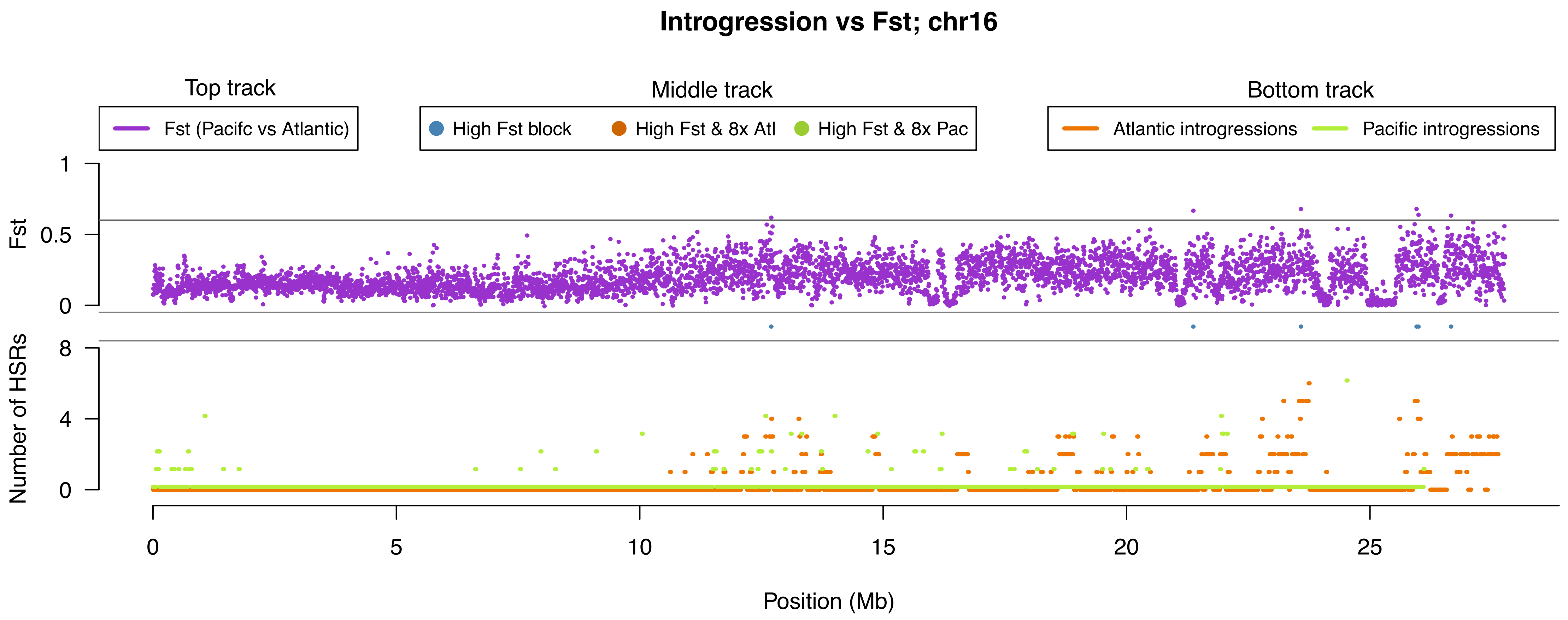
Introgression vs Fst; chr16
Top track
Fst (Pacifc vs Atlantic)
Middle track
High Fst block
High Fst & 8x Atl
High Fst & 8x Pac
Bottom track
Atlantic introgressions
Pacific introgressions
Fst
1
0.5
0
Number of HSRs
8
4
0
0
5
10
15
20
25
Position (Mb)

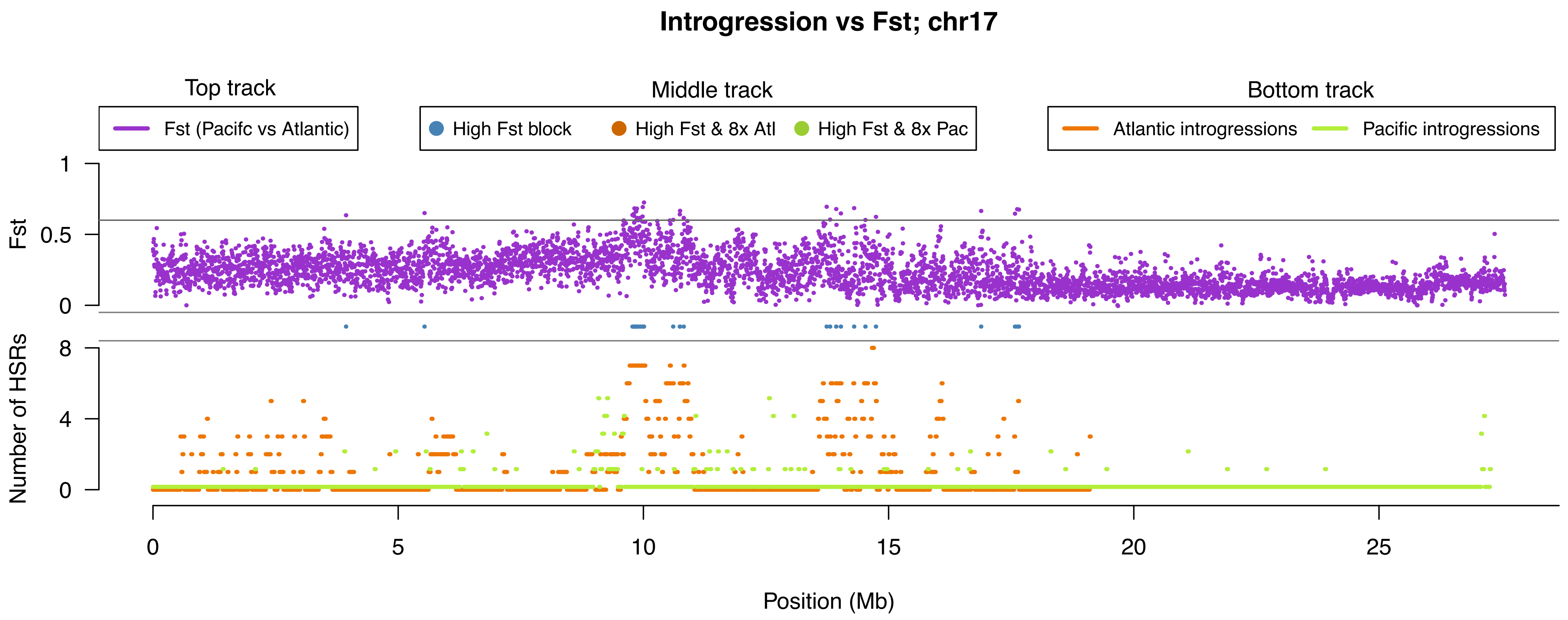

Introgression vs Fst; chr17
Top track
Fst (Pacifc vs Atlantic)
Middle track
High Fst block
High Fst & 8x Atl
High Fst & 8x Pac
Bottom track
Atlantic introgressions
Pacific introgressions
Fst
1
0.5
0
Number of HSRs
8
4
0
0
5
10
15
20
25
Position (Mb)

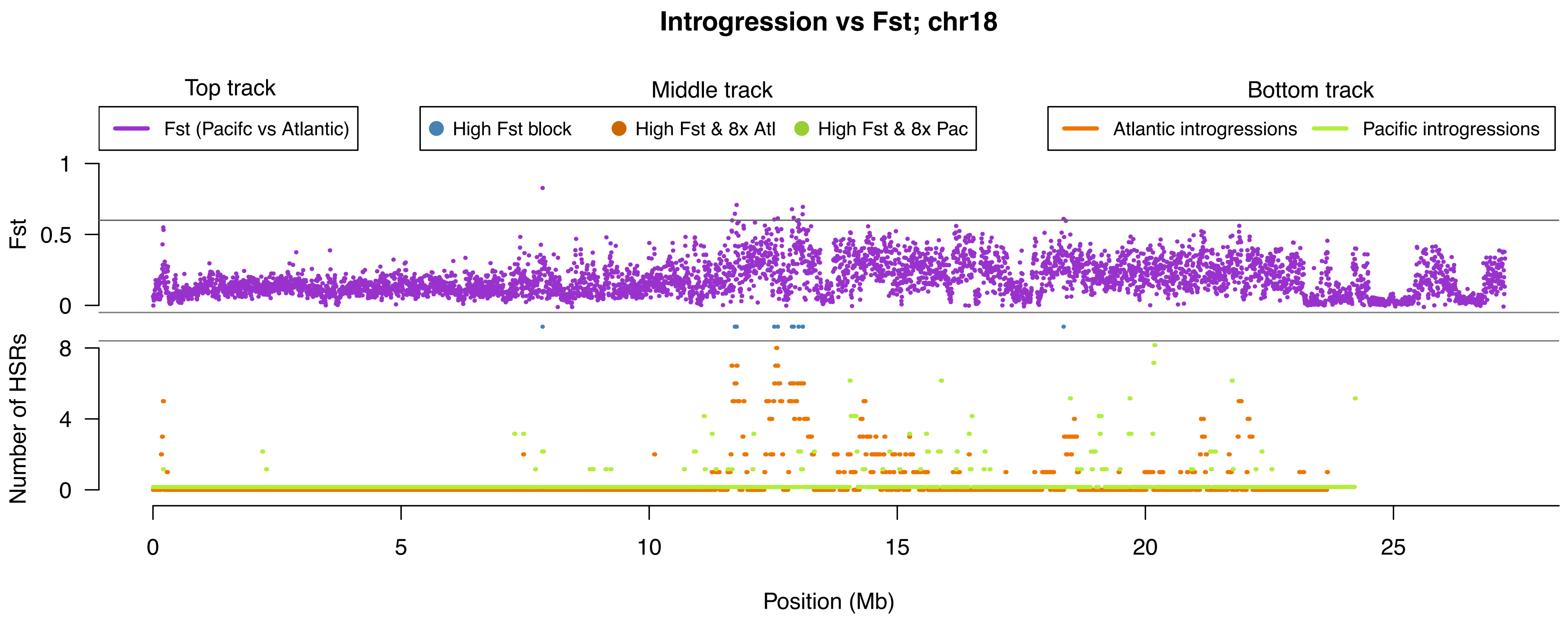
Introgression vs Fst; chr18
Top track
Fst (Pacifc vs Atlantic)
Middle track
High Fst block
High Fst & 8x Atl
High Fst & 8x Pac
Bottom track
Atlantic introgressions
Pacific introgressions
Fst
1
0.5
0
Number of HSRs
8
4
0
0
5
10
15
20
25
Position (Mb)

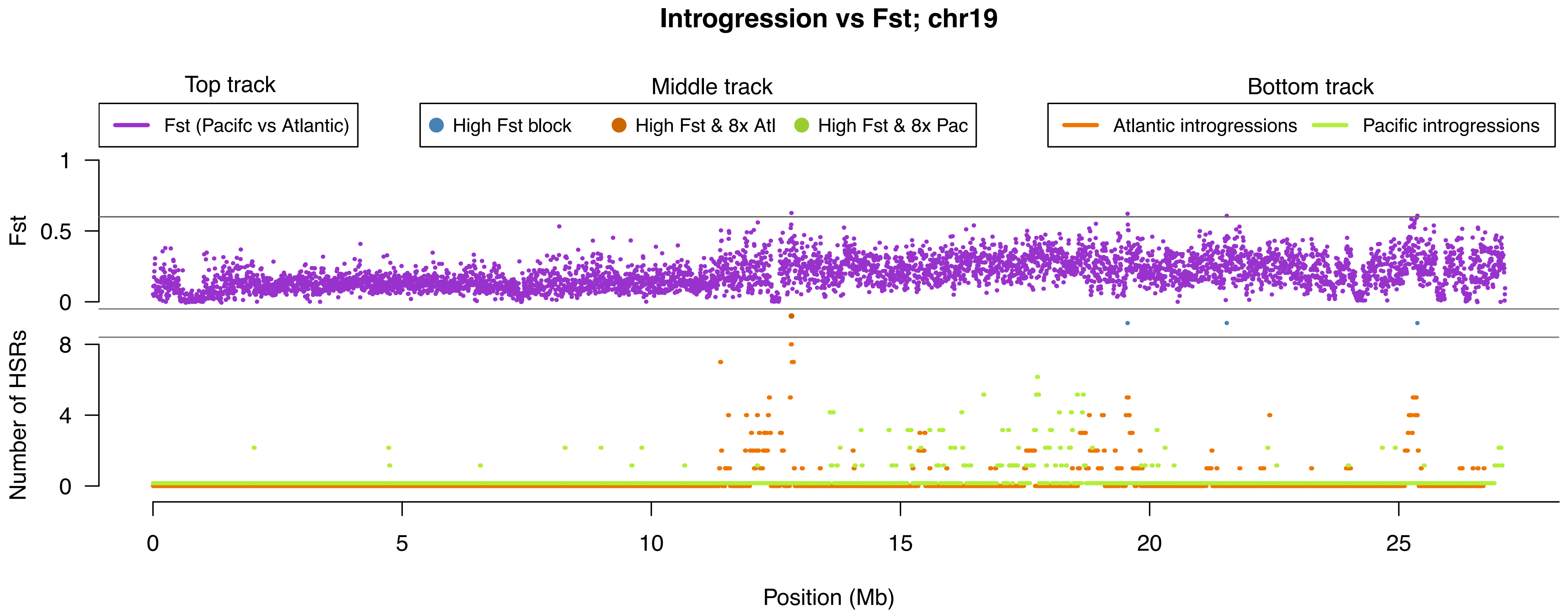

Introgression vs Fst; chr19
Top track
Fst (Pacifc vs Atlantic)
Middle track
High Fst block
High Fst & 8x Atl
High Fst & 8x Pac
Bottom track
Atlantic introgressions
Pacific introgressions
Fst
1
0.5
0
Number of HSRs
8
4
0
0
5
10
15
20
25
Position (Mb)

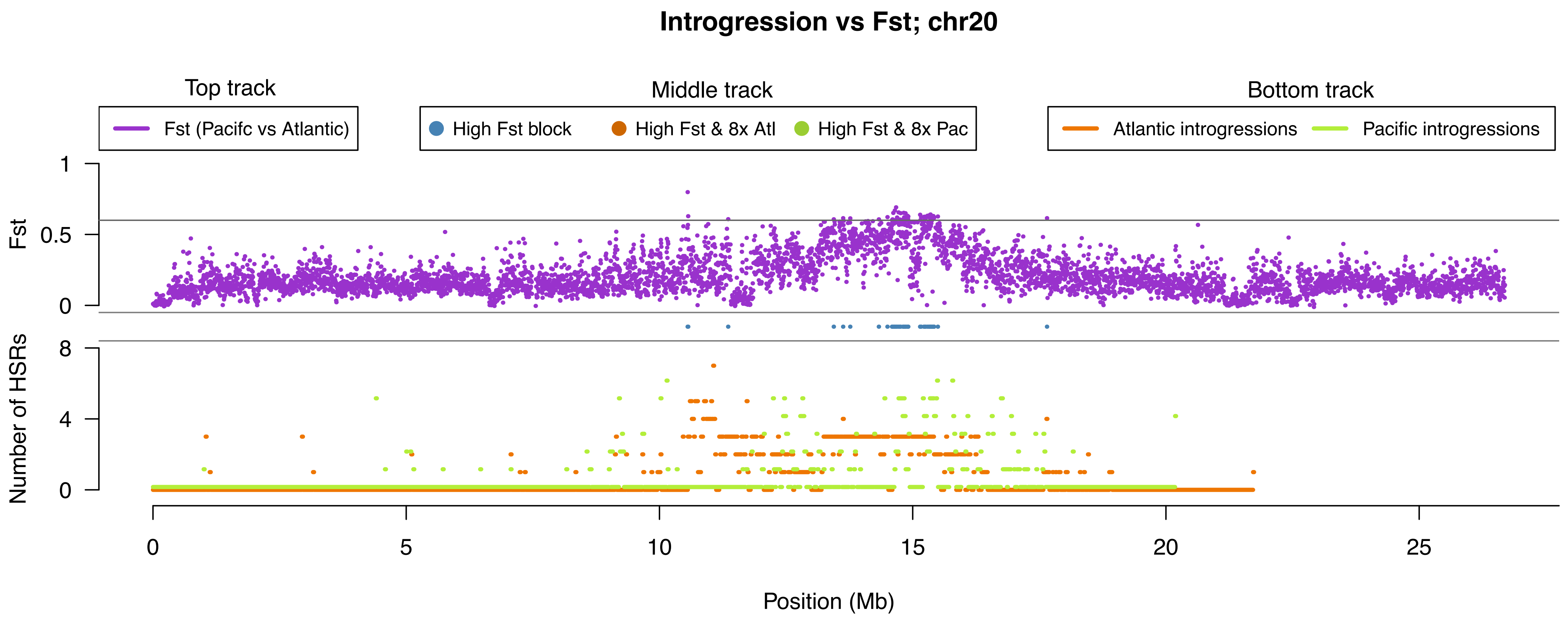

Introgression vs Fst; chr20
Top track
Fst (Pacifc vs Atlantic)
Middle track
High Fst block
High Fst & 8x Atl
High Fst & 8x Pac
Bottom track
Atlantic introgressions
Pacific introgressions
Fst
1
0.5
0
Number of HSRs
8
4
0
0
5
10
15
20
25
Position (Mb)

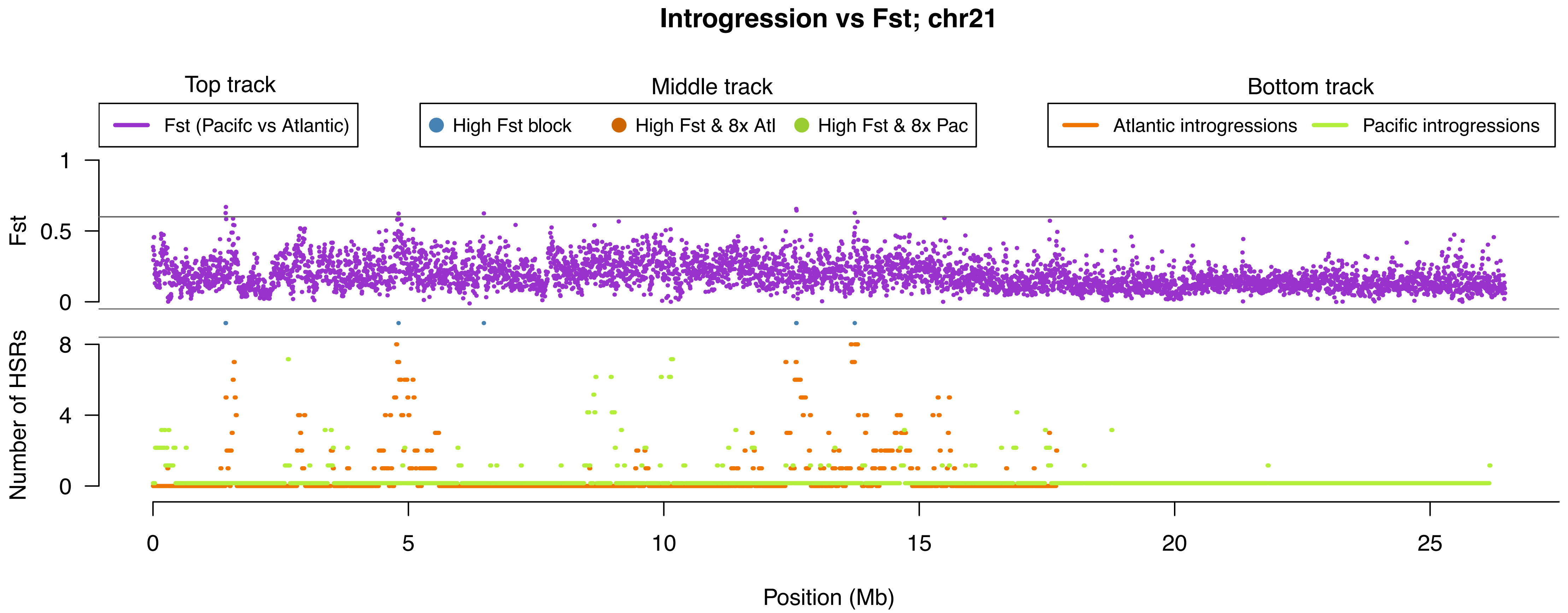
Introgression vs Fst; chr21
Top track
Fst (Pacifc vs Atlantic)
Middle track
High Fst block
High Fst & 8x Atl
High Fst & 8x Pac
Bottom track
Atlantic introgressions
Pacific introgressions
Fst
1
0.5
0
Number of HSRs
8
4
0
0
5
10
15
20
25
Position (Mb)

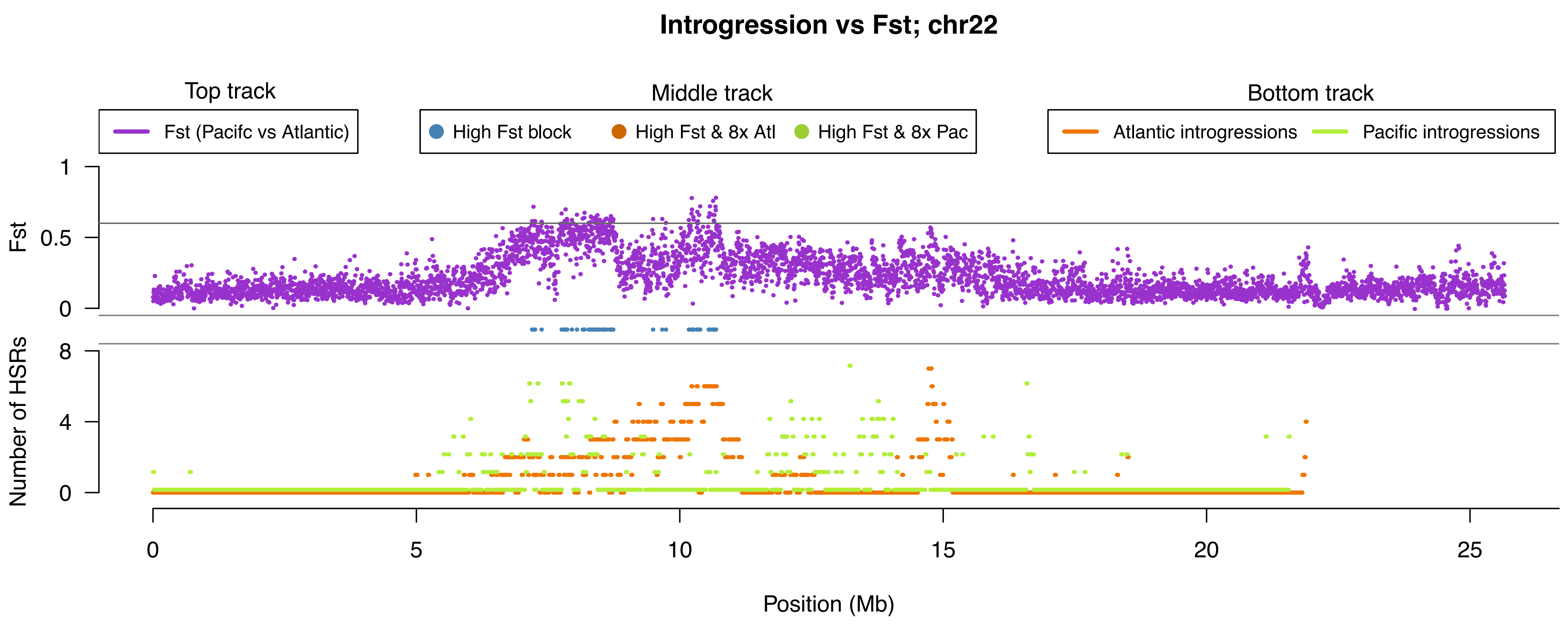
Introgression vs Fst; chr22
Top track
Fst (Pacifc vs Atlantic)
Middle track
High Fst block
High Fst & 8x Atl
High Fst & 8x Pac
Bottom track
Atlantic introgressions
Pacific introgressions
Fst
0
0.5
1
Number of HSRs
0
4
8
0
5
10
15
20
25
Position (Mb)

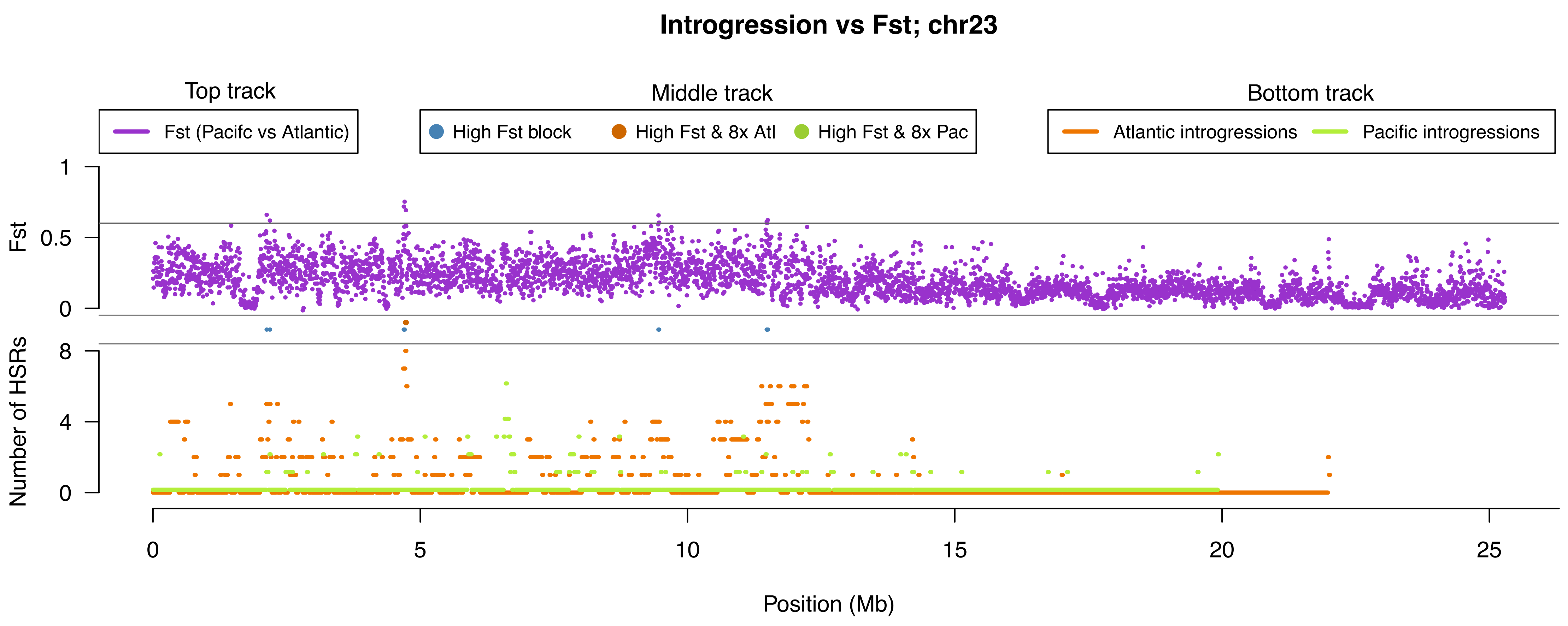

Introgression vs Fst; chr23
Top track
Fst (Pacifc vs Atlantic)
Middle track
High Fst block
High Fst & 8x Atl
High Fst & 8x Pac
Bottom track
Atlantic introgressions
Pacific introgressions
Fst
1
0.5
0
Number of HSRs
8
4
0
0
5
10
15
20
25
Position (Mb)

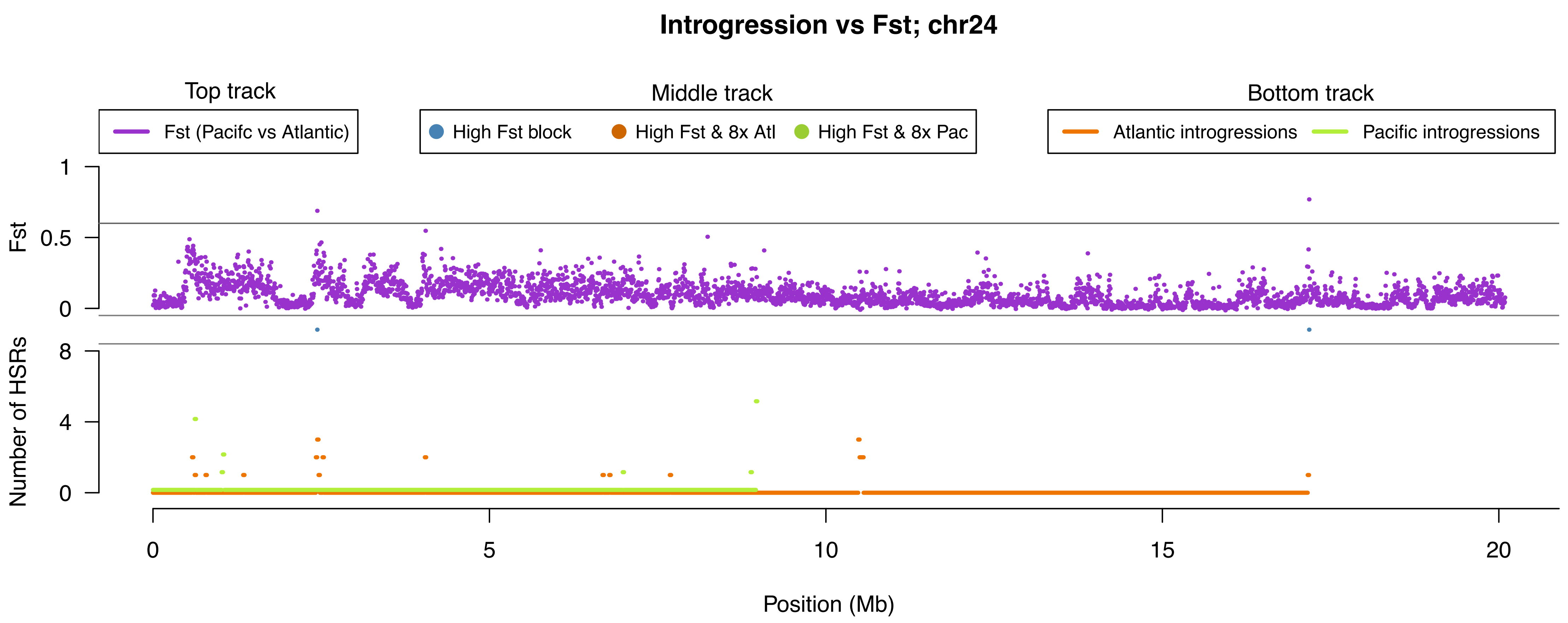

Introgression vs Fst; chr24
Top track
Fst (Pacifc vs Atlantic)
Middle track
High Fst block
High Fst & 8x Atl
High Fst & 8x Pac
Bottom track
Atlantic introgressions
Pacific introgressions
Fst
1
0.5
0
Number of HSRs
8
4
0
0
5
10
15
20
Position (Mb)

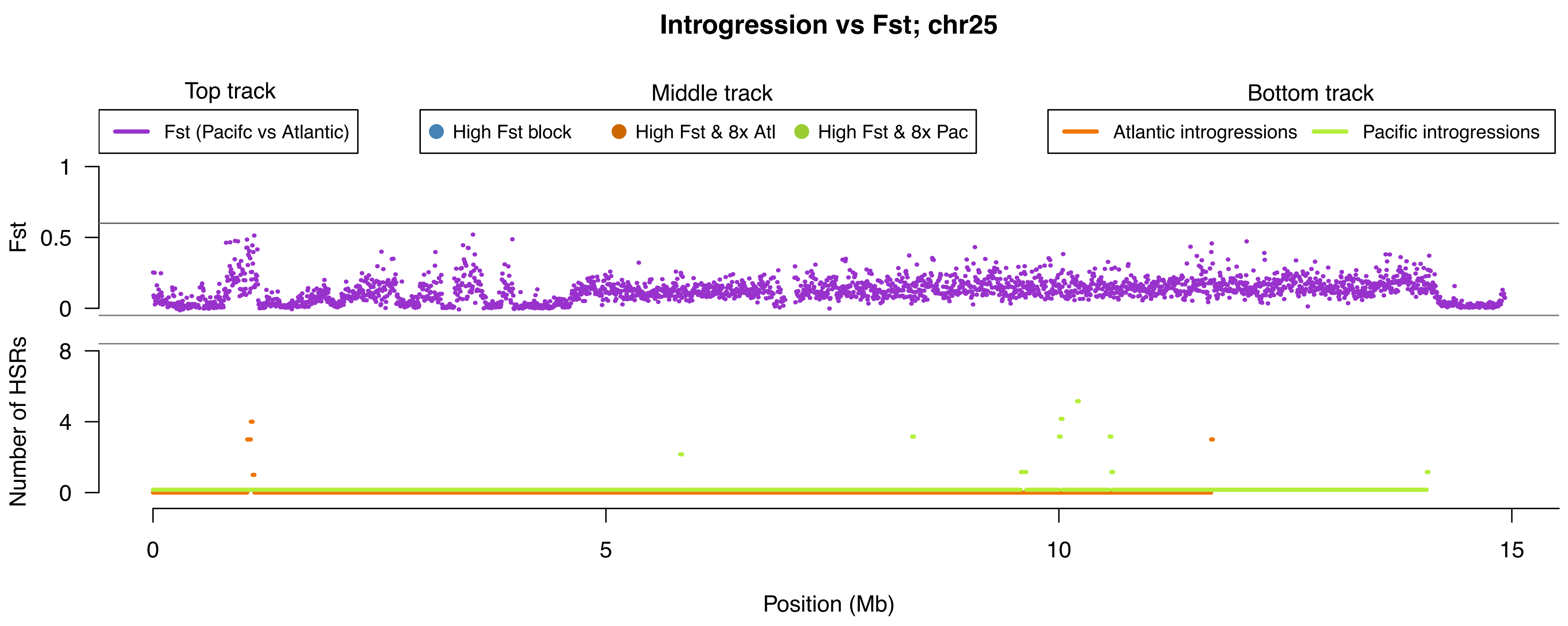

Introgression vs Fst; chr25
Top track
Fst (Pacifc vs Atlantic)
Middle track
High Fst block
High Fst & 8x Atl
High Fst & 8x Pac
Bottom track
Atlantic introgressions
Pacific introgressions
Fst
1
0.5
0
Number of HSRs
8
4
0
0
5
10
15
Position (Mb)

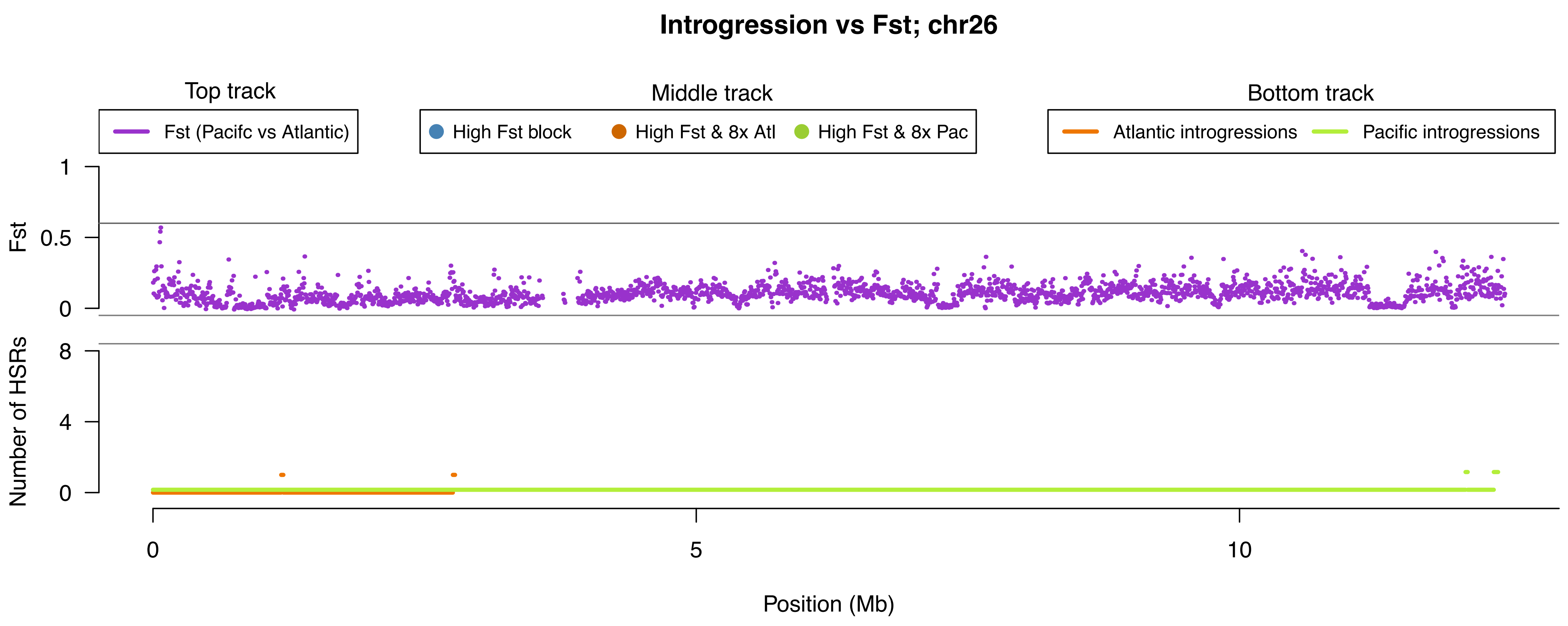

Introgression vs Fst; chr26
Top track
Fst (Pacifc vs Atlantic)
Middle track
High Fst block
High Fst & 8x Atl
High Fst & 8x Pac
Bottom track
Atlantic introgressions
Pacific introgressions
Fst
1
0.5
0
Number of HSRs
8
4
0
0
5
10
Position (Mb)
