# Supplementary material for: A Long-Standing Hybrid Population Between Pacific and Atlantic Herring in a Subarctic Fjord of Norway
Source: Genome Biol Evol. 2023 Apr 30;15(5):evad069. doi: 10.1093/gbe/evad069 (PMC10182735; doi:10.1093/gbe/evad069)
Supplement: evad069_Supplementary_Data [file evad069_supplementary_data.zip › Supplementary_Tables.docx]

| **Supplementary Table 2:** Nucleotide diversity (*π*) in sample groups, of Atlantic and Pacific herring, based on individual samples. | | | | |
| --- | --- | --- | --- | --- |
| **Population** | ***π* (%)** | **SD (%)** | ***n*** |  |
| Balsfjord | 0.35 | 0.30 | 4 |  |
| NW Pacific & European Pacific herring* | 0.30 | 0.26 | 20 |  |
| NE Pacific herring | 0.28 | 0.25 | 6 |  |
| Atlantic herring (spring) | 0.25 | 0.26 | 17 |  |
| Kandalaksha Bay, White Sea | 0.29 | 0.27 | 8 |  |
| Onega Bay, White Sea | 0.30 | 0.28 | 4 |  |
| Pechora Sea | 0.28 | 0.29 | 4 |  |
| Sea of Japan (NW Pacific) | 0.27 | 0.28 | 4 |  |

*Includes all individuals from White Sea, Pechora Sea and Sea of Japan.

**Supplementary Table 3.** Ancestry coefficients of 79 individuals estimated with the program sNMF for K = 2, Q1 is the proportion of Pacific ancestry and Q2 is Atlantic ancestry. Balsfjord samples are indicated by italics.

| **Q1** | **Q2** | **Region** | **Ind** | |
| --- | --- | --- | --- | --- |
| 3.42E-03 | 1.00 | Baltic Sea | BF16 | |
| 2.83E-03 | 1.00 | Baltic Sea | BF18 | |
| 6.25E-03 | 0.99 | Baltic Sea | BF19 | |
| 5.17E-03 | 0.99 | Baltic Sea | BF21 | |
| 5.81E-03 | 0.99 | Baltic Sea | BM14 | |
| 2.40E-03 | 1.00 | Baltic Sea | BM15 | |
| 2.43E-03 | 1.00 | Baltic Sea | BM16 | |
| 3.63E-03 | 1.00 | Baltic Sea | BM19 | |
| 1.00E-04 | 1.00 | Baltic Sea | Fehmarn3 | |
| 1.00E-04 | 1.00 | Baltic Sea | Fehmarn44 | |
| 2.47E-03 | 1.00 | Baltic Sea | Fehmarn6 | |
| 3.33E-03 | 1.00 | Baltic Sea | Gävle100 | |
| 6.17E-03 | 0.99 | Baltic Sea | Gävle54 | |
| 4.33E-03 | 1.00 | Baltic Sea | Gävle98 | |
| 1.00E-04 | 1.00 | Atlantic | AAL1 | |
| 8.77E-03 | 0.99 | Atlantic | AAL2 | |
| 4.46E-03 | 1.00 | Atlantic | AAL3 | |
| 2.82E-03 | 1.00 | Atlantic | AF29 | |
| 8.50E-03 | 0.99 | Atlantic | AF30 | |
| 9.44E-03 | 0.99 | Atlantic | AF31 | |
| 8.65E-03 | 0.99 | Atlantic | AF8 | |
| 1.14E-02 | 0.99 | Atlantic | AK1 | |
| 8.34E-03 | 0.99 | Atlantic | AK2 |  |
| 8.58E-03 | 0.99 | Atlantic | AK3 |  |
| 9.25E-03 | 0.99 | Atlantic | AM27 |  |
| 1.87E-03 | 1.00 | Atlantic | AM29 |  |
| 1.09E-02 | 0.99 | Atlantic | AM33 |  |
| 4.70E-03 | 1.00 | Atlantic | AM8 |  |
| 6.20E-03 | 0.99 | Atlantic | F3L312 |  |
| 6.28E-03 | 0.99 | Atlantic | F3L337 |  |
| 9.09E-03 | 0.99 | Atlantic | F4TH327 |  |
| 1.00E-04 | 1.00 | Atlantic | F4TH337 |  |
| 1.21E-02 | 0.99 | Atlantic | F4XQgb402 |  |
| 9.45E-03 | 0.99 | Atlantic | F4XQgb408 |  |
| 1.00E-04 | 1.00 | Atlantic | NSSH33 |  |
| 3.14E-03 | 1.00 | Atlantic | NSSH34 |  |
| 3.76E-03 | 1.00 | Atlantic | NSSH36 |  |
| 1.27E-03 | 1.00 | Atlantic | NorthSea13 |  |
| 9.08E-03 | 0.99 | Atlantic | NorthSea19 |  |
| 1.00E-04 | 1.00 | Atlantic | NorthSea34 |  |
| 7.44E-03 | 0.99 | Atlantic | S3Ps246 |  |
| 4.00E-03 | 1.00 | Atlantic | S3Ps259 |  |
| 7.09E-03 | 0.99 | Atlantic | S4TH231 |  |
| 3.62E-03 | 1.00 | Atlantic | S4TH244 |  |
| 5.21E-03 | 0.99 | Atlantic | S4TM205 |  |
| 4.83E-03 | 1.00 | Atlantic | S4TM211 |  |
| 9.61E-03 | 0.99 | Atlantic | Z12 |  |
| 1.00E-04 | 1.00 | Atlantic | Z14 |  |
| 1.00E-04 | 1.00 | Atlantic | Z4 |  |
| 1.00 | 1.00E-04 | NW Pacific (Sea of Japan) | HWS11 | |
| 1.00 | 1.00E-04 | NW Pacific (Sea of Japan) | HWS12 | |
| 1.00 | 1.00E-04 | NW Pacific (Sea of Japan) | HWS13 | |
| 1.00 | 1.00E-04 | NW Pacific (Sea of Japan) | HWS14 | |
| 0.99 | 9.32E-03 | Pechora Sea | HWS21 | |
| 0.98 | 1.72E-02 | Pechora Sea | HWS22 | |
| 0.99 | 1.27E-02 | Pechora Sea | HWS23 | |
| 1.00 | 3.74E-03 | Pechora Sea | HWS24 | |
| 0.93 | 6.90E-02 | Onega Bay | HWS31 | |
| 0.92 | 7.74E-02 | Onega Bay | HWS32 | |
| 0.92 | 7.82E-02 | Onega Bay | HWS33 | |
| 0.94 | 6.24E-02 | Onega Bay | HWS34 | |
| 0.98 | 2.18E-02 | Kandalaksha Bay (S) | HWS41 | |
| 0.98 | 2.47E-02 | Kandalaksha Bay (S) | HWS42 | |
| 0.97 | 2.81E-02 | Kandalaksha Bay (S) | HWS43 | |
| 0.97 | 2.95E-02 | Kandalaksha Bay (S) | HWS44 | |
| 0.93 | 7.36E-02 | Kandalaksha Bay (U) | HWS51 | |
| 0.93 | 6.83E-02 | Kandalaksha Bay (U) | HWS52 | |
| 0.93 | 6.95E-02 | Kandalaksha Bay (U) | HWS53 | |
| 0.95 | 5.45E-02 | Kandalaksha Bay (U) | HWS54 | |
| *0.74* | *0.26* | Balsfjord | HWS61 | |
| *0.74* | *0.26* | Balsfjord | HWS62 | |
| *0.74* | *0.26* | Balsfjord | HWS63 | |
| *0.75* | *0.25* | Balsfjord | HWS64 | |
| 1.00 | 1.00E-04 | NE Pacific (Vancouver) | Pacific16 | |
| 1.00 | 1.00E-04 | NE Pacific (Vancouver) | Pacific30 | |
| 1.00 | 1.00E-04 | NE Pacific (Vancouver) | Pacific35 | |
| 1.00 | 1.00E-04 | NE Pacific (Vancouver) | Pacific3 | |
| 1.00 | 1.00E-04 | NE Pacific (Vancouver) | Pacific44 | |
| 1.00 | 1.00E-04 | NE Pacific (Vancouver) | Pacific5 | |

**Supplementary Table 5.** Regions with Atlantic of Pacific HSRs (introgressed regions) observed in all eight haploid genomes from Balsfjord.

| **Chr** | **Start (bp)** | **End (bp)** | **Width (bp)** | **Type** |
| --- | --- | --- | --- | --- |
| 4 | 19,020,001 | 19,080,000 | 60,000 | Atlantic |
| 4 | 30,120,001 | 30,140,000 | 20,000 | Atlantic |
| 6 | 14,100,001 | 14,320,000 | 220,000 | Atlantic |
| 8 | 3,800,001 | 3,820,000 | 20,000 | Atlantic |
| 8 | 6,900,001 | 6,940,000 | 40,000 | Atlantic |
| 13 | 720,001 | 780,000 | 60,000 | Atlantic |
| 17 | 14,660,001 | 14,700,000 | 40,000 | Atlantic |
| 18 | 12,560,001 | 12,580,000 | 20,000 | Atlantic |
| 19 | 12,800,001 | 12,820,000 | 20,000 | Atlantic |
| 21 | 4,760,001 | 4,780,000 | 20,000 | Atlantic |
| 21 | 13,660,001 | 13,680,000 | 20,000 | Atlantic |
| 21 | 13,740,001 | 13,800,000 | 60,000 | Atlantic |
| 23 | 4,720,001 | 4,740,000 | 20,000 | Atlantic |
| 1 | 14,300,001 | 14,320,000 | 20,000 | Pacific |
| 1 | 17,400,001 | 17,460,000 | 60,000 | Pacific |
| 1 | 33,000,001 | 33,020,000 | 20,000 | Pacific |
| 3 | 15,600,001 | 15,640,000 | 40,000 | Pacific |
| 3 | 15,820,001 | 15,840,000 | 20,000 | Pacific |
| 3 | 19,220,001 | 19,260,000 | 40,000 | Pacific |
| 3 | 19,420,001 | 19,440,000 | 20,000 | Pacific |
| 4 | 18,040,001 | 18,060,000 | 20,000 | Pacific |
| 4 | 18,300,001 | 18,320,000 | 20,000 | Pacific |
| 6 | 26,600,001 | 26,620,000 | 20,000 | Pacific |
| 7 | 10,500,001 | 10,540,000 | 40,000 | Pacific |
| 7 | 25,800,001 | 25,820,000 | 20,000 | Pacific |
| 8 | 9,960,001 | 9,980,000 | 20,000 | Pacific |
| 8 | 19,340,001 | 19,360,000 | 20,000 | Pacific |
| 8 | 19,600,001 | 19,620,000 | 20,000 | Pacific |
| 10 | 16,320,001 | 16,340,000 | 20,000 | Pacific |
| 10 | 19,180,001 | 19,220,000 | 40,000 | Pacific |
| 12 | 9,220,001 | 9,280,000 | 60,000 | Pacific |
| 12 | 23,460,001 | 23,480,000 | 20,000 | Pacific |
| 15 | 6,120,001 | 6,140,000 | 20,000 | Pacific |
| 15 | 7,980,001 | 8,000,000 | 20,000 | Pacific |
| 15 | 8,060,001 | 8,080,000 | 20,000 | Pacific |
| 18 | 20,180,001 | 20,200,000 | 20,000 | Pacific |
