# Supplementary material for: A Long-Standing Hybrid Population Between Pacific and Atlantic Herring in a Subarctic Fjord of Norway
Source: Genome Biol Evol. 2023 Apr 30;15(5):evad069. doi: 10.1093/gbe/evad069 (PMC10182735; doi:10.1093/gbe/evad069)
Supplement: evad069_Supplementary_Data [file evad069_supplementary_data.zip › Supporting information legends_230119.docx]

**Supporting information**

**Supplementary Figure 1. *F*_ST_, *d_xy_* and *π* for individual chromosomes based on the Balsfjord samples, compared with Atlantic spring-spawning samples.** Black dots represent 5 kb windows, and the red line is a rolling average. In the *π*-panels, the blue line shows Balsfjord *π*, while the red one shows Atlantic *π*.

**Supplementary Figure 2. *F*_ST_, *d_xy_* and *π* for individual chromosomes based on the Balsfjord samples, compared with Pacific herring samples of European Russia and Northwest Pacific.** Black dots represent 5 kb windows, and the red line is a rolling average. In the *π*-panels, the blue line shows Balsfjord *π*, while the red one shows the Pacific herring *π*.

**Supplementary Figure 3. Minimal cross-entropy values.** Values corresponding to assuming 1 to 5 ancestral populations (K=1 to K=5) with the program sNMF, which estimates admixture coefficients based on genotype data.

**Supplementary Figure 4. Admixture analysis of Atlantic, Pacific, and Balsfjord individuals sequenced to high and low depth.** A total of 120 samples and 1 464 528 LD-pruned SNP markers were included in the admixture analysis performed with the program NGSadmix (Skotte et al. 2013). *a*) Bar plot representing individual admixture proportions assuming *K* = 2, which is the most likely number of clusters in the dataset according to the Evanno method based on 10 replicates per *K* value tested (from 1 to 5). In the bar plot, each horizontal bar corresponds to an individual and individuals from the same location are grouped in the same block. The size of each color division per individual bar represents the proportion of an individual’s genome that likely originated from the ancestral populations (*K*) identified, or clusters. For *K* = 2, the Atlantic cluster is indicated in orange, and the Pacific cluster in blue. *b*) Plot representing the Delta K value for K from 2 to 4 calculated with CLUMPAK (Kopelman et al. 2015).

**Supplementary Figure 5.** **Atlantic mitochondrial introgression in Balsfjord herring.** *a)* Pairwise genetic distance among 79 individuals based on 1,382 SNPs in the mtDNA. The genetic distance corresponds to the effective number of nucleotide differences (counts) between pairs. Low to high genetic distance is represented in gray shades from white to black, respectively. Individuals from the various geographic groups are denoted with a colored bar on the left, in orange for Atlantic-Baltic, black for Balsfjord, green for Subarctic, and blue for Pacific. The arrow on the right points to the Balsfjord individual with an Atlantic mtDNA type. *b)* Distance-based unrooted neighbor-joining tree based on individual mtDNA sequences. Besides the broad distinction between the Atlantic and the Pacific herring species, the Pacific herring is subdivided into a NE Pacific (Vancouver) lineage and NW Pacific + European lineage. Three of the four Balsfjord sequences cluster with NW Pacific sequences, and the fourth clusters with Atlantic. The four Balsfjord sequences are denoted with arrows. Sample label colors indicate region of origin. *c)* Heatmap plot showing the pool allele frequency (AF) of 213 SNPs in 15 populations. Cell colors indicate the frequency of the Atlantic mtDNA allele. Same as in (*b*), pool samples from the various geographic groups are denoted with a color bar on the left. Notice that in the Balsfjord pool the Atlantic allele frequency is around 27%.

**Supplementary Figure 6. Histogram of distance ratios for all 20 kb windows and all haploid Balsfjord genomes.** *d_p_* and *d_a_* are the nucleotide distances from a given Balsfjord sample (B) to the closest sample in the "Sea of Japan" (P) and "Atlantic" (A) groups, respectively. The dashed vertical line indicates the genomic average, while the solid vertical lines indicate the thresholds towards ether type of HSR, These are set at one eight and eight times the mean ratio, respectively (see methods for details).

**Supplementary Figure 7. Genomic regions showing introgression in Balsfjord herring.** *a)* Locations of introgressed regions (HSRs) and their origin, based on 20 kb windows. The dashed red lines represent the cut-offs for definition of Pacific and Atlantic HSRs, respectively. *b)* Summary of the location and frequency of Atlantic HSRs in the eight haplotypes from four individually sequenced Balsfjord herring. The 13 regions in which all eight haplotypes were deduced to originate from Atlantic herring are highlighted (boxes). Unassigned scaffolds are collected under the “Un.” label.

**Supplementary Figure 8. M-values (i.e., log2(Observed/Expected) for different counts of HSRs among the 8 haploid genomes from Balsfjord individuals.** Black and Red dots are for regions of Atlantic and Pacific origin, respectively.

**Supplementary Figure 9.** **Pool-seq frequencies at 8-fold Atlantic HSRs.** Heatmaps showing reference allele frequencies (from Atlantic herring) for pooled samples in all 13 genomic regions where all Balsfjord individual haplotypes carry an HSR of Atlantic origin.

**Supplementary Figure 10.** **Pool-seq frequencies at 8-fold Pacific HSRs.** Heatmaps showing reference allele frequencies (from Atlantic herring) for pooled samples in all 23 genomic regions where all Balsfjord individual haplotypes carry an HSR of Pacific origin.

**Supplementary Figure 11: Hierarchical clusterings of haplotypes in 8-fold Atlantic HSRs.** The dendrograms are based on Hamming distances between haplotypes in the indicated regions. One 20 kb window has been selected from each recurring HSR.

**Supplementary Figure 12.** **Inter-species *F*_ST_ and number of HSRs in Balsfjord samples (of Pacific and Atlantic origin) across all chromosomes.**

**Supplementary Figure 13. Diversity summary statistics of Atlantic and Pacific herring in regions with 8x-recurring HSRs detected in Balsfjord populations.** *a)* Nucleotide diversity (𝜋) in Atlantic and Baltic samples of Atlantic herring. b*)* Nucleotide diversity (𝜋) in European and Northwest Pacific samples of Pacific herring. c*) d_xy_* between those Atlantic herring and Pacific herring samples. The labels on the x-axis indicate the type of recurring HSR.

**Supplementary Figure 14. Recombination rate in regions of 8x-recurring HSRs.** Recombination rate across introgressed and background regions, respectively. The labels on the x-axis indicate the type of recurring HSR.

Supplementary Table 1**.** List of samples used in this study.

Supplementary Table 2**.** Nucleotide diversity (*π*) in sample groups, of Atlantic and Pacific herring, based on individual samples.

Supplementary Table 3**.** Ancestry coefficients of 79 individuals estimated with the program sNMF for K = 2, Q1 is the proportion of Pacific ancestry and Q2 is Atlantic ancestry. Balsfjord samples are indicated by italics.

Supplementary Table 4**.** All detected HSRs in the four individual Balsfjord specimens.

Supplementary Table 5**.** Recurring HSRs; regions with either Atlantic or Pacific HSRs, respectively, observed in all eight haploid genomes from Balsfjord.
