# Supplementary material for: A Long-Standing Hybrid Population Between Pacific and Atlantic Herring in a Subarctic Fjord of Norway
Source: Genome Biol Evol. 2023 Apr 30;15(5):evad069. doi: 10.1093/gbe/evad069 (PMC10182735; doi:10.1093/gbe/evad069)
Supplement: evad069_Supplementary_Data [file evad069_supplementary_data.zip › Supplementary_Figure_1.pdf]

Supplementary Figure 1. F_{ST}, dxy and π for individual chromosomes based on the Balsfjord samples, compared with Atlantic spring-spawning samples. Black dots represent 5 kb windows, and the red line is a rolling average. In the π-panels, the blue line shows Balsfjord π, while the red one shows Atlantic π.

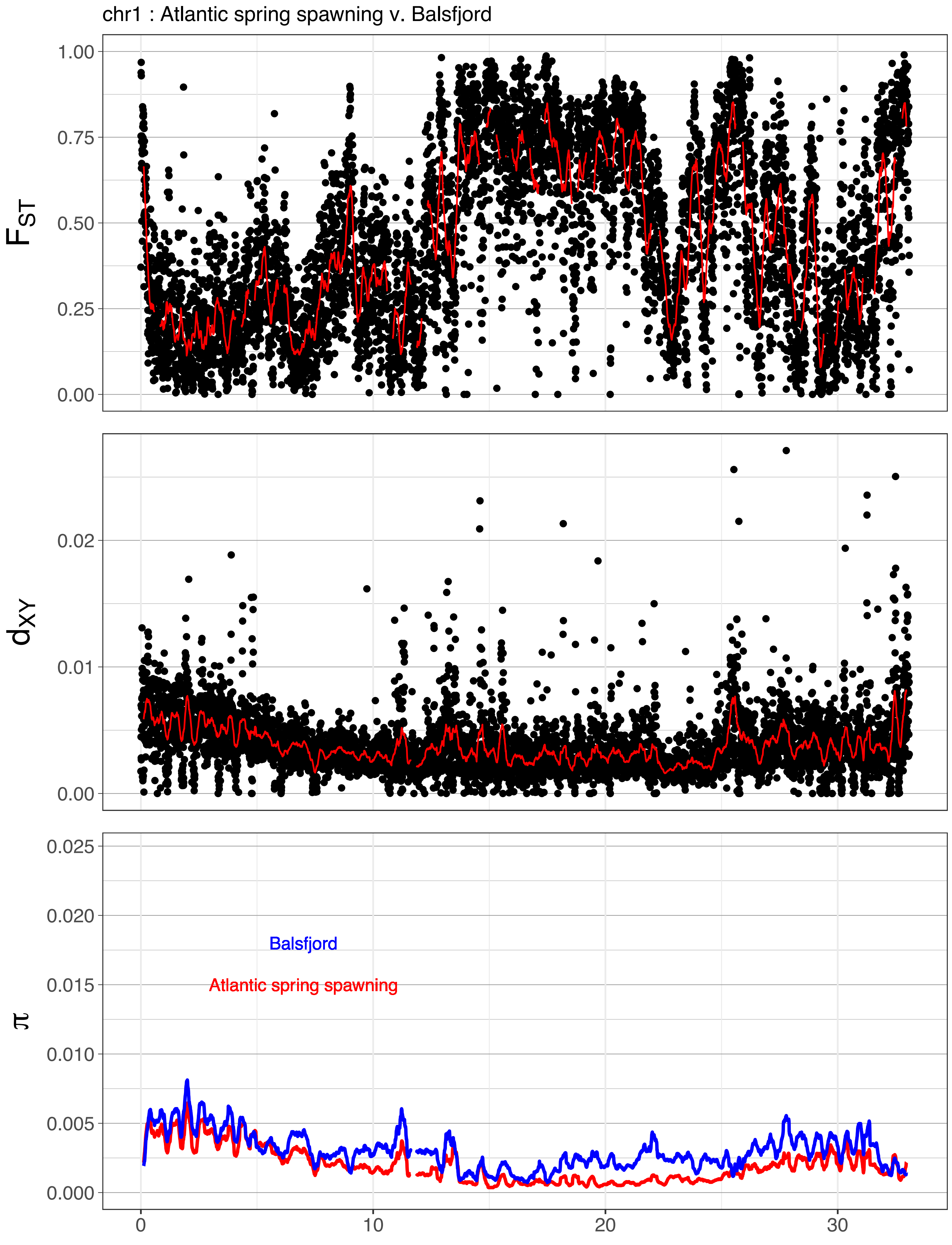
chr1 : Atlantic spring spawning v. Balsfjord
F_{ST}
1.00
0.75
0.50
0.25
0.00
d_{XY}
0.02
0.01
0.00
π
0.025
0.020
0.015
0.010
0.005
0.000
Balsfjord
Atlantic spring spawning
0
10
20
30

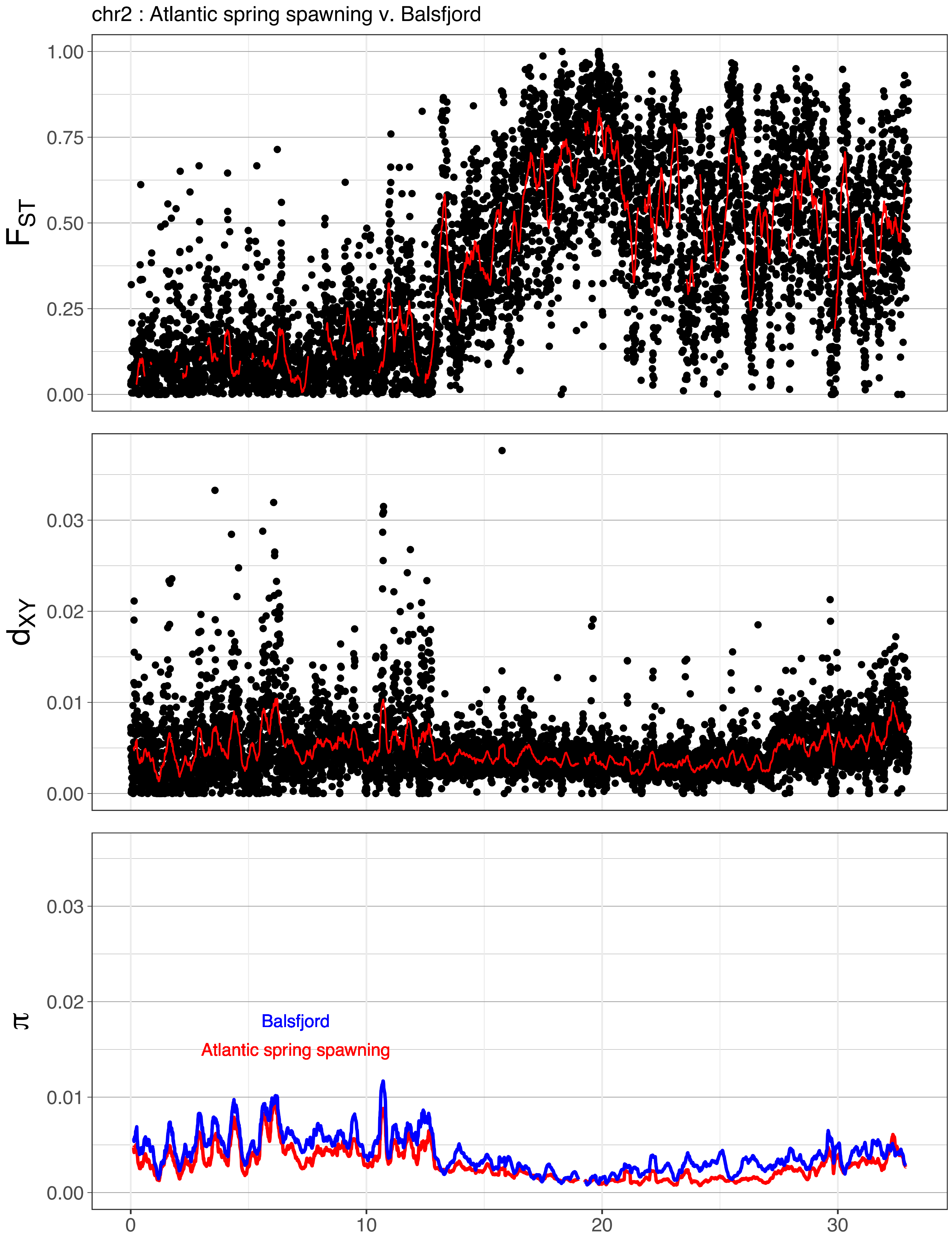

chr2 : Atlantic spring spawning v. Balsfjord
F_ST
1.00
0.75
0.50
0.25
0.00
d_XY
0.03
0.02
0.01
0.00
π
0.03
0.02
0.01
0.00
Balsfjord
Atlantic spring spawning
0
10
20
30

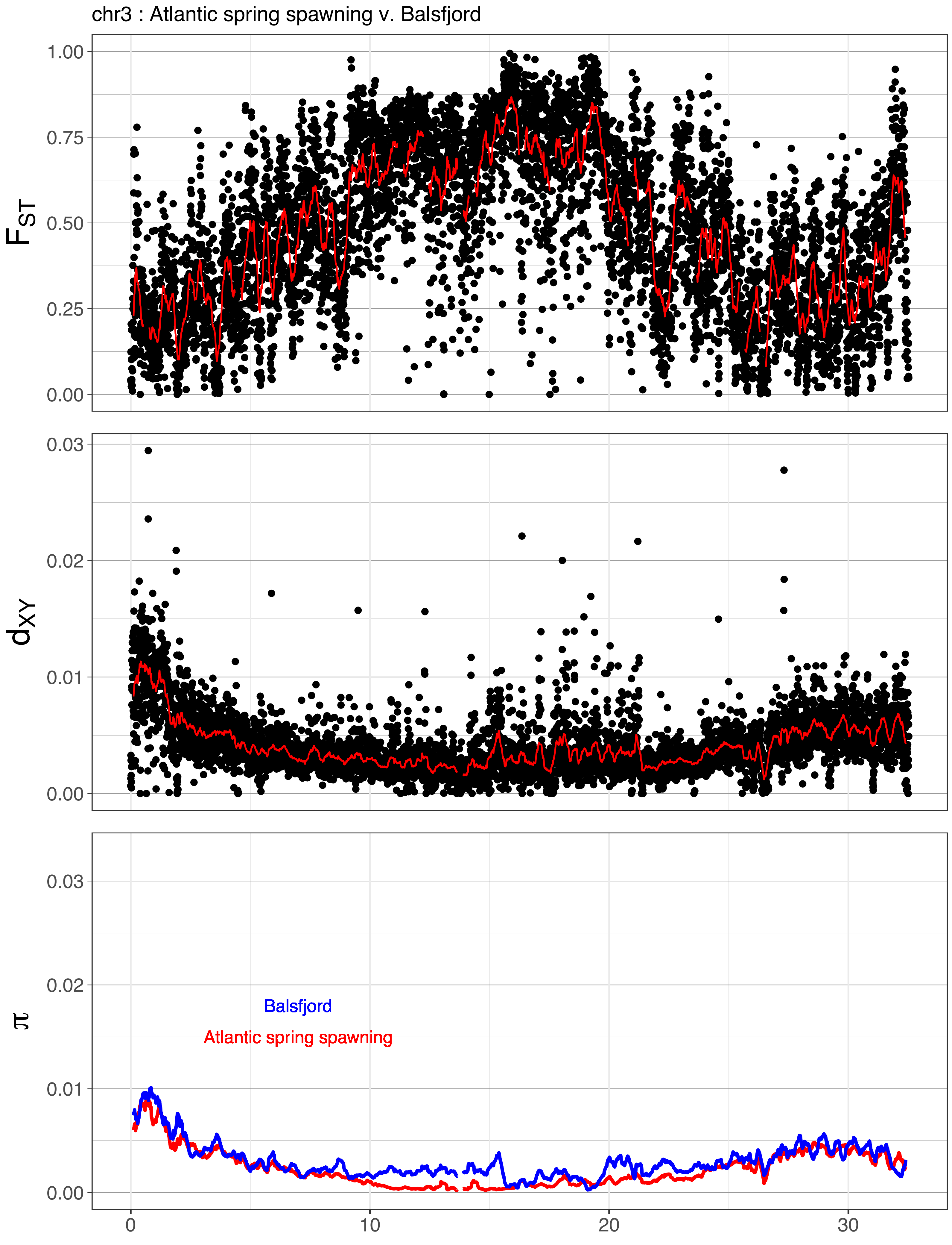
chr3 : Atlantic spring spawning v. Balsfjord
F_{ST}
d_{XY}
π
Balsfjord
Atlantic spring spawning
1.00
0.75
0.50
0.25
0.00
0.03
0.02
0.01
0.00
0
10
20
30

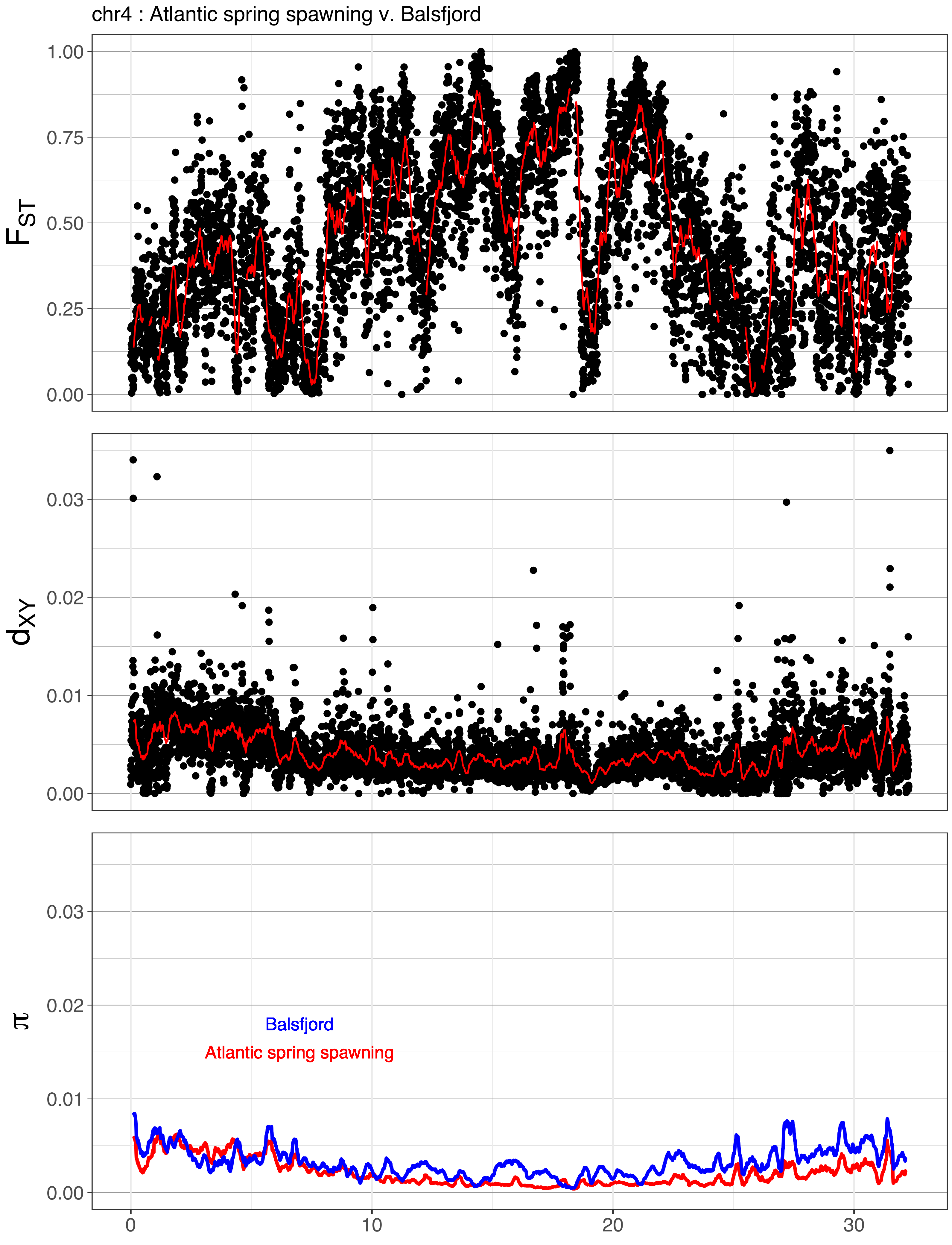
chr4 : Atlantic spring spawning v. Balsfjord
F_ST
1.00
0.75
0.50
0.25
0.00
d_XY
0.03
0.02
0.01
0.00
π
0.03
0.02
0.01
0.00
Balsfjord
Atlantic spring spawning
0
10
20
30

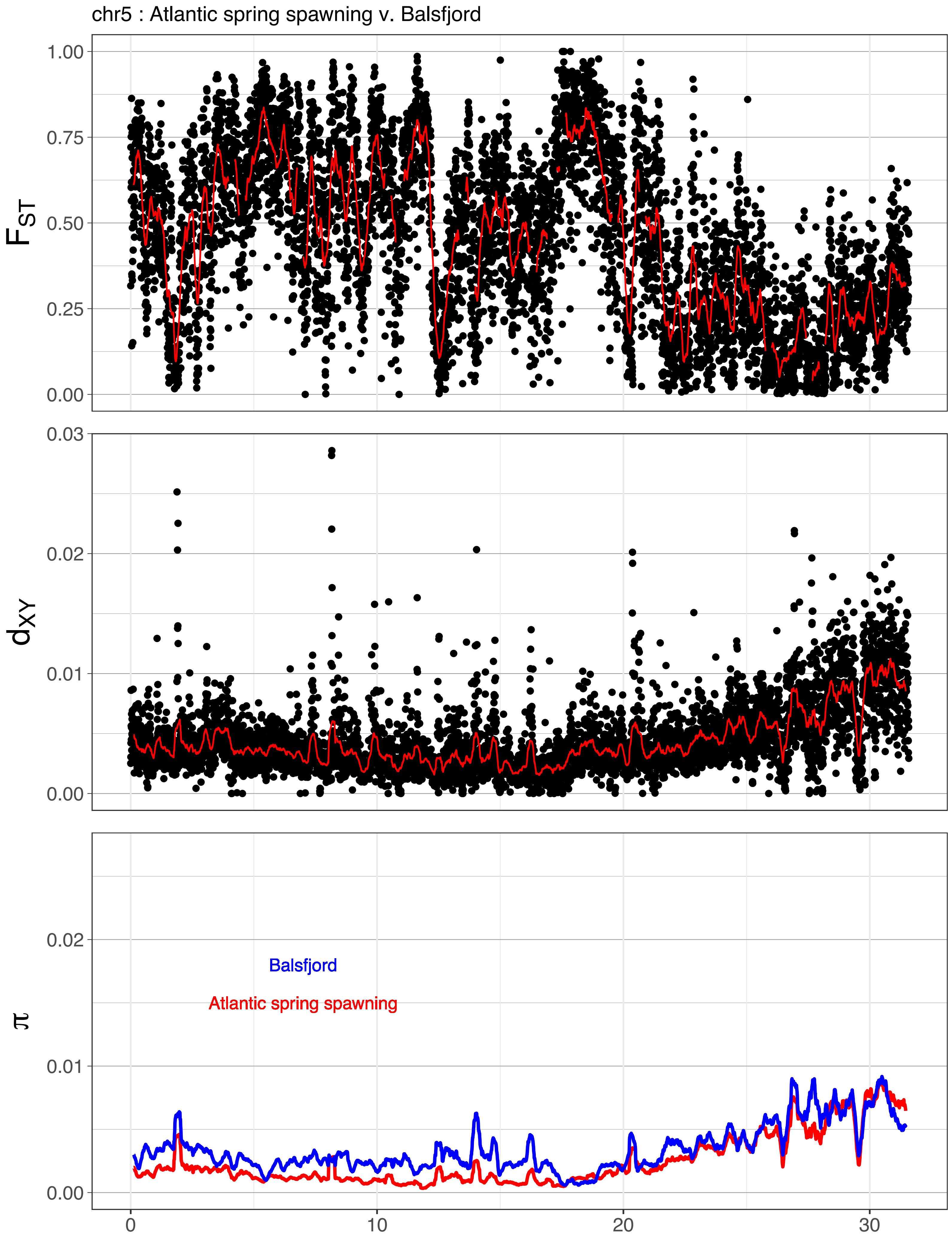

chr5 : Atlantic spring spawning v. Balsfjord
F_ST
1.00
0.75
0.50
0.25
0.00
d_XY
0.03
0.02
0.01
0.00
π
0.02
0.01
0.00
Balsfjord
Atlantic spring spawning
0
10
20
30

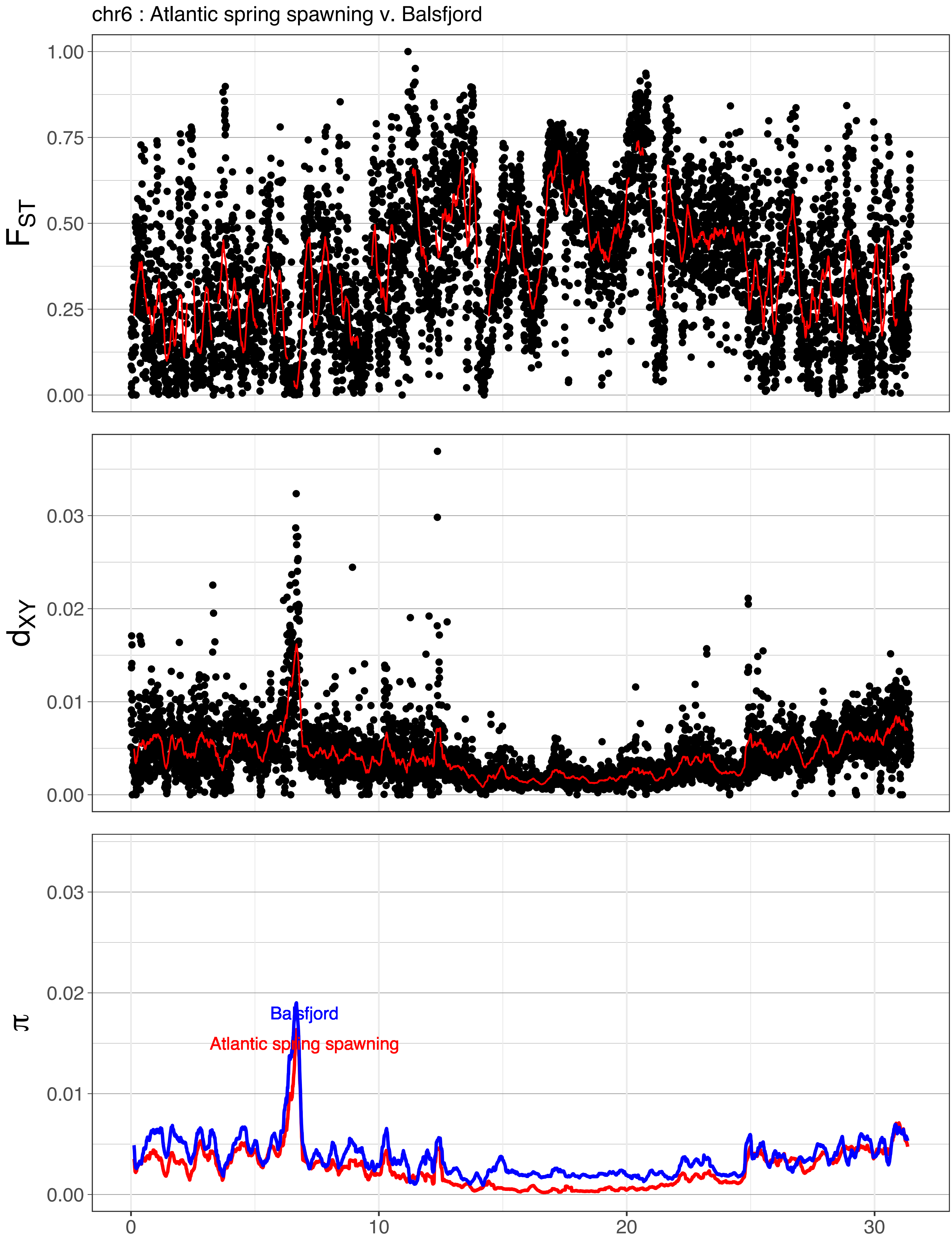
chr6 : Atlantic spring spawning v. Balsfjord
F_ST
d_XY
π
Balsfjord
Atlantic spring spawning
1.00
0.75
0.50
0.25
0.00
0.03
0.02
0.01
0.00
0.03
0.02
0.01
0.00
0
10
20
30

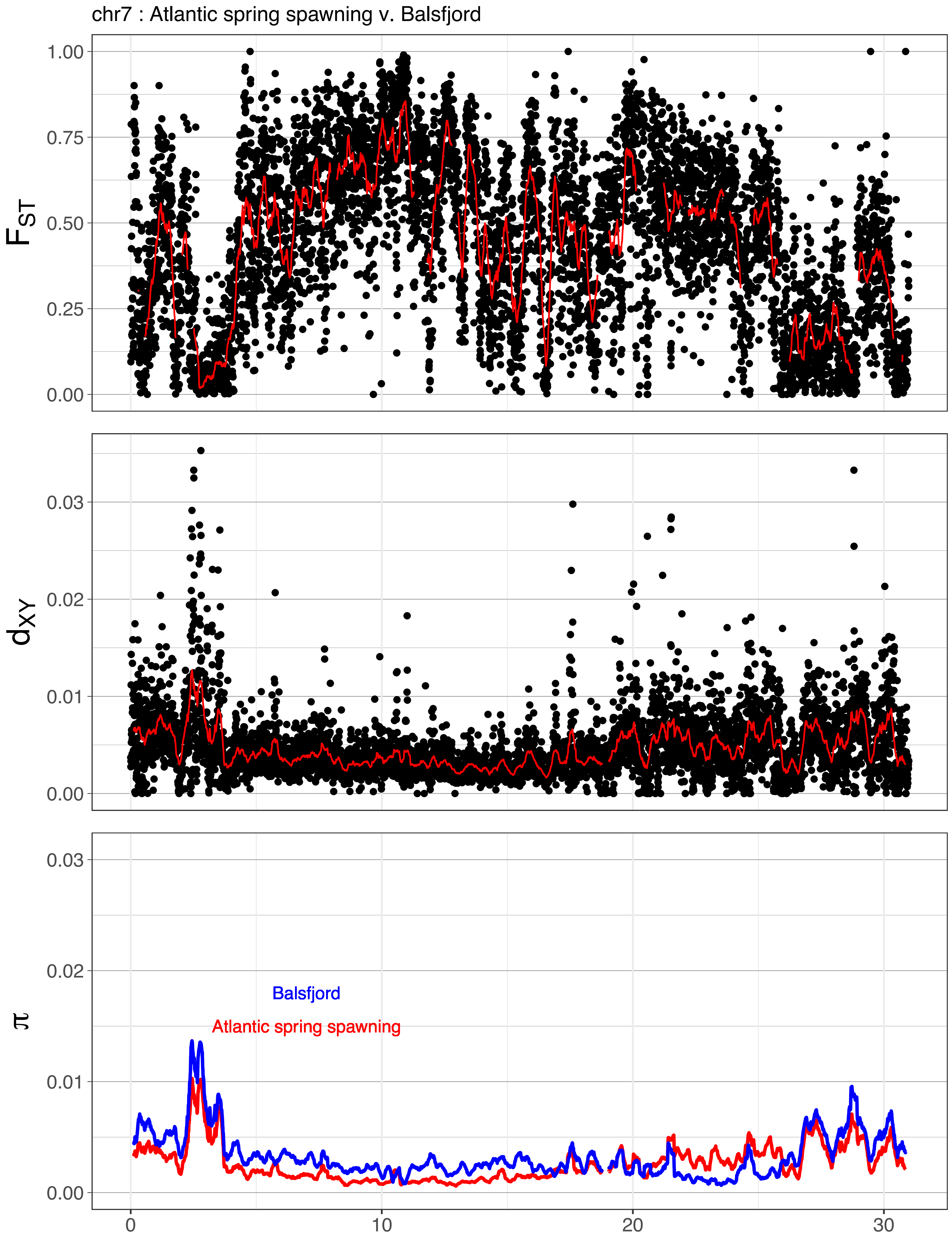
chr7 : Atlantic spring spawning v. Balsfjord
F_{ST}
1.00
0.75
0.50
0.25
0.00
d_{XY}
0.03
0.02
0.01
0.00
π
Balsfjord
Atlantic spring spawning
0
10
20
30

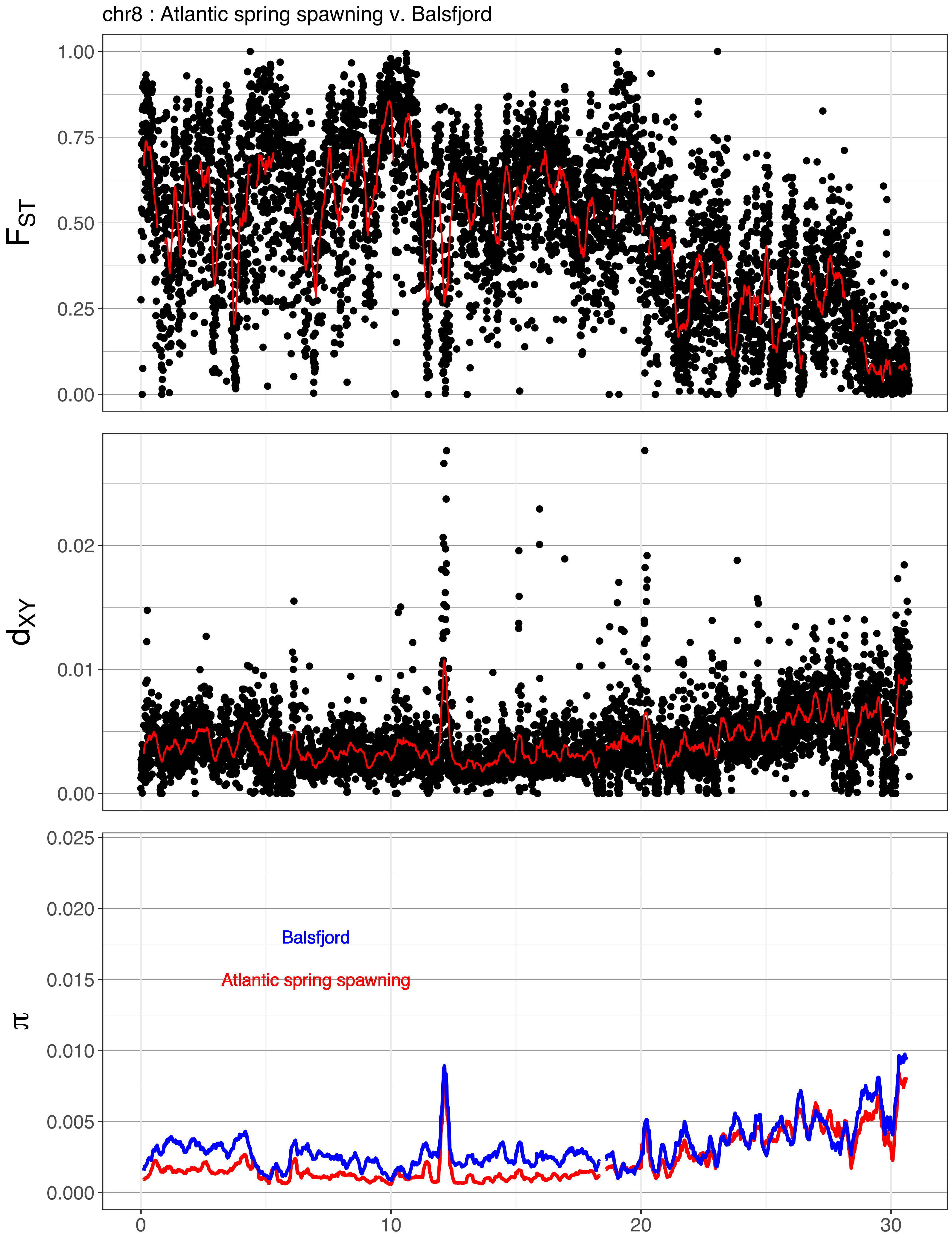

chr8 : Atlantic spring spawning v. Balsfjord
F_{ST}
1.00
0.75
0.50
0.25
0.00
d_{XY}
0.02
0.01
0.00
π
0.025
0.020
0.015
0.010
0.005
0.000
Balsfjord
Atlantic spring spawning
0
10
20
30

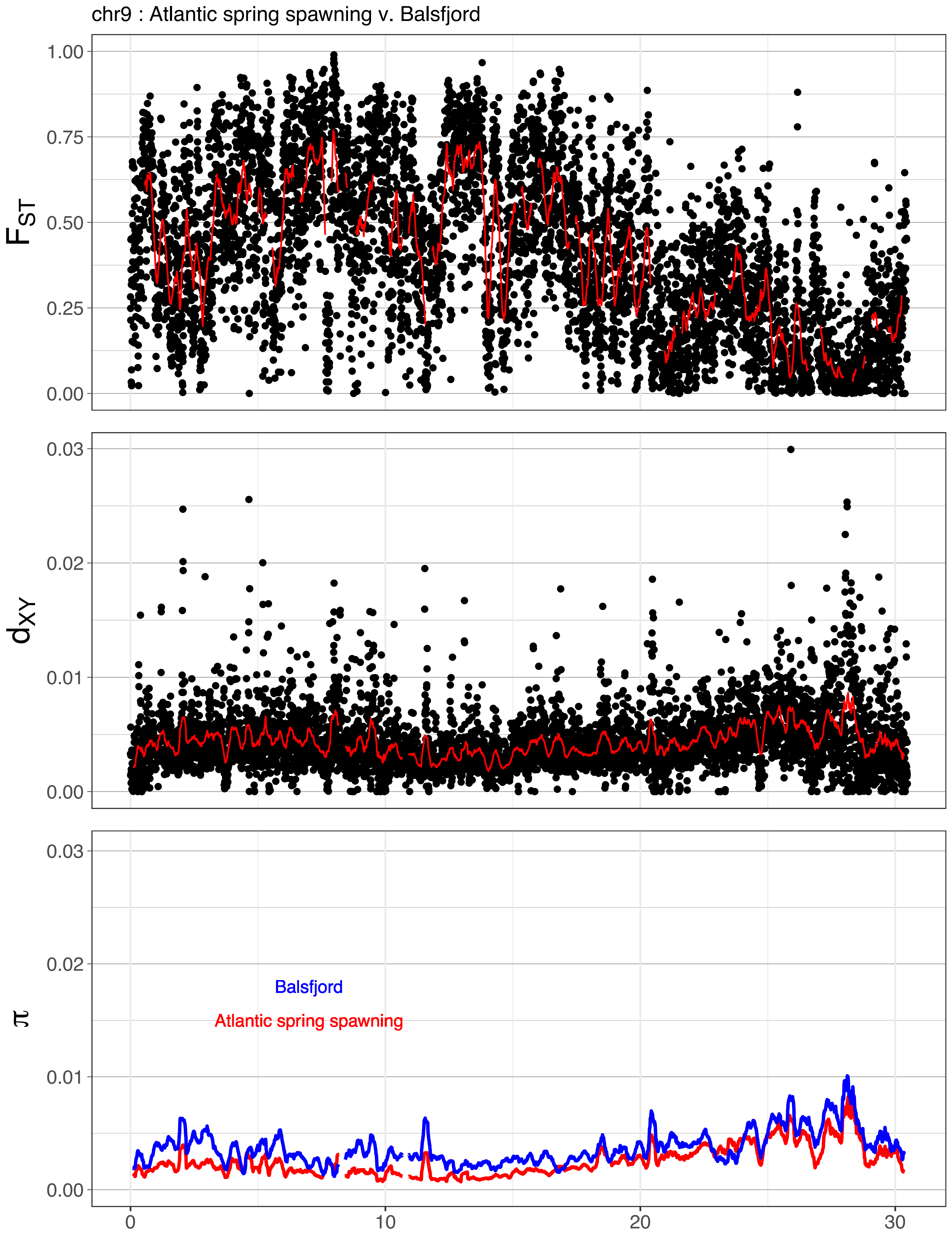
chr9 : Atlantic spring spawning v. Balsfjord
F_{ST}
1.00
0.75
0.50
0.25
0.00
d_{XY}
0.03
0.02
0.01
0.00
π
0.03
0.02
0.01
0.00
Balsfjord
Atlantic spring spawning
0
10
20
30

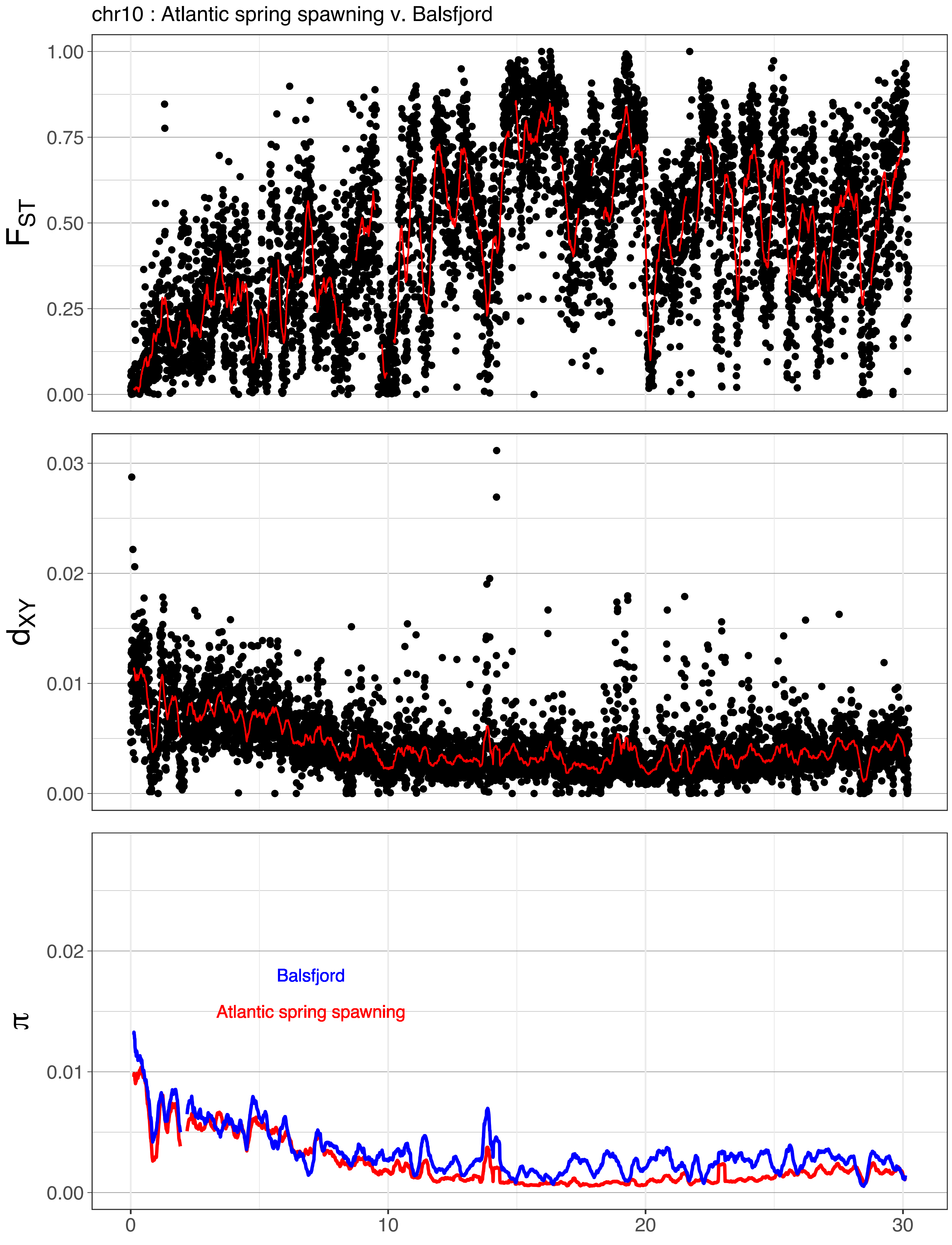

chr10 : Atlantic spring spawning v. Balsfjord
FST
1.00
0.75
0.50
0.25
0.00
dXY
0.03
0.02
0.01
0.00
π
0.02
0.01
0.00
Balsfjord
Atlantic spring spawning
0
10
20
30

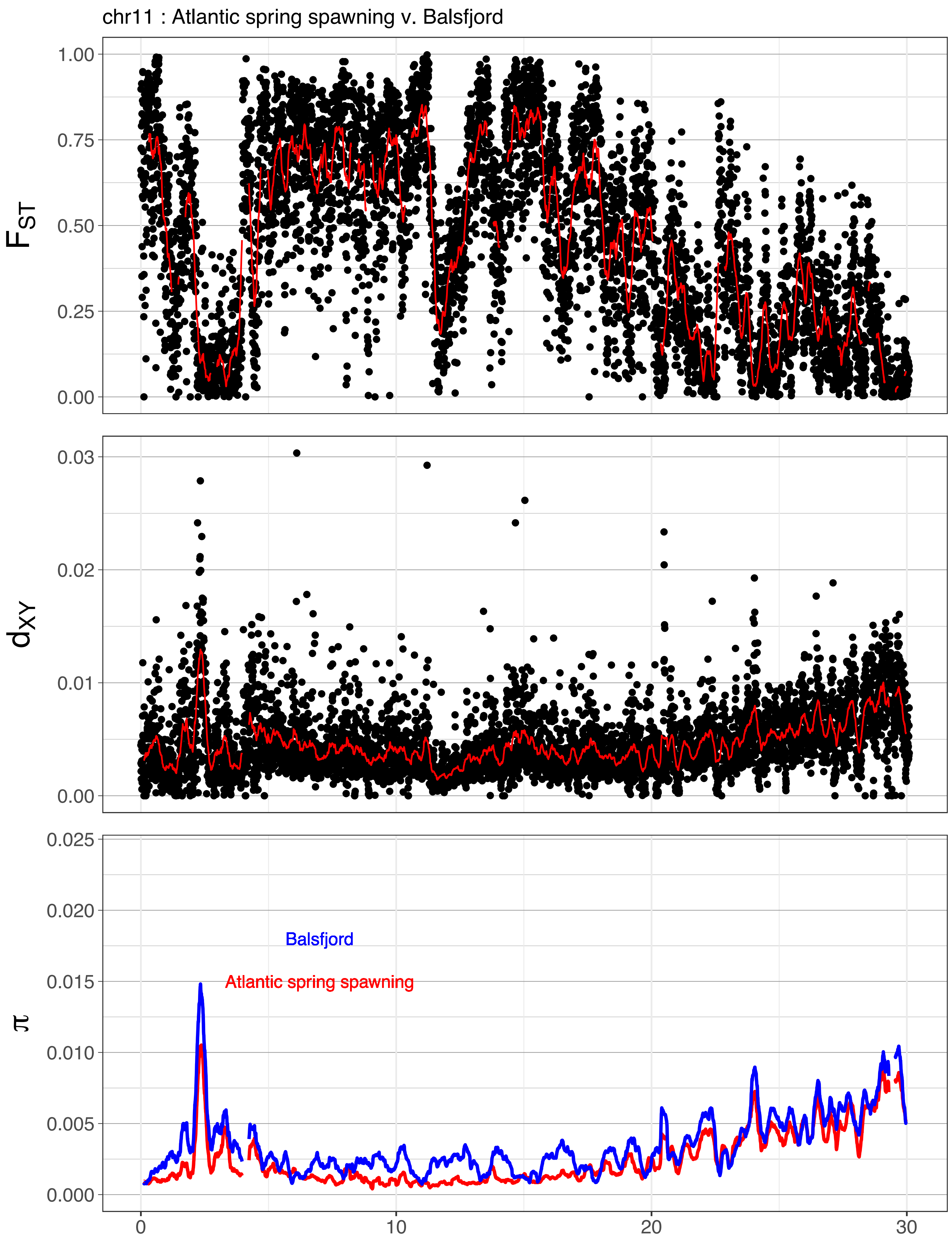

chr11 : Atlantic spring spawning v. Balsfjord
F_{ST}
1.00
0.75
0.50
0.25
0.00
d_{XY}
0.03
0.02
0.01
0.00
π
0.025
0.020
0.015
0.010
0.005
0.000
Balsfjord
Atlantic spring spawning
0
10
20
30

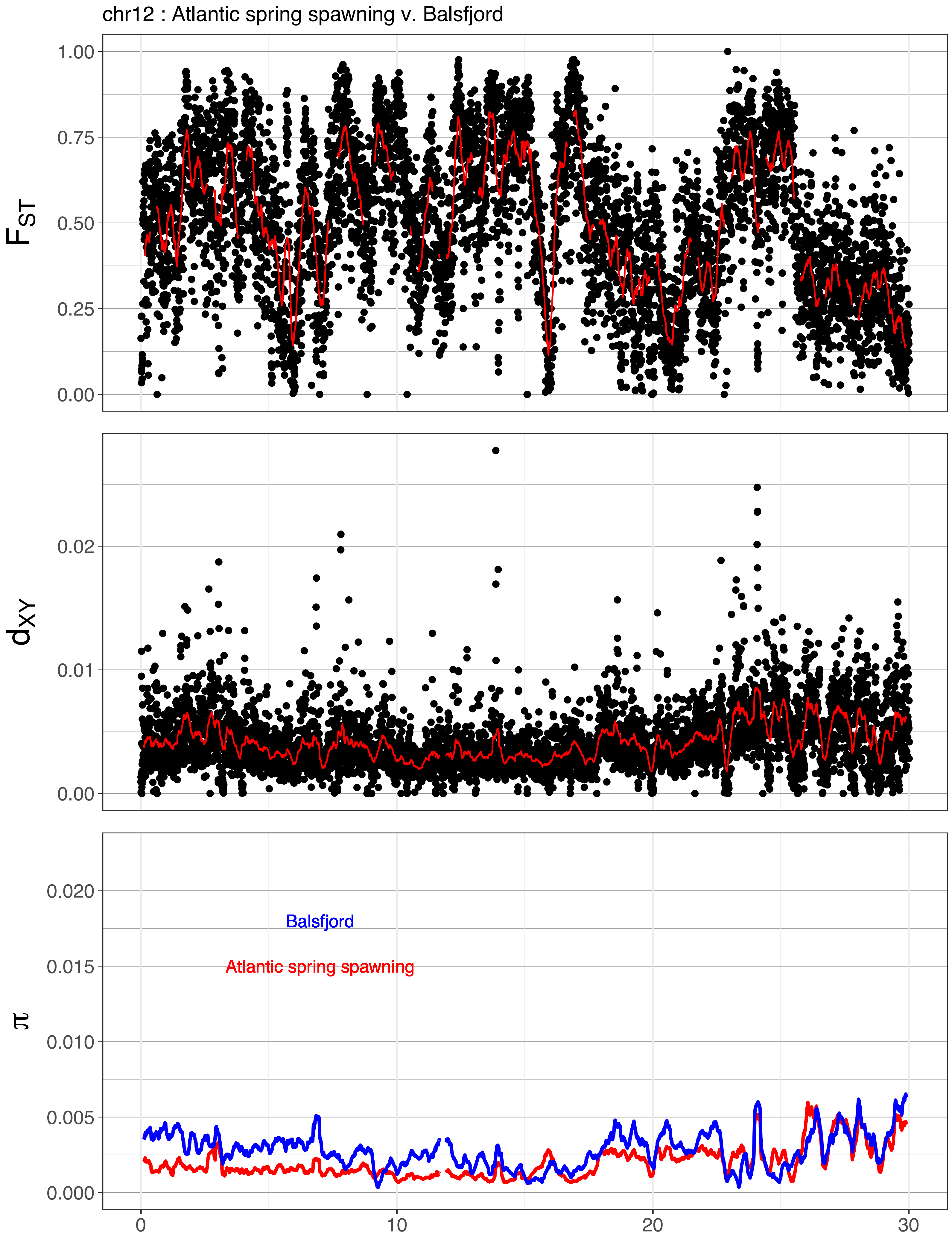

chr12 : Atlantic spring spawning v. Balsfjord
F_{ST}
1.00
0.75
0.50
0.25
0.00
d_{XY}
0.02
0.01
0.00
π
0.020
0.015
0.010
0.005
0.000
Balsfjord
Atlantic spring spawning
0
10
20
30

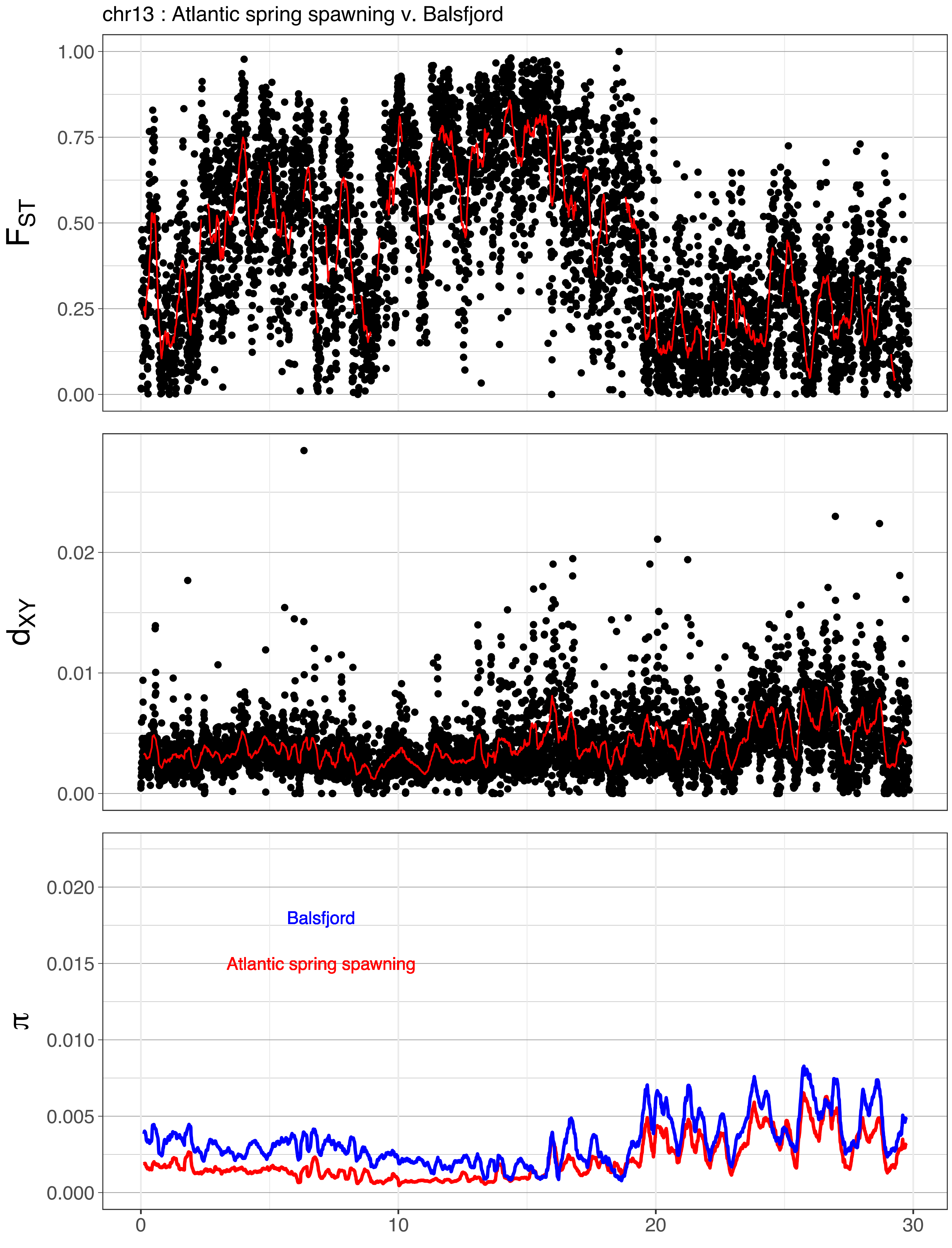

chr13 : Atlantic spring spawning v. Balsfjord
F_{ST}
1.00
0.75
0.50
0.25
0.00
d_{XY}
0.02
0.01
0.00
π
0.020
0.015
0.010
0.005
0.000
Balsfjord
Atlantic spring spawning
0
10
20
30

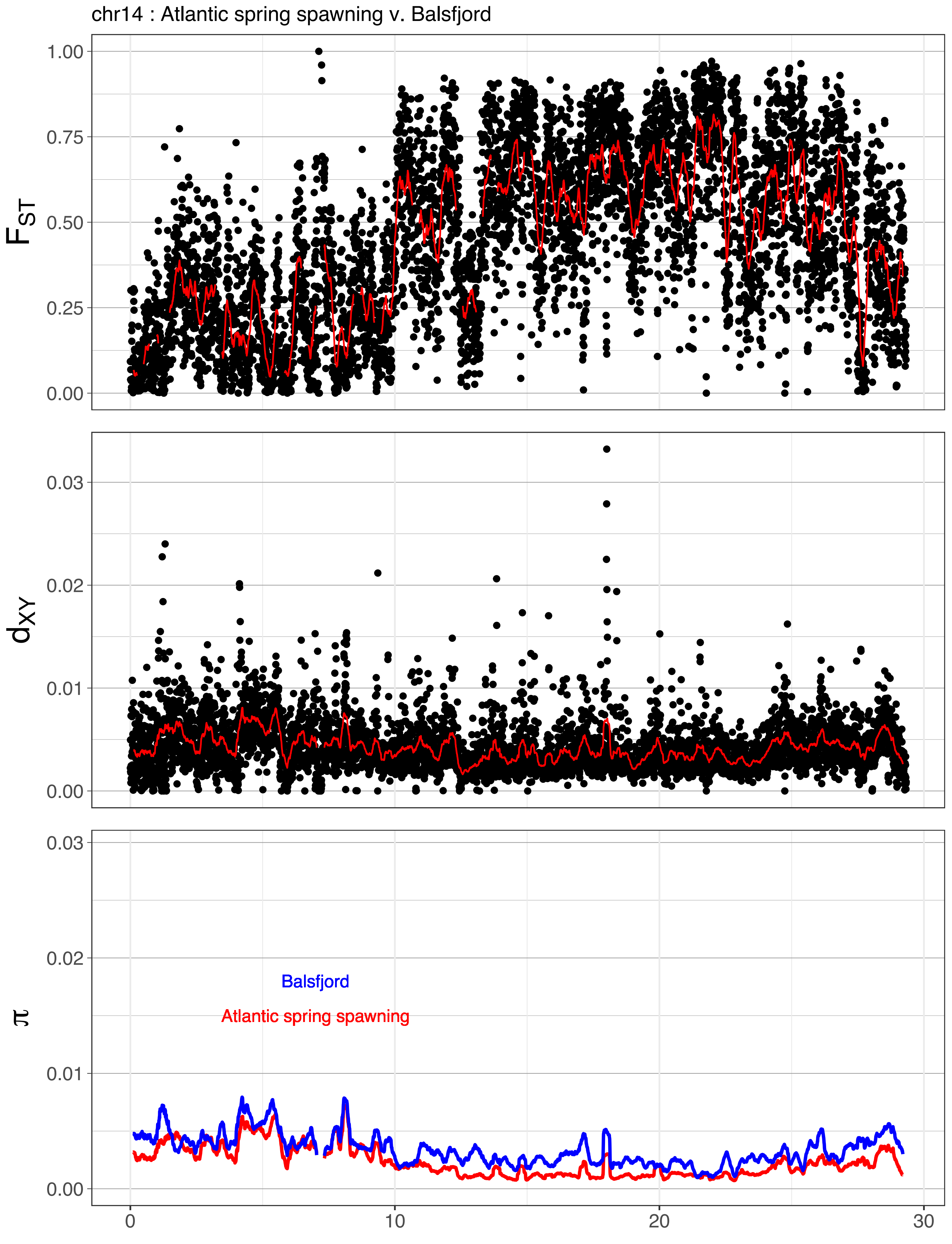
chr14 : Atlantic spring spawning v. Balsfjord
F_{ST}
1.00
0.75
0.50
0.25
0.00
d_{XY}
0.03
0.02
0.01
0.00
π
0.03
0.02
0.01
0.00
Balsfjord
Atlantic spring spawning
0
10
20
30

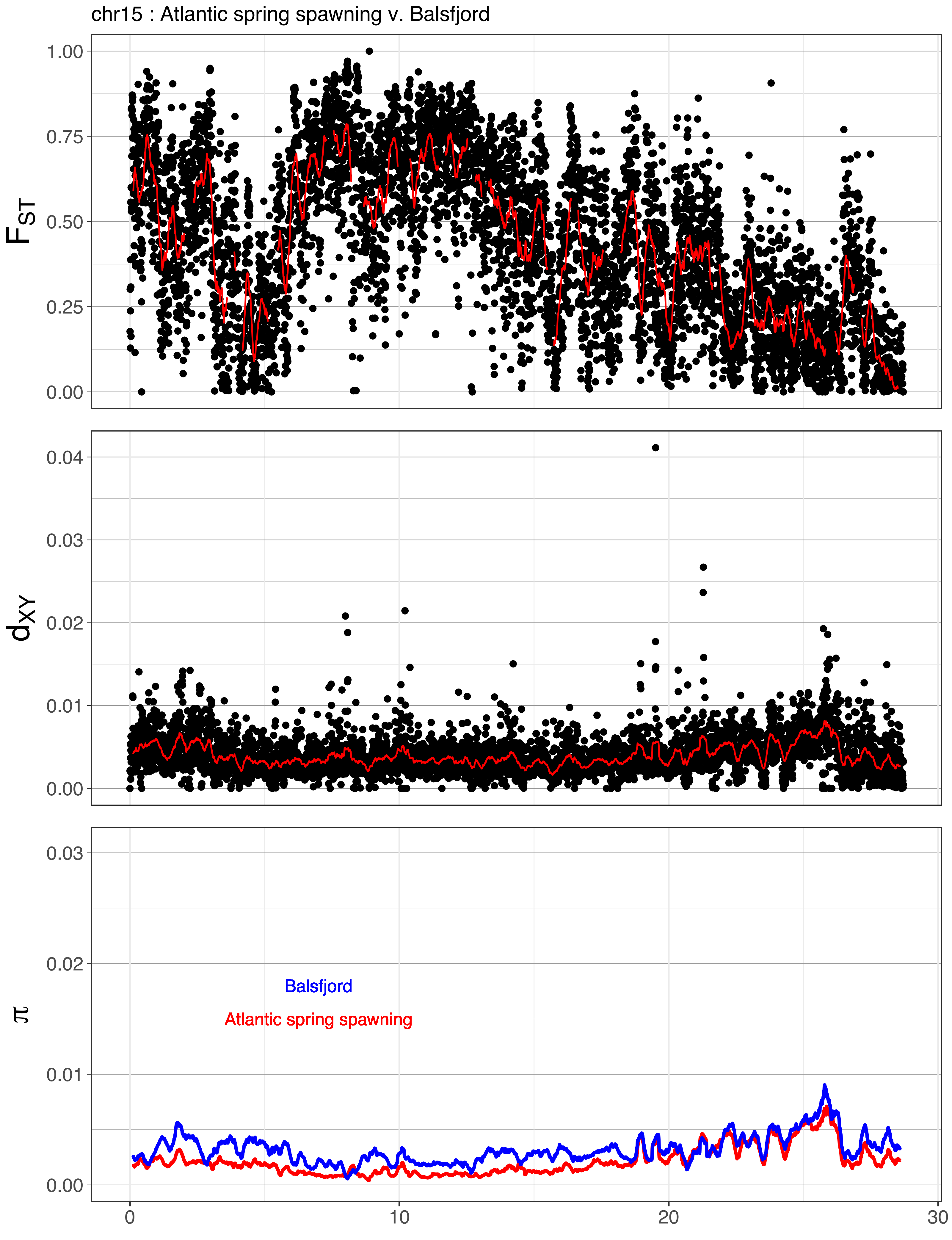
chr15 : Atlantic spring spawning v. Balsfjord
F_ST
1.00
0.75
0.50
0.25
0.00
d_XY
0.04
0.03
0.02
0.01
0.00
π
0.03
0.02
0.01
0.00
Balsfjord
Atlantic spring spawning
0
10
20
30

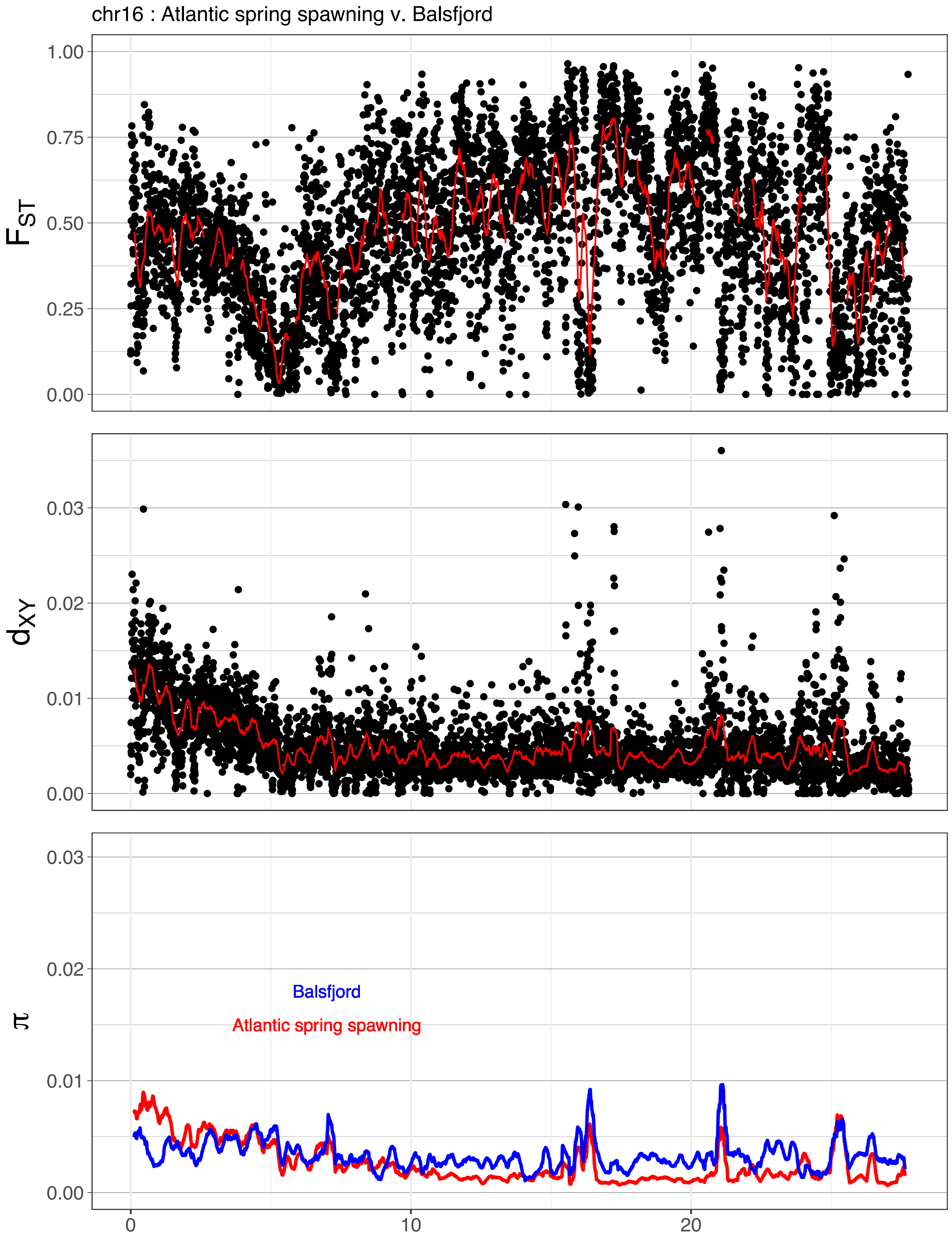
chr16 : Atlantic spring spawning v. Balsfjord
F_{ST}
1.00
0.75
0.50
0.25
0.00
d_{XY}
0.03
0.02
0.01
0.00
π
Balsfjord
Atlantic spring spawning
0.03
0.02
0.01
0.00
0
10
20

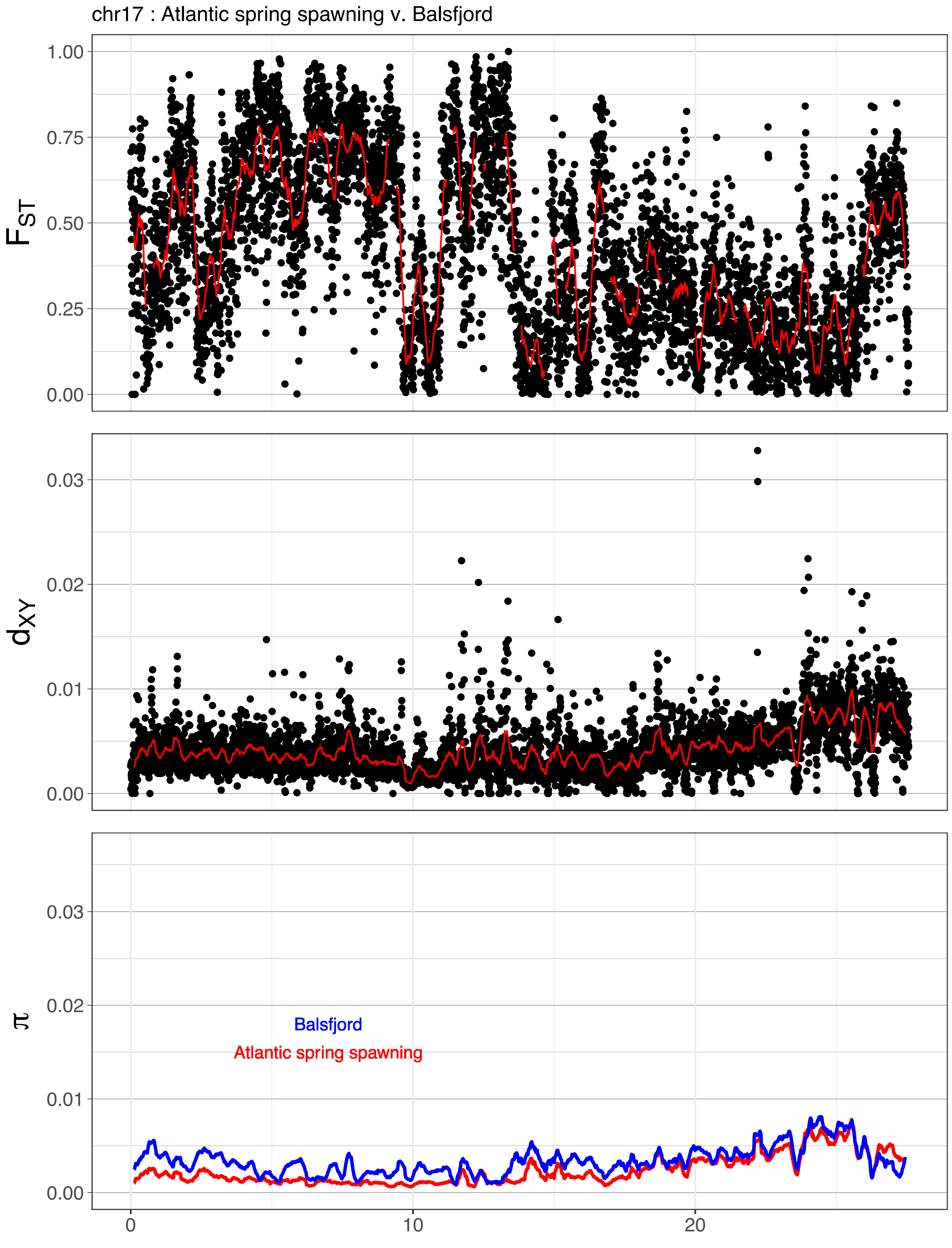

chr17 : Atlantic spring spawning v. Balsfjord
F_{ST}
1.00
0.75
0.50
0.25
0.00
d_{XY}
0.03
0.02
0.01
0.00
π
0.03
0.02
0.01
0.00
Balsfjord
Atlantic spring spawning
0
10
20

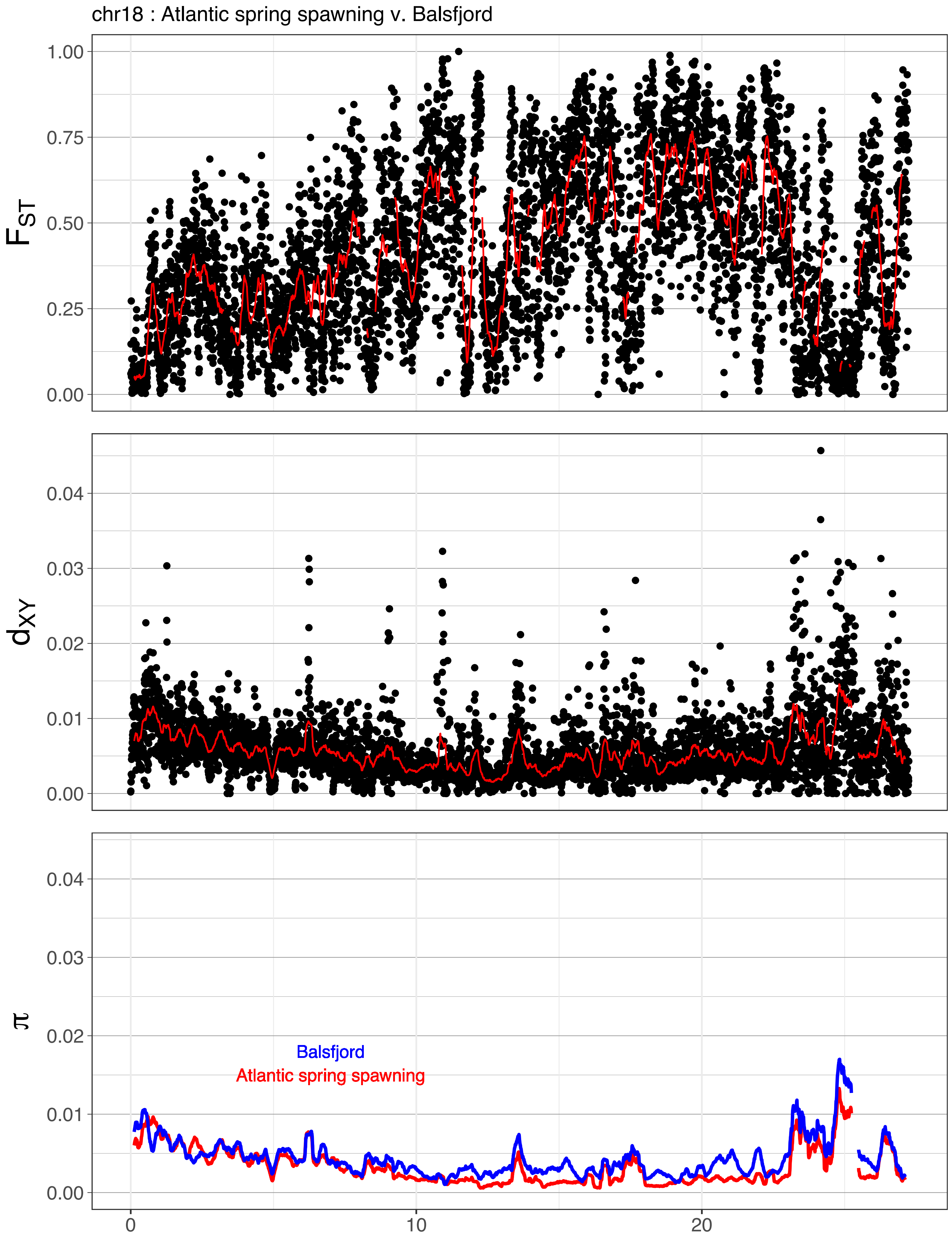
chr18 : Atlantic spring spawning v. Balsfjord
F_{ST}
1.00
0.75
0.50
0.25
0.00
d_{XY}
0.04
0.03
0.02
0.01
0.00
π
0.04
0.03
0.02
0.01
0.00
Balsfjord
Atlantic spring spawning
0
10
20

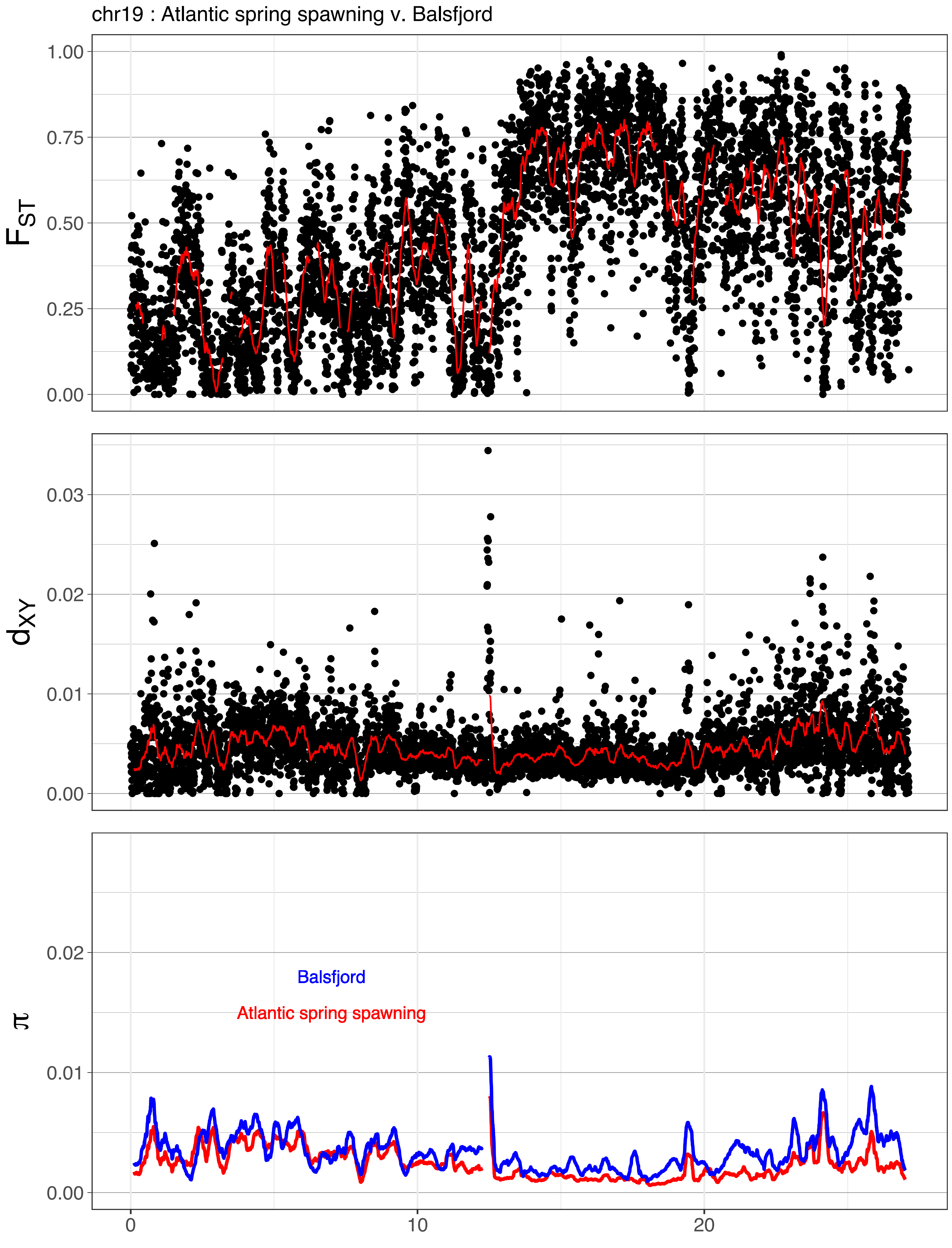

chr19 : Atlantic spring spawning v. Balsfjord
F_{ST}
1.00
0.75
0.50
0.25
0.00
d_{XY}
0.03
0.02
0.01
0.00
π
0.02
0.01
0.00
Balsfjord
Atlantic spring spawning
0
10
20

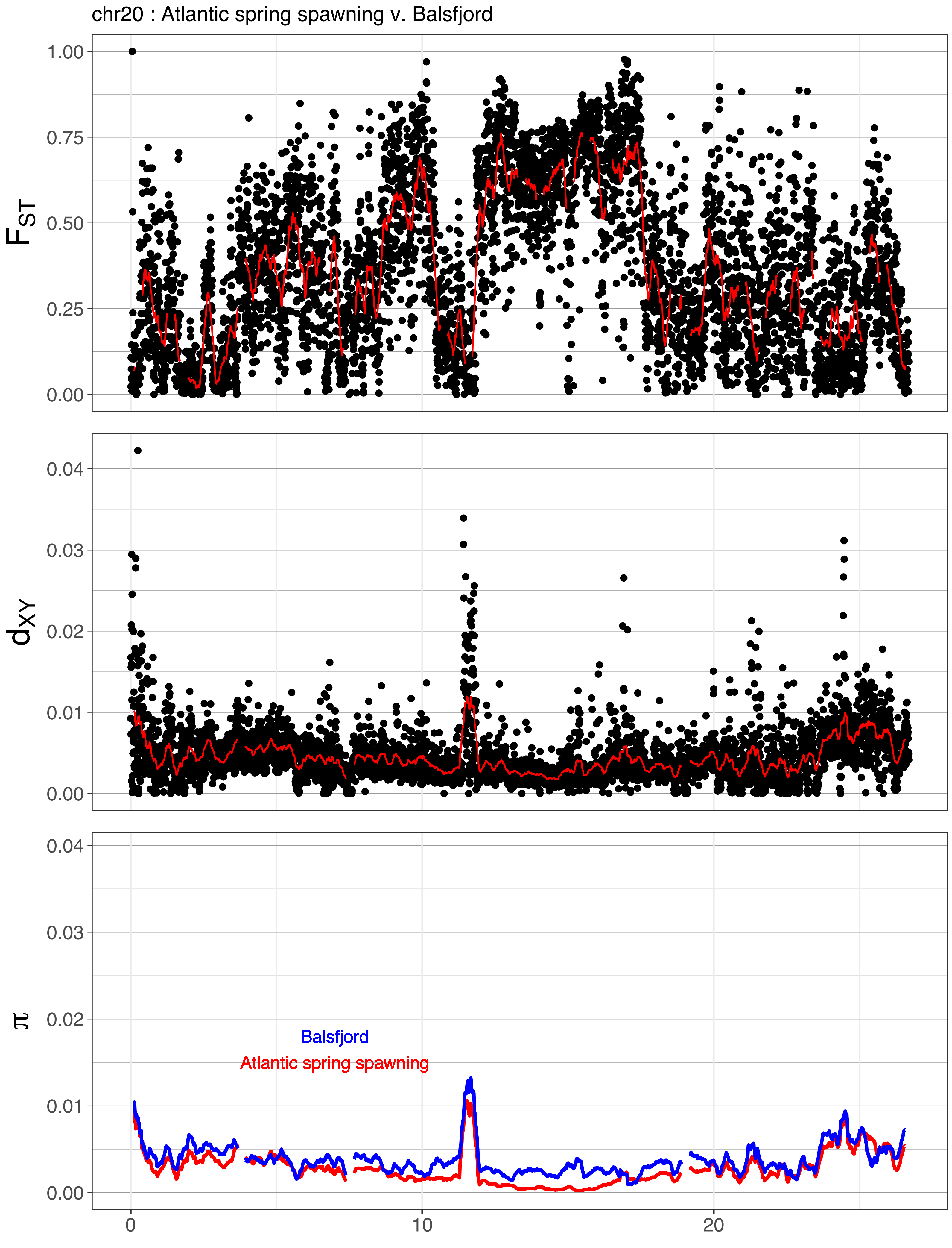

chr20 : Atlantic spring spawning v. Balsfjord
F_{ST}
1.00
0.75
0.50
0.25
0.00
d_{XY}
0.04
0.03
0.02
0.01
0.00
π
0.04
0.03
0.02
0.01
0.00
Balsfjord
Atlantic spring spawning
0
10
20

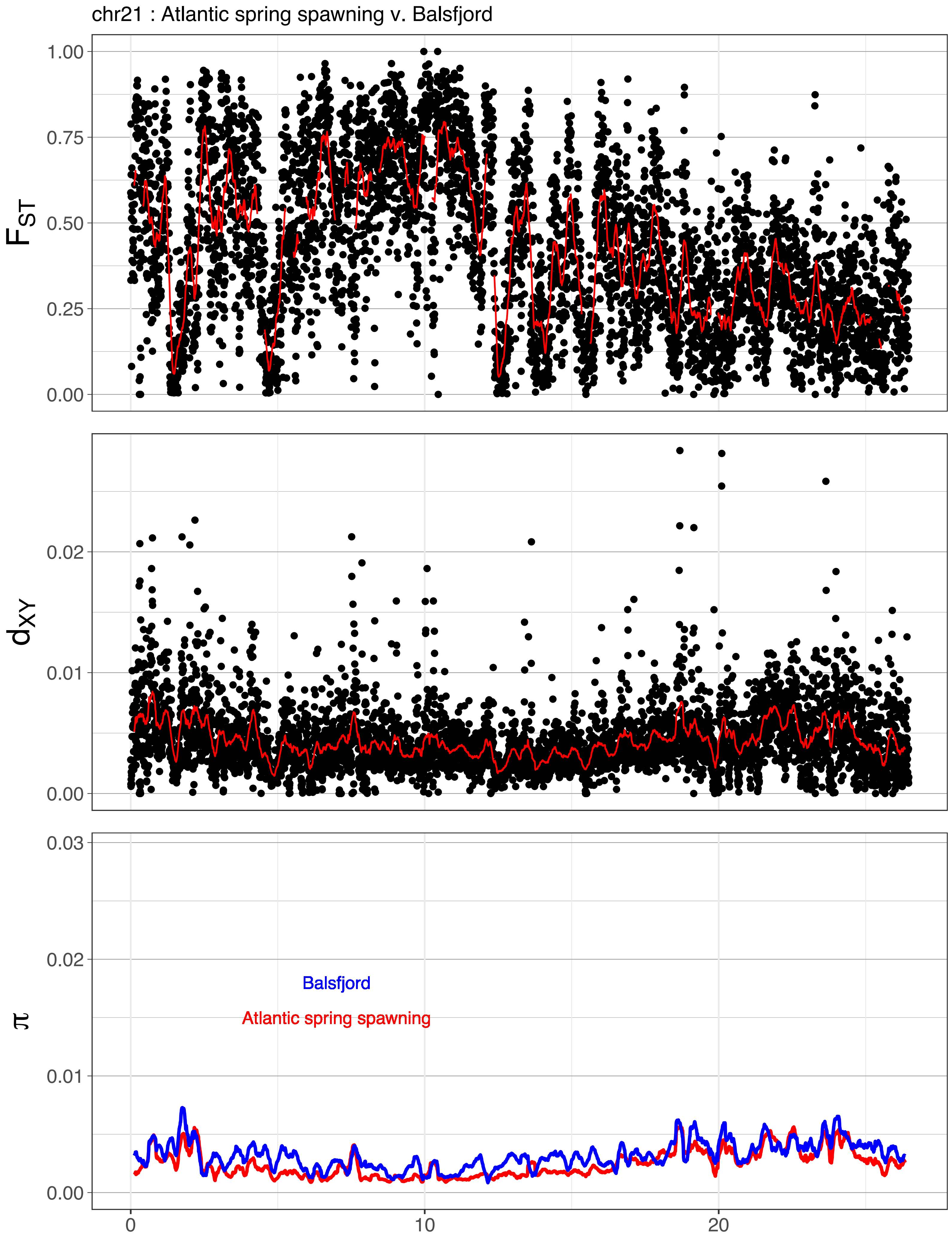
chr21 : Atlantic spring spawning v. Balsfjord
F_{ST}
1.00
0.75
0.50
0.25
0.00
d_{XY}
0.02
0.01
0.00
π
0.03
0.02
0.01
0.00
Balsfjord
Atlantic spring spawning
0
10
20

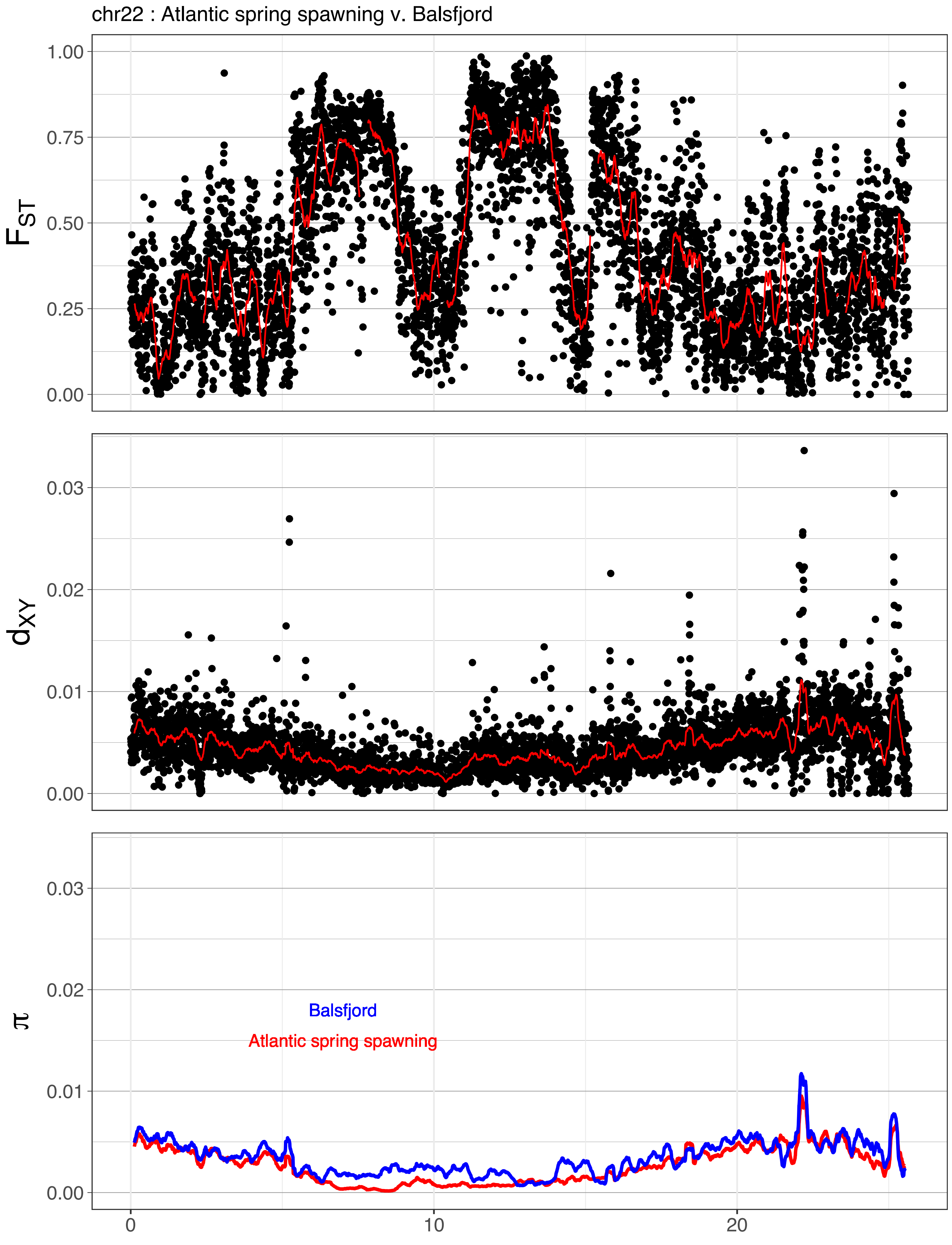
chr22 : Atlantic spring spawning v. Balsfjord
F_{ST}
1.00
0.75
0.50
0.25
0.00
d_{XY}
0.03
0.02
0.01
0.00
π
0.03
0.02
0.01
0.00
Balsfjord
Atlantic spring spawning
0
10
20

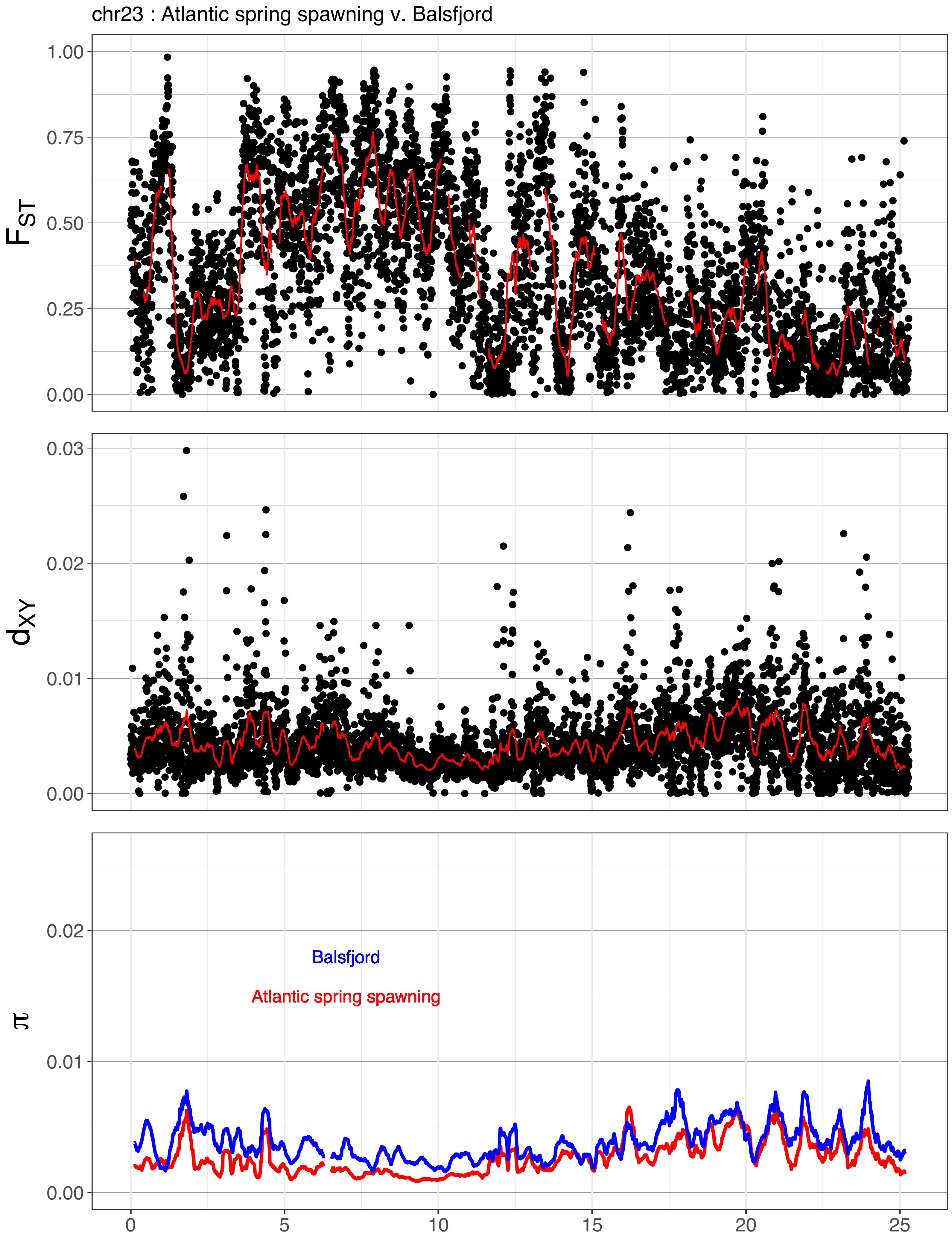

chr23 : Atlantic spring spawning v. Balsfjord
F_{ST}
1.00
0.75
0.50
0.25
0.00
d_{XY}
0.03
0.02
0.01
0.00
π
0.02
0.01
0.00
Balsfjord
Atlantic spring spawning
0
5
10
15
20
25

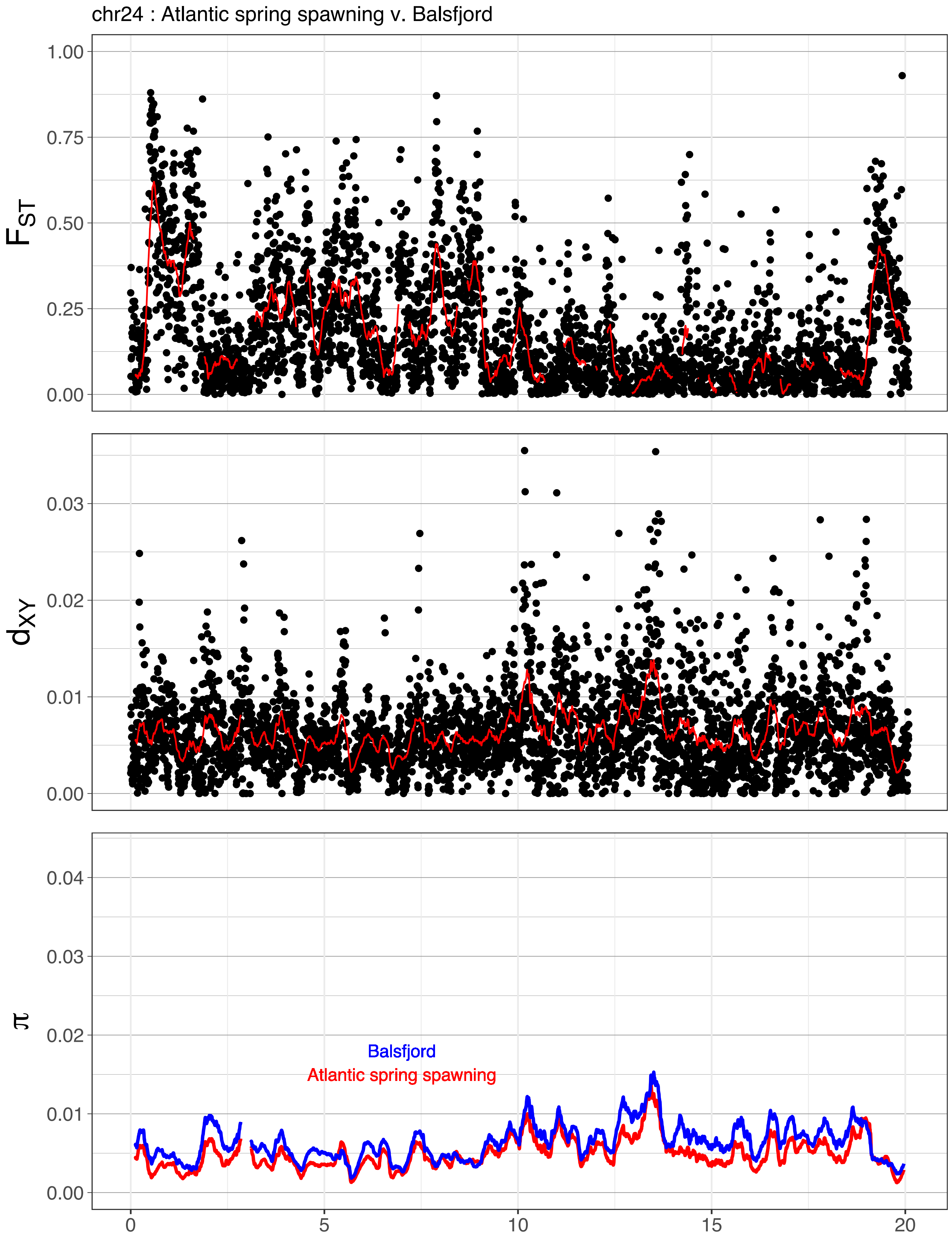

chr24 : Atlantic spring spawning v. Balsfjord
F_{ST}
1.00
0.75
0.50
0.25
0.00
d_{XY}
0.03
0.02
0.01
0.00
π
0.04
0.03
0.02
0.01
0.00
Balsfjord
Atlantic spring spawning
0
5
10
15
20

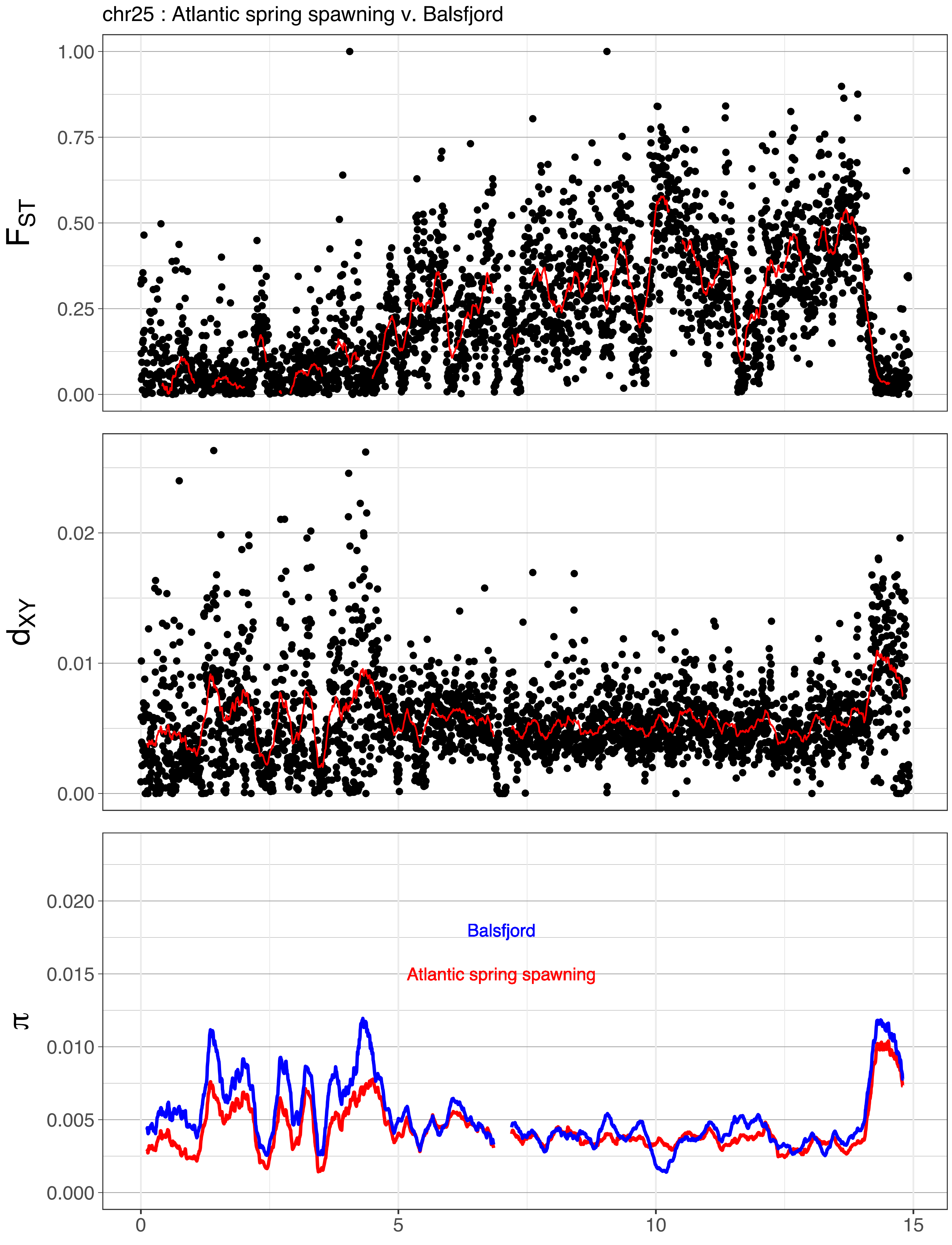
chr25 : Atlantic spring spawning v. Balsfjord
F_{ST}
1.00
0.75
0.50
0.25
0.00
d_{XY}
0.02
0.01
0.00
π
0.020
0.015
0.010
0.005
0.000
Balsfjord
Atlantic spring spawning
0
5
10
15

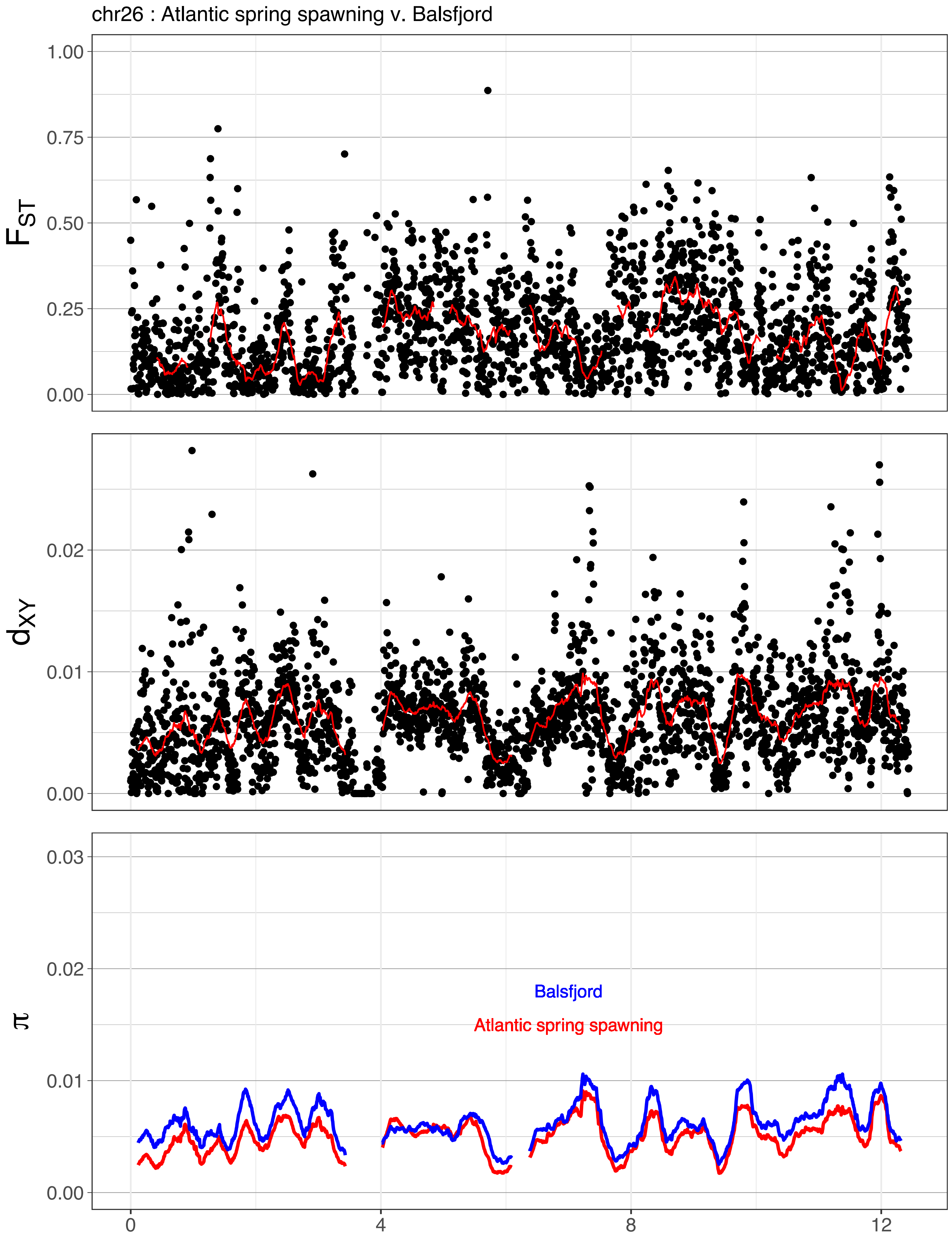

chr26 : Atlantic spring spawning v. Balsfjord
F_{ST}
1.00
0.75
0.50
0.25
0.00
d_{XY}
0.02
0.01
0.00
π
0.03
0.02
0.01
0.00
Balsfjord
Atlantic spring spawning
0
4
8
12
