# Supplementary material for: A Long-Standing Hybrid Population Between Pacific and Atlantic Herring in a Subarctic Fjord of Norway
Source: Genome Biol Evol. 2023 Apr 30;15(5):evad069. doi: 10.1093/gbe/evad069 (PMC10182735; doi:10.1093/gbe/evad069)
Supplement: evad069_Supplementary_Data [file evad069_supplementary_data.zip › Supplementary_Figure_2.pdf]

Supplementary Figure 2. F_ST, dxy and π for individual chromosomes based on the Balsfjord samples, compared with Pacific herring samples of European Russia and Northwest Pacific. Black dots represent 5 kb windows, and the red line is a rolling average. In the π-panels, the blue line shows Balsfjord π, while the red one shows the Pacific herring π.

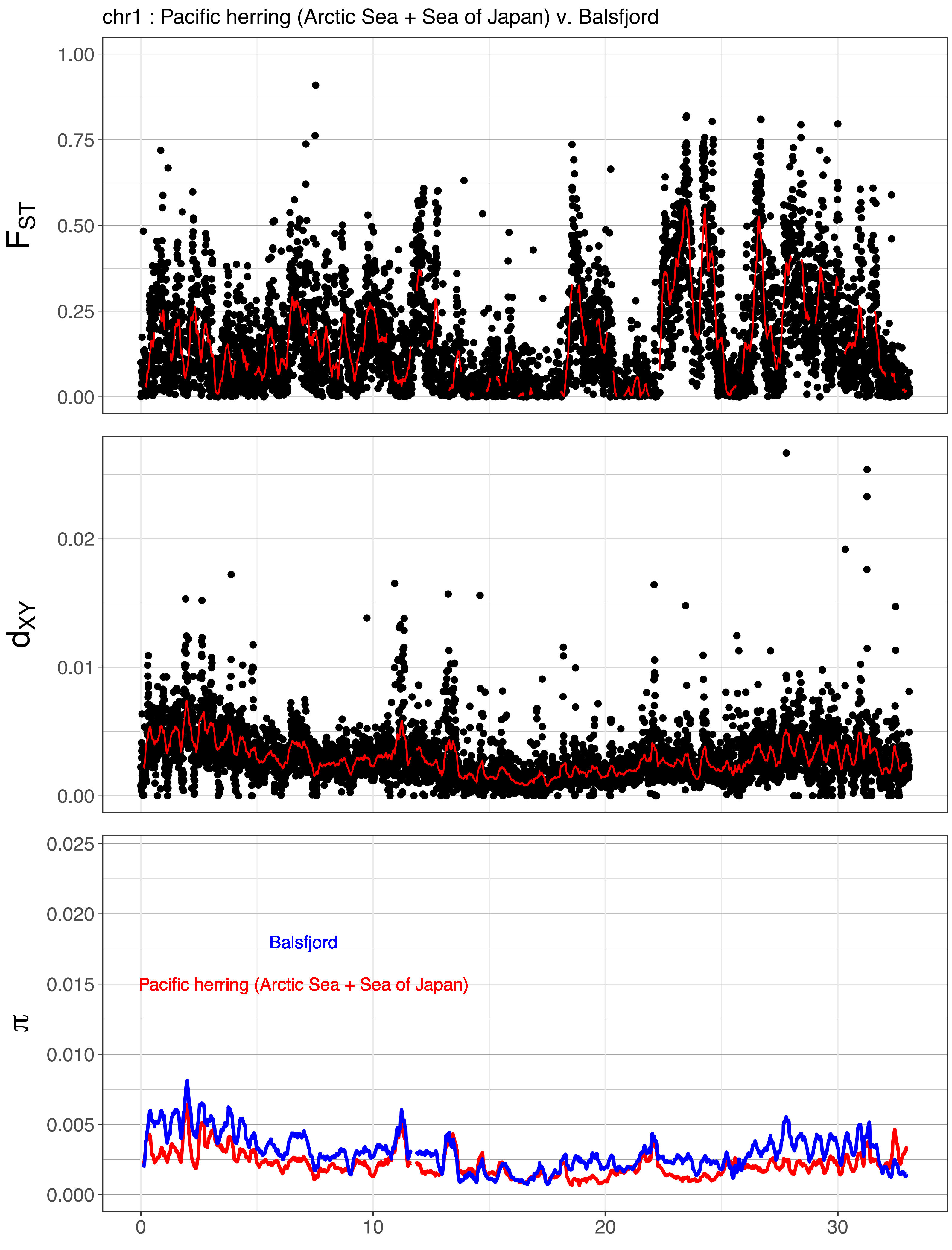

chr1 : Pacific herring (Arctic Sea + Sea of Japan) v. Balsfjord
F_{ST}
1.00
0.75
0.50
0.25
0.00
d_{XY}
0.02
0.01
0.00
π
0.025
0.020
0.015
0.010
0.005
0.000
Balsfjord
Pacific herring (Arctic Sea + Sea of Japan)
0
10
20
30

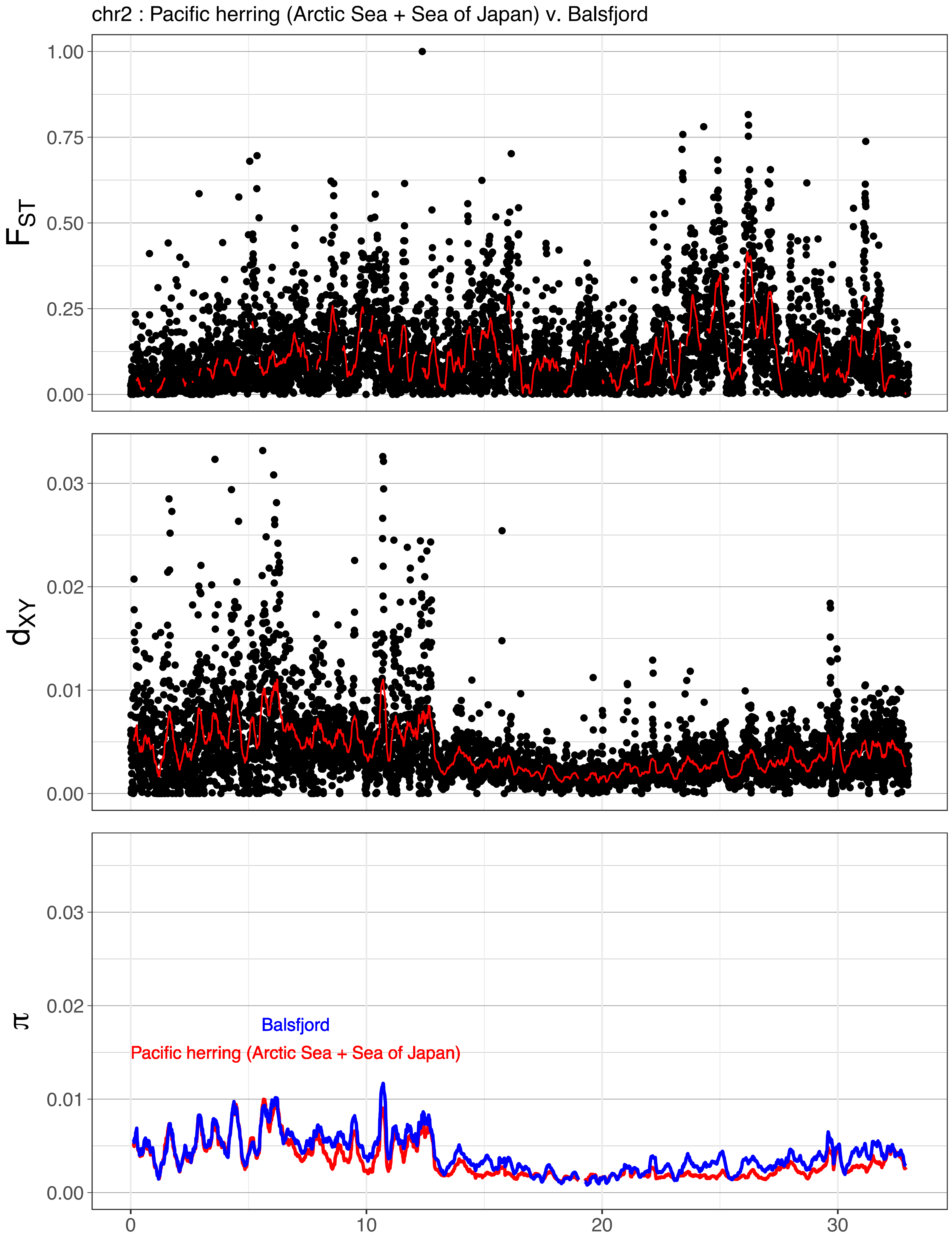

chr2 : Pacific herring (Arctic Sea + Sea of Japan) v. Balsfjord
F_{ST}
1.00
0.75
0.50
0.25
0.00
d_{XY}
0.03
0.02
0.01
0.00
π
0.03
0.02
0.01
0.00
Balsfjord
Pacific herring (Arctic Sea + Sea of Japan)
0
10
20
30

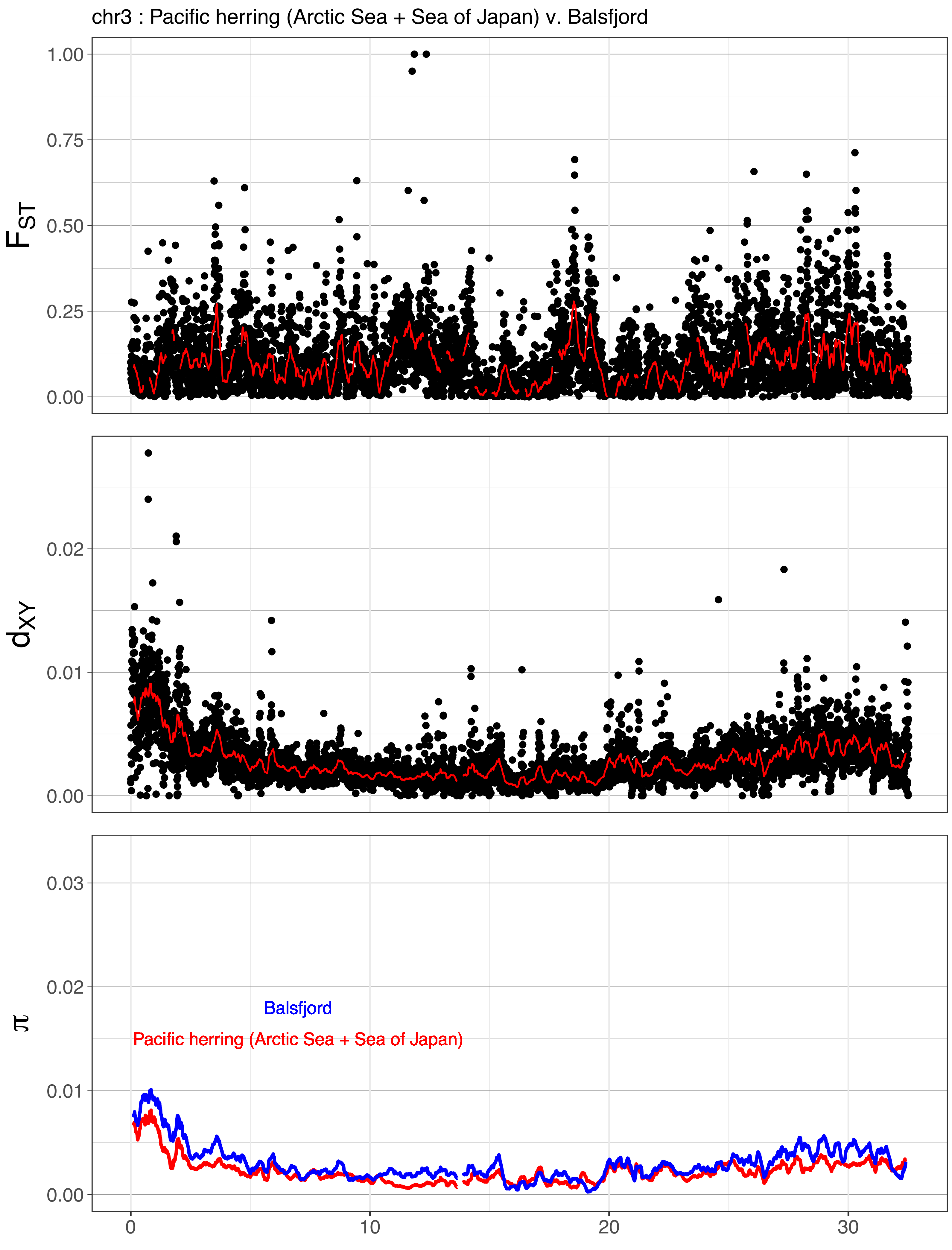
chr3 : Pacific herring (Arctic Sea + Sea of Japan) v. Balsfjord
F_{ST}
1.00
0.75
0.50
0.25
0.00
d_{XY}
0.02
0.01
0.00
π
0.03
0.02
0.01
0.00
Balsfjord
Pacific herring (Arctic Sea + Sea of Japan)
0
10
20
30

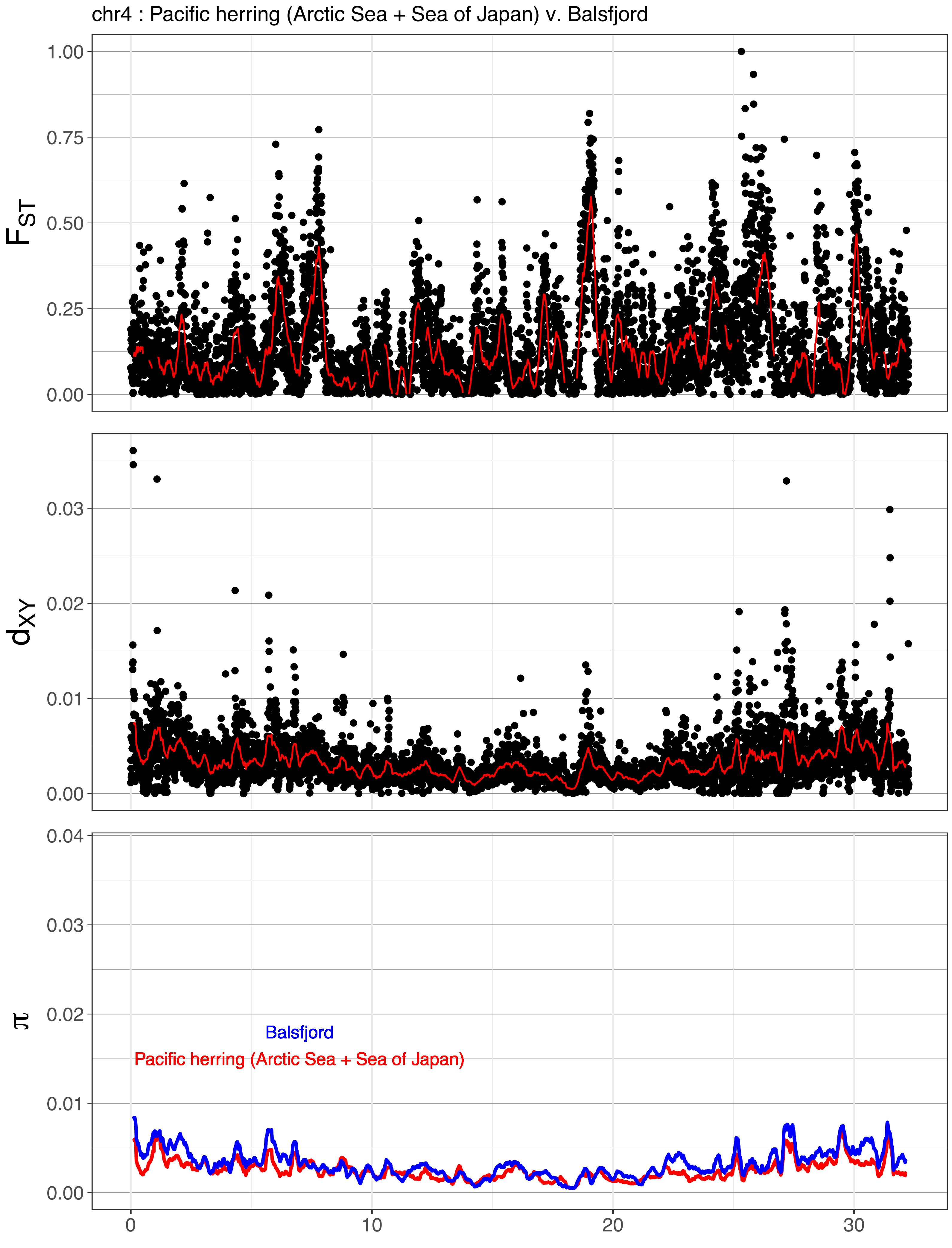
chr4 : Pacific herring (Arctic Sea + Sea of Japan) v. Balsfjord
F_ST
1.00
0.75
0.50
0.25
0.00
d_XY
0.03
0.02
0.01
0.00
π
0.04
0.03
0.02
0.01
0.00
Balsfjord
Pacific herring (Arctic Sea + Sea of Japan)
0
10
20
30

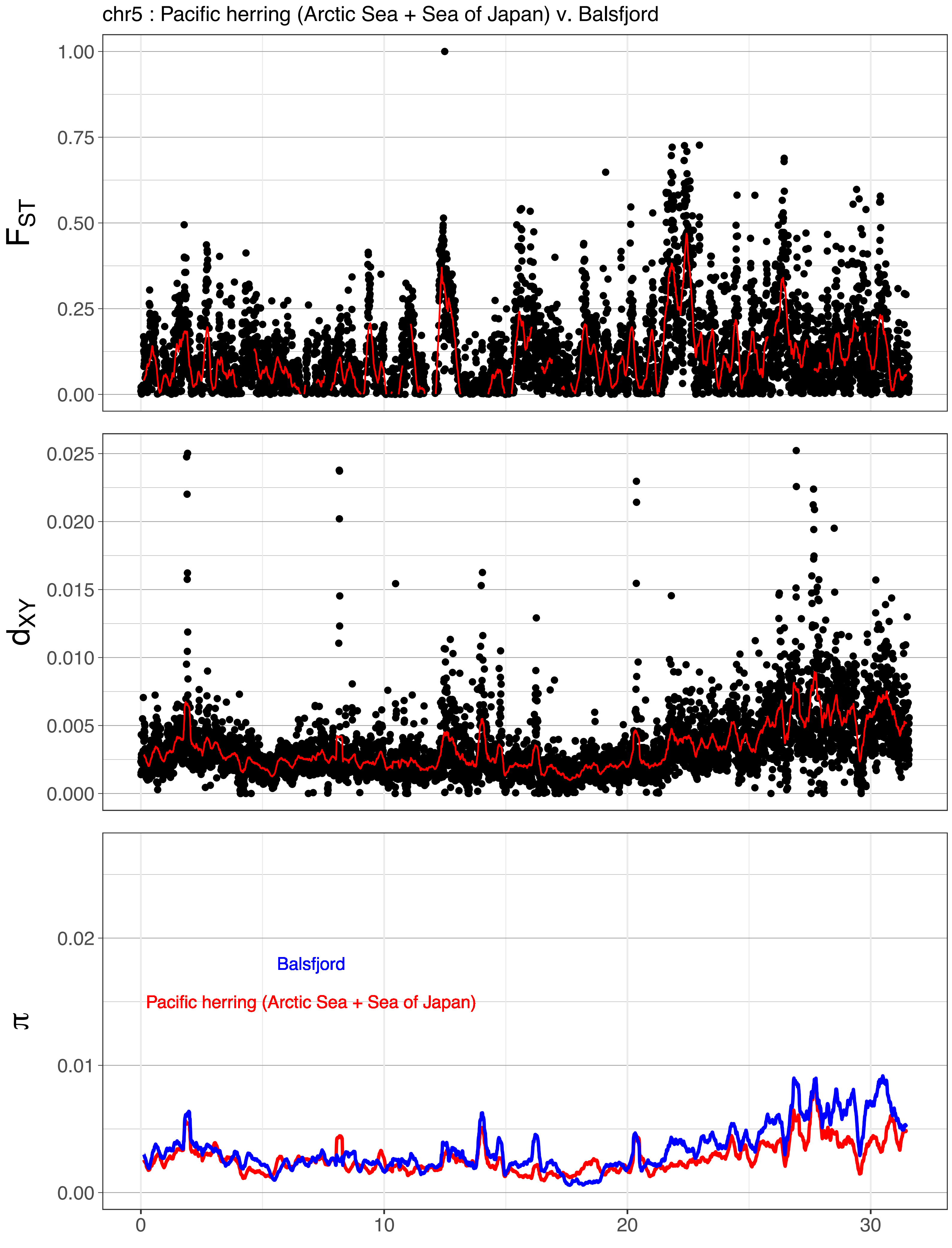
chr5 : Pacific herring (Arctic Sea + Sea of Japan) v. Balsfjord
F_{ST}
1.00
0.75
0.50
0.25
0.00
d_{XY}
0.025
0.020
0.015
0.010
0.005
0.000
π
0.02
0.01
0.00
Balsfjord
Pacific herring (Arctic Sea + Sea of Japan)
0
10
20
30

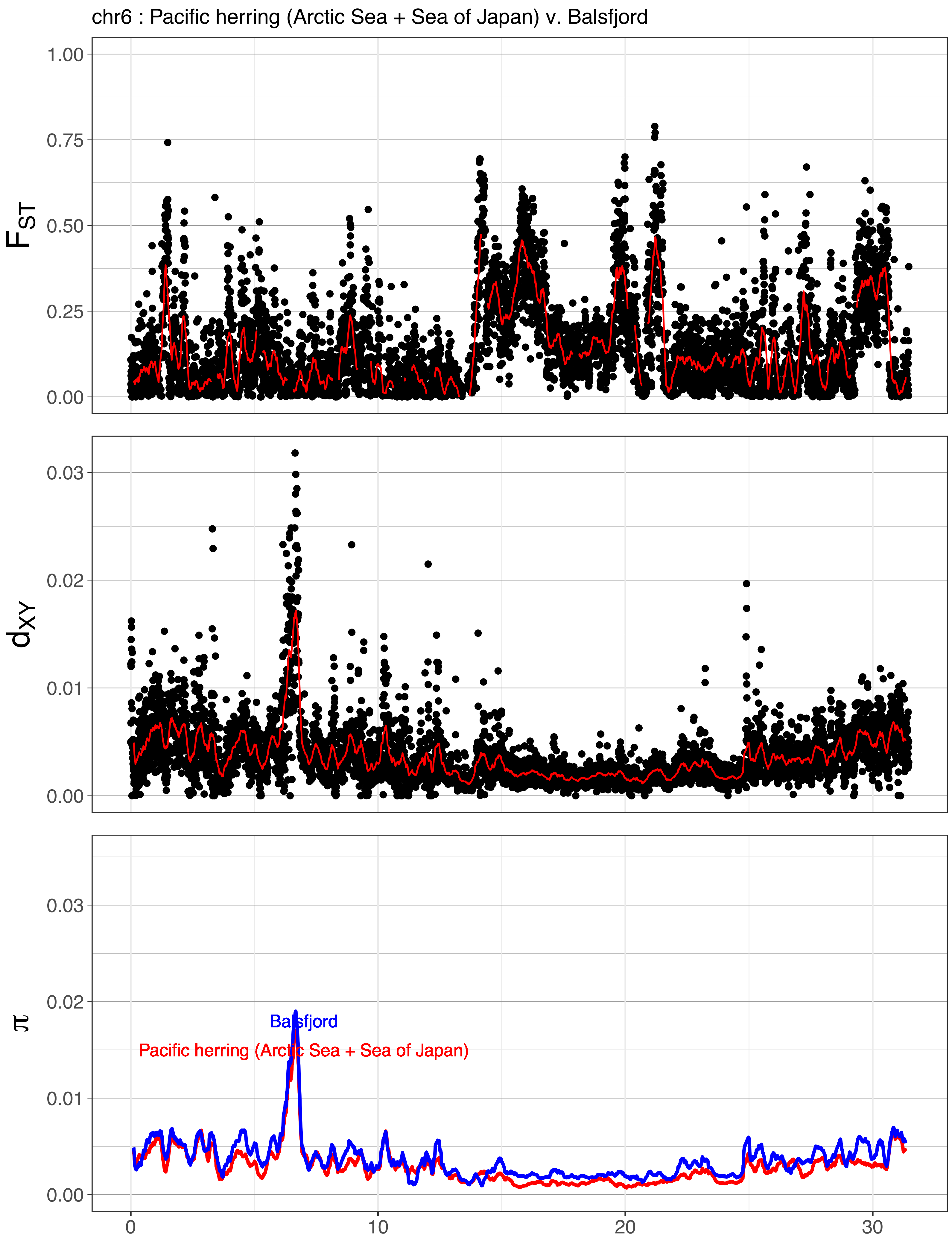
chr6 : Pacific herring (Arctic Sea + Sea of Japan) v. Balsfjord
F_{ST}
1.00
0.75
0.50
0.25
0.00
d_{XY}
0.03
0.02
0.01
0.00
π
0.03
0.02
0.01
0.00
Balsfjord
Pacific herring (Arctic Sea + Sea of Japan)
0
10
20
30

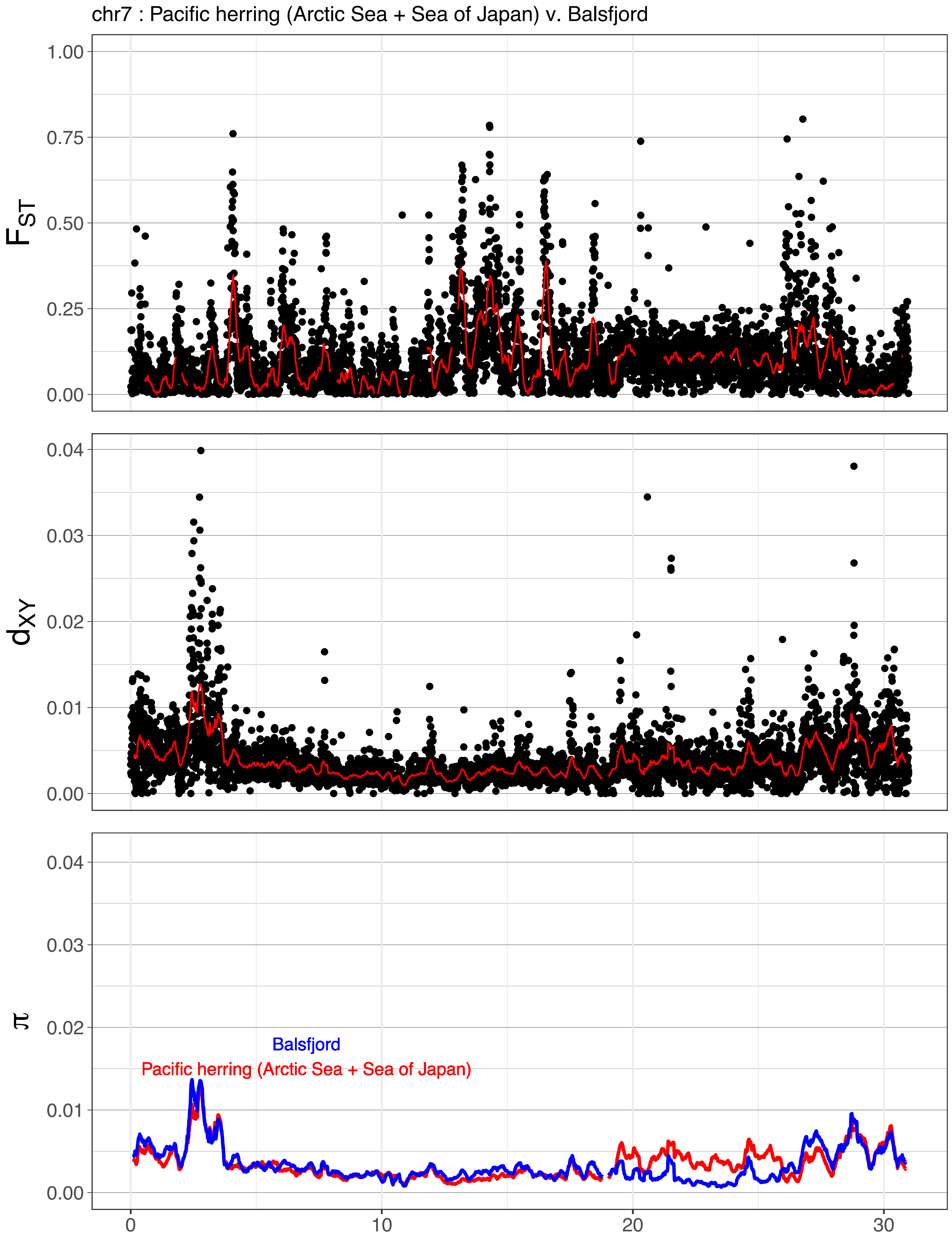

chr7 : Pacific herring (Arctic Sea + Sea of Japan) v. Balsfjord
F_{ST}
1.00
0.75
0.50
0.25
0.00
d_{XY}
0.04
0.03
0.02
0.01
0.00
π
0.04
0.03
0.02
0.01
0.00
Balsfjord
Pacific herring (Arctic Sea + Sea of Japan)
0
10
20
30

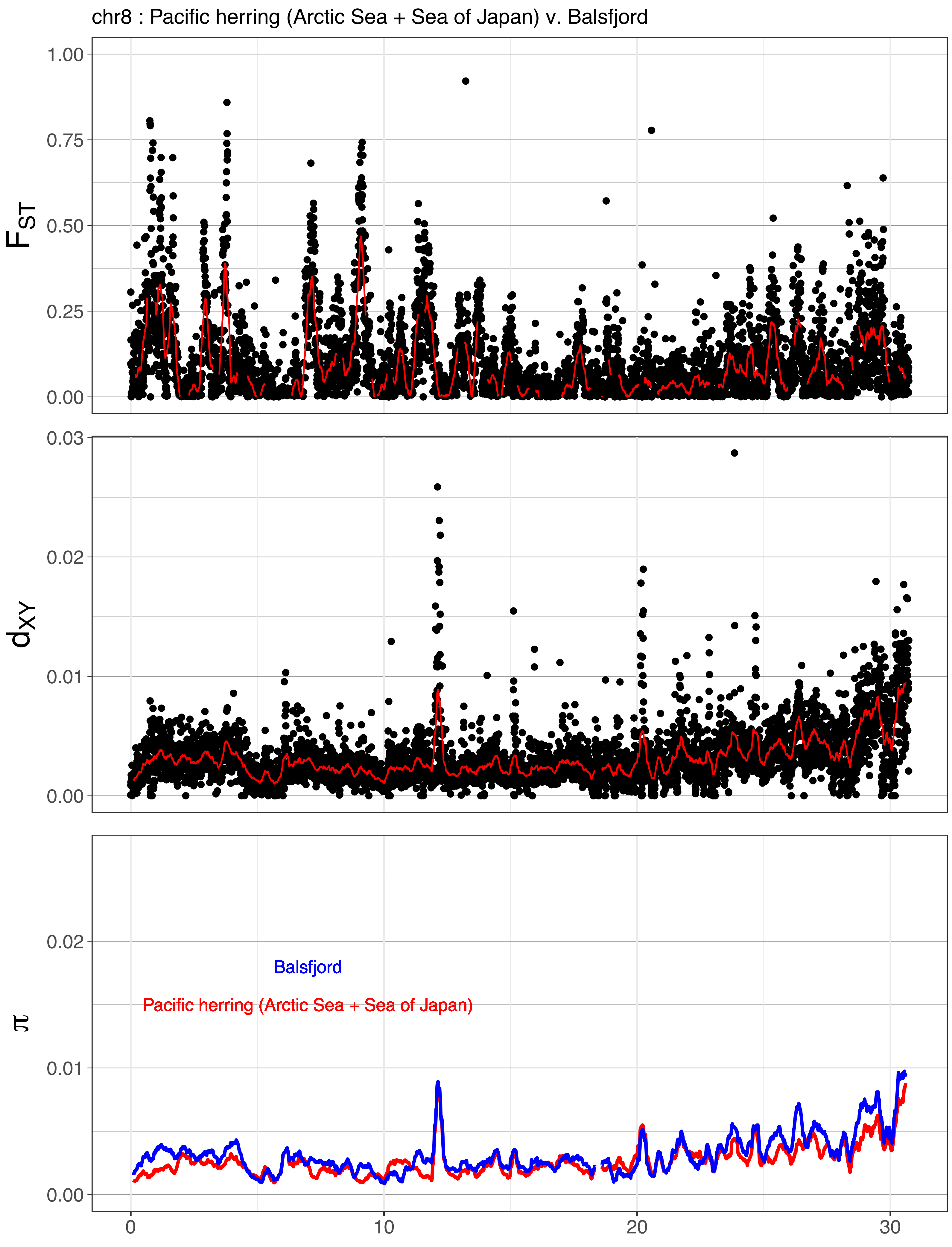

chr8 : Pacific herring (Arctic Sea + Sea of Japan) v. Balsfjord
F_ST
1.00
0.75
0.50
0.25
0.00
d_XY
0.03
0.02
0.01
0.00
π
Balsfjord
Pacific herring (Arctic Sea + Sea of Japan)
0.02
0.01
0.00
0
10
20
30

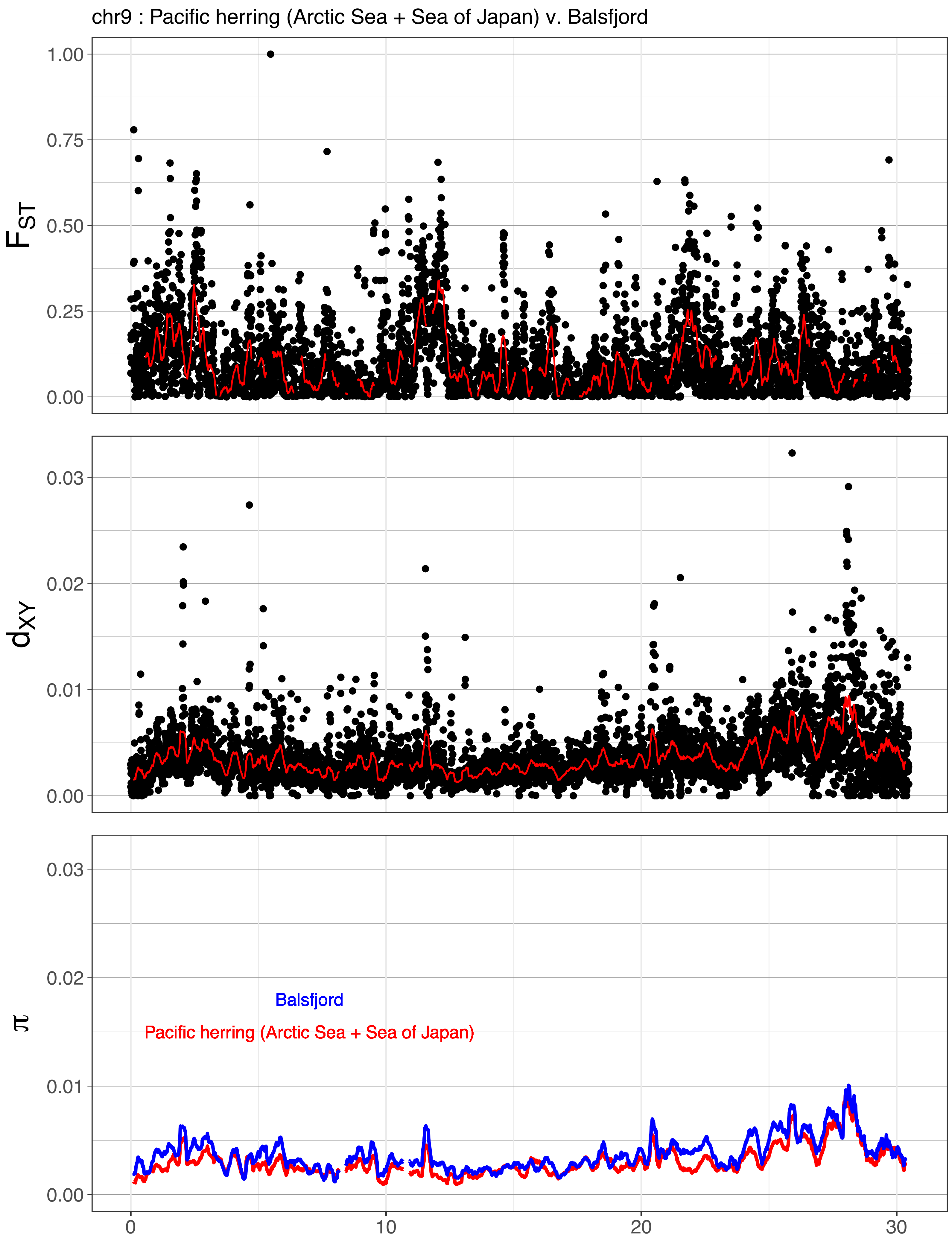
chr9 : Pacific herring (Arctic Sea + Sea of Japan) v. Balsfjord
F_{ST}
1.00
0.75
0.50
0.25
0.00
d_{XY}
0.03
0.02
0.01
0.00
π
0.03
0.02
0.01
0.00
Balsfjord
Pacific herring (Arctic Sea + Sea of Japan)
0
10
20
30

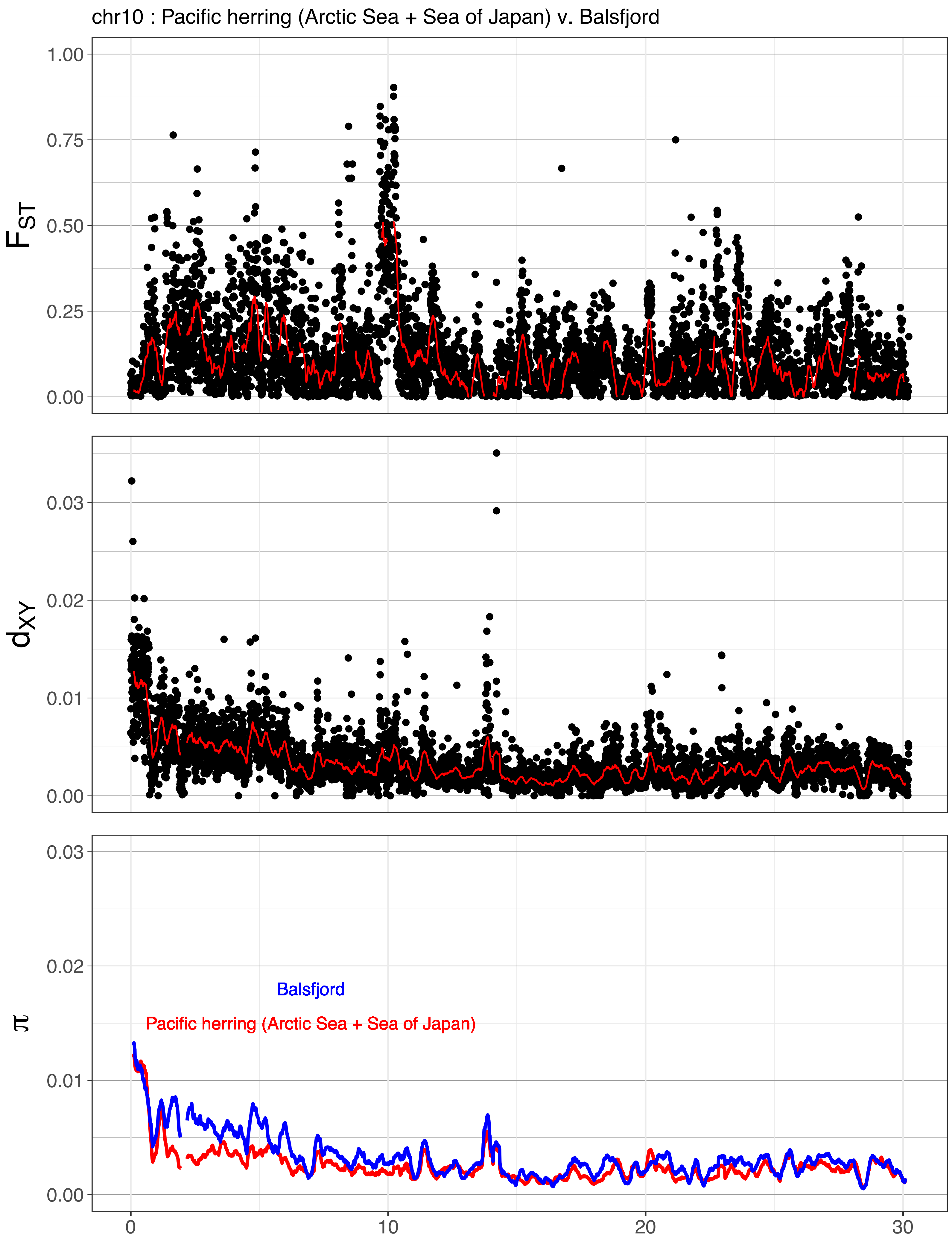
chr10 : Pacific herring (Arctic Sea + Sea of Japan) v. Balsfjord
F_{ST}
1.00
0.75
0.50
0.25
0.00
d_{XY}
0.03
0.02
0.01
0.00
π
0.03
0.02
0.01
0.00
Balsfjord
Pacific herring (Arctic Sea + Sea of Japan)
0
10
20
30

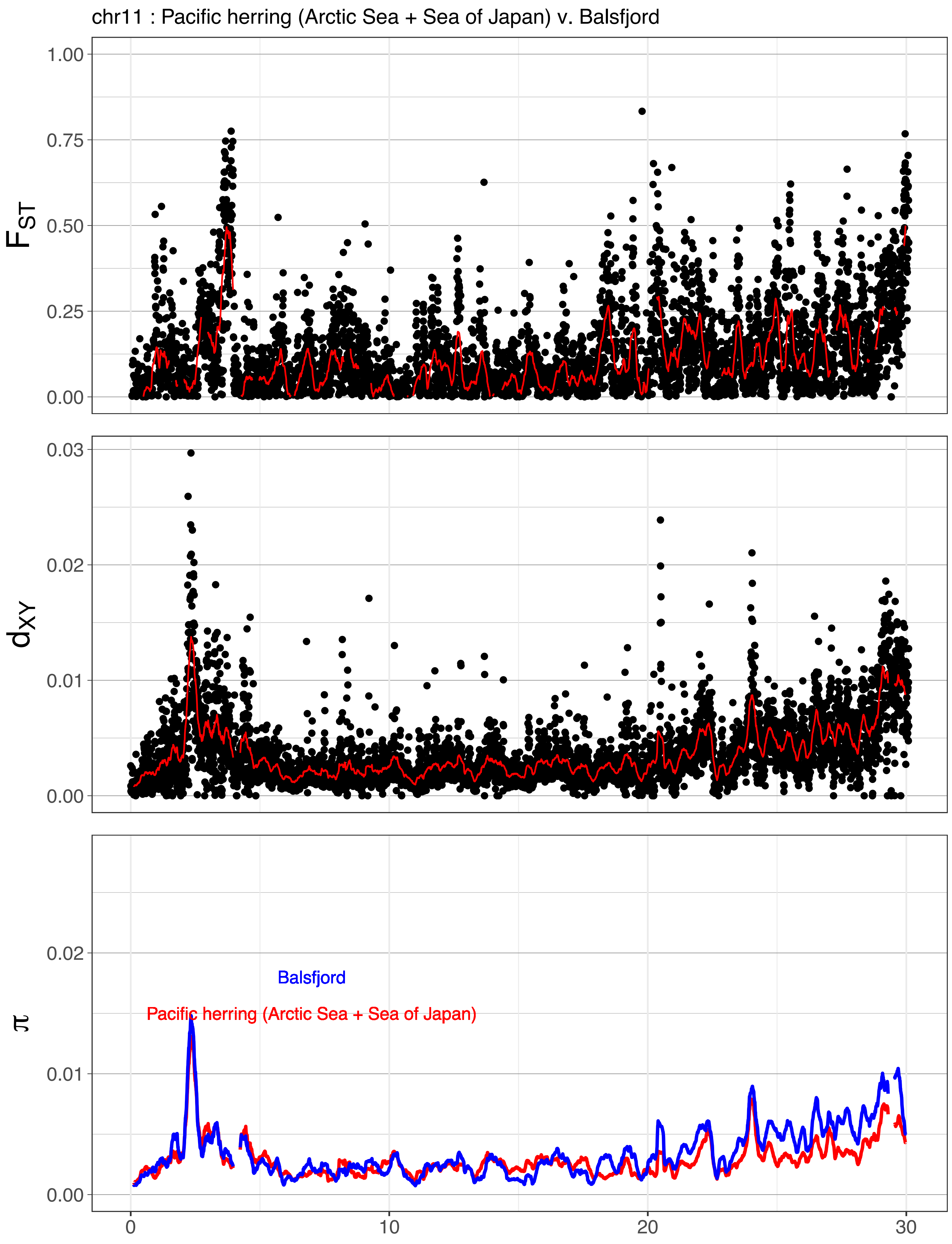

chr11 : Pacific herring (Arctic Sea + Sea of Japan) v. Balsfjord
F_{ST}
1.00
0.75
0.50
0.25
0.00
d_{XY}
0.03
0.02
0.01
0.00
π
0.02
0.01
0.00
Balsfjord
Pacific herring (Arctic Sea + Sea of Japan)
0
10
20
30

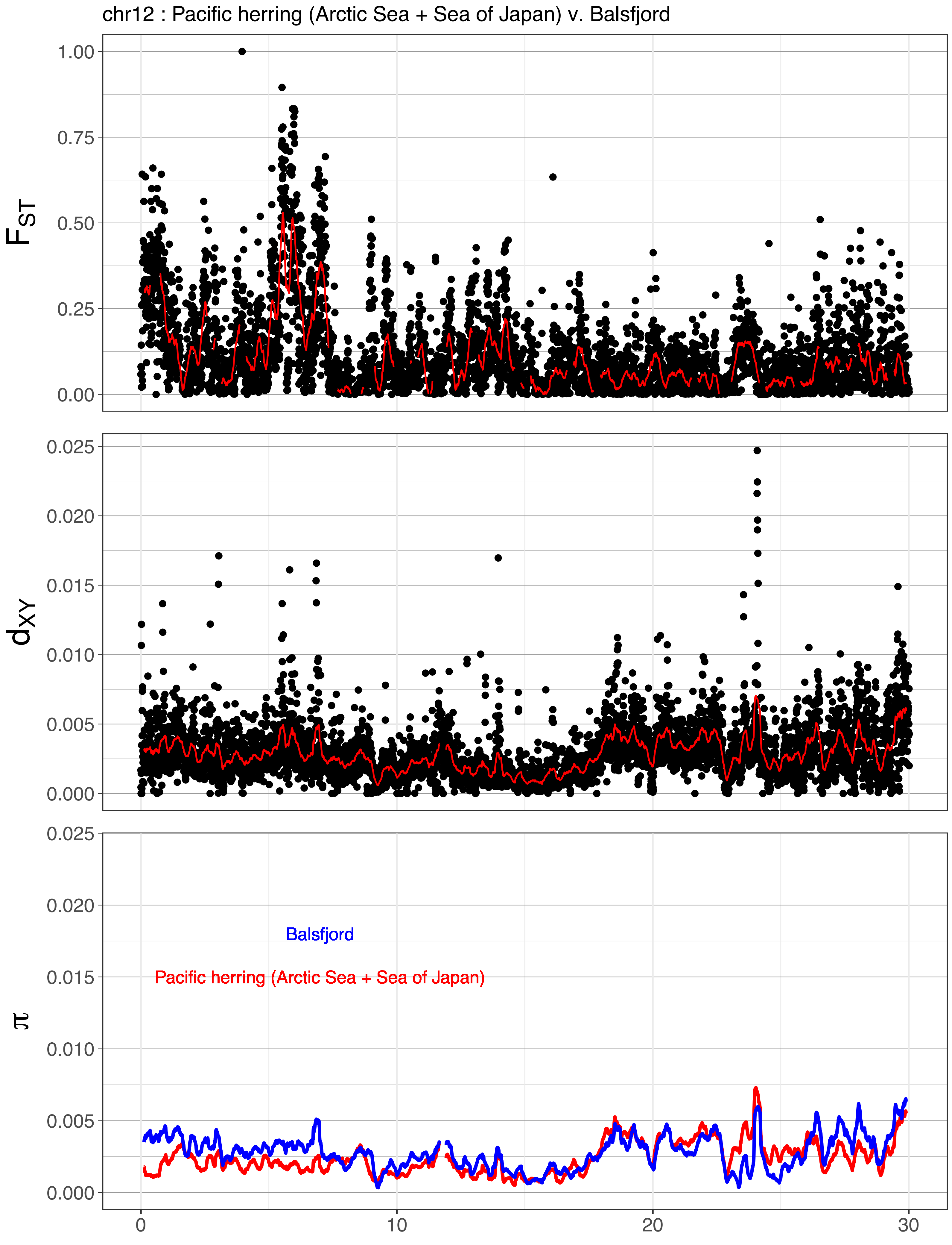

chr12 : Pacific herring (Arctic Sea + Sea of Japan) v. Balsfjord
F_{ST}
1.00
0.75
0.50
0.25
0.00
d_{XY}
0.025
0.020
0.015
0.010
0.005
0.000
π
Balsfjord
Pacific herring (Arctic Sea + Sea of Japan)
0.025
0.020
0.015
0.010
0.005
0.000
0
10
20
30

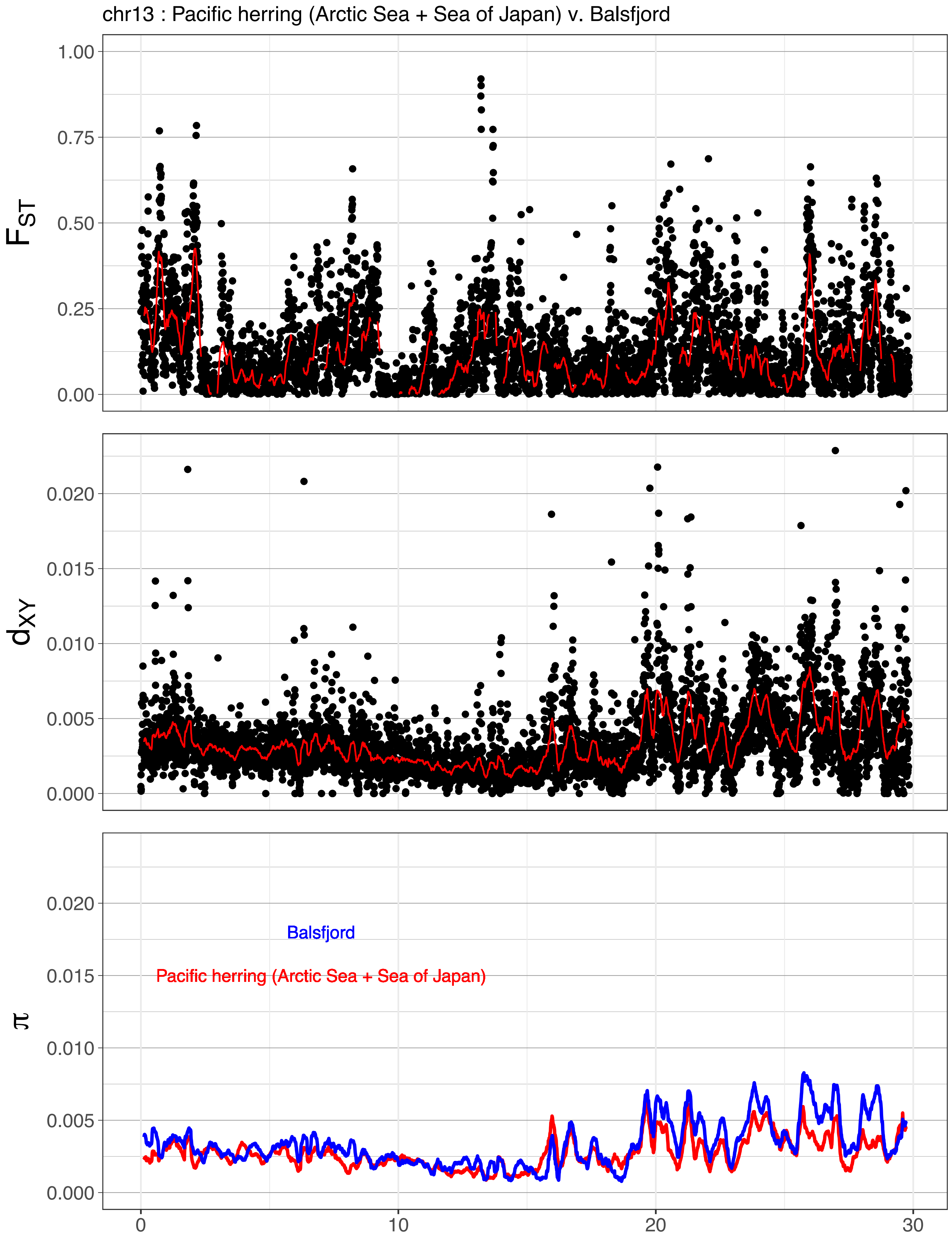

chr13 : Pacific herring (Arctic Sea + Sea of Japan) v. Balsfjord
F_{ST}
1.00
0.75
0.50
0.25
0.00
d_{XY}
0.020
0.015
0.010
0.005
0.000
π
Balsfjord
Pacific herring (Arctic Sea + Sea of Japan)
0.020
0.015
0.010
0.005
0.000
0
10
20
30

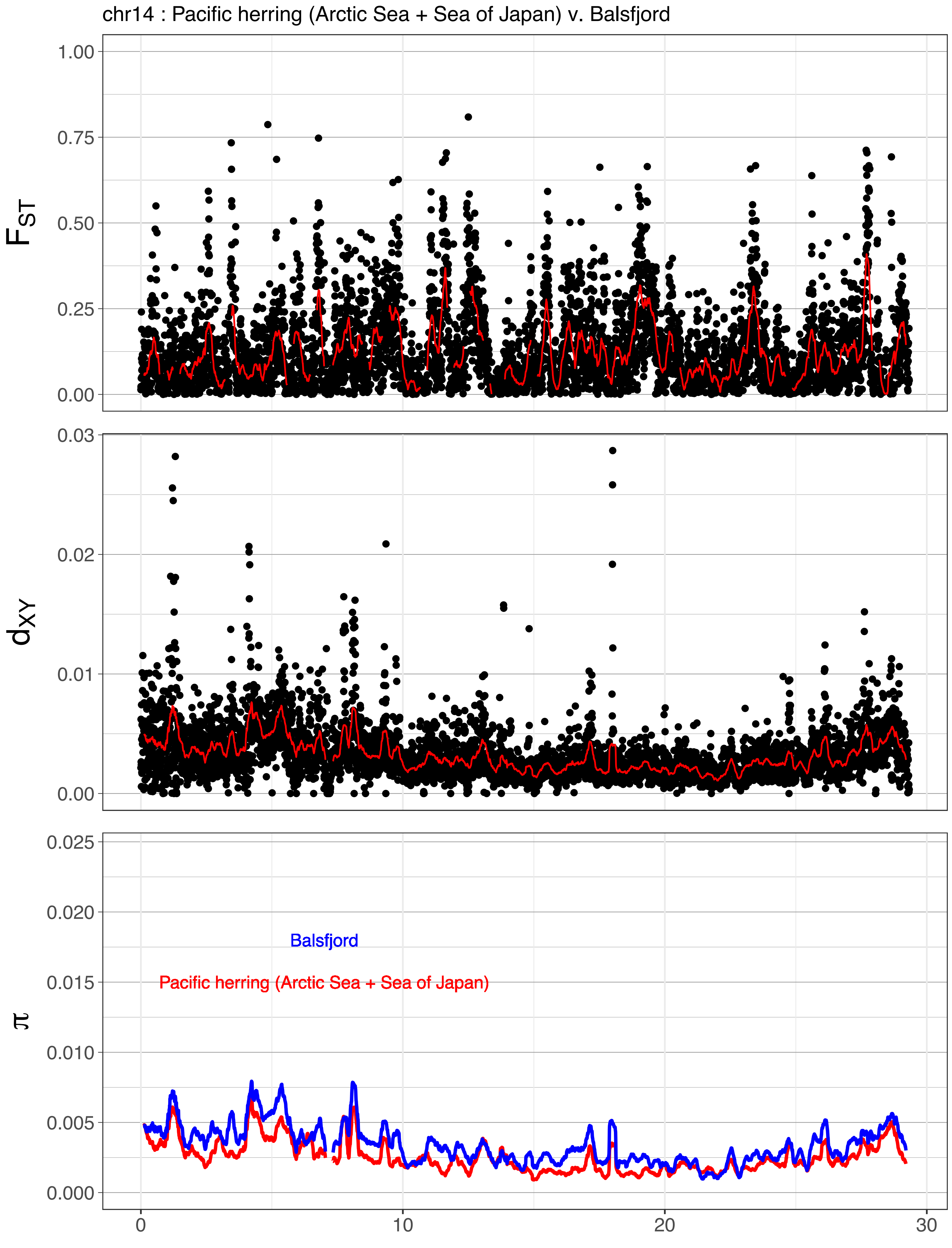

chr14 : Pacific herring (Arctic Sea + Sea of Japan) v. Balsfjord
F_{ST}
1.00
0.75
0.50
0.25
0.00
d_{XY}
0.03
0.02
0.01
0.00
π
0.025
0.020
0.015
0.010
0.005
0.000
Balsfjord
Pacific herring (Arctic Sea + Sea of Japan)
0
10
20
30

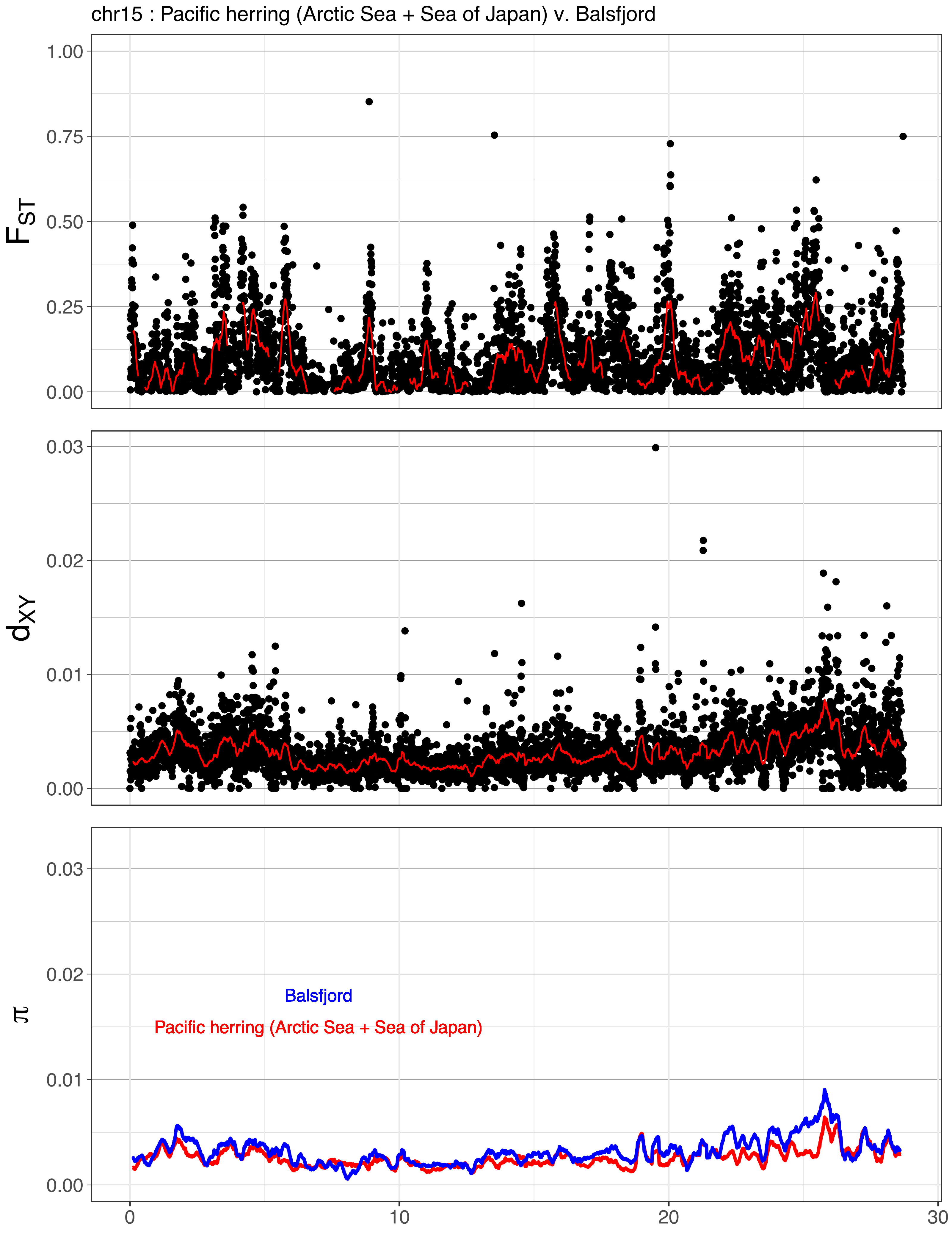

chr15 : Pacific herring (Arctic Sea + Sea of Japan) v. Balsfjord
F_{ST}
1.00
0.75
0.50
0.25
0.00
d_{XY}
0.03
0.02
0.01
0.00
π
0.03
0.02
0.01
0.00
Balsfjord
Pacific herring (Arctic Sea + Sea of Japan)
0
10
20
30

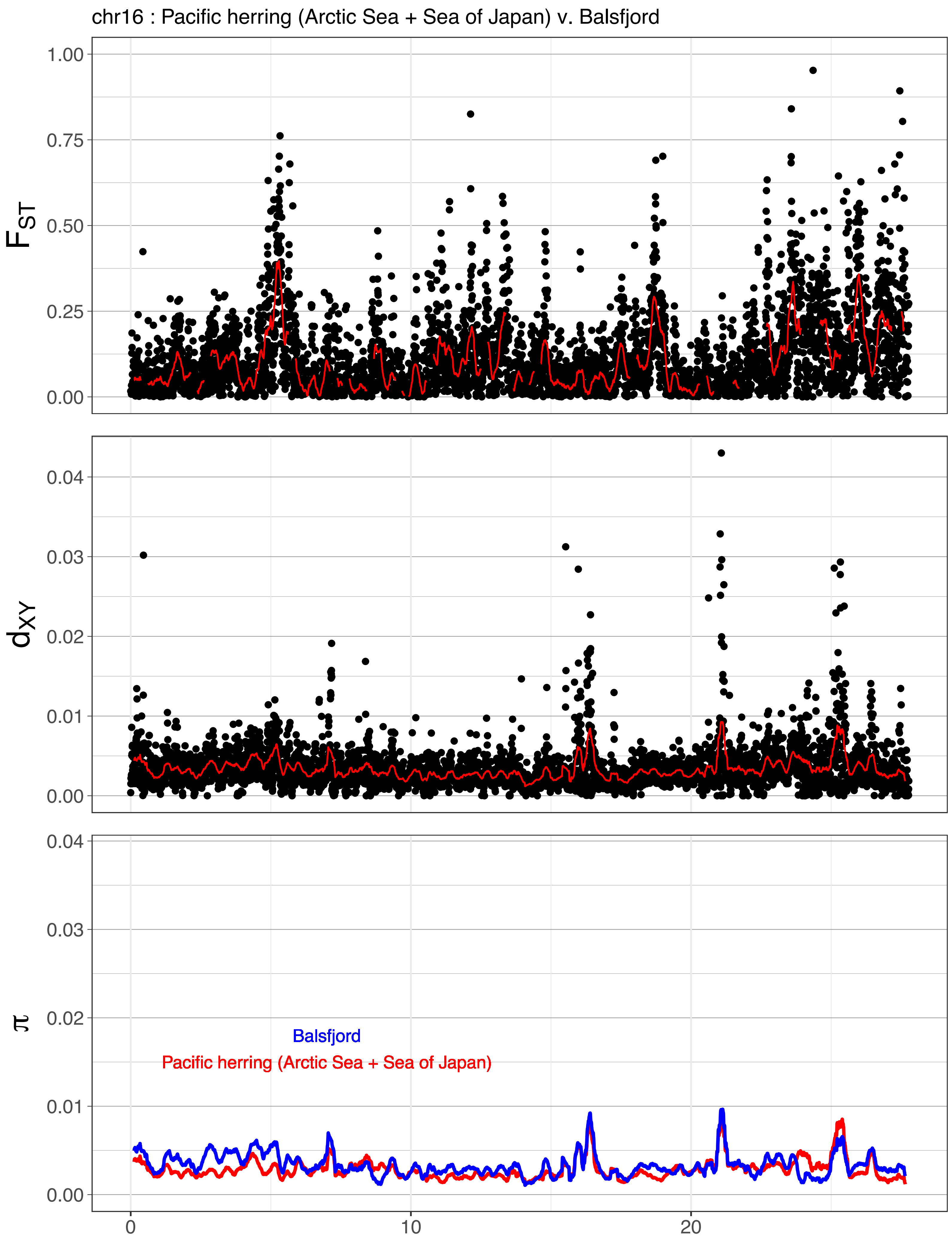
chr16 : Pacific herring (Arctic Sea + Sea of Japan) v. Balsfjord
FST
1.00
0.75
0.50
0.25
0.00
dXY
0.04
0.03
0.02
0.01
0.00
π
0.04
0.03
0.02
0.01
0.00
Balsfjord
Pacific herring (Arctic Sea + Sea of Japan)
0
10
20

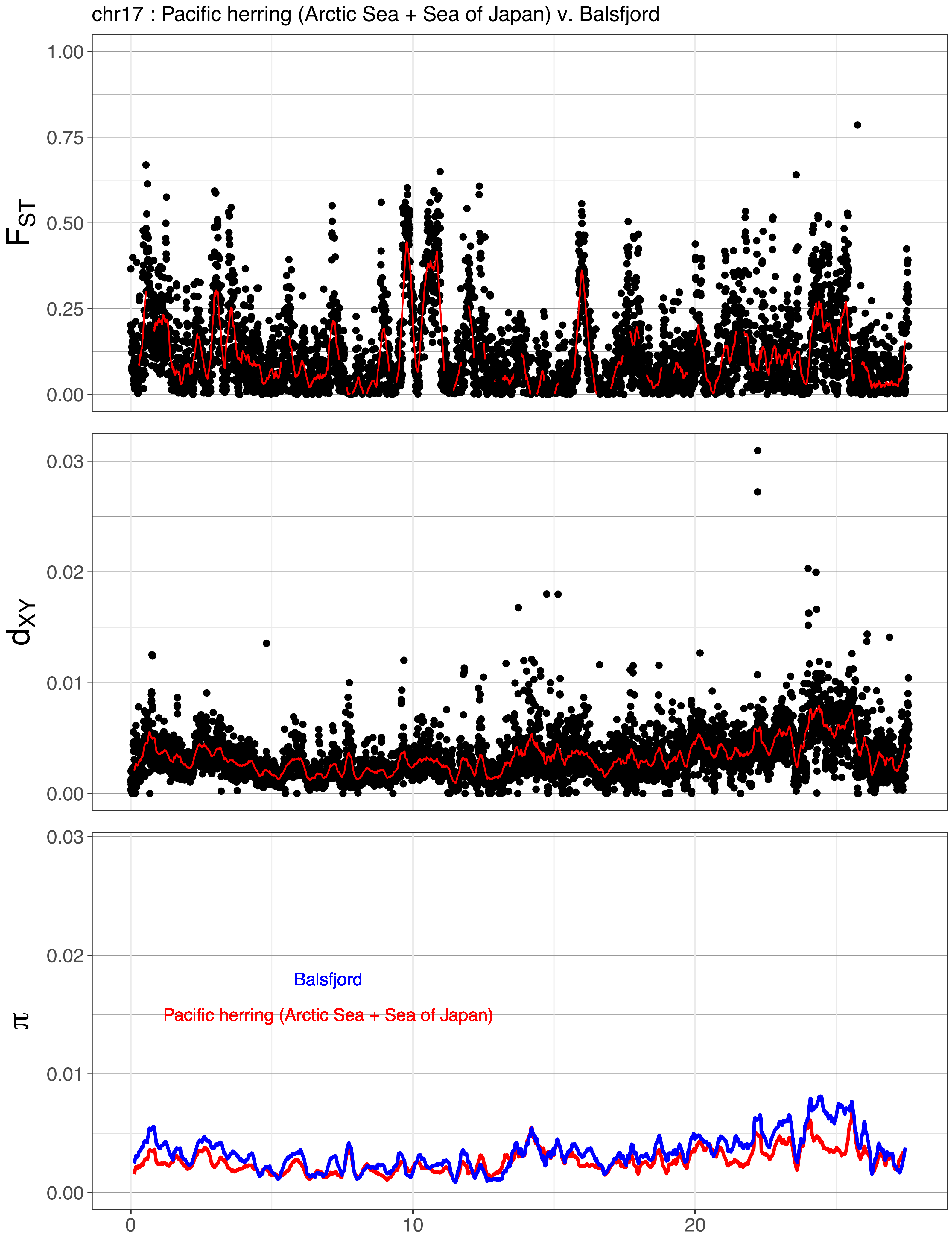

chr17 : Pacific herring (Arctic Sea + Sea of Japan) v. Balsfjord
F_{ST}
1.00
0.75
0.50
0.25
0.00
d_{XY}
0.03
0.02
0.01
0.00
π
Balsfjord
Pacific herring (Arctic Sea + Sea of Japan)
0
10
20

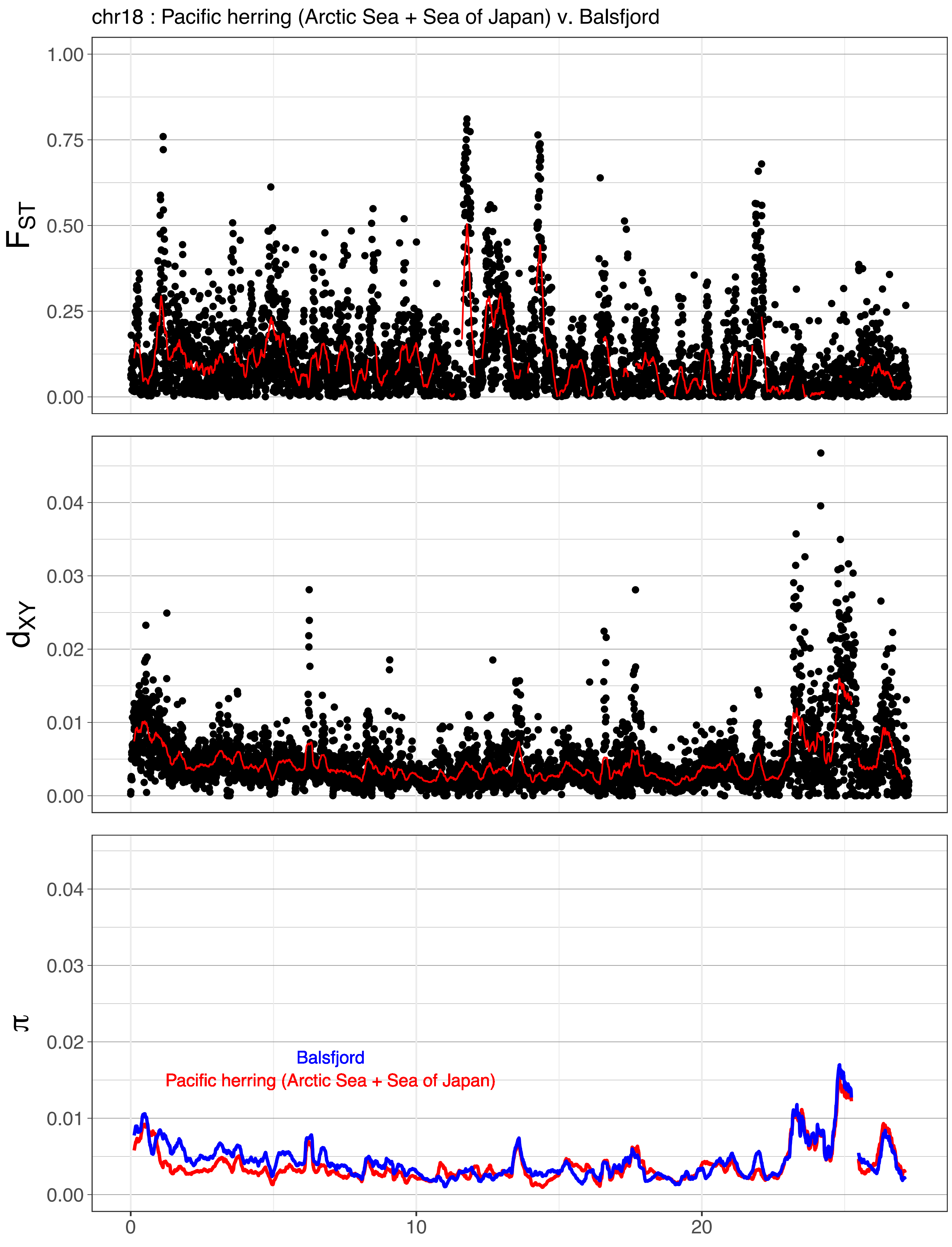

chr18 : Pacific herring (Arctic Sea + Sea of Japan) v. Balsfjord
FST
1.00
0.75
0.50
0.25
0.00
dXY
0.04
0.03
0.02
0.01
0.00
π
0.04
0.03
0.02
0.01
0.00
Balsfjord
Pacific herring (Arctic Sea + Sea of Japan)
0
10
20

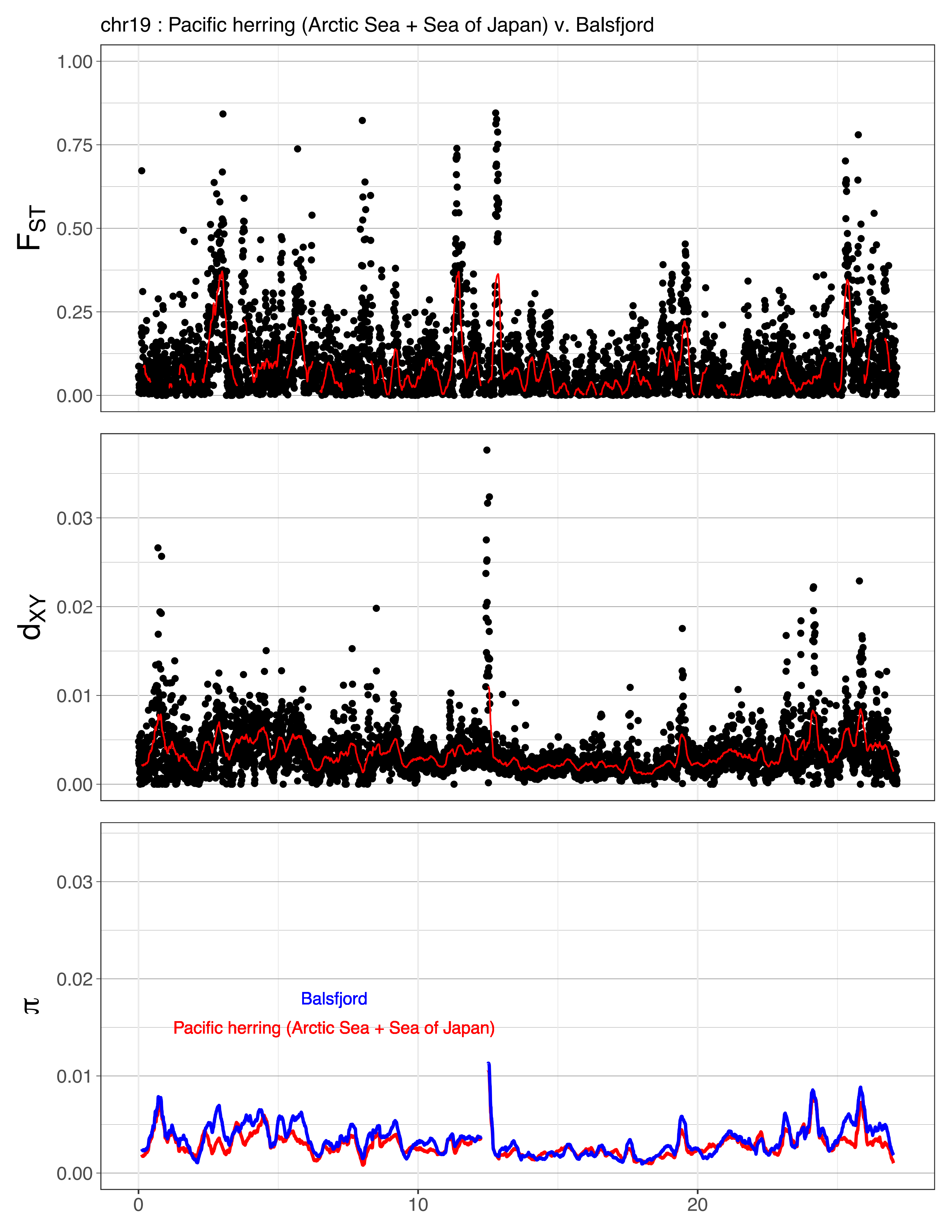
chr19 : Pacific herring (Arctic Sea + Sea of Japan) v. Balsfjord
F_{ST}
1.00
0.75
0.50
0.25
0.00
d_{XY}
0.03
0.02
0.01
0.00
π
0.03
0.02
0.01
0.00
Balsfjord
Pacific herring (Arctic Sea + Sea of Japan)
0
10
20

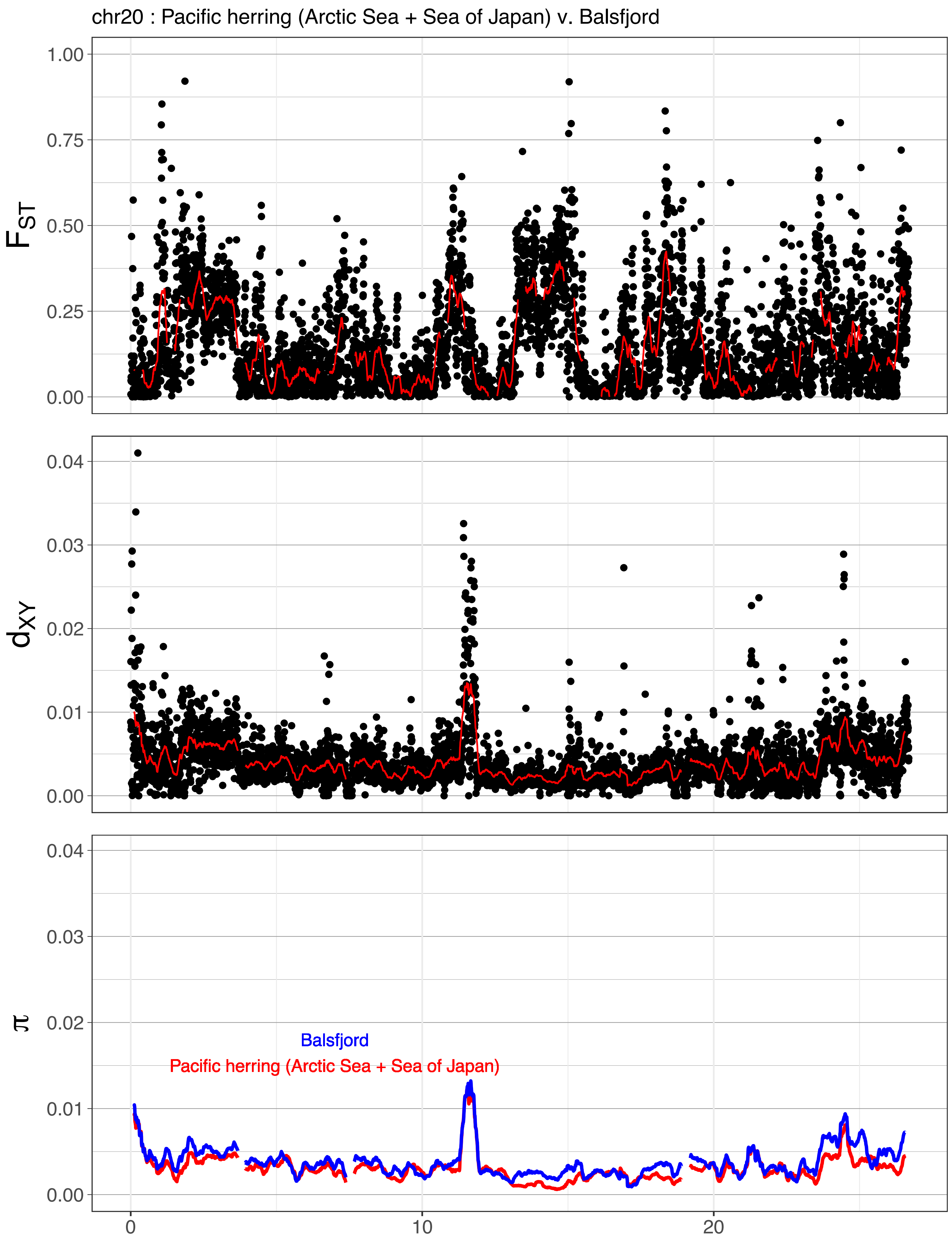

chr20 : Pacific herring (Arctic Sea + Sea of Japan) v. Balsfjord
F_{ST}
1.00
0.75
0.50
0.25
0.00
d_{XY}
0.04
0.03
0.02
0.01
0.00
π
0.04
0.03
0.02
0.01
0.00
Balsfjord
Pacific herring (Arctic Sea + Sea of Japan)
0
10
20

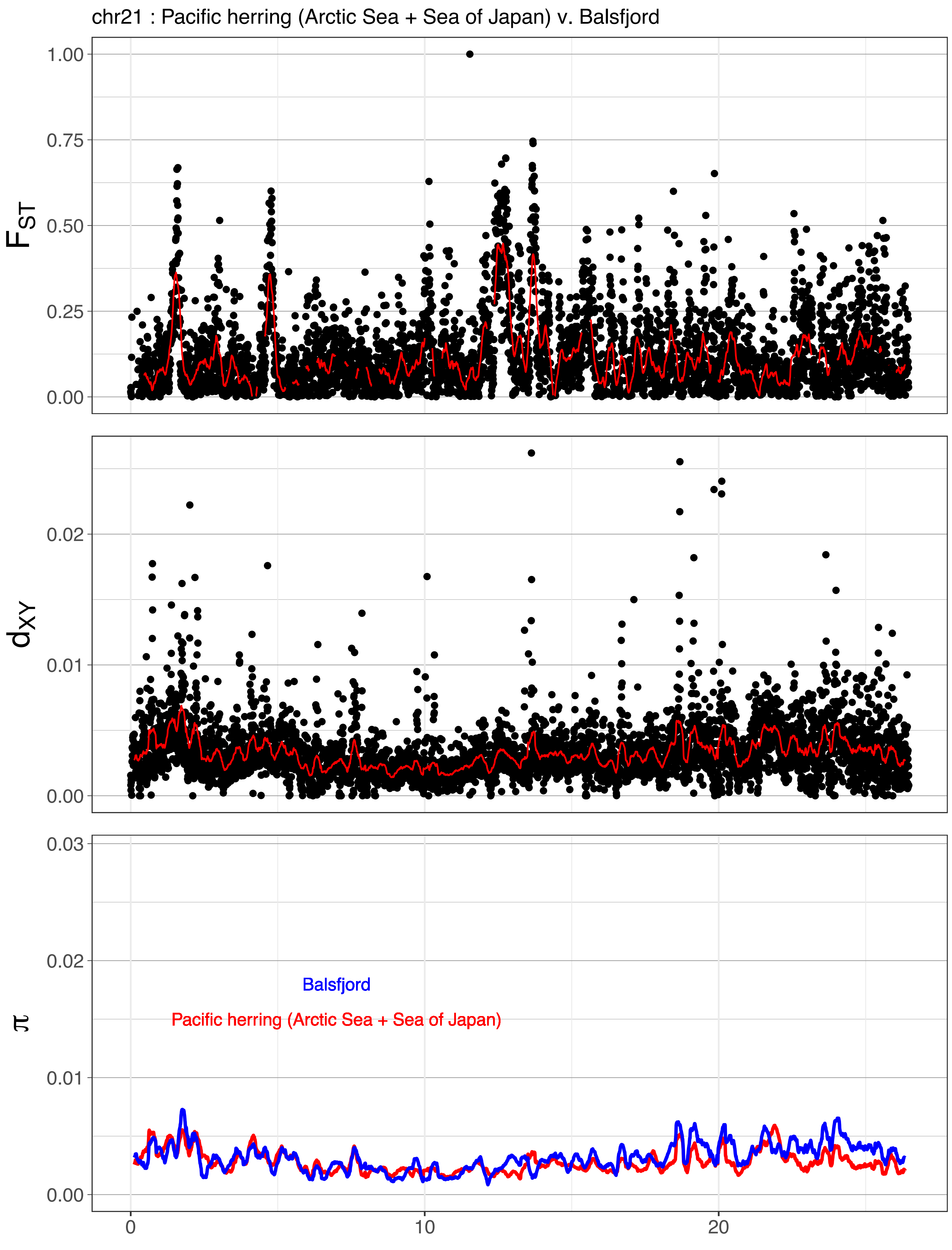

chr21 : Pacific herring (Arctic Sea + Sea of Japan) v. Balsfjord
F_{ST}
1.00
0.75
0.50
0.25
0.00
d_{XY}
0.02
0.01
0.00
π
0.03
0.02
0.01
0.00
Balsfjord
Pacific herring (Arctic Sea + Sea of Japan)
0
10
20

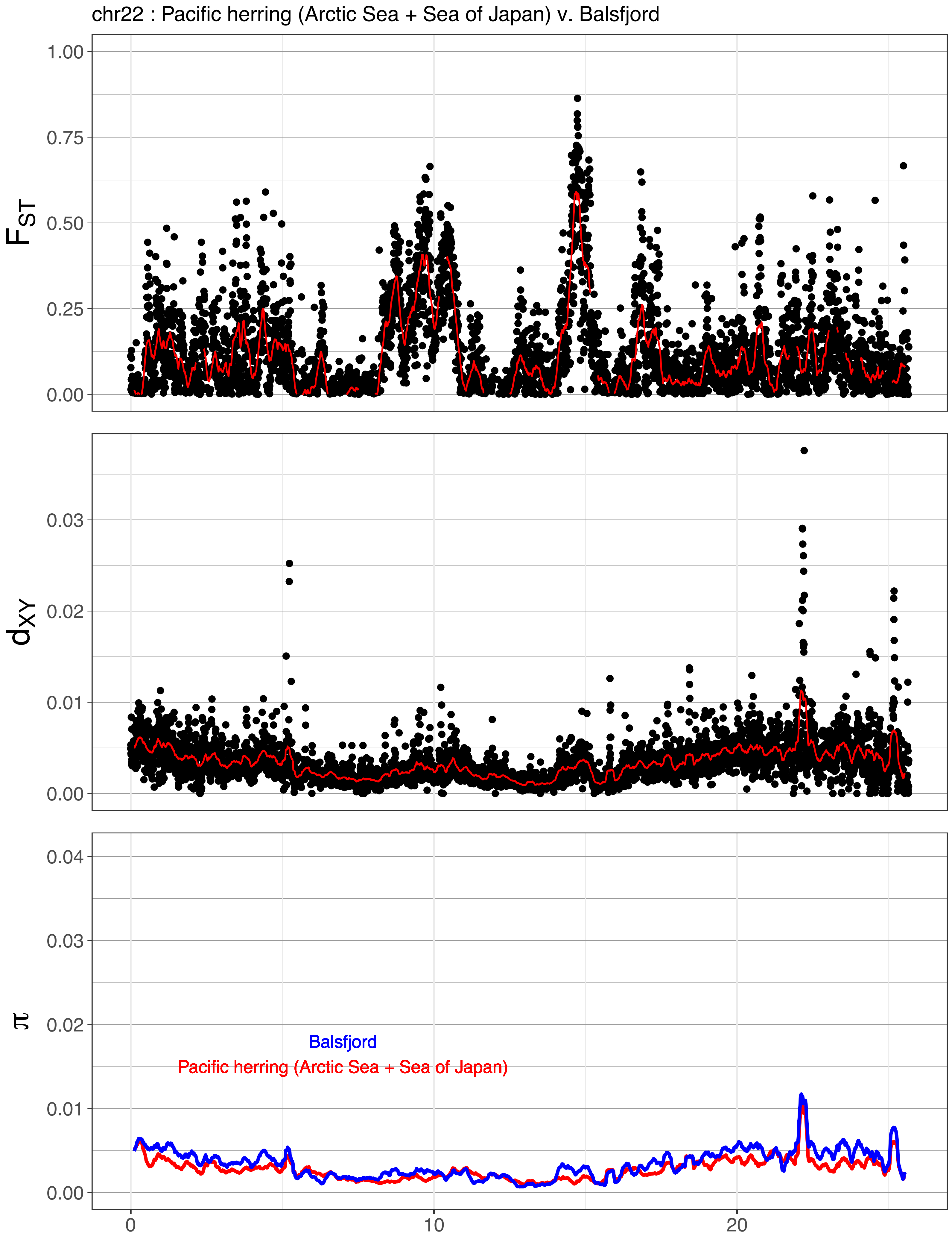

chr22 : Pacific herring (Arctic Sea + Sea of Japan) v. Balsfjord
F_ST
1.00
0.75
0.50
0.25
0.00
d_XY
0.03
0.02
0.01
0.00
π
0.04
0.03
0.02
0.01
0.00
Balsfjord
Pacific herring (Arctic Sea + Sea of Japan)
0
10
20

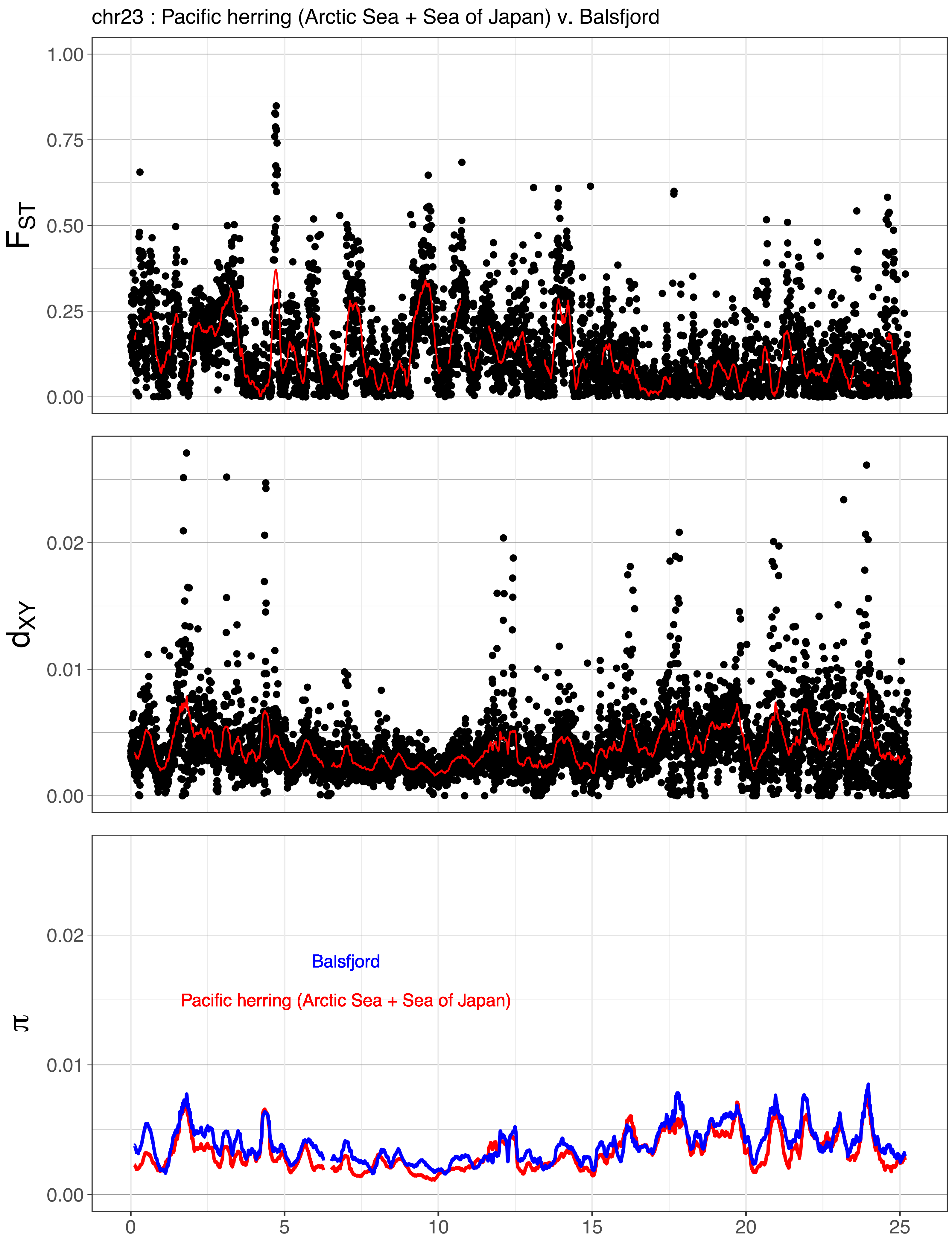
chr23 : Pacific herring (Arctic Sea + Sea of Japan) v. Balsfjord
F_ST
d_XY
π
1.00
0.75
0.50
0.25
0.00
0.02
0.01
0.00
0.02
0.01
0.00
Balsfjord
Pacific herring (Arctic Sea + Sea of Japan)
0
5
10
15
20
25

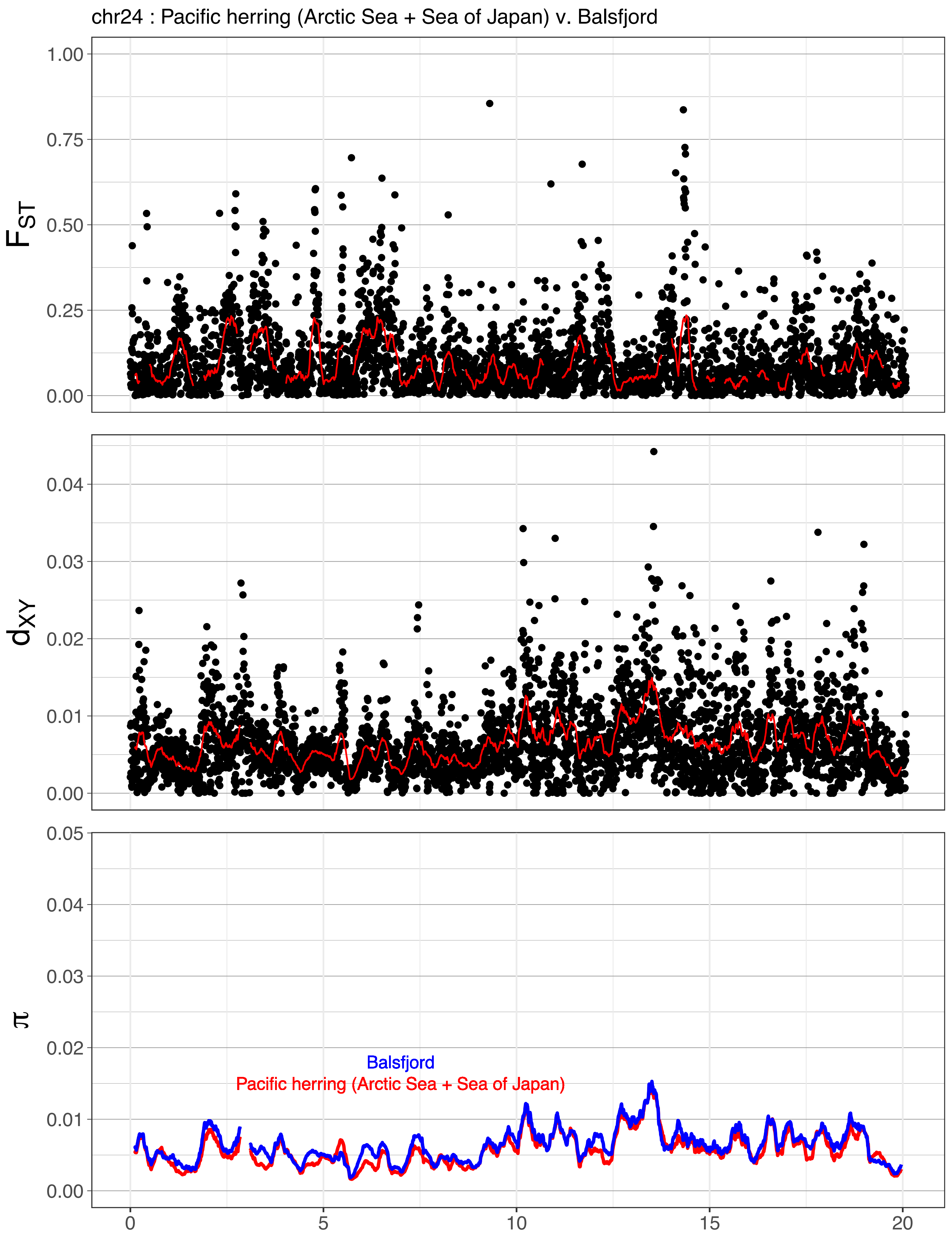

chr24 : Pacific herring (Arctic Sea + Sea of Japan) v. Balsfjord
F_{ST}
1.00
0.75
0.50
0.25
0.00
d_{XY}
0.04
0.03
0.02
0.01
0.00
π
0.05
0.04
0.03
0.02
0.01
0.00
Balsfjord
Pacific herring (Arctic Sea + Sea of Japan)
0
5
10
15
20

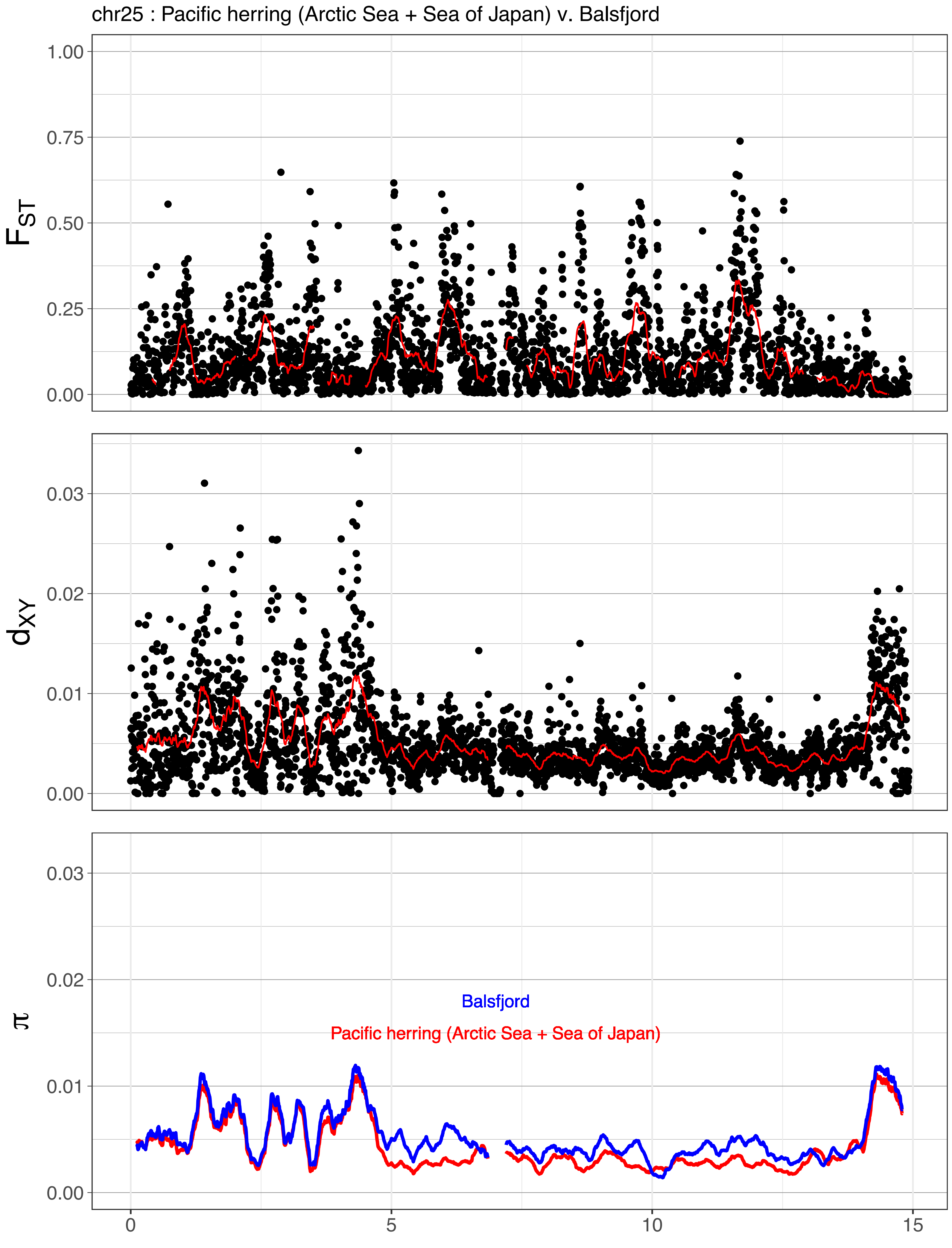

chr25 : Pacific herring (Arctic Sea + Sea of Japan) v. Balsfjord
F_{ST}
1.00
0.75
0.50
0.25
0.00
d_{XY}
0.03
0.02
0.01
0.00
π
0.03
0.02
0.01
0.00
Balsfjord
Pacific herring (Arctic Sea + Sea of Japan)
0
5
10
15

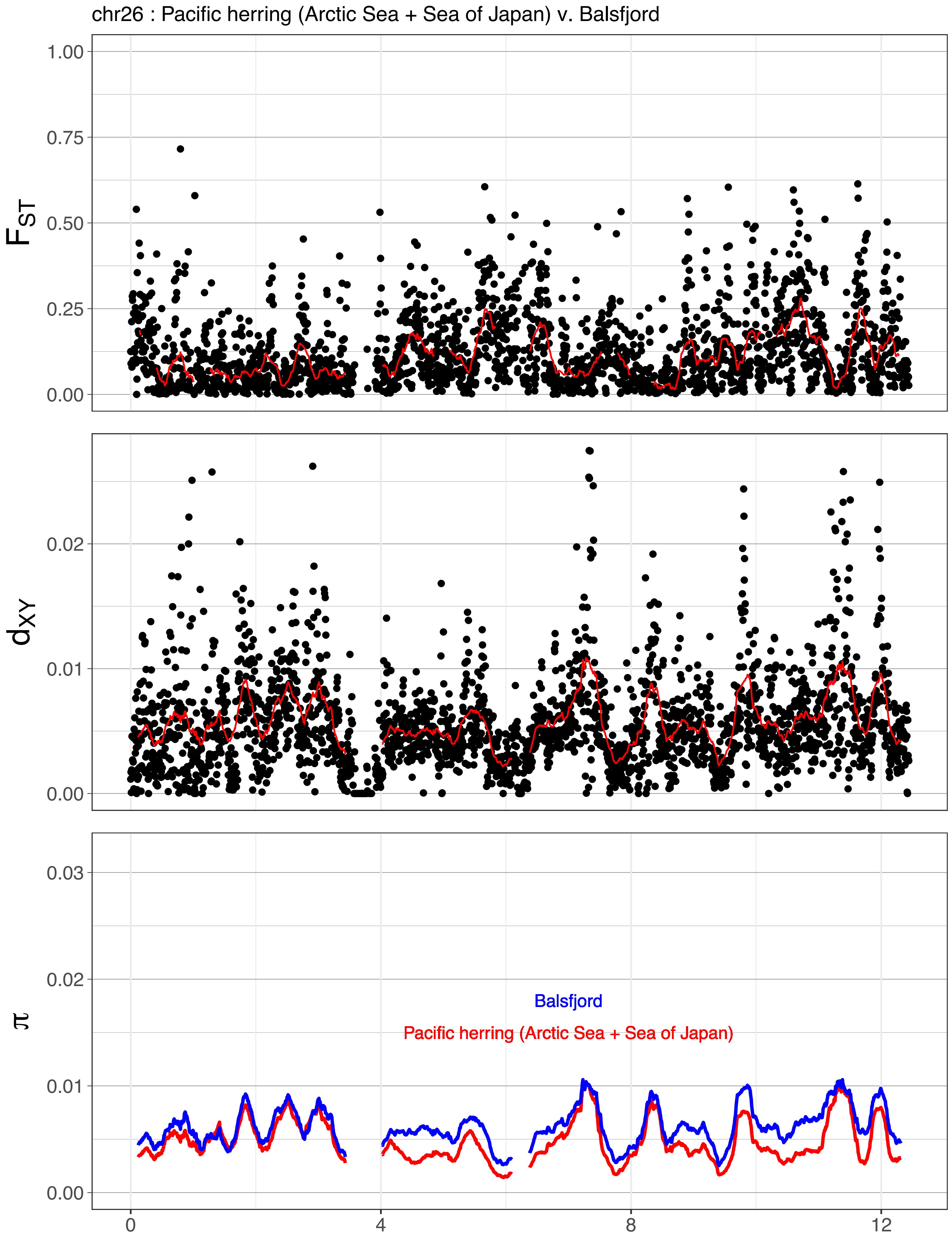
chr26 : Pacific herring (Arctic Sea + Sea of Japan) v. Balsfjord
F_{ST}
1.00
0.75
0.50
0.25
0.00
d_{XY}
0.02
0.01
0.00
π
0.03
0.02
0.01
0.00
Balsfjord
Pacific herring (Arctic Sea + Sea of Japan)
0
4
8
12
