# Supplementary material for: A Long-Standing Hybrid Population Between Pacific and Atlantic Herring in a Subarctic Fjord of Norway
Source: Genome Biol Evol. 2023 Apr 30;15(5):evad069. doi: 10.1093/gbe/evad069 (PMC10182735; doi:10.1093/gbe/evad069)
Supplement: evad069_Supplementary_Data [file evad069_supplementary_data.zip › Supplementary_Figure_9.pdf]

Supplementary Figure 9. Pool-seq frequencies at 8-fold Atlantic HSRs. Heatmaps showing reference allele frequencies (from Atlantic herring) for pooled samples in all 13 genomic regions where all Balsfjord individual haplotypes carry an HSR of Atlantic origin.

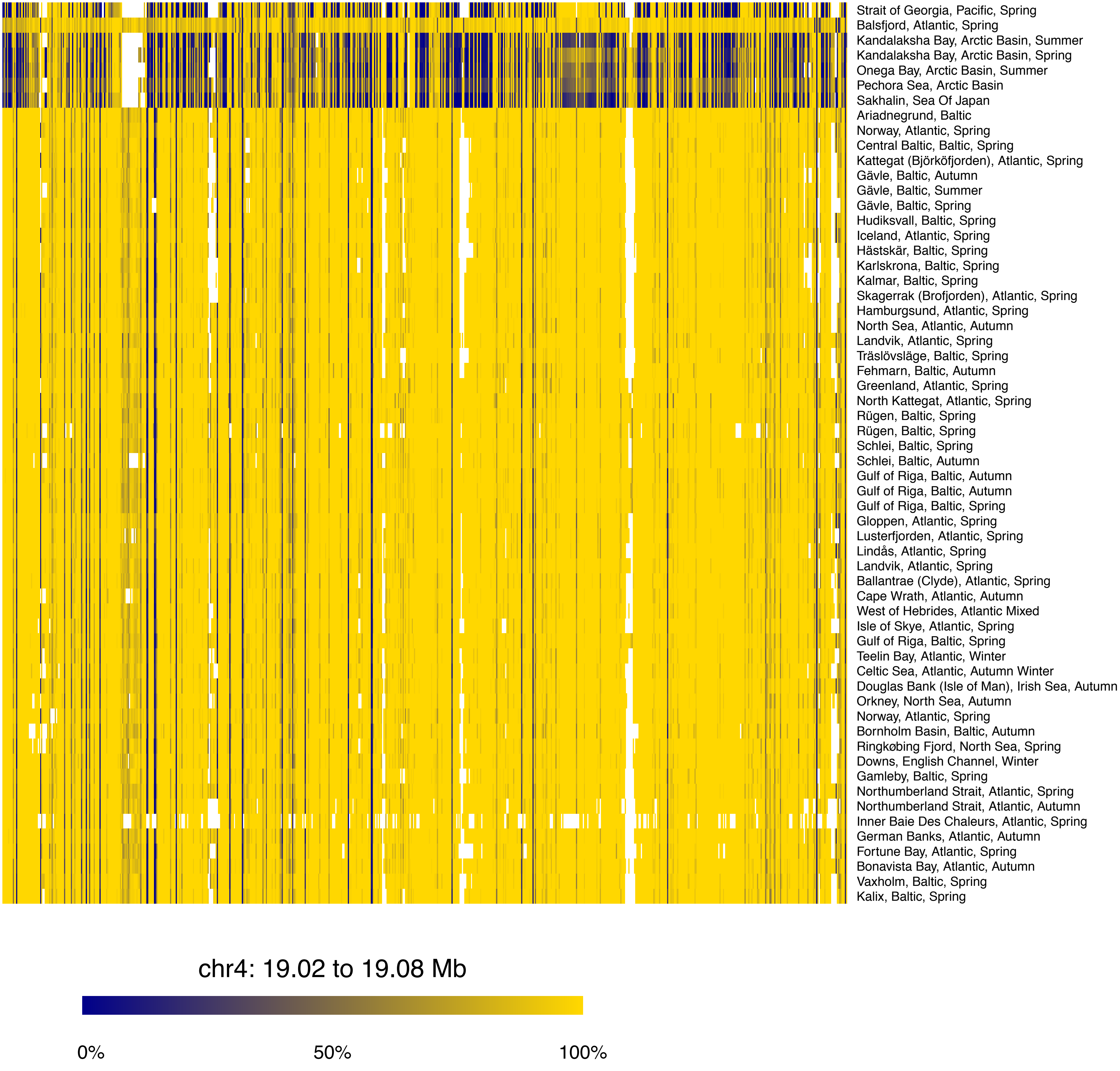
Strait of Georgia, Pacific, Spring
Balsfjord, Atlantic, Spring
Kandalaksha Bay, Arctic Basin, Summer
Kandalaksha Bay, Arctic Basin, Spring
Onega Bay, Arctic Basin, Summer
Pechora Sea, Arctic Basin
Sakhalin, Sea Of Japan
Ariadnegrund, Baltic
Norway, Atlantic, Spring
Central Baltic, Baltic, Spring
Kattegat (Björköfjorden), Atlantic, Spring
Gävle, Baltic, Autumn
Gävle, Baltic, Summer
Gävle, Baltic, Spring
Hudiksvall, Baltic, Spring
Iceland, Atlantic, Spring
Hästskär, Baltic, Spring
Karlskrona, Baltic, Spring
Kalmar, Baltic, Spring
Skagerrak (Brofjorden), Atlantic, Spring
Hamburgsund, Atlantic, Spring
North Sea, Atlantic, Autumn
Landvik, Atlantic, Spring
Träslövsläge, Baltic, Spring
Fehmarn, Baltic, Autumn
Greenland, Atlantic, Spring
North Kattegat, Atlantic, Spring
Rügen, Baltic, Spring
Rügen, Baltic, Spring
Schlei, Baltic, Spring
Schlei, Baltic, Autumn
Gulf of Riga, Baltic, Autumn
Gulf of Riga, Baltic, Autumn
Gulf of Riga, Baltic, Spring
Gloppen, Atlantic, Spring
Lusterfjorden, Atlantic, Spring
Lindås, Atlantic, Spring
Landvik, Atlantic, Spring
Ballantrae (Clyde), Atlantic, Spring
Cape Wrath, Atlantic, Autumn
West of Hebrides, Atlantic Mixed
Isle of Skye, Atlantic, Spring
Gulf of Riga, Baltic, Spring
Teelin Bay, Atlantic, Winter
Celtic Sea, Atlantic, Autumn Winter
Douglas Bank (Isle of Man), Irish Sea, Autumn
Orkney, North Sea, Autumn
Norway, Atlantic, Spring
Bornholm Basin, Baltic, Autumn
Ringkøbing Fjord, North Sea, Spring
Downs, English Channel, Winter
Gamleby, Baltic, Spring
Northumberland Strait, Atlantic, Spring
Northumberland Strait, Atlantic, Autumn
Inner Baie Des Chaleurs, Atlantic, Spring
German Banks, Atlantic, Autumn
Fortune Bay, Atlantic, Spring
Bonavista Bay, Atlantic, Autumn
Vaxholm, Baltic, Spring
Kalix, Baltic, Spring
chr4: 19.02 to 19.08 Mb
0%
50%
100%

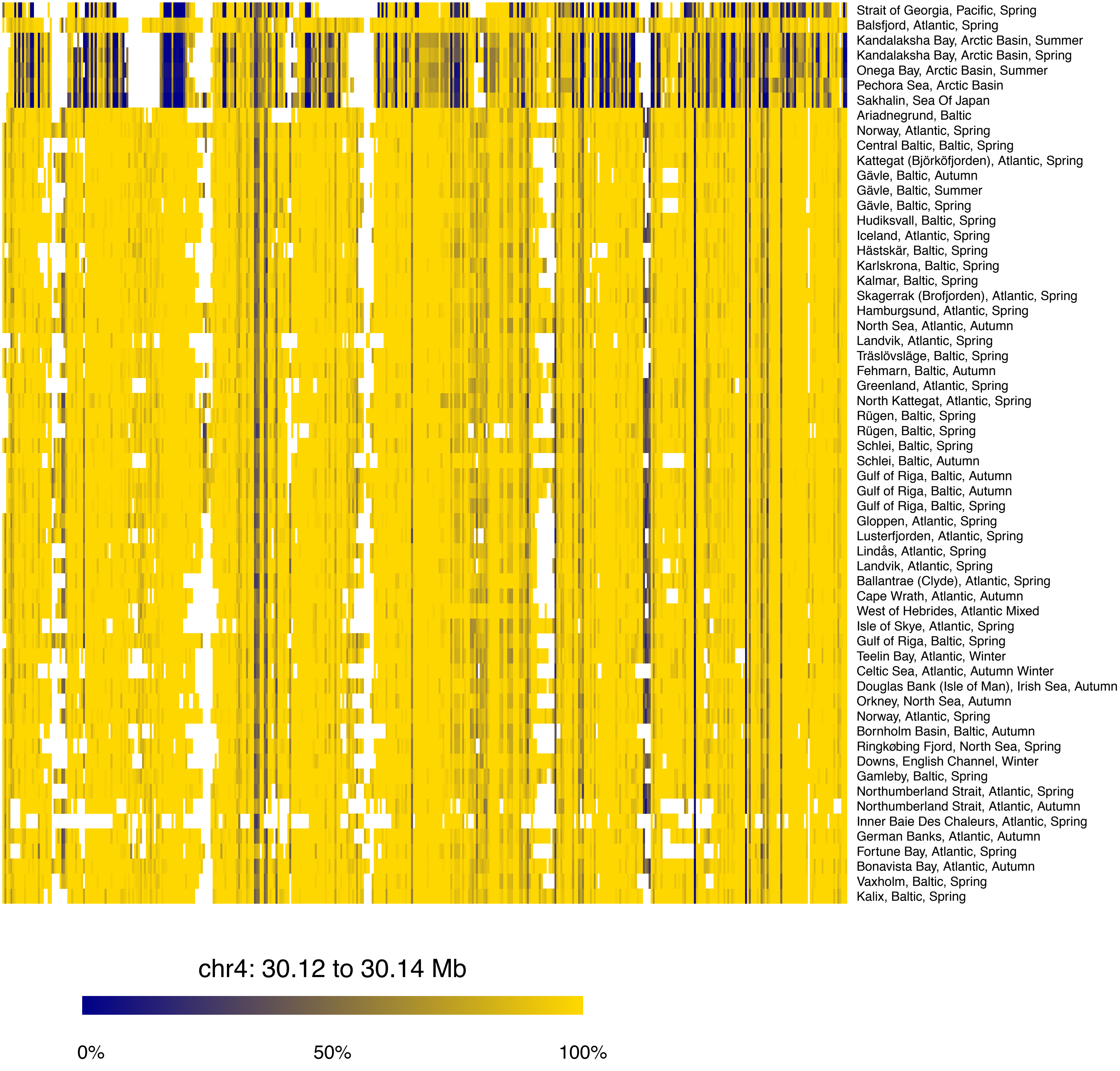
Strait of Georgia, Pacific, Spring
Balsfjord, Atlantic, Spring
Kandalaksha Bay, Arctic Basin, Summer
Kandalaksha Bay, Arctic Basin, Spring
Onega Bay, Arctic Basin, Summer
Pechora Sea, Arctic Basin
Sakhalin, Sea Of Japan
Ariadnegrund, Baltic
Norway, Atlantic, Spring
Central Baltic, Baltic, Spring
Kattegat (Björköfjorden), Atlantic, Spring
Gävle, Baltic, Autumn
Gävle, Baltic, Summer
Gävle, Baltic, Spring
Hudiksvall, Baltic, Spring
Iceland, Atlantic, Spring
Hästskär, Baltic, Spring
Karlskrona, Baltic, Spring
Kalmar, Baltic, Spring
Skagerrak (Brofjorden), Atlantic, Spring
Hamburgsund, Atlantic, Spring
North Sea, Atlantic, Autumn
Landvik, Atlantic, Spring
Träslövsläge, Baltic, Spring
Fehmarn, Baltic, Autumn
Greenland, Atlantic, Spring
North Kattegat, Atlantic, Spring
Rügen, Baltic, Spring
Rügen, Baltic, Spring
Schlei, Baltic, Spring
Schlei, Baltic, Autumn
Gulf of Riga, Baltic, Autumn
Gulf of Riga, Baltic, Autumn
Gulf of Riga, Baltic, Spring
Gloppen, Atlantic, Spring
Lusterfjorden, Atlantic, Spring
Lindås, Atlantic, Spring
Landvik, Atlantic, Spring
Ballantrae (Clyde), Atlantic, Spring
Cape Wrath, Atlantic, Autumn
West of Hebrides, Atlantic Mixed
Isle of Skye, Atlantic, Spring
Gulf of Riga, Baltic, Spring
Teelin Bay, Atlantic, Winter
Celtic Sea, Atlantic, Autumn Winter
Douglas Bank (Isle of Man), Irish Sea, Autumn
Orkney, North Sea, Autumn
Norway, Atlantic, Spring
Bornholm Basin, Baltic, Autumn
Ringkøbing Fjord, North Sea, Spring
Downs, English Channel, Winter
Gamleby, Baltic, Spring
Northumberland Strait, Atlantic, Spring
Northumberland Strait, Atlantic, Autumn
Inner Baie Des Chaleurs, Atlantic, Spring
German Banks, Atlantic, Autumn
Fortune Bay, Atlantic, Spring
Bonavista Bay, Atlantic, Autumn
Vaxholm, Baltic, Spring
Kalix, Baltic, Spring
chr4: 30.12 to 30.14 Mb
0%
50%
100%

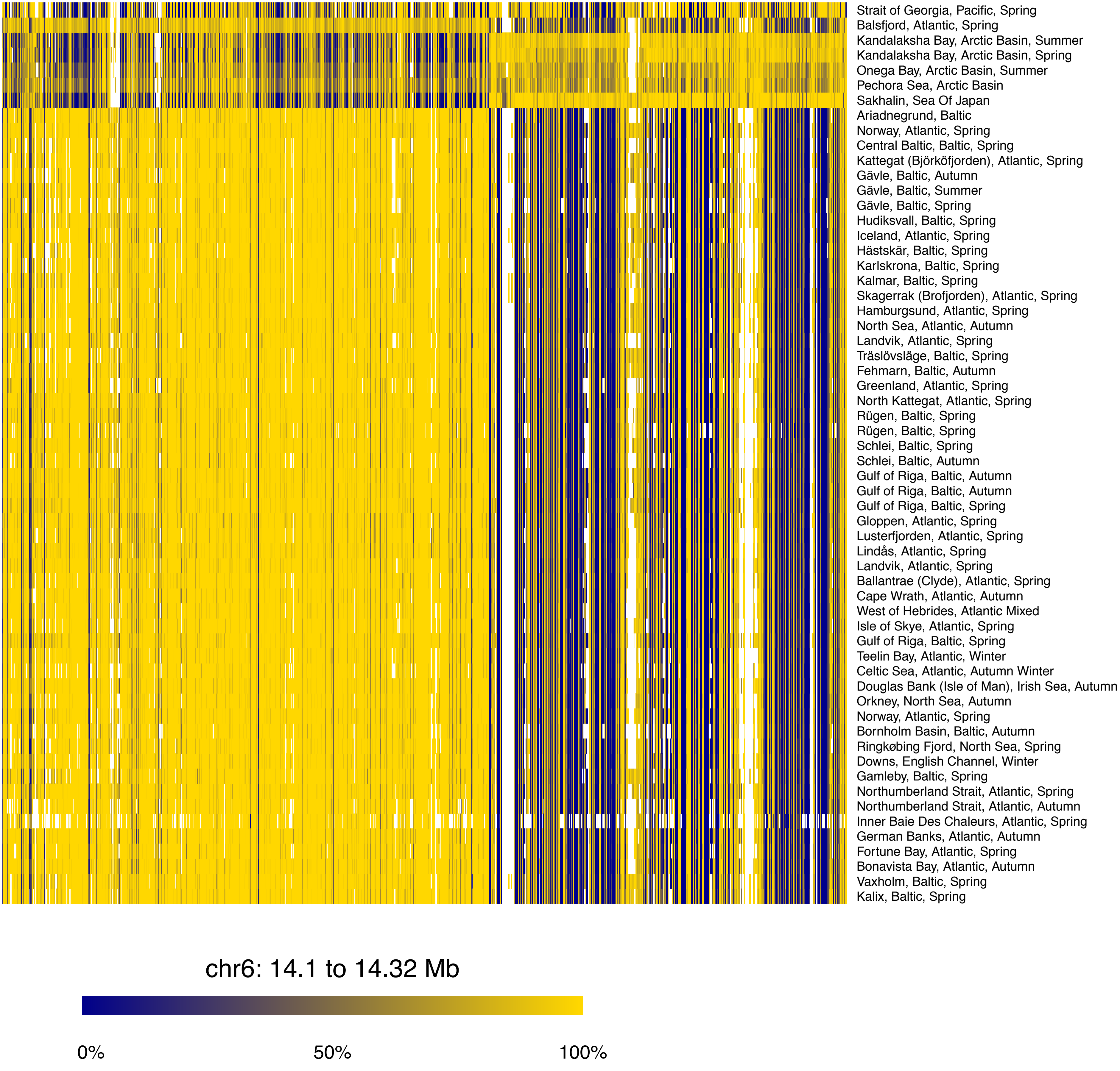
Strait of Georgia, Pacific, Spring
Balsfjord, Atlantic, Spring
Kandalaksha Bay, Arctic Basin, Summer
Kandalaksha Bay, Arctic Basin, Spring
Onega Bay, Arctic Basin, Summer
Pechora Sea, Arctic Basin
Sakhalin, Sea Of Japan
Ariadnegrund, Baltic
Norway, Atlantic, Spring
Central Baltic, Baltic, Spring
Kattegat (Björköfjorden), Atlantic, Spring
Gävle, Baltic, Autumn
Gävle, Baltic, Summer
Gävle, Baltic, Spring
Hudiksvall, Baltic, Spring
Iceland, Atlantic, Spring
Hästskär, Baltic, Spring
Karlskrona, Baltic, Spring
Kalmar, Baltic, Spring
Skagerrak (Brofjorden), Atlantic, Spring
Hamburgsund, Atlantic, Spring
North Sea, Atlantic, Autumn
Landvik, Atlantic, Spring
Träslövsläge, Baltic, Spring
Fehmarn, Baltic, Autumn
Greenland, Atlantic, Spring
North Kattegat, Atlantic, Spring
Rügen, Baltic, Spring
Rügen, Baltic, Spring
Schlei, Baltic, Spring
Schlei, Baltic, Autumn
Gulf of Riga, Baltic, Autumn
Gulf of Riga, Baltic, Autumn
Gulf of Riga, Baltic, Spring
Gloppen, Atlantic, Spring
Lusterfjorden, Atlantic, Spring
Lindås, Atlantic, Spring
Landvik, Atlantic, Spring
Ballantrae (Clyde), Atlantic, Spring
Cape Wrath, Atlantic, Autumn
West of Hebrides, Atlantic Mixed
Isle of Skye, Atlantic, Spring
Gulf of Riga, Baltic, Spring
Teelin Bay, Atlantic, Winter
Celtic Sea, Atlantic, Autumn Winter
Douglas Bank (Isle of Man), Irish Sea, Autumn
Orkney, North Sea, Autumn
Norway, Atlantic, Spring
Bornholm Basin, Baltic, Autumn
Ringkøbing Fjord, North Sea, Spring
Downs, English Channel, Winter
Gamleby, Baltic, Spring
Northumberland Strait, Atlantic, Spring
Northumberland Strait, Atlantic, Autumn
Inner Baie Des Chaleurs, Atlantic, Spring
German Banks, Atlantic, Autumn
Fortune Bay, Atlantic, Spring
Bonavista Bay, Atlantic, Autumn
Vaxholm, Baltic, Spring
Kalix, Baltic, Spring
chr6: 14.1 to 14.32 Mb
0%
50%
100%

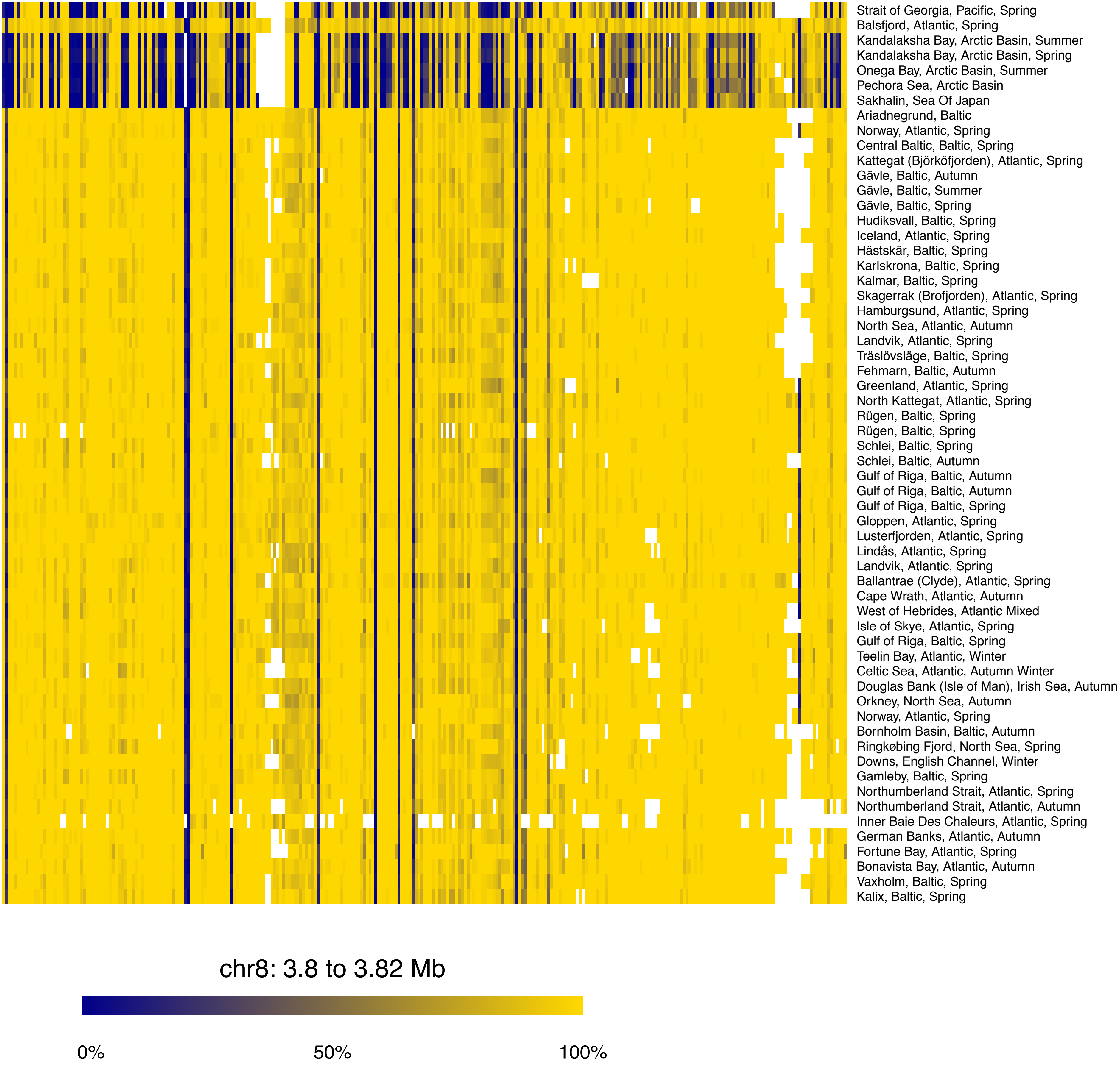
Strait of Georgia, Pacific, Spring
Balsfjord, Atlantic, Spring
Kandalaksha Bay, Arctic Basin, Summer
Kandalaksha Bay, Arctic Basin, Spring
Onega Bay, Arctic Basin, Summer
Pechora Sea, Arctic Basin
Sakhalin, Sea Of Japan
Ariadnegrund, Baltic
Norway, Atlantic, Spring
Central Baltic, Baltic, Spring
Kattegat (Björköfjorden), Atlantic, Spring
Gävle, Baltic, Autumn
Gävle, Baltic, Summer
Gävle, Baltic, Spring
Hudiksvall, Baltic, Spring
Iceland, Atlantic, Spring
Hästskär, Baltic, Spring
Karlskrona, Baltic, Spring
Kalmar, Baltic, Spring
Skagerrak (Brofjorden), Atlantic, Spring
Hamburgsund, Atlantic, Spring
North Sea, Atlantic, Autumn
Landvik, Atlantic, Spring
Träslövsläge, Baltic, Spring
Fehmarn, Baltic, Autumn
Greenland, Atlantic, Spring
North Kattegat, Atlantic, Spring
Rügen, Baltic, Spring
Rügen, Baltic, Spring
Schlei, Baltic, Spring
Schlei, Baltic, Autumn
Gulf of Riga, Baltic, Autumn
Gulf of Riga, Baltic, Autumn
Gulf of Riga, Baltic, Spring
Gloppen, Atlantic, Spring
Lusterfjorden, Atlantic, Spring
Lindås, Atlantic, Spring
Landvik, Atlantic, Spring
Ballantrae (Clyde), Atlantic, Spring
Cape Wrath, Atlantic, Autumn
West of Hebrides, Atlantic Mixed
Isle of Skye, Atlantic, Spring
Gulf of Riga, Baltic, Spring
Teelin Bay, Atlantic, Winter
Celtic Sea, Atlantic, Autumn Winter
Douglas Bank (Isle of Man), Irish Sea, Autumn
Orkney, North Sea, Autumn
Norway, Atlantic, Spring
Bornholm Basin, Baltic, Autumn
Ringkøbing Fjord, North Sea, Spring
Downs, English Channel, Winter
Gamleby, Baltic, Spring
Northumberland Strait, Atlantic, Spring
Northumberland Strait, Atlantic, Autumn
Inner Baie Des Chaleurs, Atlantic, Spring
German Banks, Atlantic, Autumn
Fortune Bay, Atlantic, Spring
Bonavista Bay, Atlantic, Autumn
Vaxholm, Baltic, Spring
Kalix, Baltic, Spring
chr8: 3.8 to 3.82 Mb
0%
50%
100%

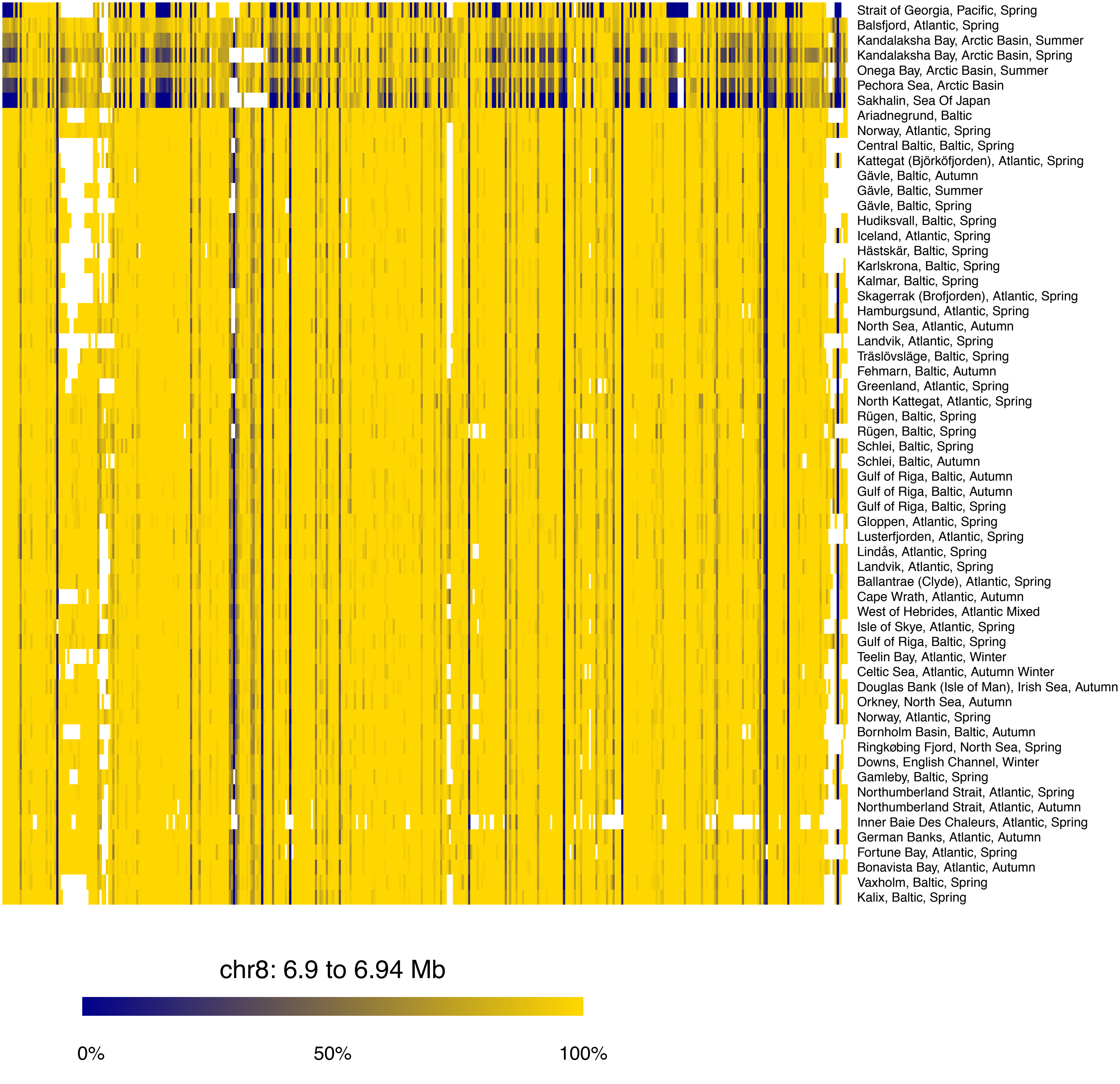
Strait of Georgia, Pacific, Spring
Balsfjord, Atlantic, Spring
Kandalaksha Bay, Arctic Basin, Summer
Kandalaksha Bay, Arctic Basin, Spring
Onega Bay, Arctic Basin, Summer
Pechora Sea, Arctic Basin
Sakhalin, Sea Of Japan
Ariadnegrund, Baltic
Norway, Atlantic, Spring
Central Baltic, Baltic, Spring
Kattegat (Björköfjorden), Atlantic, Spring
Gävle, Baltic, Autumn
Gävle, Baltic, Summer
Gävle, Baltic, Spring
Hudiksvall, Baltic, Spring
Iceland, Atlantic, Spring
Hästskär, Baltic, Spring
Karlskrona, Baltic, Spring
Kalmar, Baltic, Spring
Skagerrak (Brofjorden), Atlantic, Spring
Hamburgsund, Atlantic, Spring
North Sea, Atlantic, Autumn
Landvik, Atlantic, Spring
Träslövsläge, Baltic, Spring
Fehmarn, Baltic, Autumn
Greenland, Atlantic, Spring
North Kattegat, Atlantic, Spring
Rügen, Baltic, Spring
Rügen, Baltic, Spring
Schlei, Baltic, Spring
Schlei, Baltic, Autumn
Gulf of Riga, Baltic, Autumn
Gulf of Riga, Baltic, Autumn
Gulf of Riga, Baltic, Spring
Gloppen, Atlantic, Spring
Lusterfjorden, Atlantic, Spring
Lindås, Atlantic, Spring
Landvik, Atlantic, Spring
Ballantrae (Clyde), Atlantic, Spring
Cape Wrath, Atlantic, Autumn
West of Hebrides, Atlantic Mixed
Isle of Skye, Atlantic, Spring
Gulf of Riga, Baltic, Spring
Teelin Bay, Atlantic, Winter
Celtic Sea, Atlantic, Autumn Winter
Douglas Bank (Isle of Man), Irish Sea, Autumn
Orkney, North Sea, Autumn
Norway, Atlantic, Spring
Bornholm Basin, Baltic, Autumn
Ringkøbing Fjord, North Sea, Spring
Downs, English Channel, Winter
Gamleby, Baltic, Spring
Northumberland Strait, Atlantic, Spring
Northumberland Strait, Atlantic, Autumn
Inner Baie Des Chaleurs, Atlantic, Spring
German Banks, Atlantic, Autumn
Fortune Bay, Atlantic, Spring
Bonavista Bay, Atlantic, Autumn
Vaxholm, Baltic, Spring
Kalix, Baltic, Spring
chr8: 6.9 to 6.94 Mb
0%
50%
100%

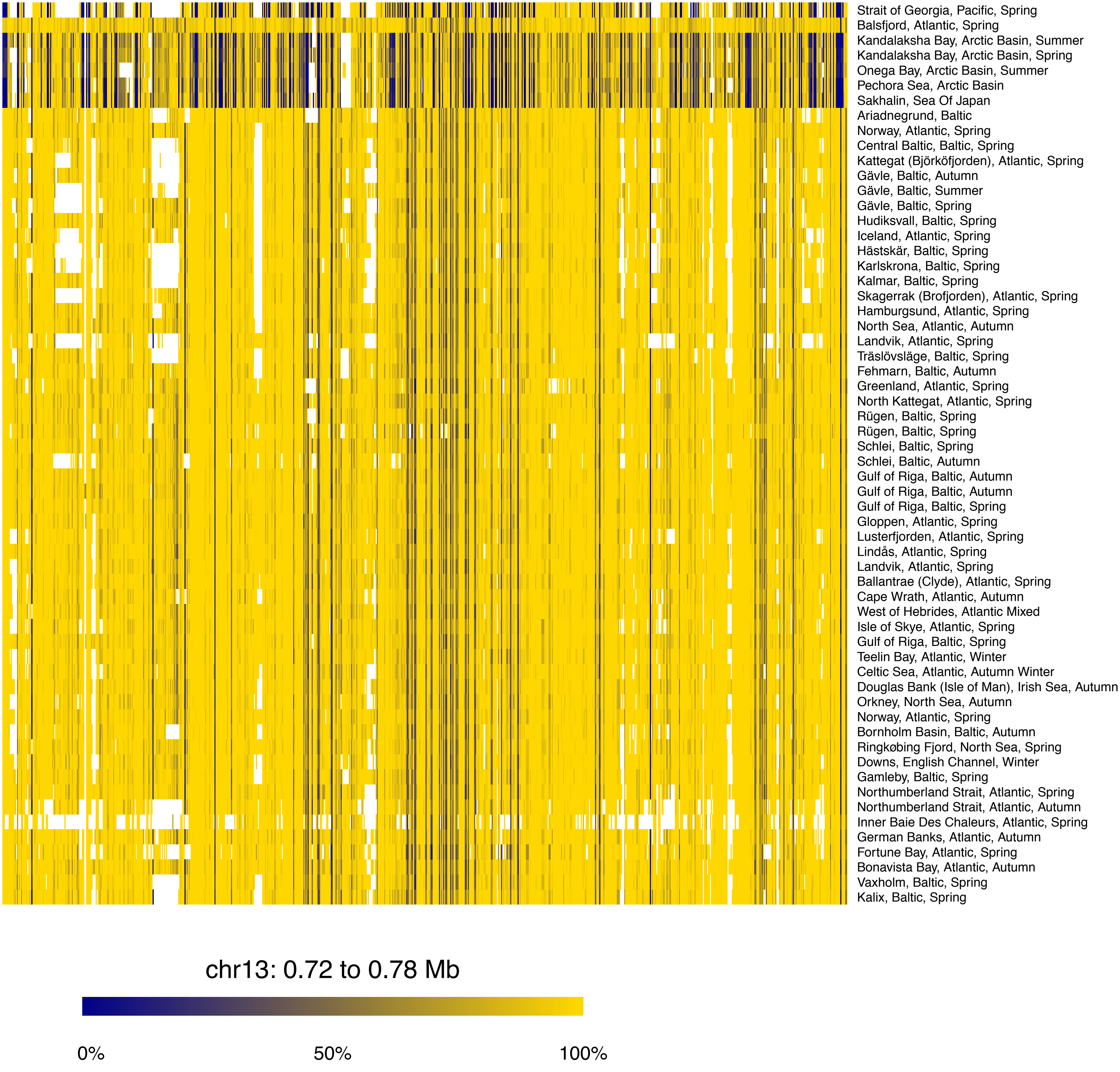
Strait of Georgia, Pacific, Spring
Balsfjord, Atlantic, Spring
Kandalaksha Bay, Arctic Basin, Summer
Kandalaksha Bay, Arctic Basin, Spring
Onega Bay, Arctic Basin, Summer
Pechora Sea, Arctic Basin
Sakhalin, Sea Of Japan
Ariadnegrund, Baltic
Norway, Atlantic, Spring
Central Baltic, Baltic, Spring
Kattegat (Björköfjorden), Atlantic, Spring
Gävle, Baltic, Autumn
Gävle, Baltic, Summer
Gävle, Baltic, Spring
Hudiksvall, Baltic, Spring
Iceland, Atlantic, Spring
Hästskär, Baltic, Spring
Karlskrona, Baltic, Spring
Kalmar, Baltic, Spring
Skagerrak (Brofjorden), Atlantic, Spring
Hamburgsund, Atlantic, Spring
North Sea, Atlantic, Autumn
Landvik, Atlantic, Spring
Träslövsläge, Baltic, Spring
Fehmarn, Baltic, Autumn
Greenland, Atlantic, Spring
North Kattegat, Atlantic, Spring
Rügen, Baltic, Spring
Rügen, Baltic, Spring
Schlei, Baltic, Spring
Schlei, Baltic, Autumn
Gulf of Riga, Baltic, Autumn
Gulf of Riga, Baltic, Autumn
Gulf of Riga, Baltic, Spring
Gloppen, Atlantic, Spring
Lusterfjorden, Atlantic, Spring
Lindås, Atlantic, Spring
Landvik, Atlantic, Spring
Ballantrae (Clyde), Atlantic, Spring
Cape Wrath, Atlantic, Autumn
West of Hebrides, Atlantic Mixed
Isle of Skye, Atlantic, Spring
Gulf of Riga, Baltic, Spring
Teelin Bay, Atlantic, Winter
Celtic Sea, Atlantic, Autumn Winter
Douglas Bank (Isle of Man), Irish Sea, Autumn
Orkney, North Sea, Autumn
Norway, Atlantic, Spring
Bornholm Basin, Baltic, Autumn
Ringkøbing Fjord, North Sea, Spring
Downs, English Channel, Winter
Gamleby, Baltic, Spring
Northumberland Strait, Atlantic, Spring
Northumberland Strait, Atlantic, Autumn
Inner Baie Des Chaleurs, Atlantic, Spring
German Banks, Atlantic, Autumn
Fortune Bay, Atlantic, Spring
Bonavista Bay, Atlantic, Autumn
Vaxholm, Baltic, Spring
Kalix, Baltic, Spring
chr13: 0.72 to 0.78 Mb
0%
50%
100%

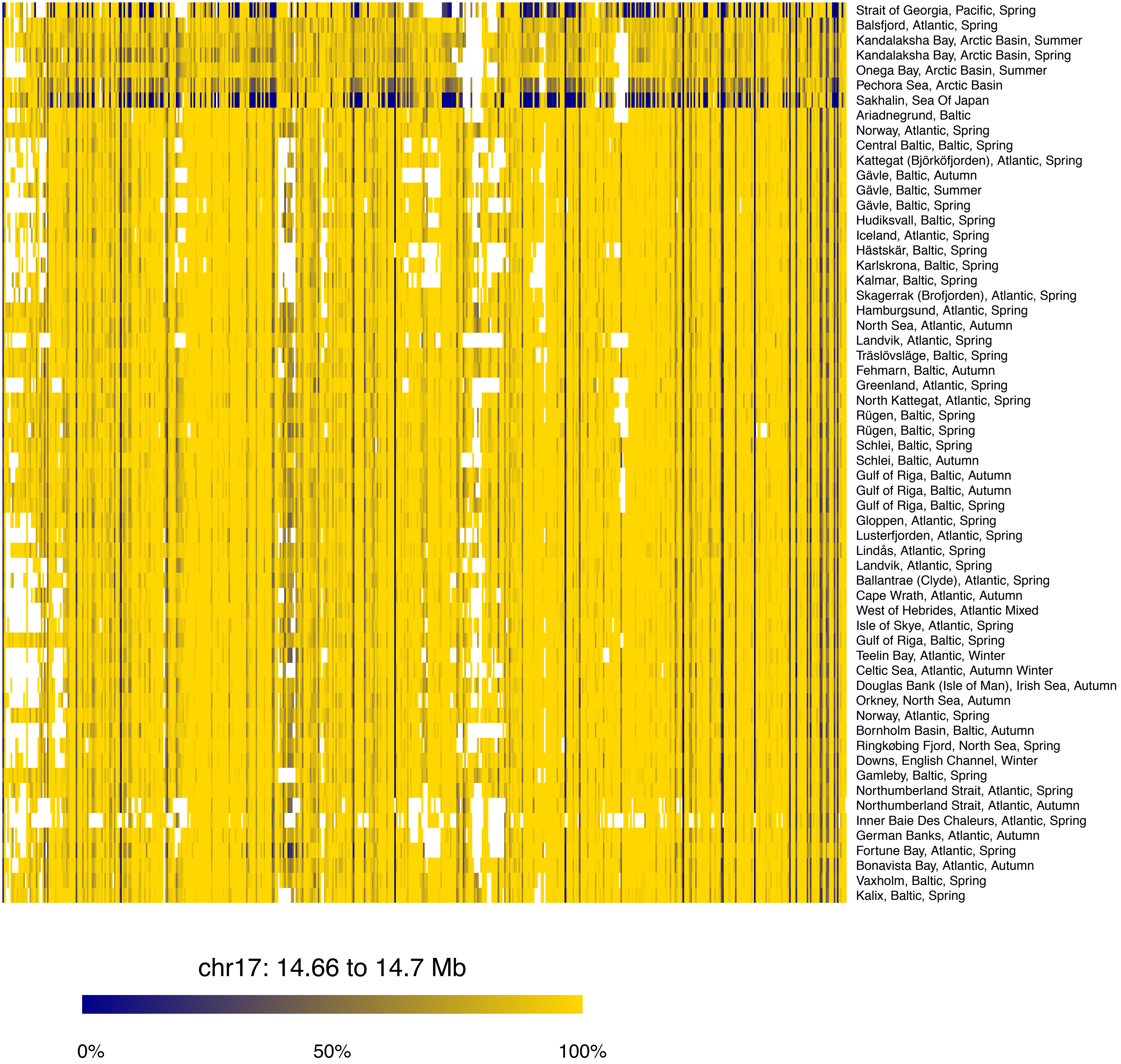
Strait of Georgia, Pacific, Spring
Balsfjord, Atlantic, Spring
Kandalaksha Bay, Arctic Basin, Summer
Kandalaksha Bay, Arctic Basin, Spring
Onega Bay, Arctic Basin, Summer
Pechora Sea, Arctic Basin
Sakhalin, Sea Of Japan
Ariadnegrund, Baltic
Norway, Atlantic, Spring
Central Baltic, Baltic, Spring
Kattegat (Björköfjorden), Atlantic, Spring
Gävle, Baltic, Autumn
Gävle, Baltic, Summer
Gävle, Baltic, Spring
Hudiksvall, Baltic, Spring
Iceland, Atlantic, Spring
Hästskär, Baltic, Spring
Karlskrona, Baltic, Spring
Kalmar, Baltic, Spring
Skagerrak (Brofjorden), Atlantic, Spring
Hamburgsund, Atlantic, Spring
North Sea, Atlantic, Autumn
Landvik, Atlantic, Spring
Träslövsläge, Baltic, Spring
Fehmarn, Baltic, Autumn
Greenland, Atlantic, Spring
North Kattegat, Atlantic, Spring
Rügen, Baltic, Spring
Rügen, Baltic, Spring
Schlei, Baltic, Spring
Schlei, Baltic, Autumn
Gulf of Riga, Baltic, Autumn
Gulf of Riga, Baltic, Autumn
Gulf of Riga, Baltic, Spring
Gloppen, Atlantic, Spring
Lusterfjorden, Atlantic, Spring
Lindås, Atlantic, Spring
Landvik, Atlantic, Spring
Ballantrae (Clyde), Atlantic, Spring
Cape Wrath, Atlantic, Autumn
West of Hebrides, Atlantic Mixed
Isle of Skye, Atlantic, Spring
Gulf of Riga, Baltic, Spring
Teelin Bay, Atlantic, Winter
Celtic Sea, Atlantic, Autumn Winter
Douglas Bank (Isle of Man), Irish Sea, Autumn
Orkney, North Sea, Autumn
Norway, Atlantic, Spring
Bornholm Basin, Baltic, Autumn
Ringkøbing Fjord, North Sea, Spring
Downs, English Channel, Winter
Gamleby, Baltic, Spring
Northumberland Strait, Atlantic, Spring
Northumberland Strait, Atlantic, Autumn
Inner Baie Des Chaleurs, Atlantic, Spring
German Banks, Atlantic, Autumn
Fortune Bay, Atlantic, Spring
Bonavista Bay, Atlantic, Autumn
Vaxholm, Baltic, Spring
Kalix, Baltic, Spring
chr17: 14.66 to 14.7 Mb
0%
50%
100%

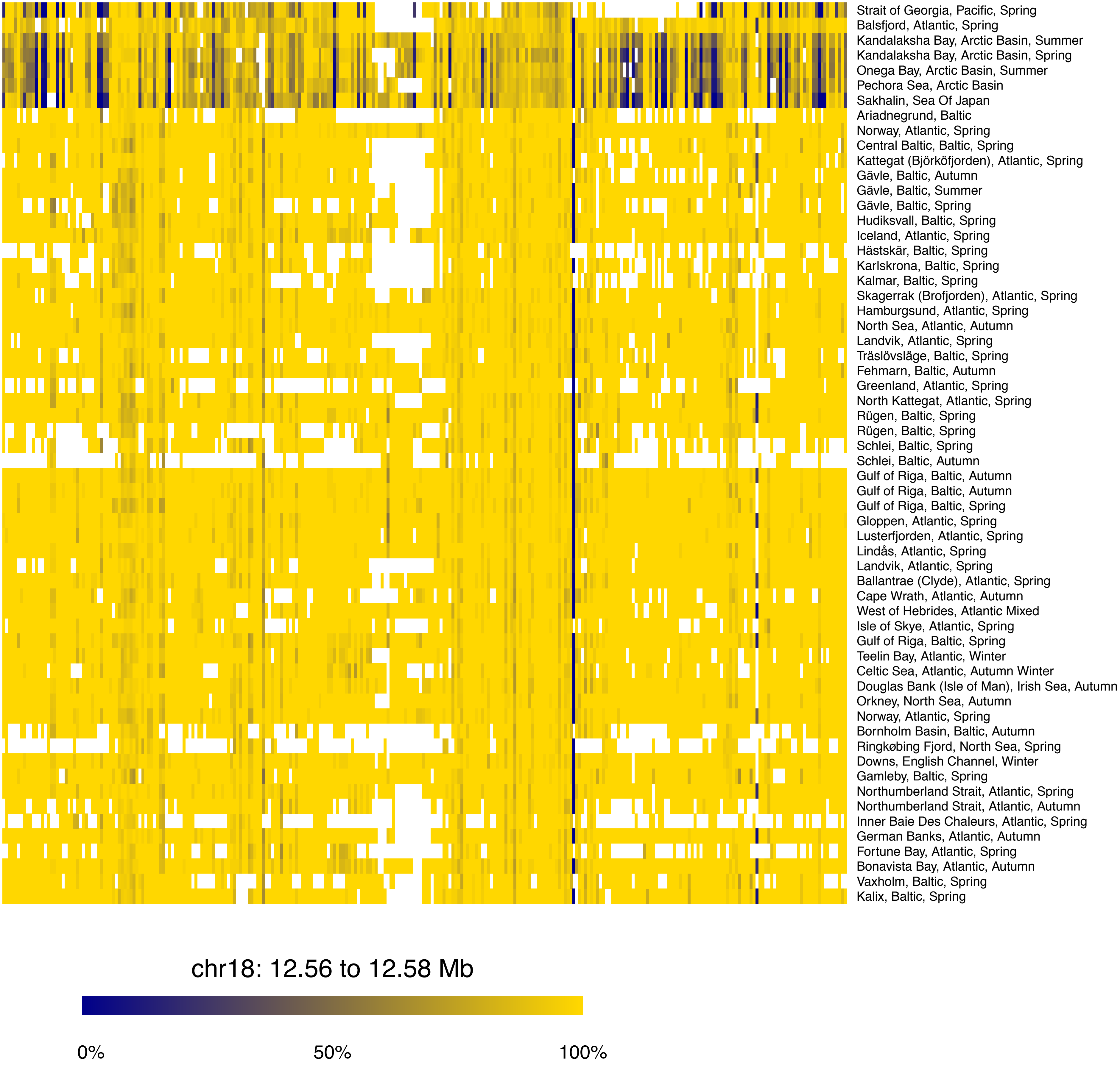
Strait of Georgia, Pacific, Spring
Balsfjord, Atlantic, Spring
Kandalaksha Bay, Arctic Basin, Summer
Kandalaksha Bay, Arctic Basin, Spring
Onega Bay, Arctic Basin, Summer
Pechora Sea, Arctic Basin
Sakhalin, Sea Of Japan
Ariadnegrund, Baltic
Norway, Atlantic, Spring
Central Baltic, Baltic, Spring
Kattegat (Björköfjorden), Atlantic, Spring
Gävle, Baltic, Autumn
Gävle, Baltic, Summer
Gävle, Baltic, Spring
Hudiksvall, Baltic, Spring
Iceland, Atlantic, Spring
Hästskär, Baltic, Spring
Karlskrona, Baltic, Spring
Kalmar, Baltic, Spring
Skagerrak (Brofjorden), Atlantic, Spring
Hamburgsund, Atlantic, Spring
North Sea, Atlantic, Autumn
Landvik, Atlantic, Spring
Träslövsläge, Baltic, Spring
Fehmarn, Baltic, Autumn
Greenland, Atlantic, Spring
North Kattegat, Atlantic, Spring
Rügen, Baltic, Spring
Rügen, Baltic, Spring
Schlei, Baltic, Spring
Schlei, Baltic, Autumn
Gulf of Riga, Baltic, Autumn
Gulf of Riga, Baltic, Autumn
Gulf of Riga, Baltic, Spring
Gloppen, Atlantic, Spring
Lusterfjorden, Atlantic, Spring
Lindås, Atlantic, Spring
Landvik, Atlantic, Spring
Ballantrae (Clyde), Atlantic, Spring
Cape Wrath, Atlantic, Autumn
West of Hebrides, Atlantic Mixed
Isle of Skye, Atlantic, Spring
Gulf of Riga, Baltic, Spring
Teelin Bay, Atlantic, Winter
Celtic Sea, Atlantic, Autumn Winter
Douglas Bank (Isle of Man), Irish Sea, Autumn
Orkney, North Sea, Autumn
Norway, Atlantic, Spring
Bornholm Basin, Baltic, Autumn
Ringkøbing Fjord, North Sea, Spring
Downs, English Channel, Winter
Gamleby, Baltic, Spring
Northumberland Strait, Atlantic, Spring
Northumberland Strait, Atlantic, Autumn
Inner Baie Des Chaleurs, Atlantic, Spring
German Banks, Atlantic, Autumn
Fortune Bay, Atlantic, Spring
Bonavista Bay, Atlantic, Autumn
Vaxholm, Baltic, Spring
Kalix, Baltic, Spring
chr18: 12.56 to 12.58 Mb
0%
50%
100%

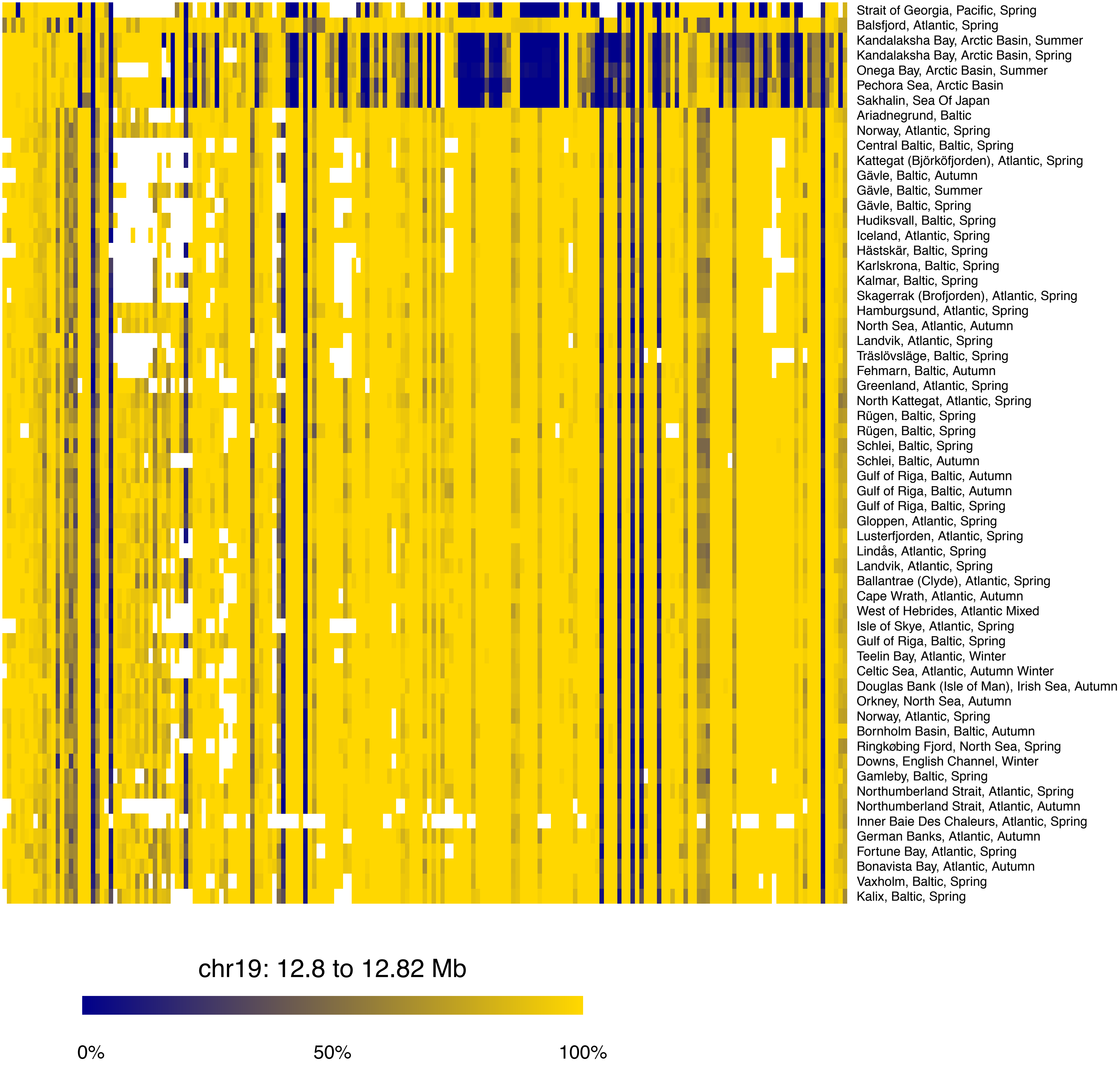
Strait of Georgia, Pacific, Spring
Balsfjord, Atlantic, Spring
Kandalaksha Bay, Arctic Basin, Summer
Kandalaksha Bay, Arctic Basin, Spring
Onega Bay, Arctic Basin, Summer
Pechora Sea, Arctic Basin
Sakhalin, Sea Of Japan
Ariadnegrund, Baltic
Norway, Atlantic, Spring
Central Baltic, Baltic, Spring
Kattegat (Björköfjorden), Atlantic, Spring
Gävle, Baltic, Autumn
Gävle, Baltic, Summer
Gävle, Baltic, Spring
Hudiksvall, Baltic, Spring
Iceland, Atlantic, Spring
Hästskär, Baltic, Spring
Karlskrona, Baltic, Spring
Kalmar, Baltic, Spring
Skagerrak (Brofjorden), Atlantic, Spring
Hamburgsund, Atlantic, Spring
North Sea, Atlantic, Autumn
Landvik, Atlantic, Spring
Träslövsläge, Baltic, Spring
Fehmarn, Baltic, Autumn
Greenland, Atlantic, Spring
North Kattegat, Atlantic, Spring
Rügen, Baltic, Spring
Rügen, Baltic, Spring
Schlei, Baltic, Spring
Schlei, Baltic, Autumn
Gulf of Riga, Baltic, Autumn
Gulf of Riga, Baltic, Autumn
Gulf of Riga, Baltic, Spring
Gloppen, Atlantic, Spring
Lusterfjorden, Atlantic, Spring
Lindås, Atlantic, Spring
Landvik, Atlantic, Spring
Ballantrae (Clyde), Atlantic, Spring
Cape Wrath, Atlantic, Autumn
West of Hebrides, Atlantic Mixed
Isle of Skye, Atlantic, Spring
Gulf of Riga, Baltic, Spring
Teelin Bay, Atlantic, Winter
Celtic Sea, Atlantic, Autumn Winter
Douglas Bank (Isle of Man), Irish Sea, Autumn
Orkney, North Sea, Autumn
Norway, Atlantic, Spring
Bornholm Basin, Baltic, Autumn
Ringkøbing Fjord, North Sea, Spring
Downs, English Channel, Winter
Gamleby, Baltic, Spring
Northumberland Strait, Atlantic, Spring
Northumberland Strait, Atlantic, Autumn
Inner Baie Des Chaleurs, Atlantic, Spring
German Banks, Atlantic, Autumn
Fortune Bay, Atlantic, Spring
Bonavista Bay, Atlantic, Autumn
Vaxholm, Baltic, Spring
Kalix, Baltic, Spring
chr19: 12.8 to 12.82 Mb
0%
50%
100%

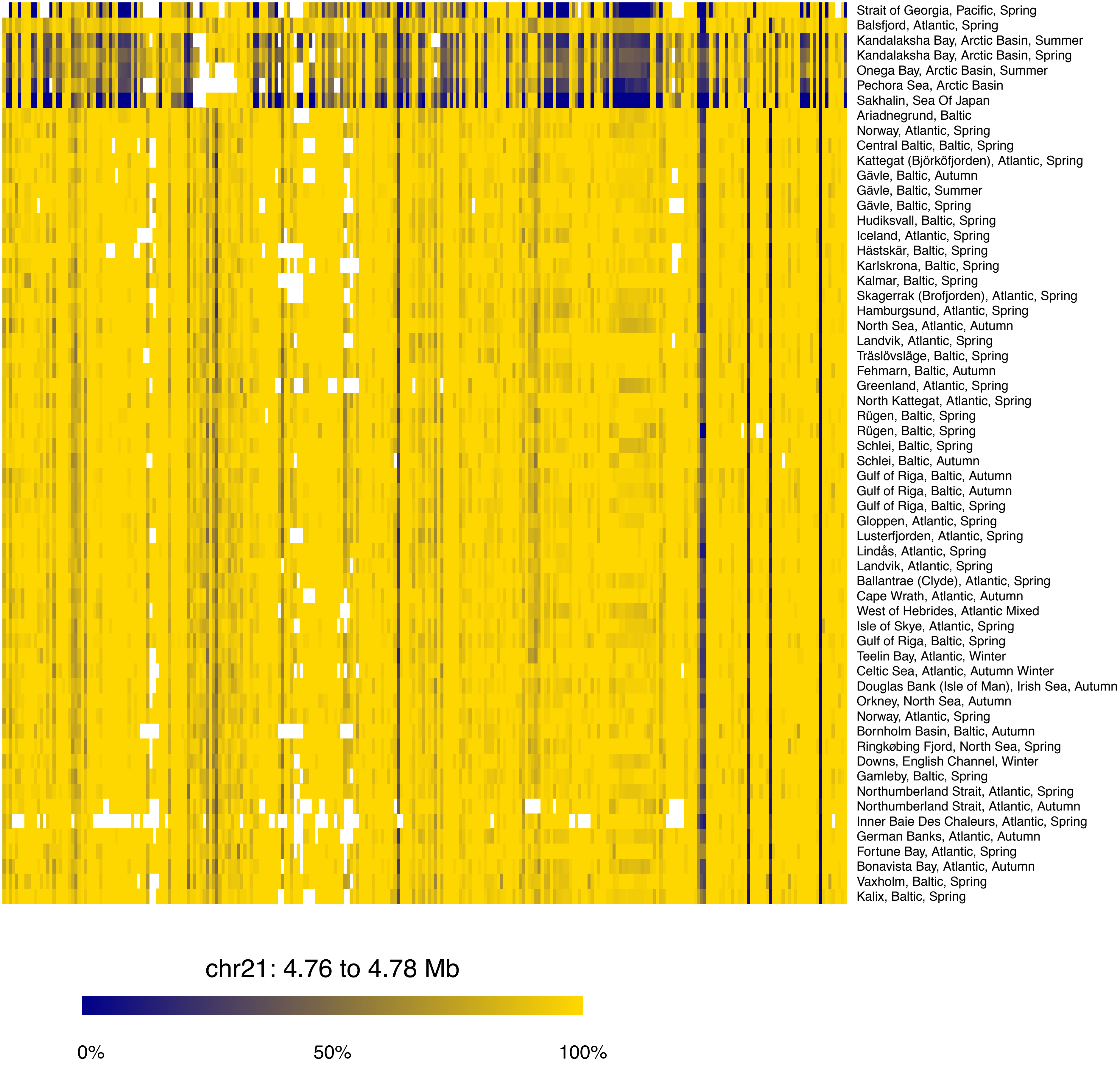
Strait of Georgia, Pacific, Spring
Balsfjord, Atlantic, Spring
Kandalaksha Bay, Arctic Basin, Summer
Kandalaksha Bay, Arctic Basin, Spring
Onega Bay, Arctic Basin, Summer
Pechora Sea, Arctic Basin
Sakhalin, Sea Of Japan
Ariadnegrund, Baltic
Norway, Atlantic, Spring
Central Baltic, Baltic, Spring
Kattegat (Björköfjorden), Atlantic, Spring
Gävle, Baltic, Autumn
Gävle, Baltic, Summer
Gävle, Baltic, Spring
Hudiksvall, Baltic, Spring
Iceland, Atlantic, Spring
Hästskär, Baltic, Spring
Karlskrona, Baltic, Spring
Kalmar, Baltic, Spring
Skagerrak (Brofjorden), Atlantic, Spring
Hamburgsund, Atlantic, Spring
North Sea, Atlantic, Autumn
Landvik, Atlantic, Spring
Träslövsläge, Baltic, Spring
Fehmarn, Baltic, Autumn
Greenland, Atlantic, Spring
North Kattegat, Atlantic, Spring
Rügen, Baltic, Spring
Rügen, Baltic, Spring
Schlei, Baltic, Spring
Schlei, Baltic, Autumn
Gulf of Riga, Baltic, Autumn
Gulf of Riga, Baltic, Autumn
Gulf of Riga, Baltic, Spring
Gloppen, Atlantic, Spring
Lusterfjorden, Atlantic, Spring
Lindås, Atlantic, Spring
Landvik, Atlantic, Spring
Ballantrae (Clyde), Atlantic, Spring
Cape Wrath, Atlantic, Autumn
West of Hebrides, Atlantic Mixed
Isle of Skye, Atlantic, Spring
Gulf of Riga, Baltic, Spring
Teelin Bay, Atlantic, Winter
Celtic Sea, Atlantic, Autumn Winter
Douglas Bank (Isle of Man), Irish Sea, Autumn
Orkney, North Sea, Autumn
Norway, Atlantic, Spring
Bornholm Basin, Baltic, Autumn
Ringkøbing Fjord, North Sea, Spring
Downs, English Channel, Winter
Gamleby, Baltic, Spring
Northumberland Strait, Atlantic, Spring
Northumberland Strait, Atlantic, Autumn
Inner Baie Des Chaleurs, Atlantic, Spring
German Banks, Atlantic, Autumn
Fortune Bay, Atlantic, Spring
Bonavista Bay, Atlantic, Autumn
Vaxholm, Baltic, Spring
Kalix, Baltic, Spring
chr21: 4.76 to 4.78 Mb
0%
50%
100%

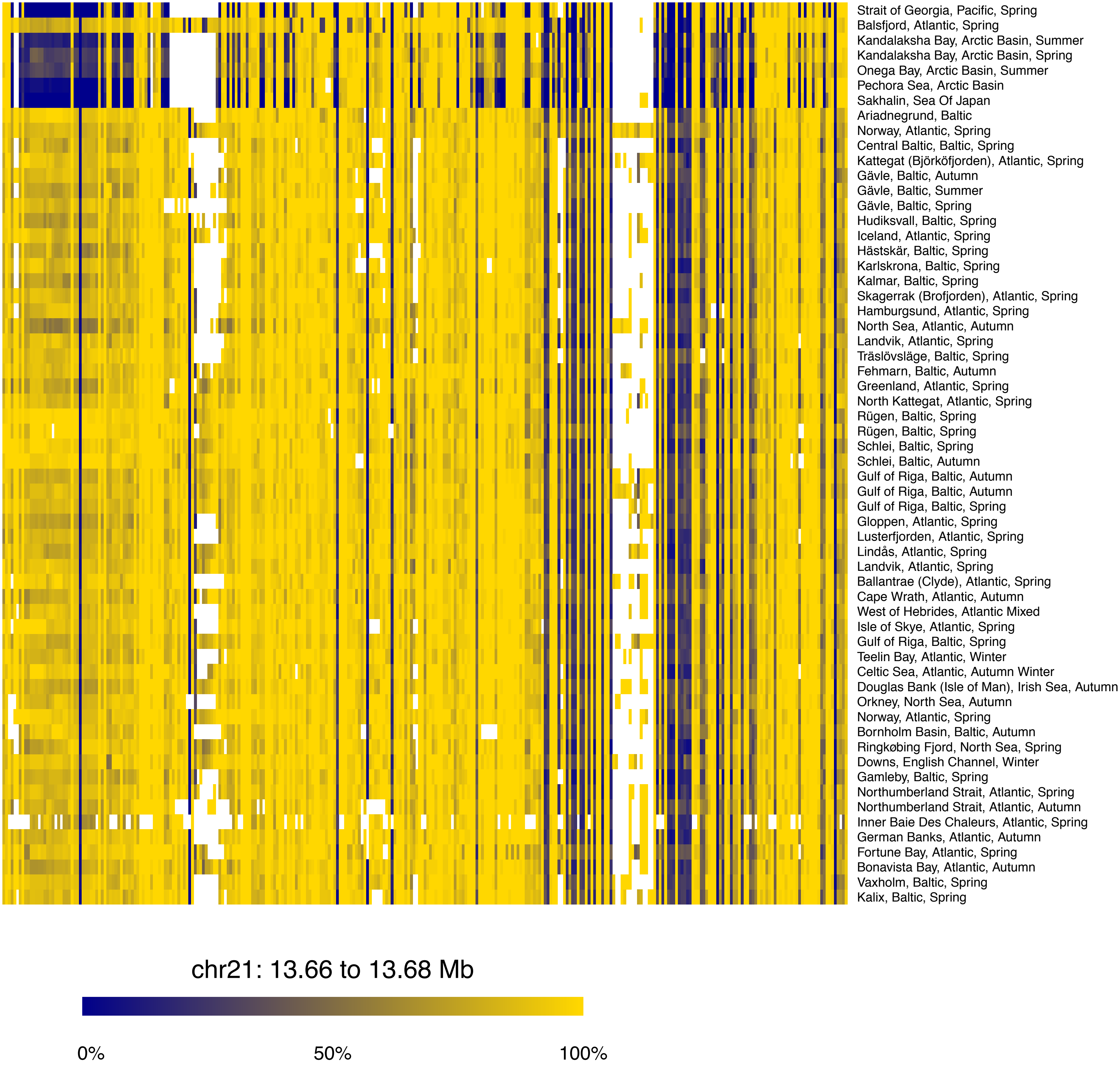
Strait of Georgia, Pacific, Spring
Balsfjord, Atlantic, Spring
Kandalaksha Bay, Arctic Basin, Summer
Kandalaksha Bay, Arctic Basin, Spring
Onega Bay, Arctic Basin, Summer
Pechora Sea, Arctic Basin
Sakhalin, Sea Of Japan
Ariadnegrund, Baltic
Norway, Atlantic, Spring
Central Baltic, Baltic, Spring
Kattegat (Björköfjorden), Atlantic, Spring
Gävle, Baltic, Autumn
Gävle, Baltic, Summer
Gävle, Baltic, Spring
Hudiksvall, Baltic, Spring
Iceland, Atlantic, Spring
Hästskär, Baltic, Spring
Karlskrona, Baltic, Spring
Kalmar, Baltic, Spring
Skagerrak (Brofjorden), Atlantic, Spring
Hamburgsund, Atlantic, Spring
North Sea, Atlantic, Autumn
Landvik, Atlantic, Spring
Träslövsläge, Baltic, Spring
Fehmarn, Baltic, Autumn
Greenland, Atlantic, Spring
North Kattegat, Atlantic, Spring
Rügen, Baltic, Spring
Rügen, Baltic, Spring
Schlei, Baltic, Spring
Schlei, Baltic, Autumn
Gulf of Riga, Baltic, Autumn
Gulf of Riga, Baltic, Autumn
Gulf of Riga, Baltic, Spring
Gloppen, Atlantic, Spring
Lusterfjorden, Atlantic, Spring
Lindås, Atlantic, Spring
Landvik, Atlantic, Spring
Ballantrae (Clyde), Atlantic, Spring
Cape Wrath, Atlantic, Autumn
West of Hebrides, Atlantic Mixed
Isle of Skye, Atlantic, Spring
Gulf of Riga, Baltic, Spring
Teelin Bay, Atlantic, Winter
Celtic Sea, Atlantic, Autumn Winter
Douglas Bank (Isle of Man), Irish Sea, Autumn
Orkney, North Sea, Autumn
Norway, Atlantic, Spring
Bornholm Basin, Baltic, Autumn
Ringkøbing Fjord, North Sea, Spring
Downs, English Channel, Winter
Gamleby, Baltic, Spring
Northumberland Strait, Atlantic, Spring
Northumberland Strait, Atlantic, Autumn
Inner Baie Des Chaleurs, Atlantic, Spring
German Banks, Atlantic, Autumn
Fortune Bay, Atlantic, Spring
Bonavista Bay, Atlantic, Autumn
Vaxholm, Baltic, Spring
Kalix, Baltic, Spring
chr21: 13.66 to 13.68 Mb
0%
50%
100%

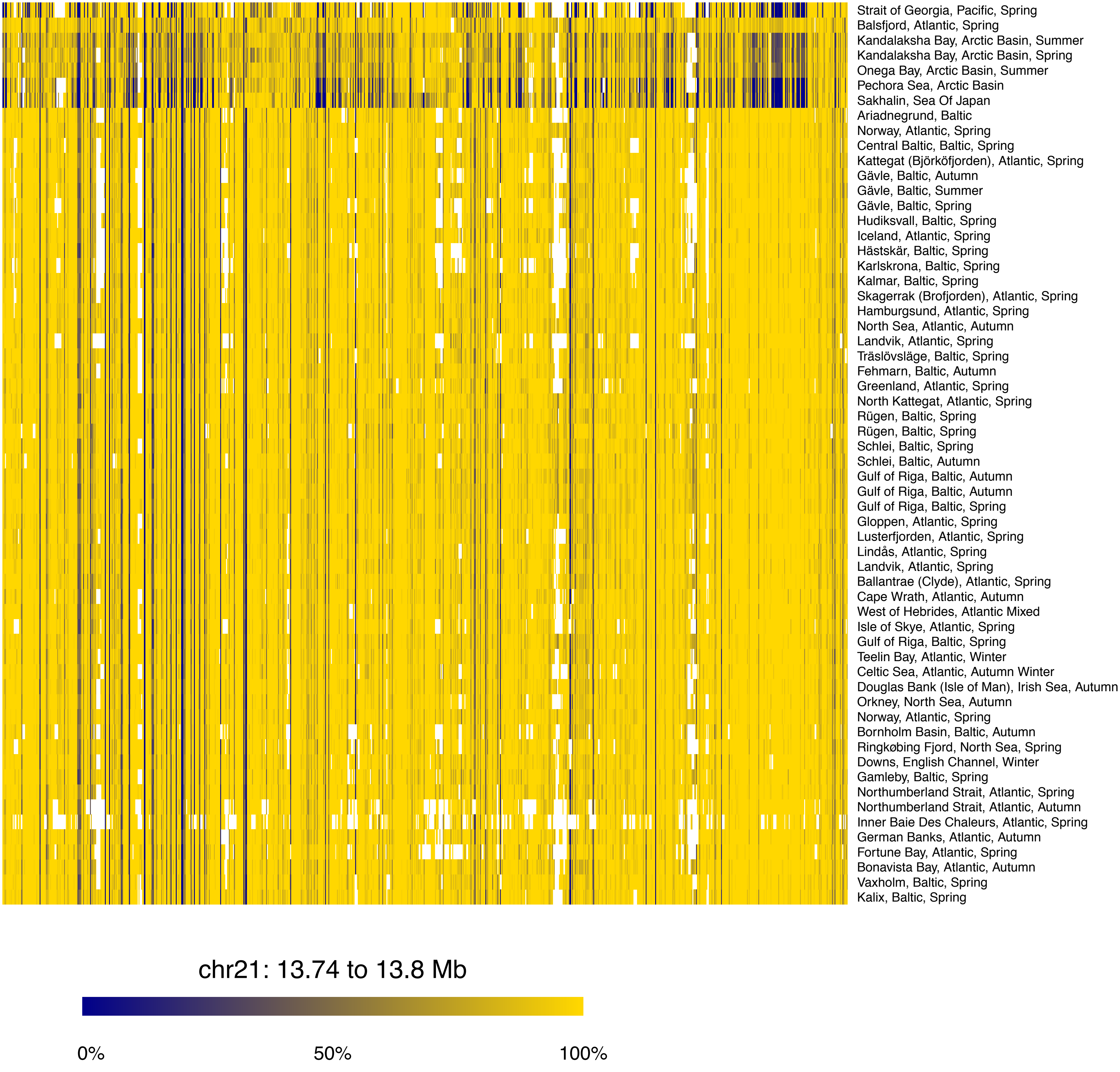
Strait of Georgia, Pacific, Spring
Balsfjord, Atlantic, Spring
Kandalaksha Bay, Arctic Basin, Summer
Kandalaksha Bay, Arctic Basin, Spring
Onega Bay, Arctic Basin, Summer
Pechora Sea, Arctic Basin
Sakhalin, Sea Of Japan
Ariadnegrund, Baltic
Norway, Atlantic, Spring
Central Baltic, Baltic, Spring
Kattegat (Björköfjorden), Atlantic, Spring
Gävle, Baltic, Autumn
Gävle, Baltic, Summer
Gävle, Baltic, Spring
Hudiksvall, Baltic, Spring
Iceland, Atlantic, Spring
Hästskär, Baltic, Spring
Karlskrona, Baltic, Spring
Kalmar, Baltic, Spring
Skagerrak (Brofjorden), Atlantic, Spring
Hamburgsund, Atlantic, Spring
North Sea, Atlantic, Autumn
Landvik, Atlantic, Spring
Träslövsläge, Baltic, Spring
Fehmarn, Baltic, Autumn
Greenland, Atlantic, Spring
North Kattegat, Atlantic, Spring
Rügen, Baltic, Spring
Rügen, Baltic, Spring
Schlei, Baltic, Spring
Schlei, Baltic, Autumn
Gulf of Riga, Baltic, Autumn
Gulf of Riga, Baltic, Autumn
Gulf of Riga, Baltic, Spring
Gloppen, Atlantic, Spring
Lusterfjorden, Atlantic, Spring
Lindås, Atlantic, Spring
Landvik, Atlantic, Spring
Ballantrae (Clyde), Atlantic, Spring
Cape Wrath, Atlantic, Autumn
West of Hebrides, Atlantic Mixed
Isle of Skye, Atlantic, Spring
Gulf of Riga, Baltic, Spring
Teelin Bay, Atlantic, Winter
Celtic Sea, Atlantic, Autumn Winter
Douglas Bank (Isle of Man), Irish Sea, Autumn
Orkney, North Sea, Autumn
Norway, Atlantic, Spring
Bornholm Basin, Baltic, Autumn
Ringkøbing Fjord, North Sea, Spring
Downs, English Channel, Winter
Gamleby, Baltic, Spring
Northumberland Strait, Atlantic, Spring
Northumberland Strait, Atlantic, Autumn
Inner Baie Des Chaleurs, Atlantic, Spring
German Banks, Atlantic, Autumn
Fortune Bay, Atlantic, Spring
Bonavista Bay, Atlantic, Autumn
Vaxholm, Baltic, Spring
Kalix, Baltic, Spring
chr21: 13.74 to 13.8 Mb
0%
50%
100%

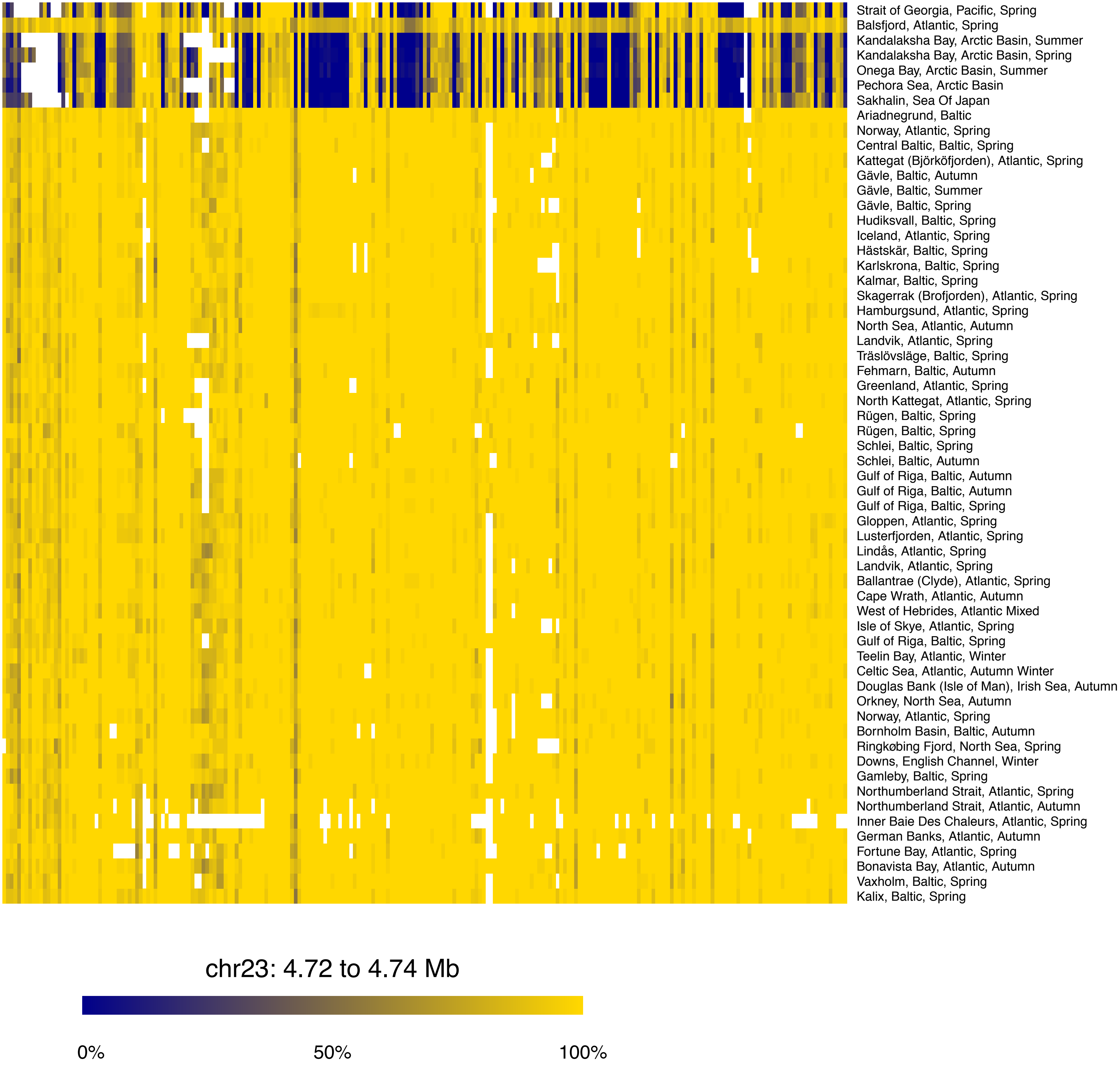
Strait of Georgia, Pacific, Spring
Balsfjord, Atlantic, Spring
Kandalaksha Bay, Arctic Basin, Summer
Kandalaksha Bay, Arctic Basin, Spring
Onega Bay, Arctic Basin, Summer
Pechora Sea, Arctic Basin
Sakhalin, Sea Of Japan
Ariadnegrund, Baltic
Norway, Atlantic, Spring
Central Baltic, Baltic, Spring
Kattegat (Björköfjorden), Atlantic, Spring
Gävle, Baltic, Autumn
Gävle, Baltic, Summer
Gävle, Baltic, Spring
Hudiksvall, Baltic, Spring
Iceland, Atlantic, Spring
Hästskär, Baltic, Spring
Karlskrona, Baltic, Spring
Kalmar, Baltic, Spring
Skagerrak (Brofjorden), Atlantic, Spring
Hamburgsund, Atlantic, Spring
North Sea, Atlantic, Autumn
Landvik, Atlantic, Spring
Träslövsläge, Baltic, Spring
Fehmarn, Baltic, Autumn
Greenland, Atlantic, Spring
North Kattegat, Atlantic, Spring
Rügen, Baltic, Spring
Rügen, Baltic, Spring
Schlei, Baltic, Spring
Schlei, Baltic, Autumn
Gulf of Riga, Baltic, Autumn
Gulf of Riga, Baltic, Autumn
Gulf of Riga, Baltic, Spring
Gloppen, Atlantic, Spring
Lusterfjorden, Atlantic, Spring
Lindås, Atlantic, Spring
Landvik, Atlantic, Spring
Ballantrae (Clyde), Atlantic, Spring
Cape Wrath, Atlantic, Autumn
West of Hebrides, Atlantic Mixed
Isle of Skye, Atlantic, Spring
Gulf of Riga, Baltic, Spring
Teelin Bay, Atlantic, Winter
Celtic Sea, Atlantic, Autumn Winter
Douglas Bank (Isle of Man), Irish Sea, Autumn
Orkney, North Sea, Autumn
Norway, Atlantic, Spring
Bornholm Basin, Baltic, Autumn
Ringkøbing Fjord, North Sea, Spring
Downs, English Channel, Winter
Gamleby, Baltic, Spring
Northumberland Strait, Atlantic, Spring
Northumberland Strait, Atlantic, Autumn
Inner Baie Des Chaleurs, Atlantic, Spring
German Banks, Atlantic, Autumn
Fortune Bay, Atlantic, Spring
Bonavista Bay, Atlantic, Autumn
Vaxholm, Baltic, Spring
Kalix, Baltic, Spring
chr23: 4.72 to 4.74 Mb
0%
50%
100%
